# Atomically Precise Bismuth Oxido Nanoclusters as Hosts for Ln^3+^: Effects of Doping on Optical and Magnetic Properties of a Soluble Metal Oxide

**DOI:** 10.1021/acs.inorgchem.6c01410

**Published:** 2026-06-19

**Authors:** Rico Thomas, Senthil Kumar Kuppusamy, Tobias Rüffer, Marcus Weber, Vanessa Stephan, Florian Taube, Andrei Kuzhelev, Björn Corzilius, Berthold Kersting, Mario Ruben, Michael Mehring

**Affiliations:** † Faculty of Natural Sciences, Institute of Chemistry, Coordination Chemistry, 38869 Chemnitz University of Technology, Straße der Nationen 62, Chemnitz 09107, Germany; ‡ Karlsruhe Institute of Technology (KIT), 150232 Institute for Quantum Materials and Technologies (IQMT), Karlsruhe 76131, Germany; § Faculty of Natural Sciences, Institute of Chemistry, Inorganic Chemistry, Chemnitz University of Technology, Straße der Nationen 62, Chemnitz 09107, Germany; ∥ Center of Materials, Architectures and Integration of Nanomembranes, Chemnitz University of Technology, Rosenbergstraße 6, Chemnitz 09126, Germany; ⊥ 9180 University of Leipzig, Faculty of Chemistry and Mineralogy, Institute of Inorganic Chemistry, Leipzig 04103, Germany; # Institute of Chemistry and Department Life, Light & Matter, 98914 University of Rostock, Albert-Einstein-Straße 27, Rostock 18059, Germany; ∇ Goethe University Frankfurt, Institute of Physical and Theoretical Chemistry and Center for Biomolecular Magnetic Resonance (BMRZ), Max von Laue Str. 7, Frankfurt am Main 60438, Germany; ○ Leibniz Institute for Catalysis (LIKAT e.V.), Albert-Einstein-Straße 29, Rostock 18059, Germany; ◆ Karlsruhe Institute of Technology (KIT), Institute of Nanotechnology (INT), Karlsruhe 76131, Germany; ¶ Centre Européen de Sciences Quantiques (CESQ), Institut de Science et d’Ingénierie Supramoléculaires (ISIS), Strasbourg 67083, France

## Abstract

A series of lanthanoid-doped bismuth oxido nanoclusters (BiO-NCs) of the type [Bi_38_O_45_(NO_3_)_24_(dmso)_*y*_]:Ln (**C-1:Ln**; Ln^3+^ = La – Lu, ≠ Pm; *y* = 26–28) including isostructural host and doped structures is reported. Successful doping with ≈1 ω% of the BiO-NCs was demonstrated by ICP-OES, ESI-MS, and exemplarily by SC XRD analysis for **C-1:Gd** and **C-1:Dy**. The dopants are statistically albeit nonuniformly distributed on the lattice position of Bi^3+^ and change the optical and magnetic properties of the BiO-NC host material. Altered optical properties (UV–vis and PL) were demonstrated with special focus on **C-1:Er**, **C-1:Yb**, and **C-2**_**d**_ **:Dy** showing dual Bi^3+^ and the Ln^3+^ specific PL emissions, resulting in characteristic emission combinations over a wide wavelength range from visible to the NIR region and lifetimes up to 0.5 ms. Starting from **C-1:Gd** and **C-1:Dy** the methacrylate-substituted BiO-NCs [Bi_38_O_45_(OMc)_24_(EtOH)_14_]:Gd (**C-2**_**E**_ **:Gd**) and [Bi_38_O_45_(OMc)_24_(EtOH)_14_]:Dy (**C-2**_**E**_ **:Dy**) were synthesized to finally give solvate-free [Bi_38_O_45_(OMc)_24_]:Ln (**C-2**_**d**_ **:Gd**, **C-2**_**d**_ **:Dy**) after drying. The introduction of paramagnetic behavior in the BiO-NC diamagnetic host structure was confirmed by SQUID measurements. We further investigated the suitability of Gd^3+^ in **C-2**_**d**_ **:Gd** as a polarization agent for dynamic nuclear polarization (DNP)-enhanced MAS NMR experiments, which resulted in a proton enhancement factor of approximately 10.

## Graphical abstract

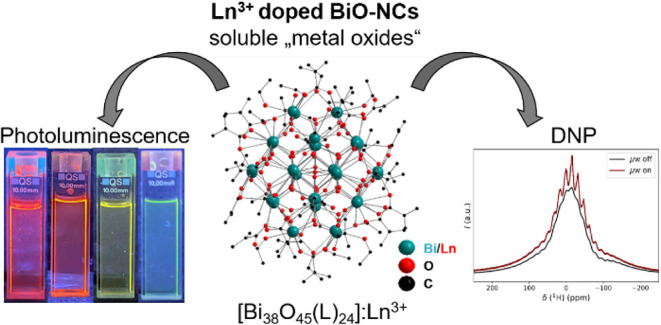

## Introduction

Atomically precise nanoclusters (NCs) are a rapidly emerging class of compounds representing the link between molecules and nanoparticles (NPs). They are characterized by their monodisperse nature with a size below 3 nm as well as their atomically precise structure.^1,2^ Their NC structure can be determined by characterization methods such as SC XRD, nuclear magnetic resonance spectroscopy (NMR), and various mass spectrometry (MS) methods, complementing methods that are typically used for NPs. Their monodisperse size distribution, uniform shape, and precise structure provide these NCs with well-defined chemical and physical properties. Therefore, atomically precise NCs are suitable for accurately characterizing the influence of structural changes in both the cluster core and the ligand periphery and thus for analyzing the resulting changes in their properties. NCs have been mostly described for metals and metalloids, but interest in metal chalcogenido clusters, primarily with Se and S, has also risen in the past.^3–6^ Within the family of oxygen-bridged NCs, the unique class of polyoxometalates (POMs) has gained much attention. POMs consist of a negatively charged cluster core, described for a variety of oxygen-bridged metal atoms (Mo, W, V, Nb, lanthanoids, for example), and various cationic counterions.^7–9^ Much less attention has been given to soluble cationic and neutral metal oxido nanoclusters (MO-NCs), which often constitute a cutout of the associated metal oxide structure.^10–14^

Among the MO-NCs, bismuth oxido nanoclusters (BiO-NCs) are of particular significance due to their structural diversity, their low toxicity, and their potential applications in fields such as medicine, photolithography, energy storage, rewritable resistive memory devices, or as transparent radiopaque materials and photocatalysts.^15–21^ BiO-NCs with a so-called “magic size” {Bi_38_O_45_} core are outstanding in this material class, as by far the most BiO-NCs reported show up with the general composition [Bi_38_O_45_(L)_24_] (e.g., L = carboxylate, nitrate, sulfonate).^11,16,22–27^ These BiO-NCs are obtained by the simultaneous hydrolysis and condensation of Bi­(NO_3_)_3_·5H_2_O or hexanuclear clusters (e.g., [Bi_6_O_4_(OH)_4_(NO_3_)_5_H_2_O]­NO_3_) in the presence of additional ligands L.^28^ The growth process was recently traced back by *in situ* investigations using ESI-MS and combined pair distribution function (PDF) and small-angle X-ray scattering (SAXS) analysis and demonstrated the stability of the {Bi_38_O_45_} motif.^29,30^ *In situ* and *ex situ* studies have demonstrated that ligand substitution can be achieved while preserving the {Bi_38_O_45_} core.^29^

Recently we were able to synthesize cerium-doped BiO-NCs based on the {Bi_38_O_45_} core by the simultaneous hydrolysis of bismuth­(III) nitrate and cerium­(III) nitrate.^31^ It has been shown that doping with about ≈1 ω% strongly influences the electronic and optical properties of the BiO-NCs and that changing the ligands at the cluster surface affects the oxidation state of the cerium in the {Bi_38_O_45_}:Ce core.^32^ The characteristic properties of the doped BiO-NCs can be summarized as follows: (i) undoped and doped clusters are isostructural, (ii) Bi^3+^ positions are partially and statistically, albeit nonuniformly occupied by the dopant, (iii) due to the low percentage of doping, the bulk material of the BiO-NCs is a nonseparable mixture of undoped and differently doped nanoclusters, and (iv) the properties of the doped material are strongly affected by the low doping content. It should be noted that these doped clusters should be distinguished from recently reported heterobimetallic or multimetallic MO-NCs, which show inherently different structures of the homometallic and heterobimetallic clusters.^33–37^

Noteworthy, in a field-specific article on metal clusters a critical discussion of the terms “doped” and “alloyed” MCs was published recently.^38^ The authors highlighted the point that a cluster is called “doped” when one or multiple atoms of an existing cluster (required) are replaced by another metal. Even in the huge field of MCs, only a few structures fulfill this criterion, and examples are mostly based on Au or Ag host cluster structures with transition metals such as Pt or Pd as dopant.^38–42^

Lanthanoid cations (Ln^3+^) as dopants in diverse solid-state materials have found applications in light-emitting diodes, displays, lasers, fibers, anticounterfeiting tags, magnetic resonance imaging, and magnetic materials.^7,43,44^ With regard to optical properties, it is appealing that Ln^3+^ ions exhibit strong absorption in various regions of the electromagnetic spectrum. Yb^3+^ and Er^3+^ absorb in the near-infrared region, while Nd^3+^, Pr^3+^, Er^3+^, Eu^3+^, and Sm^3+^ absorb in the visible part of the spectrum, whereas La^3+^ and Lu^3+^ are limited to the UV range. Certain lanthanoid cations, namely, Eu^3+^, Dy^3+^, Tb^3+^, Yb^3+^, Er^3+^, and Sm^3+^, are known to exhibit strong fluorescence, making them suitable for applications in the above-mentioned optical devices.^7,43,44^ The photoluminescent properties vary significantly over a wide spectral range, which can be used for upconversion luminescence extending the applications toward sensing, imaging, and biomedicine.^45–49^ NIR emitters showing high penetration ability and signal-to-noise ratio are further promising for quantum and satellite communication, data storage, or night vision.^50–53^ The majority of Ln^3+^ cations have unpaired electrons, resulting in a paramagnetic moment; the highest among them show Dy^3+^, Ho^3+^, Er^3+^, Tb^3+^, and Gd^3+^.^45^ Thus, they are promising candidates for ferromagnetic materials, single-molecule magnets, or contrast agents in magnetic resonance imaging.^43,54–57^ Gd^3+^ also plays a special role due to its electron paramagnetic resonance (EPR) properties, which make it an ideal polarization agent (PA) in dynamic nuclear polarization (DNP)-enhanced magic-angle spinning nuclear magnetic resonance (MAS NMR) experiments at high fields.^58–60^

Here a general synthetic approach to various doped BiO-NCs with a {Bi_38_O_45_}:Ln core is reported and the impact of lanthanoid doping on BiO-NCs with a focus on optical and magnetic properties demonstrated. Thus, the doped BiO-NCs were characterized using UV–vis and photoluminescence (PL) spectroscopy, and the magnetic properties were analyzed using variable temperature magnetic susceptibility measurements with a superconducting quantum interference device (SQUID) and DNP, in addition to structural characterization.

## Experimental Section

### Materials Characterization

Powder X-ray diffractograms were measured at ambient temperature with a STOE *Stadi P* diffractometer (Darmstadt, Germany) using Ge(111)-monochromatized Cu–K_α_ radiation (λ = 1.54056 nm, 40 kV, 40 mA). The full width at half-maximum (fwhm) is corrected for instrumental broadening using a LaB_6_ standard (*SRM 660*) purchased from the National Institute of Standards and Technology (NIST). The value of β was corrected from β^2^ = β_measured_^2^ – β_instrument_^2^ where β_measured_ and β_instrument_ are the fwhm’s of the measured and standard profiles, respectively. Single-crystal X-ray diffraction was performed using a Bruker Venture D8 diffractometer including the APEX software package for **C-1′**, **C-1:Gd, C-1:Dy**, and **C-2**_**E**_ **:Gd**. ^1^H and ^13^C­{^1^H} NMR spectra were recorded at room temperature in CDCl_3_ (dried over 4 Å molecular sieve) with an *Avance III 500* spectrometer (Bruker) at 500.30 and 125.81 MHz, respectively, and are referenced internally to the deuterated solvent relative to Si­(CH_3_)_4_ (δ = 0.00 ppm). The NMR spectra were processed using the software *MestReNova*/version (11.0.418998). Solid-state NMR spectra were recorded at 9.4 T on an Avance 400 spectrometer (Bruker) equipped with double-tuned probes capable of magic-angle spinning (MAS). ^13^C­{^1^H} CP MAS NMR spectra were measured at 100.6 MHz in 3.2 mm standard zirconium oxide rotors (Bruker) spinning at 15 kHz. Cross-polarization (CP) with a contact time of 3 ms was used to enhance sensitivity, in some of the measurements. The recycle delay was 6 s. The spectra were referenced externally to Si­(CH_3_)_4_ (δ = 0.00 ppm) as well as to adamantane (δ = 38.48 ppm for ^13^C) as secondary standard. All spectra were collected with ^1^H decoupling using a two-pulse phase modulation sequence. DNP-enhanced MAS NMR experiments were carried out on a Bruker AVANCE III HD spectrometer with an operating field of 400.5 MHz proton frequency with a Bruker ASCEND DNP 9.4 T widebore (89 mm) magnet. 263.4 GHz microwaves were produced by a Bruker/CPI second-harmonic gyrotron operating at a field of 4.8 T with 139 mA beam current. All experiments were performed using rf powers of 100 kHz (^1^H) and 60 kHz (^13^C) for 3.2 mm probe operating in ^1^H/^13^C double-channel mode. During sample preparation the pure powder of **C-2**_**d**_ **:Gd** was filled into a 3.2 mm sapphire rotor using a Vespel drive cap. The temperature was detected via a thermocouple inside the MAS stator. ^1^H and ^13^C 90°-pulse power was determined in Hartmann–Hahn cross-polarization (CP) experiments with 1 ms contact time. SPINAL-64 was used for decoupling of ^1^H during acquisition. Magic-angle spinning (MAS) with a spinning frequency of 8 kHz was used for all experiments. A Lakeshore superconducting magnet power supply (model 625) was used to sweep the ASCEND DNP 9.4 T magnet. Enhancement factors were determined by simple amplitude comparison according to *ε* = I_on_/I_off_. CHNS analyses were performed with a Foss Heraeus *Vario EL* analyzer. Infrared spectra were recorded with a *Nicolet iS 5 FT-IR (Fourier transform infrared spectroscopy)* spectrometer (Thermo Scientific) with an *iD7 AR-coated diamond crystal ATR* accessory. Solid samples were pressed with 40 pounds onto the crystal. Spectra were recorded with 32 scans and a resolution of 4 cm^–1^ using the Omnic 9 software (version 9.8.372). UV–vis spectroscopy was performed using a *Cary 60 UV–vis* (Agilent Technologies) equipped with a *Barrelino* (Harrick Scientific Products) remote diffuse reflection probe. Therefore, one tip of a spatula of the well-ground BiO-NCs sample was placed into the sample window of the solid-state diffuse reflection probe (*Barrelino*), on top of the white background and was then gently bladed to give a plane surface. The *Barrelino* measuring probe was then carefully attached. Metal content analysis was conducted via inductively coupled plasma-optical emission spectroscopy (ICP-OES), with a VistaRL (Varian). 10 mg of the samples were dissolved in 5 mL nitric acid (65%) and diluted to 10 vol % acid with ultrapure water. Note that the amount was determined in double/multidetermination in order to reduce inaccuracies. Fluorescence measurements in the solid state were performed with a Horiba Jobin Yvon *FluoroMax* spectrofluorometer. Crystalline samples were introduced into an attached sample holder and the aperture was adjusted to a 5 nm slit width. Emission spectra were recorded at different excitation wavelengths (λ_ex._ = 492 nm (Pr^3+^), λ_ex._ = 403 nm (Sm^3+^), λ_ex._ = 368 nm (Tb^3+^), λ_ex._ = 462 nm (Ho^3+^), λ_ex._ = 725 nm (Tm^3+^). Photophysical studies of the undoped and Er^3+^, Yb^3+^, and Dy^3+^ doped BiO NCs were performed using a Horiba quantum master spectrometer with an R920 photomultiplier tube detector. For PL measurements in the solid state, about 2–3 mg of well-ground crystallites of Ln-doped clusters were placed between two quartz slides. A small drop of perfluorinated oil was smeared on the substrates for trapping the crystals. The as-prepared sample was mounted on a Sumitomo closed-cycle He-cryostat for temperature-dependent measurements in the range from 2.4 to 300 K. A 400 nm long-pass filter was used to cut out the second- and higher-order diffraction peaks in the spectra, in the case of **C-1:Yb** and **C-1:Er** a 850 nm long-pass filter was used to cut out the Bi^3+^-based second-order peaks.^1,2^ Correction files supplied by the manufacturer were used to obtain the corrected emission spectra. The data plotting and decay fitting were performed using Origin 24. PL Lifetimes were measured on excitation with an LED (λ_max._ = 319 nm), the decay data were collected for a few hours at each wavelength to obtain reasonably good data sets for fitting. Additionally, the lifetime values are close to the time resolution of the time-correlated single-photon counting (TCSPC) setup; therefore, the values need to be considered with caution. CIE coordinates were calculated using the CIE-coordinate app in Origin 24. ESI-MS was carried out using a Bruker Trapped Ion Mobility Spectrometry (tims) time-of-flight (TOF) mass spectrometer (Bruker Daltonik GmbH, Bremen, Germany) with a mass accuracy of <0.8 ppm (mass drift over 8 h with Δ*T* < 1 K: <2 ppm) and a mass resolution of 50,000 FSR (full sensitivity resolution @ 1221 *m*/*z*). Calibration was done in the *m*/*z* range of 100–10,000 using cesium perfluoroheptanoate (*c* = 5 mM in H_2_O/MeCN with *V*:*V* = 1:1, abcr GmbH). The powders of compounds **C-1:Ln** (Ln = La, Pr, Nd, Sm, Gd, Tb, Ho, Er, Tm, Yb, Lu) were dissolved in dmso (LC-MS grade, purity by titration: 99.7%, water content: ≤0.1%, Thermo Scientific , *V* = 1.5 mL) at 80 °C and diluted with MeCN (HiPerSolv Chromanorm, water content: <30 ppm, *V* = 3.5 mL) to give a final concentration of 100 μM (*V*:*V* = 3:7), respectively. The crystals of compound **C-2**_**E**_ **:Gd** and **C-2**_**E**_ **:Dy** were dissolved in isopropanol (Ultra LC-MS grade, Carl Roth) with a final concentration of 100 μM, respectively. The sample was injected into the ESI source using a Hamilton syringe (*V* = 500 μL) at a flow rate of 180 μL·h^–1^. The voltage of the spray capillary was set to 4.5 kV (positive mode) with a deflection delta of 70.0 V and an end plate offset voltage of 500 V. Mass spectra were treated by smoothing (Savitzky–Golay algorithm, width 0.05 *m*/*z*) and isotope patterns were calculated using Bruker Compass DataAnalysis software (Copyright © 2023 Bruker Daltonik GmbH & Co. KG, version 6.1). The most abundant *m*/*z* signals were used for assignment. The magnetic susceptibility measurements were performed with an MPMS 7XL SQUID magnetometer (Quantum Design). Respective data were collected in the temperature range of 2 to 300 K in an applied *dc* field of 0.05 T. The powdered samples were grounded and prepared in a gelatin capsule. The measured susceptibility data were corrected based on the diamagnetic BiO-NC. The data were processed with DAVE.^3^ Pulsed EPR experiments were performed at a frequency of 180 GHz and a temperature of 10 K using a home-built G-band EPR spectrometer.^4^ For these experiments, the **C-2**_**d**_ **:Gd** powder was transferred into a quartz capillary with an inner diameter of 0.2 mm and an outer diameter of 0.33 mm. The electron spin relaxation times *T*_2_ and *T*_1_ were measured using Hahn-echo decay and inversion recovery experiments, respectively. The pulse lengths were 16 and 22 ns for π/2 and π, respectively, and the pulse separation time was set to 150 ns. The shot repetition time was 20 ms, with 50 shots per point. The relaxation times were measured at the field corresponding to the maximum of the respective echo-detected field sweep EPR spectrum.

## Materials and Methods

### Chemicals

Bi­(NO_3_)_3_ **·**5H_2_O (98%), Gd­(NO_3_)_3_ **·**6H_2_O, Dy­(NO_3_)_3_ **·**5H_2_O, Er­(NO_3_)_3_ **·**5H_2_O, La­(NO_3_)_3_ **·**6H_2_O, Nd­(NO_3_)_3_ **·**6H_2_O, Sm­(NO_3_)_3_ **·**6H_2_O, Tb­(NO_3_)_3_ **·** *n*H_2_O, Ho­(NO_3_)_3_ **·**6H_2_O (all, 99.9%), Tm­(NO_3_)_3_ **·**5H_2_O, Lu­(NO_3_)_3_ **·** *n*H_2_O (99.99%) were purchased from Alfa Aesar. Pr­(NO_3_)_3_ **·**6H_2_O (99.99%) was purchased from Thermo Scientific, and Yb­(NO_3_)_3_ **·**5H_2_O (99.9%) from Sigma-Aldrich. Sodium methacrylate (NaOMc, 99%) was purchased from Sigma-Aldrich, sodium hydroxide (99%) from Merck. All chemicals were used without further purification. The solvent dmso (99.9%, Fisher Chemicals) and ethanol (99.9%, Alfa Aesar) were used without further purification. *Iso*-propanol (99.95%, MS grade) from Carl Roth, dmso (99.5%, MS grade) from Thermo Scientific, and acetonitrile (99.98%, MS grade) from Carl Roth were used without further purification for the ESI-MS measurements.

[Bi_38_O_45_(NO_3_)_20_(dmso)_28_]­(NO_3_)_4_ **·**4dmso (**C-1**),^5^ [Bi_38_O_45_(OMc)_24_(dmso)_9_]·2dmso·7H_2_O (**C-2**),^6^ and [Bi_38_O_45_(OMc)_24_(EtOH)_13_] (**C-2**_**E**_),^7^ [Bi_38_O_45_(NO_3_)_24_(dmso)_28_]:Ce (**C-1:Ce**),^8^ and [Bi_38_O_45_(OMc)_24_(dmso)_4_]:Eu·4dmso·4H_2_O (**C-1:Eu**)^9^ were synthesized according to the literature procedures. For their detailed characterization we refer to the original publications.

### Synthesis of [Bi_38_O_45_(NO_3_)_24_(dmso)_28–_ *_y_*]:Ln (**C-1:Ln**)

Bi­(NO_3_)_3_ **·**5H_2_O (1504 mg, 3.1 mmol) and Ln­(NO_3_)_3_ **·** *n*H_2_O (*m*_1_, 0.31 mmol; Ln and *n*, see Table S1) were dissolved with vigorous stirring in dmso (70 mL) under ambient conditions. Subsequently an aqueous NaOH solution (0.31 M, 12 mL) was added dropwise to the solution avoiding turbidity. The reaction mixture was heated to 80 °C for 4 h, followed by a filtration of the hot solution. Diffusion of acetone vapor in the filtered reaction mixture results in the formation of colorless crystals (*m*_2_, η based on bismuth) after 4 weeks (Scheme S1). Leaving the crystals under air conditions results in partial loss of DMSO and the formation of a microcrystalline powder.

### [Bi_38_O_45_(NO_3_)_24_(dmso)_28_]:La **(C-1:La)**

Colorless crystals, Elemental analysis CHNS%, exp. and (calcd.) for **C-1:La** (Bi_37.4_La_0.6_O_145_N_24_C_56_H_168_S_28_, *M* = 12295.02 g·mol^–1^): C, 5.51 (5.47); H, 1.46 (1.38); N, 2.78 (2.73); S, 7.13 (7.30). ICP-OES (ω%, exp./calcd.): La, 0.63–0.73 (0.68); Bi/La = 1:0.015–0.019. ATR-IR (cm^–1^): 3700–3100 w, 3002 w, 2917 w, 1741 w, 1646 w, 1432 m, 1378 s, 1264 s, 997 s, 945 s, 921 m, 815 m, 710 m, 480 s.

### [Bi_38_O_45_(NO_3_)_24_(dmso)_28_]:Pr **(C-1:Pr)**

Colorless to Pale green crystals, Elemental analysis CHNS%, exp. and (calcd.) for **C-1:Pr** (Bi_37.27_Pr_0.73_O_145_N_24_C_56_H_168_S_28_, *M* = 12287.37 g·mol^–1^): C, 5.37 (5.47); H, 1.42 (1.38); N, 2.78 (2.73); S, 7.02 (7.30). ICP-OES (ω%, exp./calcd.): Pr, 0.82–0.86 (0.84); Bi/Pr = 1:0.021. ATR-IR (cm^–1^): 3700–3100 w, 3003 w, 2917 w, 1741 w, 1636 w, 1424 m, 1377 s, 1264 s, 997 s, 945 s, 921 m, 814 m, 710 m, 485 s.

### [Bi_38_O_45_(NO_3_)_24_(dmso)_28_]:Nd (**C-1:Nd**)

Colorless crystals, Elemental analysis CHNS%, exp. and (calcd.) for **C-1:Nd** (Bi_37.23_Nd_0.77_O_145_N_24_C_56_H_168_S_28_, *M* = 12287.21 g·mol^–1^): C, 5.33 (5.47); H, 1.41 (1.38); N, 2.78 (2.74); S, 6.97 (7.31). ICP-OES (ω%, exp./calcd.): Nd, 0.89–0.90 (0.90); Bi/Nd = 1:0.022–0.024. ATR-IR (cm^–1^): 3700–3100 w, 3003 w, 2917 w, 1741 w, 1653 w, 1429 m, 1378 s, 1264 s, 998 s, 945 s, 922 m, 815 m, 710 m, 491 s.

### [Bi_38_O_45_(NO_3_)_24_(dmso)_28_]:Sm (**C-1:Sm**)

Colorless crystals, Elemental analysis CHNS%, exp. and (calcd.) for **C-1:Sm** (Bi_37.36_Sm_0.64_O_145_N_24_C_56_H_168_S_28_, *M* = 12299.54 g·mol^–1^): C, 5.57 (5.47); H, 1.48 (1.38); N, 2.72 (2.74); S, 7.23 (7.31). ICP-OES (ω%, exp./calcd.): Sm, 0.71–0.84 (0.78); Bi/Sm = 1:0.017–0.020. ATR-IR (cm^–1^): 3700–3100 w, 3003 w, 2917 w, 1741 w, 1653 w, 1429 m, 1378 s, 1264 s, 997 s, 945 s, 921 m, 815 m, 710 m, 486 s.

### [Bi_38_O_45_(NO_3_)_24_(dmso)_26_]:Gd (**C-1:Gd**)

Colorless crystals, Elemental analysis CHNS%, exp. and (calcd.) for **C-1:Gd** (Bi_37.25_Gd_0.75_O_145_N_24_C_56_H_168_S_28_, *M* = 12287.14 g·mol^–1^): C, 5.53 (5.47); H, 1.39 (1.38); N, 2.54 (2.74); S, 7.26 (7.31). ICP-OES (ω%, exp./calcd.): Gd, 0.84–1.08 (0.96.); Bi/Gd = 1:0.017–0.019. ATR-IR (cm^–1^): 3700–3100 w, 3004 w, 2917 w, 1741 w, 1637 w, 1430 m, 1378 s, 1266 s, 1001 s, 946 s, 923 s, 814 m, 710 m, 488 s.

### [Bi_38_O_45_(NO_3_)_24_(dmso)_28_]:Tb (**C-1:Tb**)

Colorless crystals, Elemental analysis CHNS%, exp. and (calcd.) for **C-1:Tb** (Bi_37.33_Tb_0.67_O_143_N_24_C_52_H_156_S_26_, *M* = 12224.88 g·mol^–1^): C, 5.26 (5.14); H, 1.40 (1.29); N, 2.87 (2.77); S, 6.84 (6.86). ICP-OES (ω%, exp./calcd.): Tb, 0.87–0.89 (0.88); Bi/Tb = 1:0.019. ATR-IR (cm^–1^): 3700–3100 w, 3003 w, 2917 w, 1741 w, 1646 w, 1425 m, 1378 s, 1264 s, 997 s, 944 s, 921 m, 814 m, 710 m, 480 s.

### [Bi_38_O_45_(NO_3_)_24_(dmso)_28_]:Dy (**C-1:Dy**)

Colorless crystals, Elemental analysis CHNS%, exp. and (calcd.) for **C-1:Dy** (Bi_37.31_Dy_0.69_O_145_N_24_C_56_H_168_S_28_, *M* = 12304.98 g·mol^–1^): C, 5.51 (5.47); H, 1.40 (1.38); N, 2.58 (2.73); S, 7.29 (7.30). ICP-OES (ω%, exp./calcd.): Dy, 0.84–0.97 (0.91); Bi/Dy = 1:0.018–0.019. ATR-IR (cm^–1^): 3700–3100 w, 3002 w, 2917 w, 1741 w, 1637 w, 1429 m, 1378 s, 1265 s, 998 s, 945 s, 922 s, 815 m, 709 m, 479 s.

### [Bi_38_O_45_(NO_3_)_24_(dmso)_28_]:Ho (**C-1:Ho**)

Colorless to Pale red crystals, Elemental analysis CHNS%, exp. and (calcd.) for **C-1:Ho** (Bi_37.36_Ho_0.64_O_144_N_24_C_56_H_162_S_27_, *M* = 12230.73 g·mol^–1^): C, 5.32 (5.30); H, 1.34 (1.34); N, 2.47 (2.75); S, 7.01 (7.08). ICP-OES (ω%, exp./calcd.): Ho, 0.85–0.87 (0.86); Bi/Ho = 1:0.017–0.018. ATR-IR (cm^–1^): 3700–3100 w, 3005 w, 2917 w, 1741 w, 1635 w, 1423 m, 1377 s, 1264 s, 998 s, 945 s, 922 m, 813 m, 710 m, 486 s.

### [Bi_38_O_45_(NO_3_)_24_(dmso)_28_]:Er (**C-1:Er**)

Colorless to Pale red crystals, Elemental analysis CHNS%, exp. and (calcd.) for **C-1:Er** (Bi_37.41_Er_0.59_O_145_N_24_C_56_H_168_S_28_, *M* = 12212.44 g·mol^–1^): C, 5.29 (5.46); H, 1.38 (1.38); N, 2.59 (2.73); S, 6.93 (7.29). ICP-OES (ω%, exp./calcd.): Er, 0.76–0.86 (0.81); Bi/Er = 1:0.017–0.021. ATR-IR (cm^–1^): 3700–3100 w, 3002 w, 2917 w, 1741 w, 1646 w, 1429 m, 1378 s, 1264 s, 997 s, 945 s, 922 m, 814 m, 710 m, 478 s.

### [Bi_38_O_45_(NO_3_)_24_(dmso)_28_]:Tm (**C-1:Tm**)

Colorless crystals, Elemental analysis CHNS%, exp. and (calcd.) for **C-1:Tm** (Bi_37.57_Tm_0.43_O_143_N_24_C_52_H_156_S_26_, *M* = 12165.97 g·mol^–1^): C, 5.05 (5.13); H, 1.34 (1.29); N, 2.76 (2.77); S, 6.56 (6.85). ICP-OES (ω%, exp./calcd.): Tm, 0.58–0.60 (0.59); Bi/Tm = 1:0.012–0.013. ATR-IR (cm^–1^): 3700–3100 w, 3003 w, 2917 w, 1742 w, 1653 w, 1428 m, 1378 s, 1264 s, 998 s, 945 s, 922 m, 815 m, 710 m, 486 s.

### [Bi_38_O_45_(NO_3_)_24_(dmso)_28_]:Yb (**C-1:Yb**)

Colorless crystals, Elemental analysis CHNS%, exp. and (calcd.) for **C-1:Yb** (Bi_37.43_Yb_0.57_O_145_N_24_C_56_H_168_S_28_, *M* = 12316.57 g·mol^–1^): C, 5.51 (5.46); H,1.41 (1.38); N, 2.53 (2.73); S, 7.19 (7.29). ICP-OES (ω%, exp./calcd.): Yb, 0.72–0.88 (0.80); Bi/Yb = 1:0.015–0.016. ATR-IR (cm^–1^): 3700–3100 w, 3003 w, 2917 w, 1740 w, 1647 w, 1433 m, 1383 s, 1266 s, 998 s, 943 s, 925 m, 816 m, 708 m, 481 s.

### [Bi_38_O_45_(NO_3_)_24_(dmso)_28_]:Lu (**C-1:Lu**)

Colorless crystals, Elemental analysis CHNS%, exp. and (calcd.) for **C-1:Lu** (Bi_37.32_Lu_0.68_O_145_N_24_C_56_H_168_S_28_, *M* = 12323.79 g·mol^–1^): C, 5.61 (5.46); H, 1.45 (1.38); N, 2.90 (2.73); S, 7.33 (7.29). ICP-OES (ω%, exp./calcd.): Lu, 0.52–0.59 (0.55); Bi/Lu = 1:0.010–0.012. ATR-IR (cm^–1^): 3700–3100 w, 3003 w, 2917 w, 1741 w, 1646 w, 1429 m, 1378 s, 1264 s, 998 s, 945 s, 922 m, 815 m, 710 m, 485 s.

### Synthesis of [Bi_38_O_45_(OMc)_24_(EtOH)_14_]:Gd (**C-2_E_:Gd**)

**[Bi**_**38**_ **O**_**45**_ **(NO**_**3**_ **)**_**24**_ **(dmso)**_**28**_ **]:** **Gd** (**C-1:Gd**, 1.650 g, 0.134 mmol) was dissolved in dmso (25 mL) under stirring at 80 °C for 1 h. NaOMc (522 mg, 4.83 mmol) was added to the colorless solution and the mixture was kept for 4 h at 80 °C. After 2 weeks, colorless crystals of [Bi_38_O_45_(OMc)_24_(dmso)_*x*_]:Gd (0.91 g) were obtained. [Bi_38_O_45_(OMc)_24_(dmso)_*x*_]:Gd (0.75 g) was dissolved in 8 mL ethanol under stirring at 60 °C for 1.5 h. The colorless dispersion was filtrated subsequently and first allowed to cool to room temperature, and than cooled to 4 °C. After 2 days, colorless crystals of [Bi_38_O_45_(OMc)_24_(EtOH)_14_]:Gd (**C-2**_**E**_ **:Gd**, 308 mg, 0.029 mmol, *η =* 21.5% based on the full amount of **C-1:Gd**) were obtained. For the comparable analysis, the crystals **C-2**_**E**_ **:Gd** were collected and dried in the vacuum at 60 °C for 1 h to remove the EtOH and to give **C-2**_**d**_ **:Gd**.

Elemental analysis CHNS%, exp. and (calcd.) for **C-2**_**d**_ **:Gd** (Bi_37.39_Gd_0.61_O_93_C_96_H_120_, based on EA, *M =* 10671.78 g·mol^–1^): C 10.70 (10.81); H 1.44 (1.13). ICP-OES **C-2**_**d**_ **:Gd** (ω%, expt/calcd): Gd, 0.84–0.89 (0.87); Bi/Gd = 1:0.018–0.019. ^1^H NMR (500.30 MHz, CDCl_3_, 298 K, ppm): *δ =* 6.6–4.6 (broad, 2 H, CH^A^ *H* ^B^, fwhm 0.9 ppm), 4.35 (s, EtOH, OH), 3.43 (2 H, EtOH (CH_2_)), 2.4–1.1 (broad, s, 3 H, CH_3_, fwhm 0.5 ppm), 1.05 (q, EtOH (CH_3_)).^13^C NMR (125.81 MHz, dmso-d_6_, 300 K): *δ =* 121.3, 56.0 (EtOH), 19.7, 18.6 (EtOH). ATR-FTIR (cm^–1^): 3700–3100 w, 3091 w, 2969 w, 2921 w, 1640 w, 1504 s, 1449 m, 1399 s, 1378 s, 1361 s,1228 s, 1044 w, 1001 m, 927 m, 830 m, 657 w, 473 s.

### Synthesis of [Bi_38_O_45_(OMc)_24_(EtOH)_14_]:Dy (**C-2_E_:Dy**)

**[Bi**_**38**_ **O**_**45**_ **(NO**_**3**_ **)**_**24**_ **(dmso)**_**28**_ **]:**Dy (**C-1:Dy**, 2.000 g, 0.200 mmol) was dissolved in dmso (35 mL) under stirring at 80 °C for 1 h. NaOMc (633 mg, 5.864 mmol) was added to the colorless solution and the mixture was kept for 4 h at 80 °C. After 2 weeks, colorless crystals of [Bi_38_O_45_(OMc)_24_(dmso)_*x*_]:Dy (1.35 g) were obtained. [Bi_38_O_45_(OMc)_24_(dmso)_*x*_]:Dy (0.71 g) was dissolved in 7.5 mL ethanol under stirring at 60 °C for 1.5 h. The dispersion was filtered subsequently and first allowed to cool to room temperature, and second cooled to 4 °C. After 2 days, colorless crystals of [Bi_38_O_45_(OMc)_24_(EtOH)_14_]:Dy (**C-2**_**E**_ **:Dy**, 284 mg, 0.133 mmol, *η =* 25.3% based on the full amount of **C-1:Dy**) were obtained. The crystals **C-2**_**E**_ **:Dy** were collected and dried in the vacuum at 60 °C for 1 h to remove the EtOH and to give **C-2**_**d**_ **:Dy**.

Elemental analysis CHNS%, exp. and (calcd.) for **C-2**_**d**_ **:Dy** (Bi_37.39_Dy_0.61_O_93_C_96_H_120_, *M =* 10674.83 g·mol^–1^): C 10.72 (10.80); H 1.45 (1.13). ICP-OES **C-2**_**d**_ **:Dy** (ω%, exp./calcd.): Dy, 0.92–0.94 (0.93); Bi/Dy = 1:0.018–0.019. ^1^H NMR (500.30 MHz, DMSO, 298 K, ppm): *δ =* 5.81 (broad, s, 1 H, CH^A^ *H* ^B^,fwhm 0.4 ppm), 5.30 (broad, s, 1 H, CH^A^ *H* ^B^,fwhm 0.4 ppm), 3.77 (s, EtOH, OH), 3.69 (q, 2 H, EtOH (CH_2_)), 1.82 (broad, s, 3 H, CH_3_, fwhm 0.3 ppm), 1.19 (q, EtOH (CH_3_)).^13^C NMR (125.81 MHz, dmso-d_6_, 300 K): *δ =* 176.0, 140.6, 123.5, 58.3 (EtOH), 19.5, 18.6 (EtOH). ATR FTIR (cm^–1^): 3700–3100 w, 3090 w, 2968 w, 2921 w, 1640 w, 1504 s, 1450 s, 1398 m, 1377 s, 1360 s,1227 s, 1001 s, 927 s, 845 m, 830 s, 656 w, 467 s

## Results and Discussion

### Synthesis and Characterization

The lanthanoid-doped BiO-NCs were synthesized through the simultaneous hydrolysis of Bi­(NO_3_)_3_·5H_2_O and Ln­(NO_3_)_3_·*n*H_2_O (*n* = 5, 6) in dmso using an aqueous NaOH solution in accordance with our study on cerium-doped BiO-NCs.^32,61^ The lanthanoid-doped BiO-NCs [Bi_38_O_45_(NO_3_)_24_(dmso)_28–*y*_]:Ln (**C-1:Ln**; Ln = La, Pr, Nd, Sm, Eu, Gd, Tb, Dy, Ho, Er, Tm, Yb, Lu; *y* = 0–2) were obtained from dmso after acetone vapor diffusion into the dmso solution as crystalline solids. Exemplarily, the ligand shell of **C-1:Gd** and **C-1:Dy** was substituted using sodium methacrylate and the resulting BiO-NCs were recrystallized in ethanol to give [Bi_38_O_45_(OMc)_24_(EtOH)_14_]:Ln (**C-2**_**E**_ **:Ln**; Ln = Gd, Dy), of higher solubility and were surrounded by solvent molecules that are much easier to remove.^62^ Thus, upon gentle heating in a vacuum, these BiO-NCs can be converted into solvate -free [Bi_38_O_45_(OMc)_24_]:Ln (**C-2**_**d**_ **:Ln**; Ln = Gd, Dy).

The amount of dopants in the as-prepared BiO-NCs was analyzed using ICP-OES and revealed values of about ≈1 ω% (Table 1). In combination with elemental analysis, the chemical compositions of **C-1:Ln** were determined leading to a general formula of [Bi_38–*x*_Ln_*x*_O_45_(NO_3_)_24_(dmso)_28–*y*_] (*x* = 1.0–0.4; *y* = 0–2) (Table 1). The compositions of **C-2**_**E**_ **:Ln** were determined as [Bi_37.4_Ln_0.6_(OMc)_24_(EtOH)_14_] for both Gd^3+^ and Dy^3+^ doped BiO-NCs. Similar to the previously published cerium- and europium-doped BiO-NCs, the herein-reported clusters show nonstoichiometric doped core compositions suggesting a statistical, yet nonuniform, distribution of dopants with position-dependent probabilities, leading to bulk material that is composed of undoped and differently doped BiO-NCs.

**Table 1:** Overview about the Lanthanoid Content and the Ln/Bi Ration in the Different Lanthanoid Doped BiO-NCs Determined at the Crystalline Bulk Samples Using ICP-OES^a^

**BiO-NC:** **Ln**	**ICP-OES** **Ln [ω%]**	**ICP-OES** **Ln/Bi**	**Composition**
**C-1:La**	0.63(1)–0.73(2)	0.015–0.019	[Bi_37.40_La_0.60_O_45_(NO_3_)_24_(dmso)_28_]
**C-1:Ce** ^32^	1.10(3)	0.022	[Bi_37_Ce_1_O_45_(NO_3_)_24_(dmso)_28_]
**C-1:Pr**	0.82(2)–0.86(2)	0.021	[Bi_37.27_Pr_0.73_O_45_(NO_3_)_24_(dmso)_28_]
**C-1:Nd**	0.89(4)–0.90(4)	0.022–0.024	[Bi_37.23_Nd_0.77_O_45_(NO_3_)_24_(dmso)_28_]
**C-1:Sm**	0.71(3)–0.84(2)	0.017–0.020	[Bi_37.36_Sm_0.64_O_45_(NO_3_)_24_(dmso)_28_]
**C-1:Eu** ^61^	0.87(3)–1.17(3)	0.018–0.029	[Bi_37.40_Eu_0.60_O_45_(NO_3_)_24_(dmso)_28_]
**C-1:Gd**	0.84(2)–1.08(2)	0.017–0.019	[Bi_37.25_Gd_0.75_O_45_(NO_3_)_24_(dmso)_28_]
**C-1:Tb**	0.87(3)–0.89(4)	0.019	[Bi_37.33_Tb_0.67_O_45_(NO_3_)_24_(dmso)_26_]
**C-1:Dy**	0.84(3)–0.97(2)	0.018–0.019	[Bi_37.32_Dy_0.68_O_45_(NO_3_)_24_(dmso)_27_]
**C-1:Ho**	0.85(1)–0.87(2)	0.017–0.018	[Bi_37.36_Ho_0.64_O_45_(NO_3_)_24_(dmso)_27_]
**C-1:Er**	0.76(3)–0.87(2)	0.017–0.021	[Bi_37.41_Er_0.59_O_45_(NO_3_)_24_(dmso)_28_]
**C-1:Tm**	0.58(2)–0.61(2)	0.012–0.013	[Bi_37.57_Tm_0.43_O_45_(NO_3_)_24_(dmso)_26_]
**C-1:Yb**	0.72(2)–0.88(2)	0.015–0.016	[Bi_37.43_Yb_0.57_O_45_(NO_3_)_24_(dmso)_28_]
**C-1:Lu**	0.52(1)–0.59(1)	0.010–0.012	[Bi_37.61_Lu_0.39_O_45_(NO_3_)_24_(dmso)_28_]
**C-2**_**d**_ **:Gd**	0.84(2)–0.89(2)	0.018–0.019	[Bi_37.39_Gd_0.61_(OMc)_24_]
**C-2**_**d**_ **:Dy**	0.92(3)–0.94(1)	0.018–0.019	[Bi_37.39_Dy_0.61_(OMc)_24_]

a Please note that for the bulk analysis of BiO-NC **C-2**_**E**_ **:Gd** and **C-2**_**E**_ **:Dy**, the completely dried samples, BiO-NC **C-2**_**d**_ **:Gd** and **C-2**_**d**_ **:Dy** were used, in order to have a defined composition without the volatile packing solvent ethanol.

In order to prove the chemical compositions of **C-1:Ln**, electrospray ionization mass spectrometry (ESI-MS) studies were carried out. Applying soft ionization conditions, the BiO-NCs maintain their molecular structures and preserve the majority of the coordinated ligands.^30^ By stripping off the appropriate number of counterions and dmso solvates in the ion source, doubly, triply, and quadruply charged BiO-NC cations of the clusters **C-1:Ln** are detected, in multiple reaction monitoring (MRM) experiments (cf. Supporting Information). The quadruply positively charged region of the spectrum has proven to give valuable insights for doped BiO-NCs, wherefore this is analyzed in more detail.^31,32^ The results for the gadolinium-doped cluster **C-1:Gd** are discussed (Figure 1), all other doped BiO-NCs show similar characteristics (Figures S1–S42). In addition to the quadruply positively charged species of **C-1:Gd**, the undoped cationic BiO-NC species are observed with high intensity. The homometallic cation [Bi_38_O_46_(NO_3_)_18_(dmso)_7_]^4+^ (**V**, *m*/*z* = 2584.7334), which results from [Bi_38_O_45_(NO_3_)_20_(dmso)_*y*_]^4+^ by stepwise loss of dmso and N_2_O_5_,^30^ shows the signal of highest intensity followed by the signal of the homometallic [Bi_38_O_46_(NO_3_)_18_(dmso)_6_]^4+^ (**VI**, *m*/*z* = 2565.2208) among others. Doped BiO-NC species with up to three bismuth atoms substituted by gadolinium such as [Bi_38–*x*_Gd_*x*_O_46_(NO_3_)_18_(dmso)_*y*_]^4+^ (**XI**, *x* = 1, *y* = 6, *m*/*z* = 2552.4575; **XII**, *x* = 2, *y* = 7, *m*/*z* = 2559.2104; **XIII**, *x* = 3, *y* = 6, *m*/*z* = 2526.4283) are observed. Additionally, assigned cationic BiO-NCs of **C-1:Gd** are summarized in Table S3, and the calculated and experimental patterns are shown in Figure S3.

**Figure 1:**
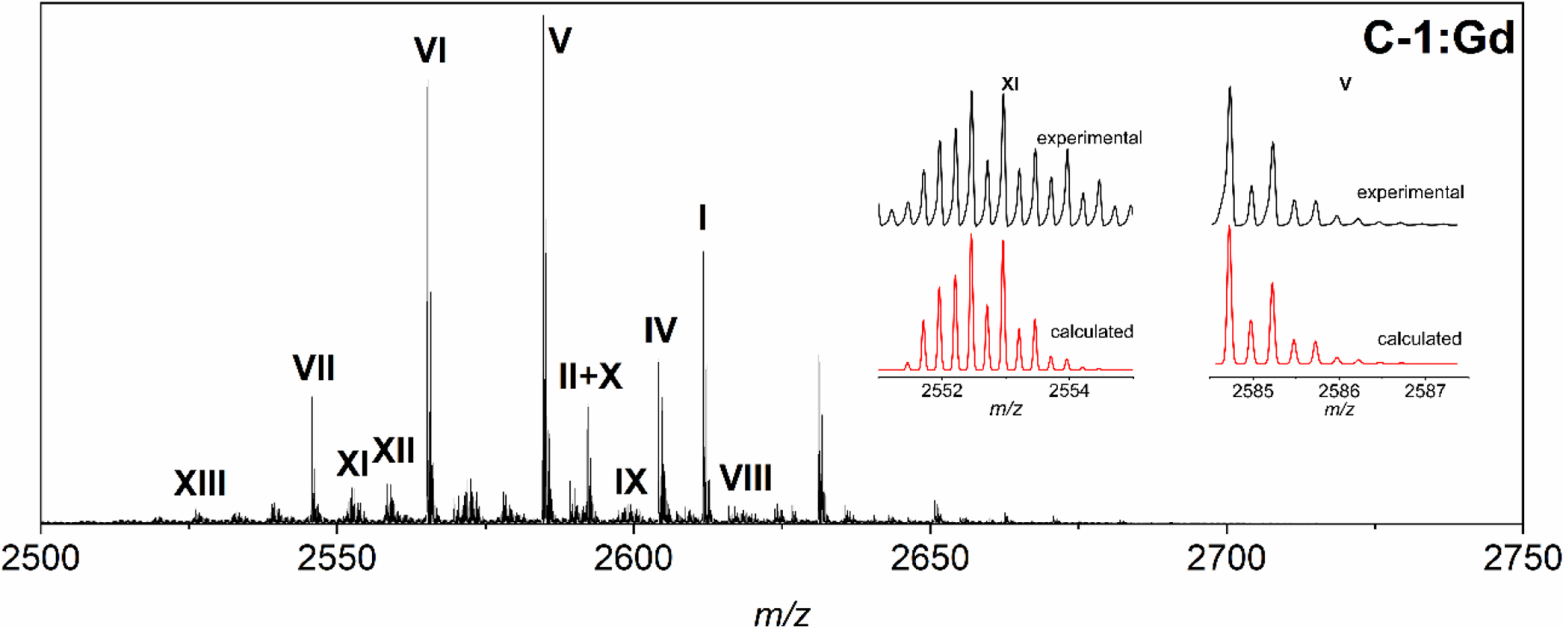
Part of the ESI mass spectrum of compound **C-1:Gd** electrosprayed from MeCN/dmso showing quadruply positively charged homometallic cations (**I**–**VII**) with [Bi_38_O_46_(NO_3_)_18_(dmso)_7_]^4+^ (**V**, *m*/*z* = 2611.7214) and heterobimetallic BiO-NC cations (**VIII**–**XIII**) with [Bi_37_GdO_46_(NO_3_)_18_(dmso)_6_]^4+^ (**XI**, *m*/*z* = 2552.4575) showing the highest abundance.

For all other lanthanoid-doped nitrate-decorated BiO-NCs **C-1:Ln** similarly, the homometallic species, either [Bi_38_O_45_(NO_3_)_20_(dmso)_7_]^4+^ (**I**, *m*/*z* = 2611.7195) or [Bi_38_O_46_(NO_3_)_18_(dmso)_6_]^4+^ (**VI**, *m*/*z* = 2565.2208), dominate the mass spectra as the most intense *m*/*z* signals. Further cations with varying numbers of dopants, dmso solvates and nitrate anions were assigned, whereby species with cluster cores up to {Bi_33_Ln_5_O_45_} (**C-1:La**, **C-1:Pr**), {Bi_34_Ln_4_O_45_} (**C-1:Nd**, **C-1:Tb**, **C-1:Ho**, **C-1:Er**, **C-1:Lu**, **C-1:Tm**) and {Bi_35_Ln_3_O_45_} (**C-1:Sm**, **C-1:Yb**, **C-1:Gd, C-1:Dy**) are observed (cf. Figures S1–S42, Tables S2–S15). The abundance of the respective species decreases from undoped to finally multiple-doped cluster fragments.

The ESI-MS spectra of the methacrylate-substituted BiO-NCs **C-2**_**d**_ **:Gd** (Figure 2) and **C-2**_**d**_ **:Dy** show fewer and less overlapping *m*/*z* signals due to the absence of solvates in the gas phase and a significantly higher stability. For these clusters, the most intense and best-separated fragments are observed in the +2 charge state. The dominant species for both BiO-NCs is the undoped [Bi_38_O_45_(OMc)_22_]^2+^ (**XXXII**, *m*/*z* = 5265.8311). In addition, a series of doped fragments with the composition [Bi_38–*x*_Ln_*x*_O_45_(OMc)_22_]^2+^ (*x* = 1–4) is identified (cf. Figures S15, S18). In agreement with our previous results, we estimate that the doped BiO-NCs consist of homometallic BiO-NCs (≈50%) and of heterobimetallic BiO-NCs. This is in contrast to most heterobimetallic clusters reported so far, which show substitution at a defined position in the core structure. The fact that the signal intensity is highest for undoped species and decreases for the respective multimetallic species is consistent with the dopant amount (ω ≈ 1%).

**Figure 2:**
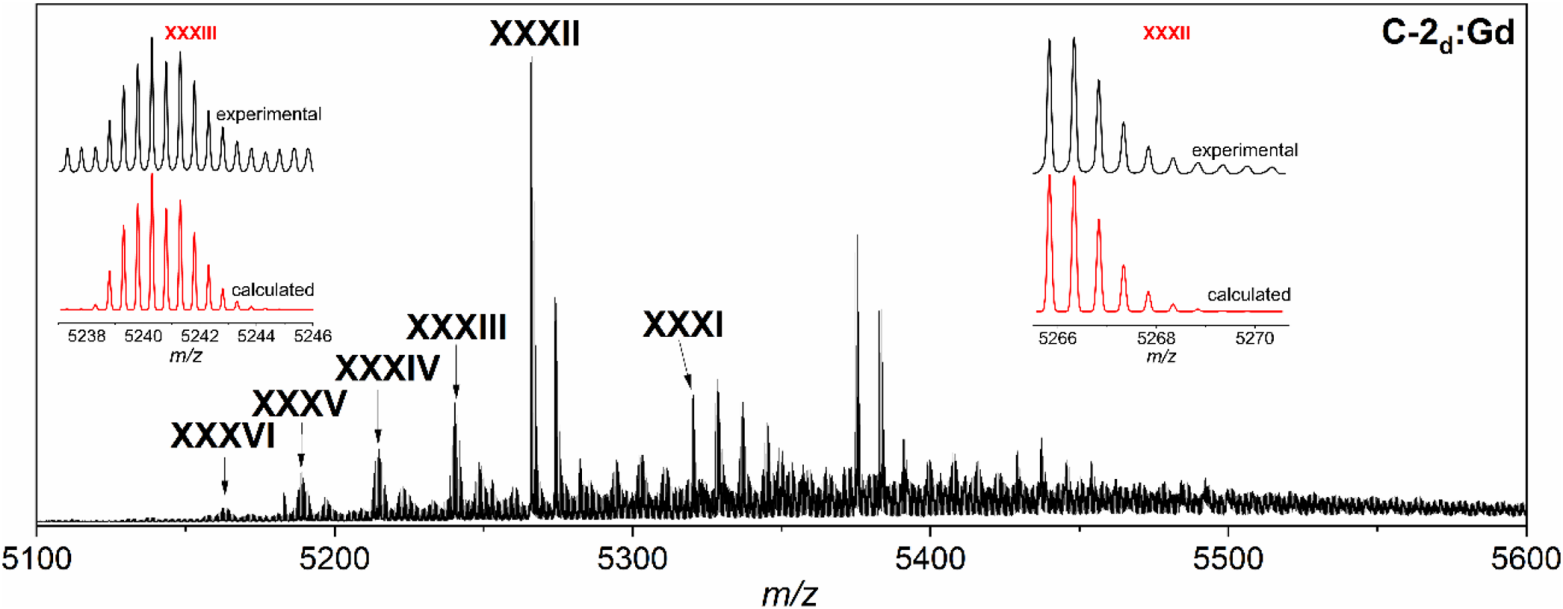
Part of the ESI mass spectrum of compound **C-2_d_:Gd** electrosprayed from *^i^* PrOH showing doubly charged homometallic cations (**XXXI**, **XXXII**) with [Bi_38_O_45_(OMc)_22_]^2+^ (**XXXII**, *m*/*z* = 5265.8311) and heterobimetallic BiO-NC cations (**XXXIII**–**XXXVI**) with [Bi_37_GdO_45_(OMc)_22_]^2+^ (**XXXIII**, *m*/*z* = 5240.3042) showing the highest abundance.

The ATR-IR spectra of the nitrate-decorated BiO-NCs **C-1:Ln** are in good agreement with that of the undoped cluster **C-1** (Figure S43).^28,31^ They show vibrations for NO_3_^–^ at 995 cm^–1^ (ν_sym._(NO_2_)) and 1430 cm^–1^ (ν_*a*s._(NO_2_)) for monodentate NO_3_^–^, at 1265 cm^–1^ (ν_sym._(NO_2_)) and 1385 cm^–1^ (ν_*a*s._(NO_2_)) for bidentate NO_3_^–^ and at about 1740 cm^–1^ (ν­(N=O)_free_) for free nitrate, as well as coordinated dmso with a characteristic symmetric S=O valence vibration at 948 cm^–1^.^63^ For the solvate-free methacrylate-substituted clusters **C-2**_**d**_ **:Gd** and **C-2**_**d**_ **:Dy**, ATR-IR spectra confirm the absence of nitrate and dmso whereas C=C, C–H, and C—O vibration bands are assigned to methacrylate; thus, we conclude with a complete ligand exchange (Figure S44).^62^ All clusters exhibit a broad band at approximately 480 cm^–1^ attributed to Bi–O vibrations of the BiO-NC core. A trend regarding the dopant influence on the Bi–O vibration band in **C-1:Ln** does not become obvious in the IR spectra.

The powder X-ray diffraction pattern (PXRD) of the lanthanoid-doped BiO-NCs **C-1:Ln**and methacrylate-functionalized BiO-NCs **C-2_d_:Ln** (Figures S45–S46) show the typical pattern for the BiO-NCs with a {Bi_38_O_45_} cluster core. The patterns show a major diffraction peak appearing at 2*θ ≈* 5°, which corresponds to the interlayer distance of the nearly close-packed nanoclusters in the solid state.^32^ The corresponding interlayer distances and the thus-calculated diameters using Bragg’s law, of the lanthanoid-doped BiO-NCs **C-1:Ln**, **C-2_d_:Gd**, and **C-2_d_:Dy**are primarily influenced by the respective size of the cluster shell due to the nearly constant [Bi_38–*x*_Ln_*x*_O_45_]^24+^ core size (Table S16). The determined values for nitrate-functionalized BiO-NCs (*d* ≈ 1.7 nm) and methacrylate-functionalized BiO-NCs (*d* ≈ 1.6 nm) are in line with previous results.^32^

Single crystal X-ray diffraction analysis (SC XRD) was performed exemplarily for the BiO-NCs **C-1:Gd, C-1:Dy**, and **C-2**_**E**_ **:Gd** (cf. Figures S50–S53, Tables S17–S25). The corresponding data are summarized in the Supporting Information and the resulting structures of the Gd^3+^ doped BiO-NCs (Figure 3) are visualized. All crystal structures show a good agreement with their isostructural homometallic host structures **C-1′** and **C-2**_**E**_ (discussed in SI). The doped BiO-NCs exhibit slightly different electron densities at some Bi^3+^ positions, indicating partial substitution by the dopant element in those structures. The dopant occupancy at all bismuth positions was evaluated in accordance to our previously published strategy and is described in detail in the SI.^31,32^ A nonuniform distribution with preferred lanthanoid occupancy at the inner cluster core positions was revealed, in accordance to the ESI-MS studies and the results on cerium-doped BiO-NCs.^31,32^ Thus, the BiO-NC core compositions from SC XRD were determined as [Bi_37.31_Gd_0.69_O_45_]^24+^ (**C-1:Gd**), [Bi_36.97_Dy_1.03_O_45_]^24+^ (**C-1:Dy**), and [Bi_33.24_Gd_4.76_O_45_]^24+^ (**C-2**_**E**_ **:Gd)**, which are slightly lower for **C-1:Ln** and higher for **C-2**_**E**_ **:Gd** than the values determined for the clusters in the bulk material using ICP-OES (cf. Table 1). When considering the 3σ significance criteria in the SC XRD data treatment compared to the undoped structure (Tables S19, S22, S24),^64^ the cluster core values show better agreement with those from the bulk materials.

**Figure 3:**
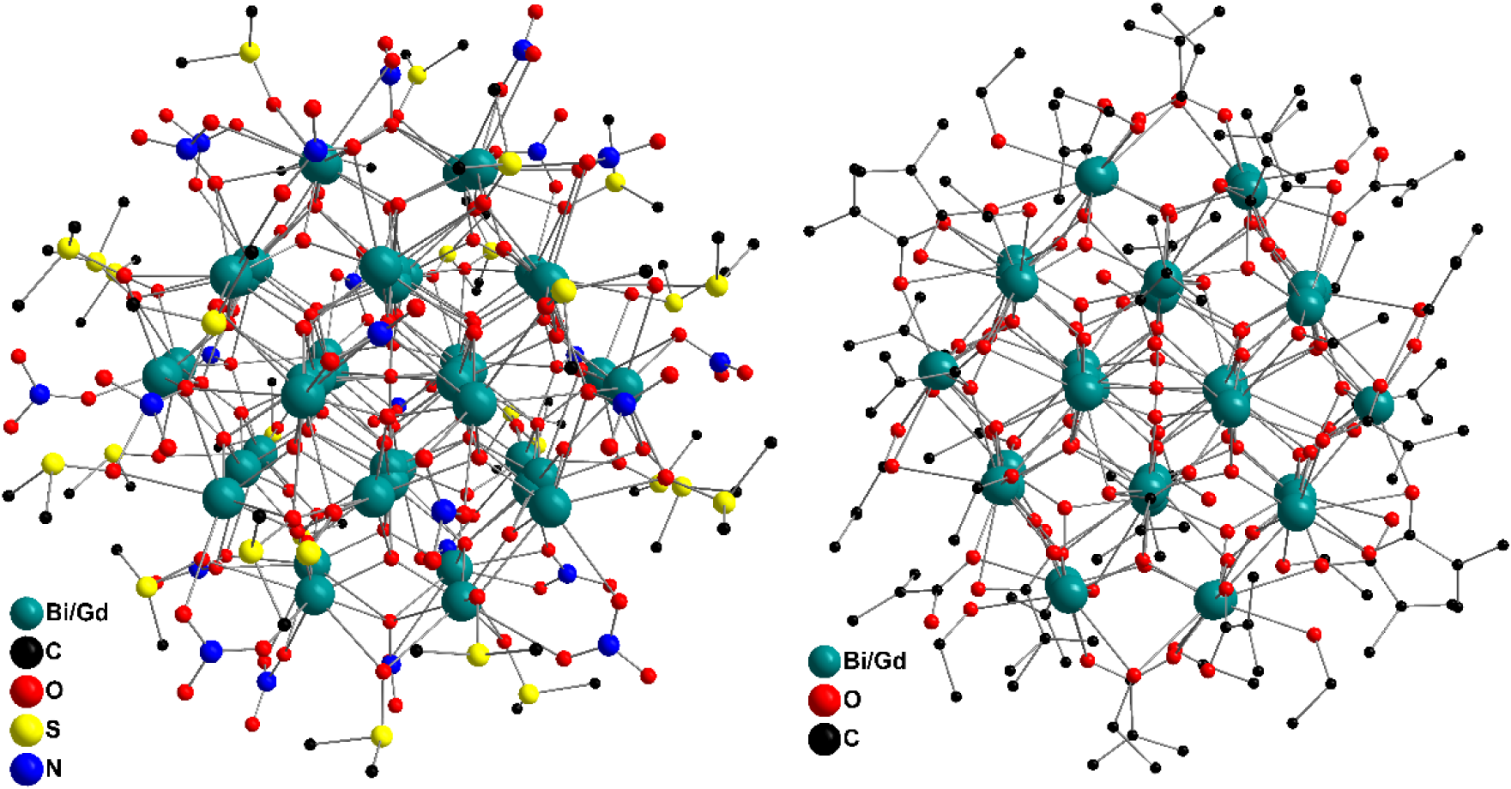
“Ball-and-Stick” model of the Gd^3+^-doped BiO-NCs **C-1:Gd** (*left*) and **C-2_E_:Gd** (*right*). Hydrogen atoms as well as noncoordinated molecules are omitted for clarity. The calculated occupancy factors of Bi^3+^ positions by Gd^3+^ are summarized in Tables S19 and S24.

In accordance with previous studies, the dopants Gd^3+^ and Dy^3+^ only have a minor effect on the M–O bond distances and the connectivity in the cluster core.^31,32,61^ However, the coordination environment of the ligands and the solvates slightly differs in the diverse structures reported yet as a result of the flexible coordination environment of the ligands and partial disorder (Tables S18, S21, S25). The simulated PXRD patterns from the SC XRD data for **C-1:Gd** and **C-1:Dy** agree with the respective measured pattern of the bulk samples (Figure S52).

In summary, in the synthetic and characterization part, doping with all lanthanoids (except Pm) was successful under the formation of the {Bi_38_O_45_}:Ln core. Only marginal differences in the dopant concentration were revealed in several BiO-NCs. By this means, trends or influences for the doping within the lanthanoid row, e.g., as a result of differences in size, were not identified. In the second part, we describe the optical and magnetic properties, focusing on selected lanthanoid-doped BiO-NCs.

### UV–vis and PL Spectroscopic Studies

Most lanthanoid-based compounds exhibit absorption in the UV–vis or NIR region, enabling the tuning of the optical properties of the host materials through doping. The UV–vis absorption spectra in diffuse reflectance (DRS) for all lanthanoid-doped BiO-NCs show typical transitions for Pr^3+^, Nd^3+^, Sm^3+^, Dy^3+^, Ho^3+^, Er^3+^, Tm^3+^ and Yb^3+^ in the measured range from 250 nm–1000 nm (Figure 4), whereas La^3+^, Gd^3+^, Tb^3+^ and Lu^3+^ do not show any lanthanoid-specific absorption bands (Figure S54). Cutouts as well as an assignment of the detected bands to the respective transitions for the doped BiO-NCs are summarized in the SI (Figures S55–S62; Tables S26–S33). In addition, the BiO-NCs show broad absorption bands in the range between 400 and 250 nm, which are attributed to the absorption of the bismuth oxido core. In the spectra of **C-2**_**d**_ **:Gd** and **C-2**_**d**_ **:Dy**, this absorption is slightly red-shifted compared to the nitrate-substituted BiO-NCs, likely as a result of the LMCT between the cluster core and the carboxylate ligands.

**Figure 4:**
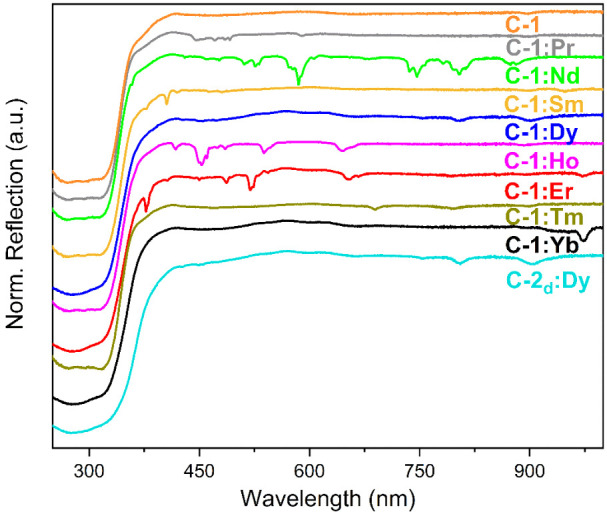
UV–vis diffuse reflectance spectra (DRS) of undoped BiO-NC **C-1**, lanthanoid-doped BiO-NCs **C-1:Ln** (Ln = Pr, Nd, Sm, Dy, Ho, Er, Tm, Yb), and **C-2_d_:Dy** showing lanthanoid-specific absorptions.

Lanthanoid-doped BiO-NCs show PL emissions from the visible to the NIR range as a result of lanthanoid-specific f–f transitions (Figure S63). In general, the bismuth cations in the BiO-NCs show asymmetric coordination environments with varying Bi–O bond distances as well as further long-range interactions in the periphery, likewise this is expected for the lanthanoid ions replacing the bismuth in the doped metal oxido core. Owing to reports on strong luminescence with sharp emission bands in different ranges of the electromagnetic spectrum, we selected the nitrate-functionalized doped BiO-NCs, with Er^3+^ (**C-1:Er**) and Yb^3+^ (**C-1:Yb**) and exemplarily the methacrylate-substituted doped BiO–NC **C-2**_**d**_ **:Dy** for a detailed temperature-, emission-, and excitation wavelength-dependent PL analysis.^65^

All undoped and doped BiO-NCs exhibit a broad Bi^3+^-based excitation signal (350 nm–275 nm) arising from the^3^P_1_ ← ^1^S_0_ transition and LMCT from O^2–^ to Bi^3+^ in the cluster core and an emission signal (425 nm–700 nm) resulting from the ^3^P_1_ → ^1^S_0_ transition of Bi^3+^ at low temperature, which we have previously reported on.^61^

To provide additional information on the phosphorescent nature of the Bi^3+^-based transitions in **C-1**, the Gd^3+^ doped BiO-NC **C-1:Gd** was studied at 2.4 K and a broad emission in the visible region was obtained (Figure S64). The CIE coordinates of *X* = 0.385 and *Y* = 0.499 are comparable with the ones obtained for the undoped **C-1** (Figure S65, Table S34), as are the lifetimes of τ_1_ = 29 μs and τ_2_ = 102 μs (Figure S66). Such observations indicate the Bi^3+^-based nature of emission in the cluster, as visible emission from Gd^3+^ is not expected due to the high-lying energy levels of the ion relative to the ^3^P_1_ level of Bi^3+^.^66,67^

### Photophysical Studies of **C-1:Er**

The PL emission spectra of Er^3+^ doped BiO-NC **C-1:Er** showed the strong Er^3+^-based ^4^I_13/2_ → ^4^I_15/2_ NIR transition (λ_max_ = 1534 nm), in addition to the broad Bi^3+^-based emission in the visible region (425 nm–700 nm), with CIE coordinates indicating a yellow-orange emission color (Figure S67) under excitation at 310 nm (Figure 5). The presence of the Er^3+^-based ^4^I_13/2_ → ^4^I_15/2_ transition upon excitation at 310 nm indicates the Bi^3+^ sensitized nature of the emission.^68^ The PLE spectrum of **C-1:Er** is dominated by the broad Bi^3+^-based transition at 310 nm, when emission is monitored at 600 nm. On the other hand, when the emission is monitored at 1534 nm, the PLE spectrum is composed of several strong Er^3+^-based transitions in addition to the Bi^3+^-based transition (Figure 5, Table S35). This dual excitation offers to study the excitation wavelength dependence of NIR emission of **C-1:Er** (cf. Figure S68). Excitation of Er^3+^-based transitions, such as at 381 nm (^4^G_11/2_ ← ^4^I_15/2_) and at 523 nm (^2^H_11/2_ ← ^4^I_15/2_) result likewise in the observation of ^4^I_13/2_ → ^4^I_15/2_ emission in the NIR region (λ_max_ = 1534 nm). The Er^3+^-based NIR emission is slightly affected by the different excitation wavelengths, likely as a result of the different excitation paths from direct Er^3+^ f–f transitions or energy transfer from Bi^3+^-based excitations.

**Figure 5:**
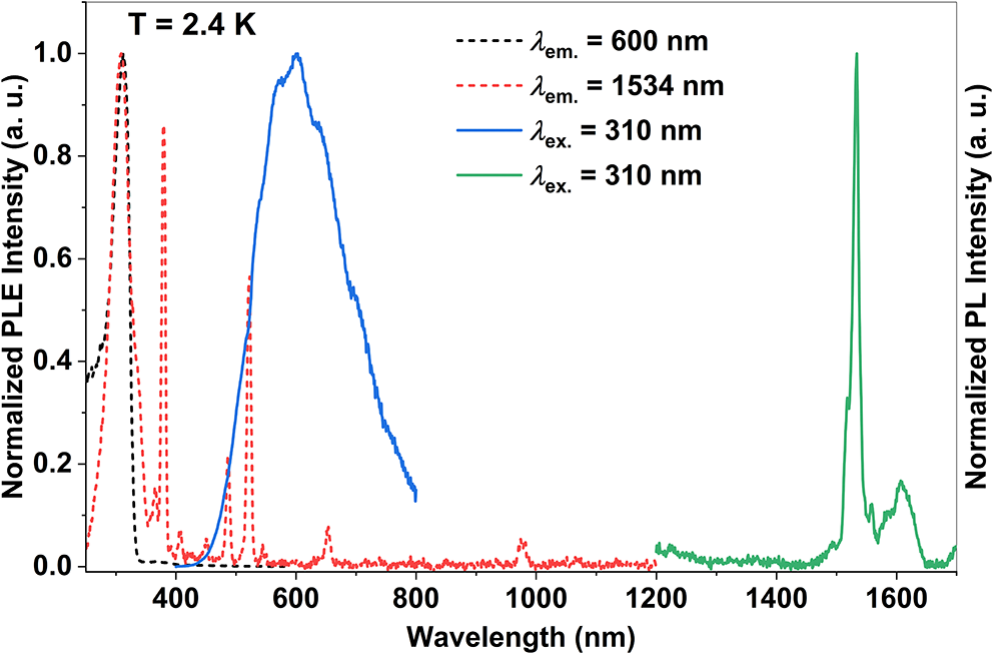
Normalized PLE and PL spectra of BiO-NC **C-1:Er** at 2.4 K. On excitation at 310 nm, Bi^3+^-based phosphorescence in the visible and Er^3+^-based f–f ^4^I_13/2_ → ^4^I_15/2_ emission in the NIR ranges are observed.

In addition, the temperature dependence of the PLE and PL intensities in both the visible light region and the NIR region (Figure S69) for **C-1:Er** is observed, upon excitation at 310 nm. Similar to the previously reported behavior for the Eu^3+^-doped BiO-NCs, the intensities tend to decrease with temperature increase. In contrast to the previous studies, in the Er^3+^-doped BiO-NCs, the Bi^3+^-based emission is still observed at temperatures above 180 K.^61^ This is likely a result of the closely lying energy levels of Bi^3+^ and Er^3+^.

We further investigated Er^3+^-centered visible PL emission at 300 K as a function of the excitation wavelength in the doped BiO-NCs (Figure 6). The choice of 300 K is justified considering the weak intensity of the Bi^3+^-based emission, allowing the observation of faint Er^3+^-based transitions in the visible region. To obtain noise-free emission profiles, wider emission slit widths were used than the ones used for the temperature-dependent studies (Figure S69a). Excitation of the cluster at 310 and 317 nm resulted in the observation of dual emission composed of sharp Er^3+^-based transitions (Table S35) and the weak and broad Bi^3+^-based ^3^P_1_ → ^1^S_0_ emission (Figure 6). Remarkably, excitation at 470 nm yielded almost exclusively a sharp Er^3+^-based (^4^F_9/2_ → ^4^I_15/2_) emission at 650 nm. Excitation spectra obtained by monitoring emission at 600 nm yielded only the broadband (λ_max_ = 310 nm) related to Bi^3+^-based excitation, while at 650 nm both the broad Bi^3+^-based transition, and the Er^3+^-based ^4^F_7/2_ ← ^4^I_15/2_ transition at 470 nm are observed. Therefore, we conclude that the visible ^4^F_9/2_ → ^4^I_15/2_ emission at 650 nm can be sensitized either through excitation of the Bi^3+^-based transition at 310 nm or via direct excitation of the Er^3+^-based f–f transition at 470 nm.

**Figure 6:**
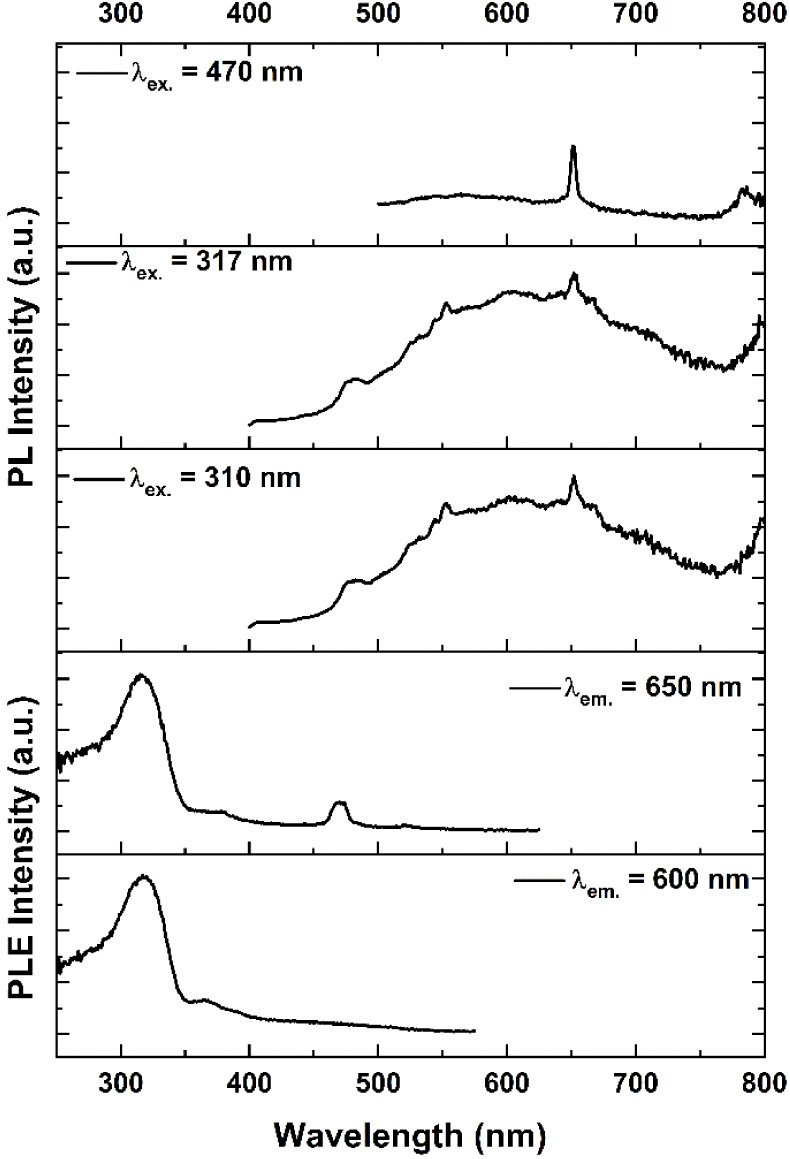
Emission wavelength-dependent PLE and excitation wavelength-dependent PL spectra of BiO-NC **C-1:Er** at 300 K.

Excitation of the cluster at 310 nm leads to the broad radiative Bi^3+^ emission, additionally, a fraction of the absorbed energy is transferred nonradiatively to Er^3+^, populating higher-lying 4f manifolds that lead to the Er^3+^-based visible emission. The strong overlap between the Bi^3+^-based emission and Er^3+^-based excitations (Figure 5) indicates energy transfer from the Bi^3+^-based ^3^P_1_ level to the excited manifolds of Er^3+^. This pathway also leads to Er^3+^ emission in the visible spectrum. Direct excitation at the Er^3+^ absorption at 470 nm (^4^F_7/2_ ← ^4^I_15/2_) causes the ^4^F_7/2_ state to nonradiatively relax and populate the ^4^F_9/2_ state. Subsequent radiative relaxation of this state results in the observation of the red ^4^F_9/2_ → ^4^I_15/2_ transition, which is centered at 655 nm. The nearly exclusive emission at 655 nm upon excitation at 470 nm is attributed to the selective population of the Er^3+^ states.

Lifetime studies at cryogenic temperatures are performed to get insights into the dynamics of the excited states. On exciting the sample at 319 nm and monitoring the Bi^3+^-based ^3^P_1_ → ^1^S_0_ emission at 600 nm at 2.4 and 77 K, lifetimes of about 8 μs (2.4 K) and 7 μs (77 K) are estimated from the monoexponential fitting of the decay profile (Figure S70, Table S37). These values are smaller than the ones obtained for the undoped and the Gd^3+^ doped clusters.^61^ Such reduction of the lifetime value in the Er^3+^ doped BiO-NCs is attributed to a complex energy transfer pathway involving the donating Bi^3+^-centered ^3^P_1_ level and the accepting 4f states of Er^3+^. The strong overlap between the Bi^3+^-centered emission and Er^3+^-based excitation peaks shown in Figure 5 is in line with the above hypothesis.^69,70^ Also, such pathways involving closely lying 4f levels of Er^3+^ and Bi^3+^-based levels are the likely factors behind the observation of the Bi^3+^-based ^3^P_1_ → ^1^S_0_ emission at higher temperatures. Lifetime profiles of the cluster at 300 K, obtained by monitoring emissions at 600 and 652 nm, can be fitted with a monoexponential function (Figure S71, Table S38). The fittings yielded comparable lifetimes of 4 μs (λ_em._ = 600 nm) and 3 ns (λ_em._ = 652 nm), indicating a comparable radiative decay rate of the Bi^3+^-based emission at 300 K and cryogenic temperatures. The short lifetime estimated for the Er^3+^-based component at 652 nm indicates the fast-radiative decay of the ^4^F_9/2_ state, explaining its weak intensity at 300 K. Since the emission signals arising from the Bi^3+^ and Er^3+^ centers are quite weak, the data were collected for a few hours at each wavelength to receive a relatively noise-free data set, allowing us to estimate the lifetimes by fitting the data with a monoexponential function.

Overall, the Er^3+^-doped cluster exhibits intriguing photophysical properties such as a combination of dual Bi^3+^- and Er^3+^-based emission in the visible range and Er^3+^-based emission in the NIR range, which has been reported for the first time to the best of our knowledge.

### Photophysical Studies of **C-1:Yb**

Temperature-dependent PL and PLE measurements of **C-1:Yb** were performed in the visible and NIR regions. The PL and PLE spectra in the visible region (Figure S72), as well as the PL lifetimes, are in good agreement with those of the undoped and Gd^3+^ doped BiO-NCs. For **C-1:Yb** the CIE coordinates reveal a yellow emission color (Figure S73). PL lifetimes (Figure S74, Table S39) of τ_1_ = 57 μs and τ_2_ = 124 μs are observed at 300 K under excitation with 310 nm.

The PL spectra of **C-1:Yb** at 2.4 K showed the ^2^F_5/2_ → ^2^F_7/2_ transition in the typical range from 970 to 1100 nm. A strong excitation wavelength dependence of the Yb^3+^ emission maxima is observed (cf. Figure 7). Similarly, the PLE profile has a strong emission wavelength dependence. The Yb^3+^-specific emission is split into different Stark sublevels under the influence of the crystal field, in both the ground ^2^F_7/2_ state (four Stark-split levels) and the excited ^2^F_5/2_ state (three Stark-split levels). Considering population polarization at the lowest energy level of the excited ^2^F_5/2_ manifold, four transitions are expected in the 970 nm–1100 nm range with a zero-phonon line (ZPL) centered around 975 nm. Excitation of the cluster at 310 and 324 nm at 2.4 K resulted in the observation of several Yb^3+^ emission peaks in the expected range. Remarkably, excitation at 310 nm resulted in higher emission intensities at 977 and 1002 nm than the ones at 973 and 1019 nm (cf. Figure S75). On excitation at λ_ex._ = 324 nm, changes in the intensity ratios are observed, relative to the intensities obtained on excitation at λ_ex._ = 310 nm. The intensities at 973 and 1019 nm are increased, whereas the intensities at 977 and 1002 nm are decreased. Excitation at 375 nm leads to Yb^3+^ emission with only one maximum centered at 977 nm. The maxima in the excitation spectra are centered at about 310 nm when monitored at λ_em._ = 977 and 1019 nm, but centered around 324 nm when monitored at λ_em._ = 973 and 1002 nm, in accordance with the observed emission properties. The excitation wavelength-dependent variations in the Yb^3+^ emission intensity ratios observed for **C-1:Yb** are tentatively attributed to the host-mediated sensitization mechanism. In **C-1:Yb**, excitation at 310 nm is assigned to a Bi–O charge-transfer (CT) transition, while excitations at 324 and 375 nm might be assigned to Bi^3+^ based ^1^S_0_ → ^3^P_1_ and ^1^S_0_ → ^3^P_0_ transitions, respectively. These Bi–O CT and direct Bi^3+^ based transitions should differ in their relaxation pathways and coupling to Yb^3+^ ions, resulting in excitation pathway-dependent population of the crystal field-split ^2^F_5/2_ excited state of Yb^3+^. At cryogenic temperature (2.4 K), thermal redistribution among the sublevels of the Yb^3+^ excited state is suppressed, so the emission spectra directly reflect these non-Boltzmann population distributions. Consequently, different excitation wavelengths lead to different relative intensities of the Yb^3+^ emission lines. We attribute the strong wavelength dependence of the emission and excitation profiles in **C-1:Yb** to at least three different Yb^3+^ sites (1–3) present in the cluster.^71,72^ The varying sensitization of different Yb^3+^ sites on Bi^3+^-based excitations at 310 and 324 nm indicates local coordination environment variations around Yb^3+^, which selectively couples with the excitation leading to site-selective excitation and emission.

**Figure 7:**
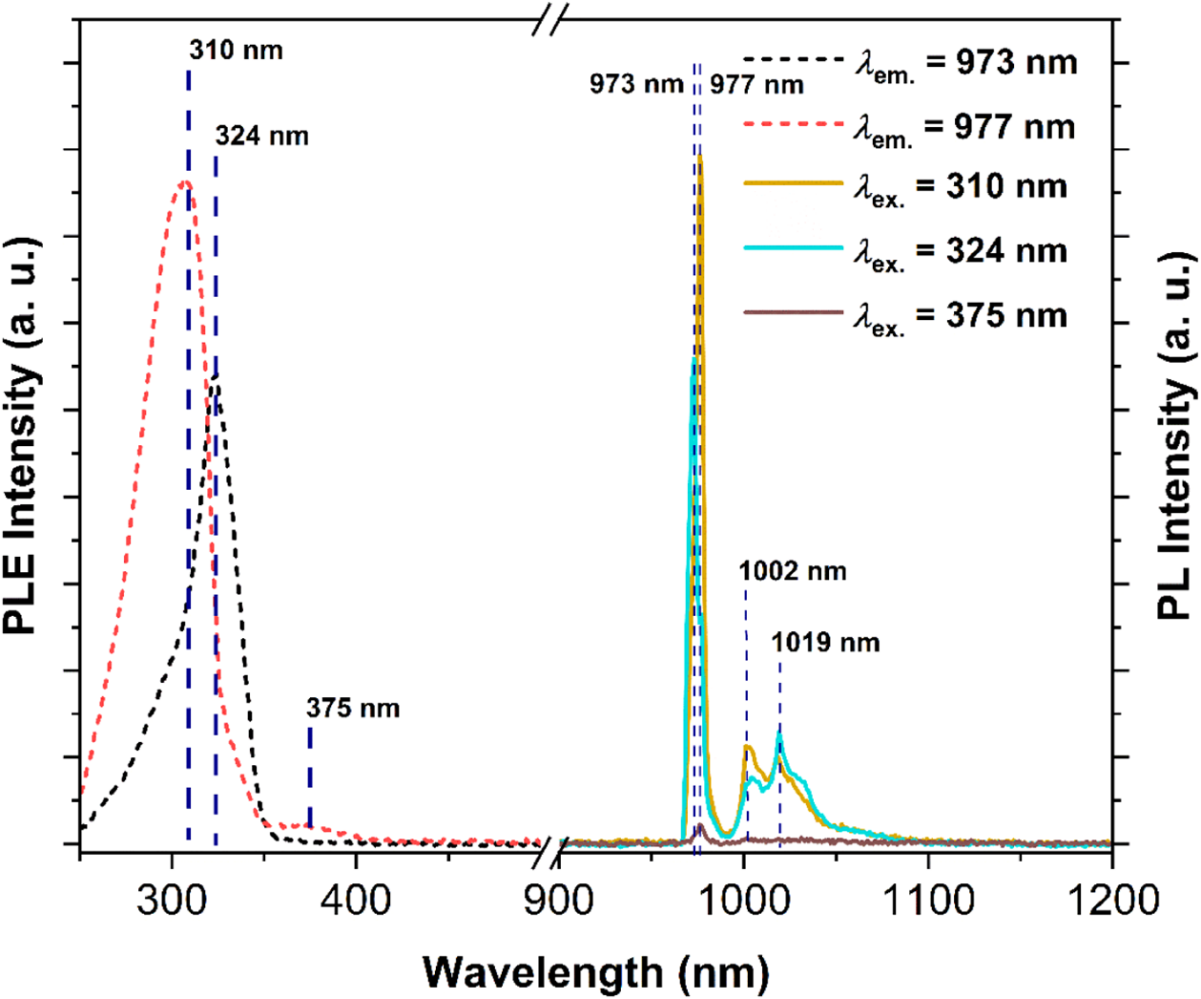
PLE and PL spectra of BiO-NC **C-1:Yb** at 2.4 K.

From the temperature-dependent PL and PLE studies of **C-1:Yb** in the NIR range (Figures S76, S77), we note that the excitation maxima remained close to 310 nm with temperature rise. On the other hand, we noted a strong intensity reduction of this excitation peak at 180 K; at 300 K it is not observed. This is likely a result of the sensitive nature of the Bi^3+^-based excitation at elevated temperatures,^61^ or the mixed population of different states as well as higher phonon coupling at higher temperatures. A notable feature of the emission of **C-1:Yb** is the clear structure observed in the PL spectrum obtained by exciting the sample at 324 nm at 2.4 K, relative to the comparable but not well-resolved transitions obtained on the 310 nm excitation. This Yb^3+^ emission profile (λ_ex._ = 324 nm) was studied temperature-dependent in the range of 2.4 K–100 K (Figures 8, S78). The most notable changes are the different decreasing PL signal intensities and signal broadening with increased temperature. Further, a maximum shift from 973 to 977 nm and from 1019 to 1002 nm is observed. In the range of 965 nm–980 nm the intensity and the splitting are significantly changed with respect to temperature. At 2.4 K the peaks show a maximum at 973 nm with two shoulders at around 970 and 977 nm, which have lower intensities. As the temperature increases in steps of 10 K, the intensity of the peak centered at 973 nm decreases more than its shoulder peaks. At around 50 K all three peaks have nearly the same intensity and a clear splitting is observed. With further temperature increase until 100 K, the intensity of the peak at 977 nm remains nearly constant, while the intensities of the peaks at 973 and 970 nm decrease slightly (Figure S79). We attribute this variation in peak intensities to the population transfer from the Yb^3+^ sites (2 and 3) that are lying slightly above (973 and 970 nm) site 1 at 977 nm (Figure 8). The temperature-dependent bathochromic (red) shifting at 180 and 300 K causing the prominent emission at 977 nm is evidence of complete population transfer from the higher energy sites. In order to quantify the site-dependent population, we have employed Lorentz deconvolution of the emission bands at 2.4 K and 100 K (Table S40). The data can be satisfactorily fitted with three Lorentz functions at each temperature. The decrease in the populations of the higher energy sites 2 and 3 relative to site 1 at 100 K indicates the temperature-mediated population transfer from sites 2 and 3 to site 1.

**Figure 8:**
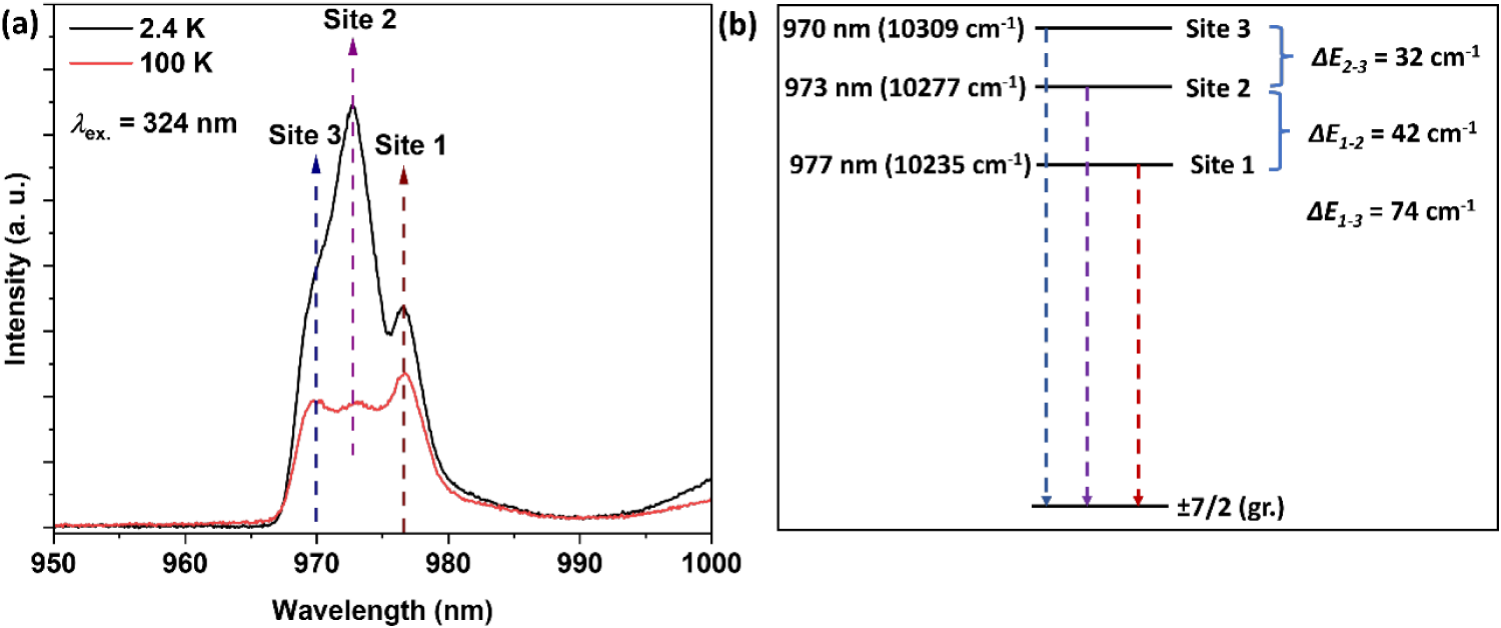
(a) PL emission of **C-1:Yb** in the 950 to 1000 nm range on excitation at 324 nm (*T* = 2.4 and 100 K). (b) Energy levels involved in the transitions observed at 970 nm, 973 nm, and 977 nm.

The Yb^3+^-based emissions are contaminated by the presence of vibronic fine structure and hot bands. The former one is observed in the low-energy regions of the ZPL; therefore, its involvement in the observation of three distinct peaks is ruled out. The observed energy differences of 42 cm^–1^ (Δ*E*_1–2_) and 32 cm^–1^ (Δ*E*_2–3_) are too small to be attributed to CF splitting in the excited ^2^F_5/2_ manifold, ruling out the origin of the splitting to hot bands. Crucially, the observed emission wavelength-dependent excitation profiles indicate the presence of different Yb^3+^ sites with slightly varying coordination geometries. Overall, the Yb^3+^-doped cluster shows interesting wavelength-dependent dual Bi^3+^-based and Er^3+^-based PL and PLE. Further, site-selective Yb^3+^-based emissions in the NIR range with temperature dependence are observed, likely useful for applications such as temperature sensing.

### Photophysical Studies of **C-2_d_:Dy**

Temperature-dependent PL and PLE studies of dysprosium-doped BiO-NC **C-2**_**d**_ **:Dy** revealed dual emission at cryogenic temperatures (Figure S80). The emission is composed of Bi^3+^-based phosphorescence, as inferred from the lifetime studies presented below, and f–f transitions (^4^F_9/2_ → ^6^H_*J*_; *J* = 15/2, 13/2, 11/2, or 9/2; Table S41) from the Dy^3+^ centers. The ^4^F_9/2_ → ^6^H_15/2_ Dy^3+^-transition was used to calculate the energy separation (69 cm^–1^) between the ground state and the first excited state (Figure S81). The Bi^3+^-based phosphorescence is absent at 200 and 300 K, as reported for undoped BiO-NCs,^61^ leading to prominent Dy^3+^-based f–f transitions. The temperature dependence of the emission characteristic of the cluster is reflected on the emission colors. The CIE coordinates obtained at 2.4 K (*x* = 0.477 and *y* = 0.469) and 300 K (*x* = 0.404 and *y* = 0.416) indicate warm yellow-orange and yellow-white emission colors, respectively (cf. Figure S82). PLE studies of the cluster in the range of 2.4 to 300 K by monitoring emission at 577 nm (Dy^3+^; ^4^F_9/2_ → ^6^H_13/2_) revealed the presence of a broad excitation peak at 310 nm (Bi^3+^-based transition), as well as a cohort of relatively narrow and intense transitions involving Dy^3+^ (Table S42). In line with the temperature-dependent PL studies, intensity weakening of the Bi^3+^-based band centered at 310 nm is observed with temperature increase. As a consequence of such weakening, the Dy^3+^-based transitions can be clearly observed in the PLE spectra collected at 200 and 300 K (Figure S80). Emission monitoring at 640 nm (Bi^3+^; 2.4 and 77 K) resulted in the observation of a broad transition centered at 310 nm and a shoulder at 340 nm. Remarkably, the Dy^3+^-based narrow transitions are not observed when the emission was monitored at 640 nm. From the above analysis we infer that the Dy^3+^-based emission can be observed via Bi^3+^-based sensitization, on excitation at 310 nm, and direct f–f excitation, e.g., in the visible region at 448 nm.^65,73,74^

Taking advantage of the presence of the dual excitation at 2.4 K, we investigated the wavelength dependence of the emission characteristics of **C-2**_**d**_ **:Dy** (Figure 9). As discussed above, excitation at 310 nm resulted in a dual emission. Excitation of various transitions in the range of 340 to 448 nm also revealed dual emission (Figure S83). We assume that Bi^3+^-based emission can still be observed upon excitation of f–f transitions, particularly at 448 nm, resulting from complex energy-transfer processes involving Bi^3+^ and Dy^3+^ centers. Excitation of various transitions in the range of 312 to 448 nm at 300 K solely revealed Dy^3+^-based emissions (Figure S84).

**Figure 9:**
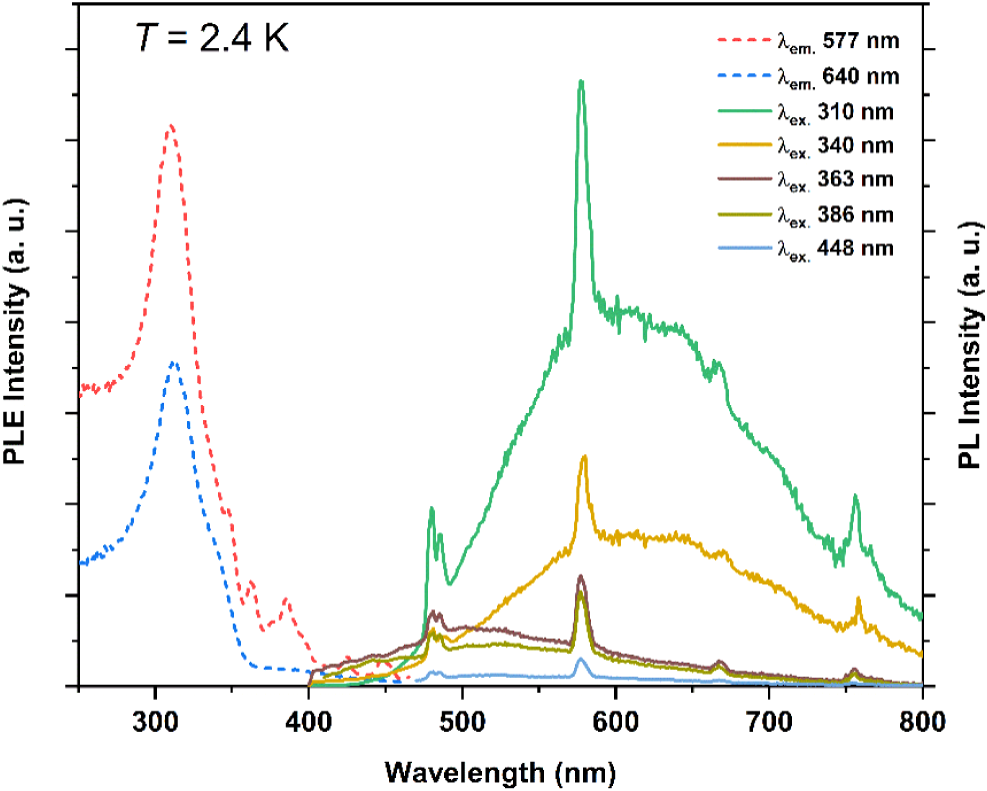
Wavelength-dependent PLE and PL spectra of BiO-NC **C-2_d_:Dy** at 2.4 K.

Lifetime studies for the cluster were performed in the temperature range from 2.4 to 300 K. At 2.4 K, the studies were performed by monitoring the emission at 577 nm (Dy^3+^) and 640 nm (Bi^3+^). The decay profile corresponding to the Bi^3+^-based emission can be satisfactorily fitted with a biexponential function, yielding τ_1_ = 36 μs and τ_2_ = 101 μs (Figure S85, Table S43). These values are comparable with the ones obtained for the undoped BiO-NCs with τ_1_ = 38 μs and τ_2_ = 110 μs.^61^ When the Dy^3+^-based emission at 577 nm is analyzed, the decay profile can be satisfactorily fitted with a triexponential function providing the following values: τ_1_ = 51 μs, τ_2_ = 169 μs, and τ_3_ = 573 μs (Figure S85). Due to the dual emission characteristics of the cluster, it is not possible to exclusively probe the Dy^3+^-based emission at 577 nm, as a result of the simultaneous Bi^3+^-based phosphorescence. The triexponential decay is a result of dual emission with the presence of Bi^3+^-based phosphorescence (τ_1_, τ_2_) and the longer τ_3_ component, which is assigned to the Dy^3+^-based (^4^F_9/2_ → ^6^H_13/2_) emission.

Interestingly, the profile at 77 K obtained by monitoring the emission at 577 nm (λ_ex._ = 312 nm) can be satisfactorily fitted with a monoexponential function yielding a lifetime of 432 μs (Figure S86, Table S44), comparable with the long Dy^3+^-based component obtained at 2.4 K (Figure S85). A close inspection of the decay profile reveals the presence of a short component in the low μs regime, attributed to the Bi^3+^-based emission. On exciting the sample at 319 nm using a pulsed LED source and monitoring emission at 640 nm, we did record a decay profile that allows to be fitted using a monoexponential function. A lifetime of 3.6 μs is obtained, indicating temperature-mediated quenching of the Bi^3+^-based emission. The profiles obtained at 200 and 300 K (λ_ex._ = 312 nm; λ_em._ = 577 nm) can be satisfactorily fitted with a monoexponential function yielding lifetimes of 467 μs (200 K) and 474 μs (300 K) (Figure S87, Table S45). Additionally, the short component observed in the 77 K profile is absent at 200 K and 300 K indicating the quenching of the Bi^3+^-based emission with respect to temperature. Such observations are in line with the steady-state emission profiles recorded at 200 K and 300 K, see the discussion above. Our attempt to study the lifetime of the Bi^3+^-based transition at 640 nm (*T* = 200 K) upon excitation with the LED source was insufficient to detect the weak emissive nature of the **C-2**_**d**_ **:Dy**.

In summary, **C-2**_**d**_ **:Dy** is dual emissive at cryogenic temperatures as a consequence of μs long lifetimes associated with the excited states of Bi^3+^ (^3^P_1_) and Dy^3+^ (^4^F_9/2_). The Dy^3+^-based emission persists at 300 K with a 474 μs lifetime, indicating the absence of channels facilitating nonradiative decay of the emissive ^4^F_9/2_ state. The estimated lifetimes of the Dy^3+^-based ^4^F_9/2_ state fall in the range from 573 μs (2.4 K) to 474 μs (300 K), which are in the upper range reported for Dy^3+^ incorporated in metal oxide matrices,^75,76^ but much higher compared to values obtained for molecular systems, typically several 10s of μs.^77^ This indicates the presence of Dy^3±^ centers in the inner core of the cluster, decoupled from the quenching molecular vibrations in the BiO-NCs periphery.

### Magnetic Studies

The *dc* magnetic susceptibility of **C-2**_**d**_ **:Gd** and **C-2**_**d**_ **:Dy** was measured in an applied magnetic field of 0.05 T over a temperature range of 2 K–300 K. Molar susceptibilities at 300 K of the BiO-NCs were determined with χ_M_ *T* = 5.10 cm^3^·K·mol^–1^ for **C-2**_**d**_ **:Gd** and with χ_M_ *T* = 10.00 cm^3^·K·mol^–1^ for **C-2**_**d**_ **:Dy** (Figure 10, Tables S46, S47). When cooled down, χ_M_ *T* of **C-2**_**d**_ **:Gd** gradually decreases to 4.93 cm^3^·K·mol^–1^ at 40 K and sharply declines to 3.67 cm^3^·K·mol^–1^ at 2 K. In the case of **C-2**_**d**_ **:Dy**, χ_M_ *T* drops steadily to 9.37 cm^3^·K·mol^–1^ at 90 K and then rapidly to 7.20 cm^3^·K·mol^–1^ at 2 K. The decrease of the χ_M_ *T* can be attributed to the depopulation of the individual Stark levels split by the crystal field. No hysteresis or macroscopic magnetization was observed. The magnetic susceptibilities are smaller than the values expected for an isolated Gd^3+^ (^8^S_7/2_ ground state) and Dy^3+^ (^6^H_15/2_ ground state) ion with 7.88 cm^3^·K·mol^–1^ and 14.17 cm^3^·K·mol^–1^ respectively, however, the magnetic behavior is in agreement with some mononuclear Gd^3+^ and Dy^3+^ complexes.^78^ This deviation in experimental and theoretical values corresponds well to the core composition of [Bi_38–*x*_Ln_*x*_O_45_]^24+^ (*x* < 1), as indicated by ESI-MS and ICP-OES results. The bulk material consists primarily of undoped BiO-NCs, followed by heterobimetallic BiO-NCs substituted with a single lanthanoid, which do not show intermolecular coupling. In cases where multiple lanthanoids are incorporated into a single cluster, coupling is theoretically feasible, but no indication of such interactions is observed. This is the result of first, a small fraction of multiply doped BiO-NCs and second that these do not seem to be magnetically coupled, or the coupling is too weak.

**Figure 10:**
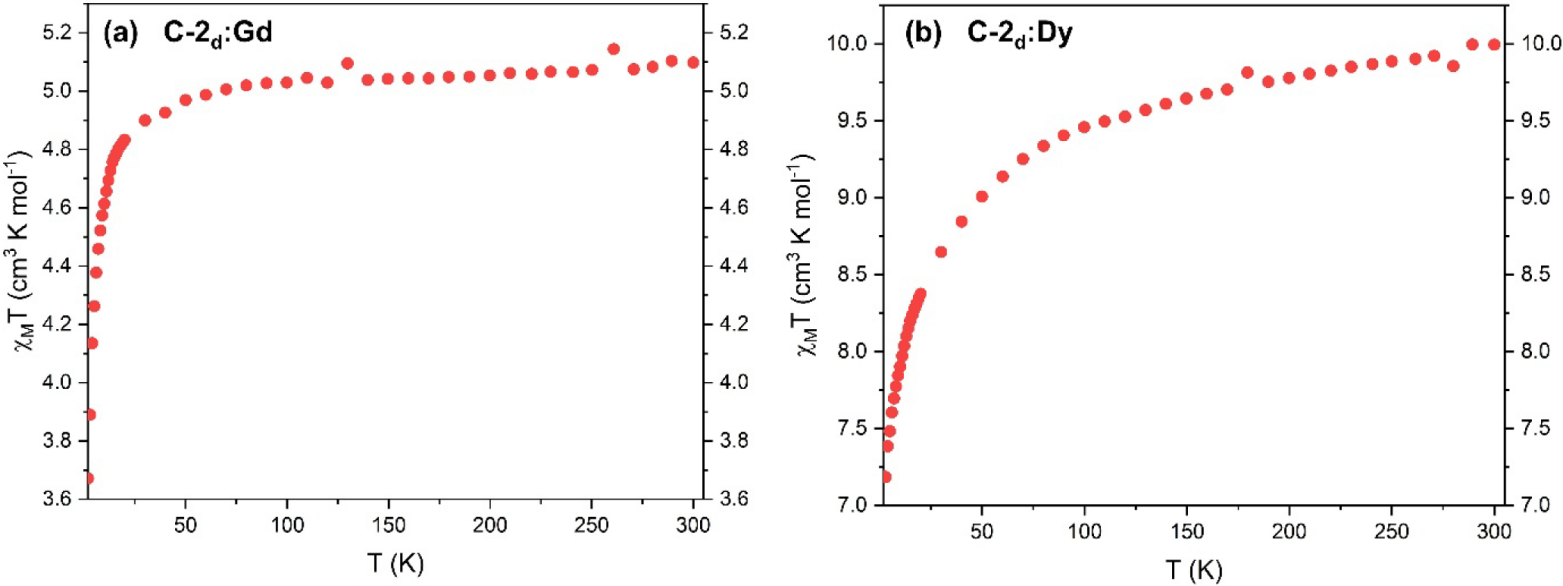
Temperature dependence of the χ_M_ *T* values from susceptibility measurements of BiO-NC **C-2_d_:Gd** (a) and **C-2_d_:Dy** (b) at 0.05 T.

### NMR

As a result of the incorporation of paramagnetic Dy^3+^ and Gd^3+^ dopants, a strong broadening of the ^1^H and ^13^C NMR signals in solution assigned to the methacrylate ligands of **C-2**_**d**_ **:Dy** and **C-2**_**d**_ **:Gd** was obtained as compared to the pristine BiO-NC (cf. Figures S88–S93). In the solid-state ^1^H NMR, only a broad background signal appears for **C-2**_**d**_ **:Gd**, whereas for **C-2**_**d**_ two separate signals for the methacrylate ligands are observed (Figure S94). Similarly, in the ^13^C­{^1^H} CP MAS NMR spectra **C-2**_**d**_ **:Gd** exhibits only signals with low intensity, which can hardly be distinguished from the background noise, whereas the BiO-NC **C-2**_**d**_ shows all respective ^13^C NMR signals for the methacrylate ligands (Figure S95). However, ^13^C­{^1^H} MAS NMR measurements of **C-2**_**d**_ **:Gd** without CP but using background canceling revealed a much better signal-to-noise ratio with all expected signals and interestingly indicates a fast relaxation time *d*_1_ = 6 s as a result of the presence of the Gd^3+^ dopant (Figure S96). The absence of additional signals from dmso or ethanol proves the success of the drying process for sample **C-2**_**d**_ **:Gd**, which still is soluble in diverse organic solvents.

### DNP

Dynamic nuclear polarization (DNP) increases the intrinsically low sensitivity of nuclear magnetic resonance (NMR) by transferring the high electron spin polarization to nearby nuclei using microwave irradiation.^79^ This process requires the presence of paramagnetic species in the NMR sample. In practice, stable organic radicals or high-spin metal ions are employed as polarization agents (PAs), whose performance is primarily determined by their electron paramagnetic resonance (EPR) properties.^80^ Gd^3+^, with its 4f^7^ electronic configuration, a high-spin ground state (*S* = 7/2), and negligible *g*-anisotropy, is a well-established PA. Gd^3+^ is commonly used in the form of chelated complexes, such as Gd-DOTA.^81^ However, even the free ion has been shown to yield substantial DNP enhancement factors.^58,60,81,82^ Motivated by this, we conducted preliminary investigations of Gd^3+^ incorporated within the respective bismuth oxido clusters to assess their potential as intrinsic sources of polarization in DNP-enhanced MAS NMR experiments.

As a proof of concept, we investigated the potential of Gd^3+^ incorporated into the BiO-NCs as PAs for solid-state DNP. To this end, pure **C-2**_**d**_ **:Gd** powder was packed into a solid-state NMR rotor and analyzed at 100 K and 6 kHz MAS frequency. Experiments were performed at the positive solid-effect DNP (SE-DNP) matching condition for protons, both with and without continuous microwave irradiation. Without microwave irradiation no signals were observed in the ^1^H–^13^C cross-polarization (CP) spectra of **C-2**_**d**_ **:Gd**. However, all methacrylate resonances were clearly observed under microwave irradiation (cf. Figure 11). Based on the CP spectra, no enhancement factor could be determined for **C-2**_**d**_ **:Gd** in a reasonable measurement time. In contrast, direct polarization (DP) experiments on protons result in an enhancement factor of ε ≈ 10 for the periodic rotational bands, which stem from protons inside the MAS rotor; as expected, a larger, unstructured background signal does not show any signal for DNP enhancement. In the case of cluster **C-1:Gd**, the determination of proton enhancement factors was successful using DP and CP experiments (cf. Figure S97). We herein observed a proton enhancement factor of ε = 10 for Gd^3+^ doped BiO-NCs in the solid state. Similar values were reported for Gd^3+^ containing complexes dissolved in the standard DNP matrix of glycerol-*d*_8_:D_2_O:H_2_O (60:30:10 vol %).^82,83^

**Figure 11:**
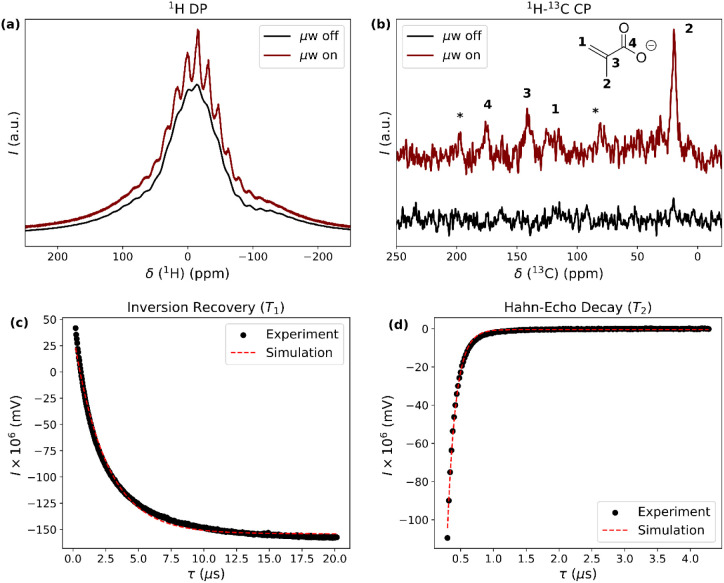
*Top:* ^1^H DP (a) and ^1^H–^13^C DPMAS (b) spectra of pure **C-2_d_:Gd** at 100 K and 6 kHz MAS frequency with (red) and without (black) microwave irradiation (263 GHz) at the positive ^1^H solid effect DNP matching condition. Rotational sidebands are marked with an asterisk. *Bottom:* Inversion recovery (c) and Hahn-echo decay (d) relaxation curves for **C-2_d_:Gd** measured at 10 K at 180 GHz microwave frequency. Simulations (red) of the experimental (black) data were performed using an exponential model. The resulting electron relaxation times are *T*_1e_ = 2.45 μs and *T*_2e_ = 0.13 μs.

To further characterize the electron spin properties of the clusters, electron spin relaxation times (*T*_1e_ and *T*_2e_) were determined using inversion recovery and Hahn-echo decay experiments at G-band (180 GHz). The relaxation times were measured at the maximum of the echo-detected field sweep (cf. Figure S98). Measurements at 10 K yielded *T*_1e_ = 2.45 μs and *T*_2e_ = 0.13 μs using a G-band (180 GHz) EPR spectrometer. Even though the conditions are difficult to directly compare (e.g., different field strengths, sample constitution, etc.), these values are about 1–2 orders of magnitude shorter than those reported for Gd-DOTA—the most widely used Gd^3+^-based PA—using a W-band EPR spectrometer^83,84^ at the same temperature in frozen solutions. We attribute this reduction in relaxation time constants to the starkly different chemical environment of the Gd^3+^ ions, in particular the vicinity of the heavy Bi atoms. Since we find only a negligible amount of BiO-NCS with more than one Gd^3+^ atom being present per cluster it is highly unlikely that strong spin–spin interactions between Gd^3+^ centers are responsible for this reduction. Furthermore, interactions between Gd^3+^ in neighboring clusters are expected to cause much smaller effects; for example, the coupling of two nitroxides with a distance of about 1.3 nm^85^ only causes a reduction in both *T*_1e_ and *T*_2e_ of ∼30%.^86^

While these initial findings highlight the potential of Gd^3+^ doped BiO-NCs as solid-state MAS PA agent, a systematic, concentration- and temperature-dependent, EPR study is necessary to fully characterize the clusters’ electron properties, followed by an in-depth DNP investigation. TGA analysis (Figure S99) shows that the BiO-NCs are stable up to about 100 °C (**C-1:Gd**) and 150 °C (**C-2**_**d**_ **:Gd**), respectively, which mark the limit of their stability as PA. Nonetheless, the current data suggest that these clusters can be used for electron–nuclear polarization transfer, potentially enabling more detailed studies of surface structures and local environments.

## Conclusion

In this study, we report on the synthesis of lanthanoid-doped bismuth oxido nanoclusters (BiO-NCs) based on the “magic cluster” {Bi_38_O_45_} motif, demonstrating their suitability for studying dopant effects in "soluble metal oxides". The doping of the BiO-NCs **C-1:Ln** was proven by ESI-MS showing a mixture (≈1:1) of undoped and doped cluster molecules, in accordance with the dopant content of about 1 ω% as determined by ICP-OES. SC-XRD proved the respective doped and undoped nitrate-functionalized BiO-NCs to be isostructural. A statistical, albeit nonuniform distribution of the dopants at different Bi^3+^ positions within the cluster core is observed, however, with preference of substitution of the inner core positions of [Bi_38_O_45_(NO_3_)_24_(dmso)_28–*y*_]:Ln (**C-1:Ln**, *y* = 0–2). Overall the dopant content of about ≈1 ω% was sufficient to significantly modify the BiO-NCs’ optical and magnetic properties. Dual Bi^3+^- and Ln^3+^-based PLE and PL, with mutual effects such as energy transfer from Bi^3+^ to Ln^3+^, and lifetime quenching (Er^3+^) were observed in the BiO-NCs doped with Er^3+^, Yb^3+^, and Dy^3+^. The absorption as well as the emission spectra of the lanthanoid-doped BiO-NCs vary in a wide range of the electromagnetic spectrum. Especially the different ranges of PL emission with visible light (e.g., Dy^3+^, Sm^3+^, Tb^3+^, Eu^3+^) and NIR light (e.g., Er^3+^, Yb^3+^) make them promising candidates for applications in the area of fluorescent tags or light-emitting devices. To what extent the combinations of lanthanoid doping in BiO-NCs can lead to fine regulation of emission colors or whether they show upconversion PL will be investigated in future work. The lanthanoid doping with Dy^3+^ and Gd^3+^ in the diamagnetic host structure introduced paramagnetic behavior but did not show macroscopic magnetization, which is in line with the conclusions that (i) the majority of doped clusters does contain one lanthanoid cation and (ii) in the rarer cases of multiple doping the lanthanoids are not magnetically coupled. The gadolinium-doped BiO-NCs act as effective polarization agents with an enhancement factor of about 10. Thus, such doped metal oxido clusters might serve as model compounds for studies on surface functionalization of metal oxides using the DNP effect.

## Supplementary Material



## Notes

The SI contains additional experimental details, additional data, visualizations, and a brief discussion on ESI-MS, IR, UV–vis, PL, NMR, and EPR spectroscopy as well as PXRD, SC XRD, SQUID, and DNP Measurements (PDF)
