# Supplementary material for: Atomically Precise Bismuth Oxido Nanoclusters as Hosts for Ln3+: Effects of Doping on Optical and Magnetic Properties of a Soluble Metal Oxide
Source: Inorg Chem. 2026 Jun 19;65(26):14758–74. doi: 10.1021/acs.inorgchem.6c01410 (PMC13343463; doi:10.1021/acs.inorgchem.6c01410)
Supplement: Supplementary file 1 [file ic6c01410_si_001.pdf]

Supporting Information

Atomically Precise Bismuth Oxido Nanoclusters as Hosts for Ln^{3+}: Effects of Doping on Optical and Magnetic Properties of a Soluble Metal Oxide

Rico Thomas[a], Senthil Kumar Kuppusamy[b], Tobias Rüffer[c], Marcus Weber[a,d] Vanessa Stephan[e], Florian Taube[f], Andrei Kuzhelev[g], Björn Corzilius[f], Berthold Kersting[e], Mario Ruben[b,h,i], Michael Mehring[a,d*]*

[a] *University of Technology Chemnitz, Faculty of Natural Sciences, Institute of Chemistry, Professorship of Coordination Chemistry, 09107 Chemnitz, Germany*

[b] *Karlsruhe Institute of Technology, Institute of Quantum Materials and Technology (IQMT), 76344 Eggenstein-Leopoldshafen, Germany*

[c] *University of Technology Chemnitz, Faculty of Natural Sciences, Institute of Chemistry, Professorship of Inorganic Chemistry, 09107 Chemnitz, Germany*

[d] *Center of Materials, Architectures and Integration of Nanomembranes, Chemnitz University of Technology, 09126 Chemnitz, Germany*

[e] *University of Leipzig, Faculty of Chemistry and Mineralogy, Institute of Inorganic Chemistry, 04103 Leipzig, Germany*

[f] *University of Rostock, Institute of Chemistry and Department Life, Light & Matter, Albert-Einstein-Strape 25/27, 18059 Rostock, Germany*

[g] *Goethe University Frankfurt, Institute of Physical and Theoretical Chemistry and Center for Biomolecular Magnetic Resonance (BMRZ), Max von Laue Str. 7, 60438 Frankfurt am Main, Germany*

[h] *Institute of Nanotechnology (INT), Karlsruhe Institute of Technology, Karlsruhe, 76131, Germany.*

[i] *Centre Européen de Sciences Quantiques (CESQ), Institut de Science et d'Ingénierie Supramoléculaires (ISIS), Strasbourg, 67083, France*

***Correspondence: michael.mehring@chemie.tu-chemnitz.de, mario.ruben@kit.edu**

Content

1. Synthesis 3

2. Results 3

ESI-MS Spectra of Doped BiO-NCs: 3

IR Studies: 35

PXRD: 36

Crystal Structure of **C-1**, **C-1’** and **C-1:Gd**: 37

Crystal Structure of **C-1:Dy** and **C-1**: 41

Crystal Structure of **C-2$_E$** and **C-2$_E$:Gd:** 44

UV–vis Studies and Transition Assignment: 46

PL Studies of **C-1:Ln** at ambient conditions: 51

PL Studies of **C-1:Gd**: 52

PL Studies of **C-1:Er**: 53

PL Studies of **C-1:Yb**: 55

PL Studies of **C-2$_d$:Dy**: 59

Magnetic Susceptibility Measurements of BiO-NCs: 64

NMR Studies on Methacrylate Functionalized BiO-NCs: 66

DNP Spectra of **C-1:Gd:** 69

G-band EPR Spectra of **C-2d:Gd:** 69

TGA Analysis of Gd doped BiO-NCs**:** 70

References: 70

1. Synthesis

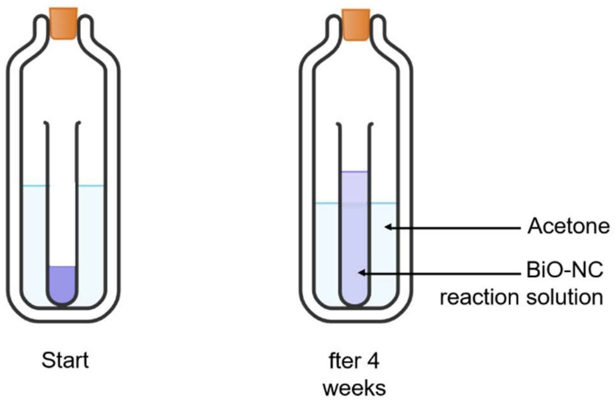

Scheme 1. Model setup for the crystallization of BiO-NCs, a desiccator is used for larger batches.

Table S 1. Summary of relevant parameters (n, m_1, m_2, η) for the synthesis of the different lanthanide doped BiO-NCs.

Lanthanide	n (H_2O, educt)	m_1 (educt) [mg]	m_2 (product) [mg]	η (Yield) [%]
**C-1:La**	6	134.2	314.5	31
**C-1:Pr**	6	134.9	335.3	34
**C-1:Nd**	6	135.9	346.7	35
**C-1:Sm**	6	137.8	361.0	36
**C-1:Gd**	6	139.9	393.0	38
**C-1:Tb**	6	140.4	342.8	34
**C-1:Dy**	6	141.5	379.0	38
**C-1:Ho**	5	136.7	355.1	36
**C-1:Er**	5	137.4	396	40
**C-1:Tm**	5	138.0	345.5	35
**C-1:Yb**	5	139.2	418.0	42
**C-1:Lu**	5	139.8	345.3	34

2. Results

<u>ESI-MS Spectra of Doped BiO-NCs:</u>

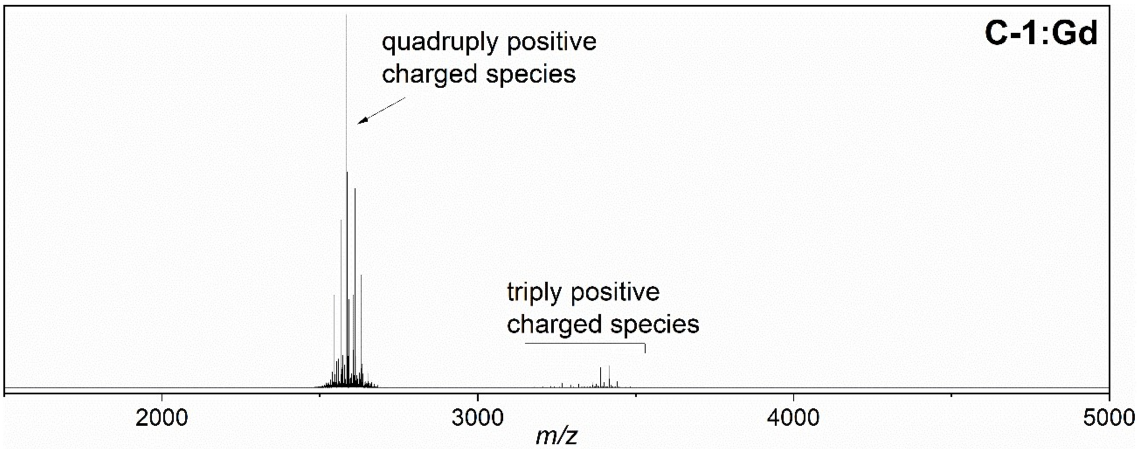

Figure S 1. Survey ESI-MS spectra generated from cluster **C-1:Gd** after electrospraying from MeCN/dmso, with marked triply and quadruply positive charged cationic species.

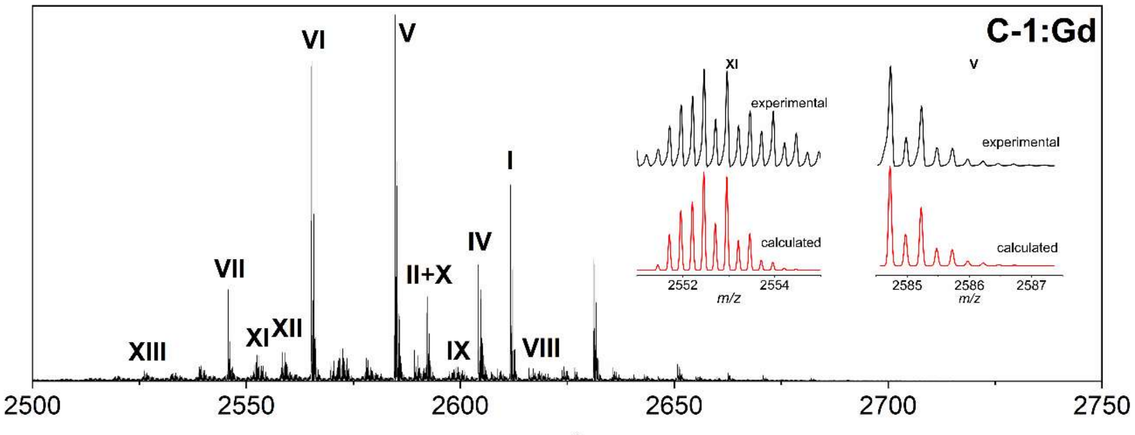

Figure S 2. Part of the ESI mass spectrum of compound **C-1:Gd** electrosprayed from MeCN/dmso showing quadruply positive charged homometallic cations (**I**–**VII**) with $[Bi_{38}O_{46}(NO_3)_{18}(dmso)_7]^{4+}$ (**V**, *m*/*z* = 2611.7214) and heterobimetallic BiO-NC cations (**VIII**–**XIII**) with $[Bi_{37}GdO_{46}(NO_3)_{18}(dmso)_6]^{4+}$ (**XI,** *m*/*z* = 2552.4575) showing the highest abundance.

Table S 2. Selection of quadruply positive charged bismuth oxido nanocluster cations detected in the survey mass spectrum of BiO-NC **C-1:Gd** electrosprayed from MeCN/dmso. Assignment was carried out using the most abundant *m/z* signals.

Label	Cation	*m/z*	
		**calculated**	**BiO-NC C-1:Gd**
**I**	$[Bi_{38}O_{45}(NO_3)_{20}(dmso)_7]^{4+}$	2611.7195	2611.7214
**II**	$[Bi_{38}O_{45}(NO_3)_{20}(dmso)_6]^{4+}$	2592.2160	2592.2174
**IV**	$[Bi_{38}O_{46}(NO_3)_{18}(dmso)_8]^{4+}$	2604.2278	2604.2313
**V**	$[Bi_{38}O_{46}(NO_3)_{18}(dmso)_7]^{4+}$	2584.7243	2584.7299
**VI**	$[Bi_{38}O_{46}(NO_3)_{18}(dmso)_6]^{4+}$	2565.2208	2565.2245
**VII**	$[Bi_{38}O_{46}(NO_3)_{18}(dmso)_5]^{4+}$	2545.7174	2545.7170
**VIII**	$[Bi_{37}GdO_{45}(NO_3)_{20}(dmso)_8]^{4+}$	2618.4589	2618.4657
**IX**	$[Bi_{37}GdO_{45}(NO_3)_{20}(dmso)_7]^{4+}$	2598.9554	2598.9611
**X**	$[Bi_{37}GdO_{46}(NO_3)_{18}(dmso)_8]^{4+}$	2591.4637	2591.4653
**XI**	$[Bi_{37}GdO_{46}(NO_3)_{18}(dmso)_6]^{4+}$	2552.4568	2552.4575
**XII**	$[Bi_{36}Gd_2O_{46}(NO_3)_{18}(dmso)_7]^{4+}$	2559.1961	2559.2104
**XIII**	$[Bi_{35}Gd_3O_{46}(NO_3)_{18}(dmso)_6]^{4+}$	2526.4437	2526.4283

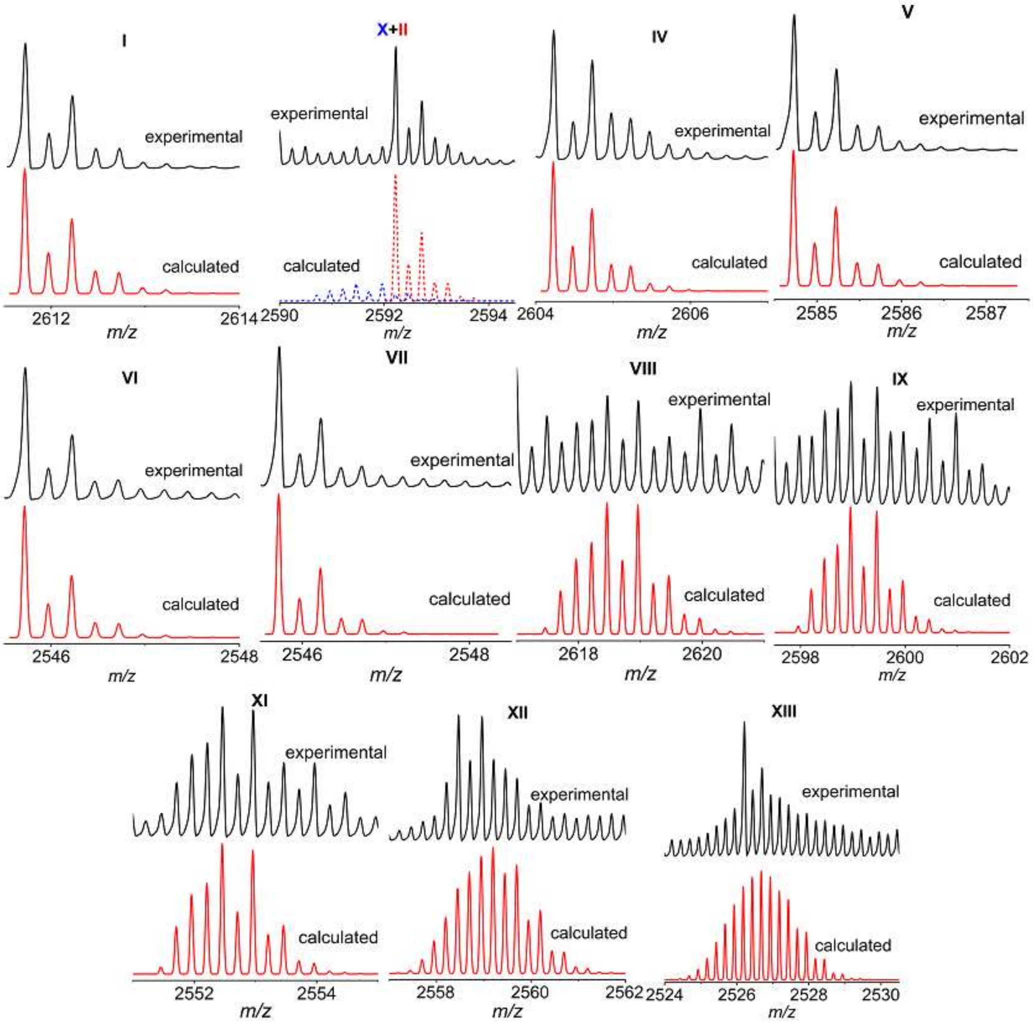

Figure S 3. Isotopic patterns (exp. and calcd.) of different quadruply positive charged bismuth oxido nanocluster cations detected in the gas phase generated from compound **C-1:Gd** after electrospraying from MeCN/dmso. $[Bi_{38}O_{45}(NO_3)_{20}(dmso)_7]^{4+}$ (**I**), $[Bi_{38}O_{45}(NO_3)_{20}(dmso)_6]^{4+}$ (**II**), $[Bi_{38}O_{46}(NO_3)_{18}(dmso)_8]^{4+}$ (**IV**), $[Bi_{38}O_{46}(NO_3)_{18}(dmso)_7]^{4+}$ (**V**), $[Bi_{38}O_{46}(NO_3)_{18}(dmso)_6]^{4+}$ (**VI**), $[Bi_{38}O_{46}(NO_3)_{18}(dmso)_5]^{4+}$ (**VII**), $[Bi_{37}GdO_{45}(NO_3)_{20}(dmso)_8]^{4+}$ (**VIII**), $[Bi_{37}GdO_{45}(NO_3)_{20}(dmso)_7]^{4+}$ (**IX**), $[Bi_{37}GdO_{46}(NO_3)_{18}(dmso)_8]^{4+}$ (**X**), $[Bi_{37}GdO_{46}(NO_3)_{18}(dmso)_6]^{4+}$ (**XI**), $[Bi_{36}Gd_2O_{46}(NO_3)_{18}(dmso)_7]^{4+}$ (**XII**), $[Bi_{36}Gd_3O_{46}(NO_3)_{18}(dmso)_6]^{4+}$ (**XIII**).

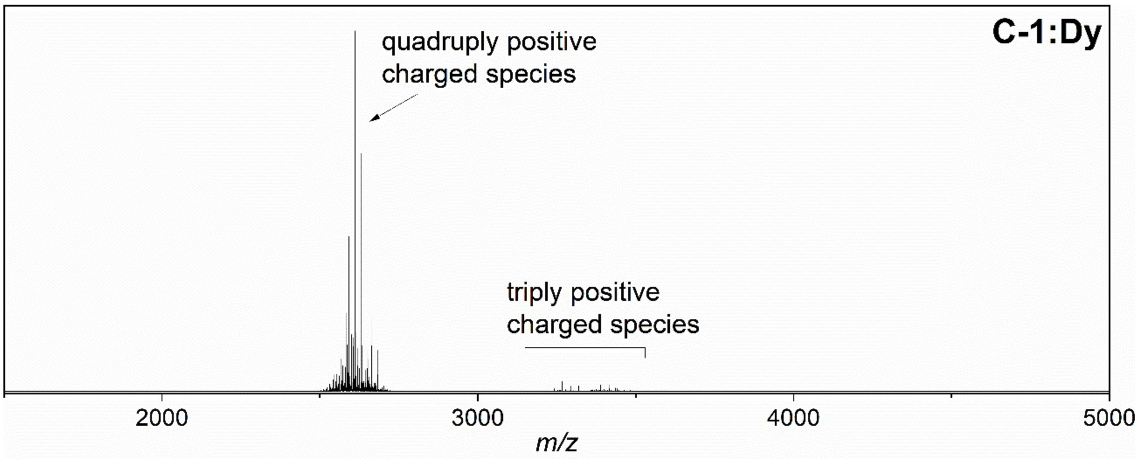

Figure S 4. Survey ESI-MS spectra generated from cluster **C-1:Dy** after electrospraying from MeCN/dmso, with marked triply and quadruply positive charged cationic species.

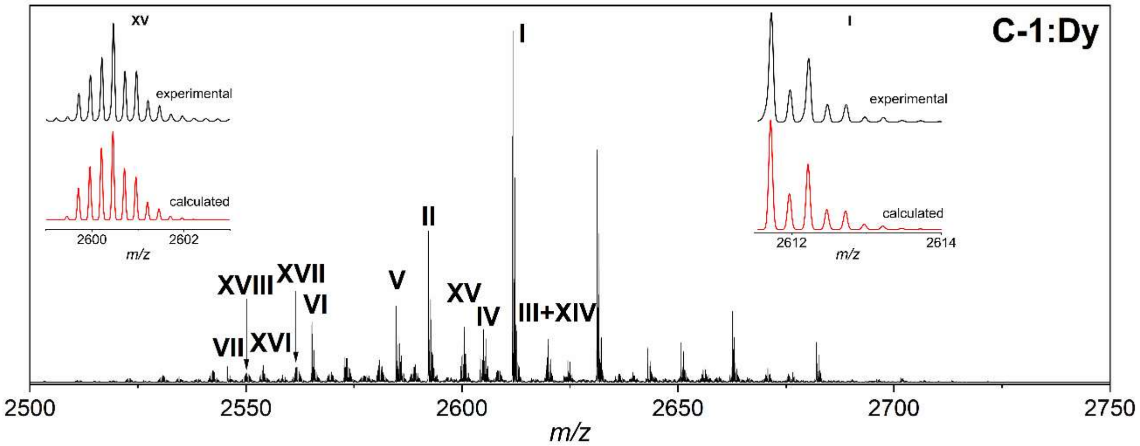

Figure S 5. Part of the ESI mass spectrum of compound **C-1:Dy** electrosprayed from MeCN/dmso showing quadruply positive charged homometallic cations (**I**–**VII**) with $[Bi_{38}O_{45}(NO_3)_{20}(dmso)_7]^{4+}$ (**I**, *m*/*z* = 2611.7214) and heterobimetallic BiO-NC cations (**XIV**–**XVIII**) with $[Bi_{37}DyO_{45}(NO_3)_{20}(dmso)_7]^{4+}$ (**XV**, *m*/*z* = 2600.2064) showing the highest abundance.

Table S 3. Selection of quadruply positive charged bismuth oxido nanocluster cations detected in the survey mass spectrum of BiO-NC **C-1:Dy** electrosprayed from MeCN/dmso. Assignment was carried out using the most abundant *m/z* signals.

**Label**	**Cation**	***m/z***	
		**calculated**	**BiO-NC C-1:Dy**
**I**	$[Bi_{38}O_{45}(NO_3)_{20}(dmso)_7]^{4+}$	2611.7195	2611.7272
**II**	$[Bi_{38}O_{45}(NO_3)_{20}(dmso)_6]^{4+}$	2592.2160	2592.2250
**III**	$[Bi_{38}O_{45}(NO_3)_{19}(OH)(dmso)_8]^{4+}$	2619.9767	2619.9693
**V**	$[Bi_{38}O_{46}(NO_3)_{18}(dmso)_7]^{4+}$	2584.7243	2584.7334
**VI**	$[Bi_{38}O_{46}(NO_3)_{18}(dmso)_6]^{4+}$	2565.2208	2565.2282
**VII**	$[Bi_{38}O_{46}(NO_3)_{18}(dmso)_5]^{4+}$	2545.7174	2545.7257
**XIV**	$[Bi_{37}DyO_{45}(NO_3)_{20}(dmso)_8]^{4+}$	2619.7099	2619.7181
**XV**	$[Bi_{37}DyO_{45}(NO_3)_{20}(dmso)_7]^{4+}$	2600.2064	2600.2159
**XVI**	$[Bi_{37}DyO_{46}(NO_3)_{18}(dmso)_6]^{4+}$	2553.9579	2553.9695
**XVII**	$[Bi_{36}Dy_2O_{46}(NO_3)_{18}(dmso)_7]^{4+}$	2561.6981	2561.7095
**XVIII**	$[Bi_{35}Dy_3O_{46}(NO_3)_{18}(dmso)_7]^{4+}$	2550.1851	2550.1973

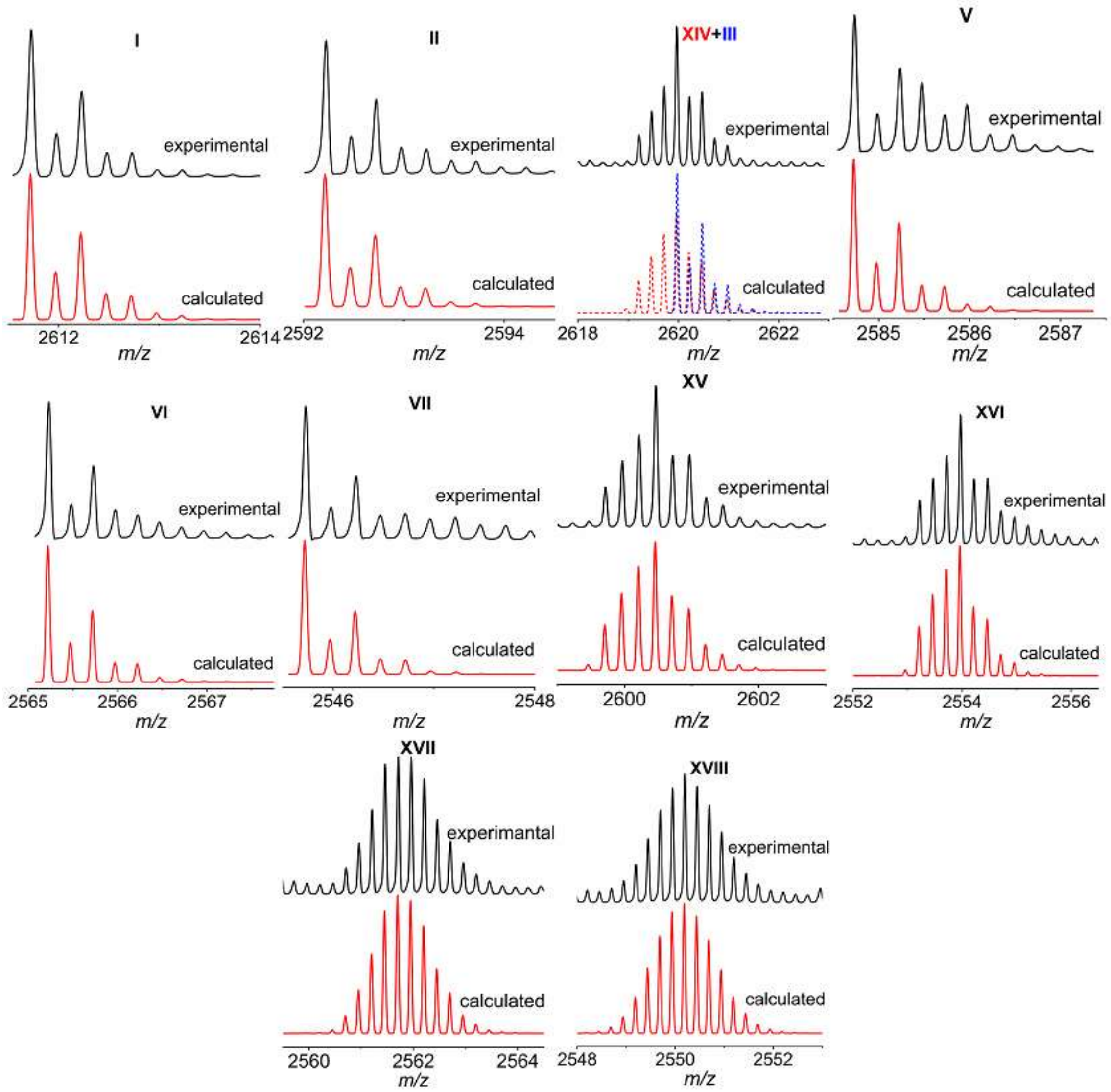

Figure S 6. Isotopic patterns (exp. and calcd.) of different quadruply positive charged bismuth oxido nanocluster cations detected in the gas phase generated from compound **C-1:Dy** after electrospraying from MeCN/dmso. $[Bi_{38}O_{45}(NO_3)_{20}(dmso)_7]^{4+}$ (**I**), $[Bi_{38}O_{45}(NO_3)_{20}(dmso)_6]^{4+}$ (**II**), $[Bi_{38}O_{45}(NO_3)_{19}(OH)(dmso)_8]^{4+}$ (**III**), $[Bi_{38}O_{46}(NO_3)_{18}(dmso)_7]^{4+}$ (**V**), $[Bi_{38}O_{46}(NO_3)_{18}(dmso)_6]^{4+}$ (**VI**), $[Bi_{38}O_{46}(NO_3)_{18}(dmso)_5]^{4+}$ (**VII**), $[Bi_{37}DyO_{45}(NO_3)_{20}(dmso)_8]^{4+}$ (**XIV**), $[Bi_{37}DyO_{45}(NO_3)_{20}(dmso)_7]^{4+}$ (**XV**), $[Bi_{37}DyO_{46}(NO_3)_{18}(dmso)_6]^{4+}$ (**XVI**), $[Bi_{36}Dy_2O_{46}(NO_3)_{18}(dmso)_7]^{4+}$ (**XVII**), $[Bi_{35}Dy_3O_{46}(NO_3)_{18}(dmso)_7]^{4+}$ (**XVIII**).

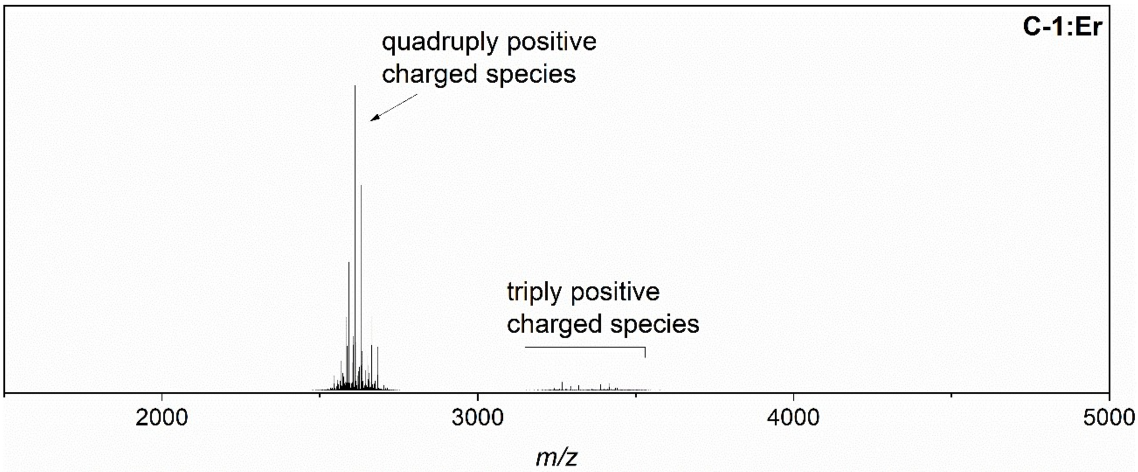

Figure S 7. Survey ESI-MS spectra generated from cluster **C-1:Er** after electrospraying from MeCN/dmso, with marked triply and quadruply positive charged cationic species.

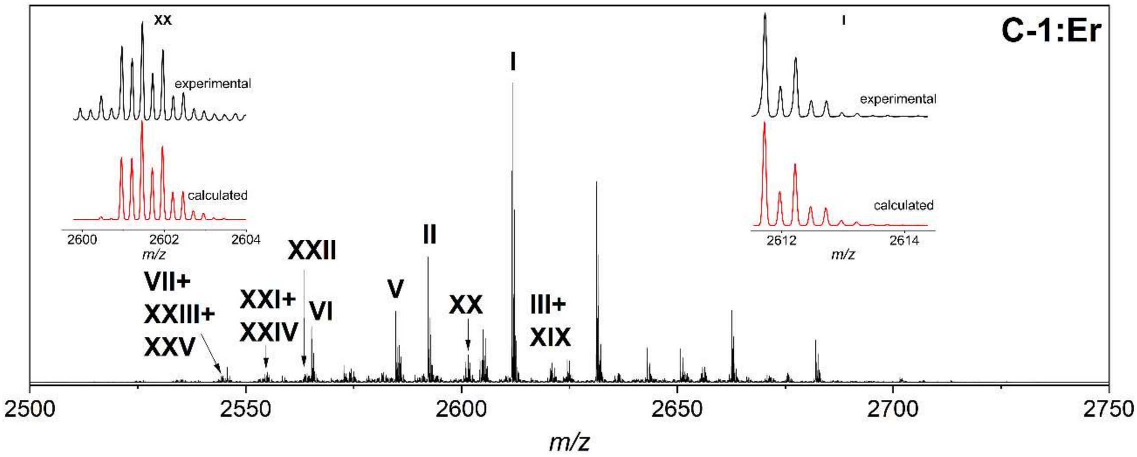

Figure S 8. Part of the ESI mass spectrum of compound **C-1:Er** electrosprayed from MeCN/dmso showing quadruply positive charged homometallic cations (**I**–**VII**) with $[Bi_{38}O_{45}(NO_3)_{20}(dmso)_7]^{4+}$ (**I**, *m/z* = 2611.7214) and heterobimetallic BiO-NC cations (**XIX**–**XXV**) with $[Bi_{37}ErO_{45}(NO_3)_{20}(dmso)_7]^{4+}$ (**XX**, *m/z* = 2601.4661) showing the highest abundance.

Table S 4. Selection of quadruply positive charged bismuth oxido nanocluster cations detected in the survey mass spectrum of BiO-NC **C-1:Er** electrosprayed from MeCN/dmso. Assignment was carried out using the most abundant *m/z* signals.

Label	Cation	*m/z*	
		calculated	BiO-NC C-1:Er
I	$[Bi_{38}O_{45}(NO_3)_{20}(dmso)_7]^{4+}$	2611.7195	2611.7277
II	$[Bi_{38}O_{45}(NO_3)_{20}(dmso)_6]^{4+}$	2592.2160	2592.2252
III	$[Bi_{38}O_{45}(NO_3)_{19}(OH)(dmso)_8]^{4+}$	2619.9767	2619.9766
V	$[Bi_{38}O_{46}(NO_3)_{18}(dmso)_7]^{4+}$	2584.7243	2584.7337
VI	$[Bi_{38}O_{46}(NO_3)_{18}(dmso)_6]^{4+}$	2565.2208	2565.2306
VII	$[Bi_{38}O_{46}(NO_3)_{18}(dmso)_5]^{4+}$	2545.7174	2545.7256
XIX	$[Bi_{37}ErO_{45}(NO_3)_{20}(dmso)_8]^{4+}$	2620.9608	2620.9685
XX	$[Bi_{37}ErO_{45}(NO_3)_{20}(dmso)_7]^{4+}$	2601.4573	2601.4661
XXI	$[Bi_{37}ErO_{46}(NO_3)_{18}(dmso)_6]^{4+}$	2554.9587	2554.9675
XXII	$[Bi_{36}Er_2O_{46}(NO_3)_{18}(dmso)_7]^{4+}$	2564.2001	2564.2107
XXIII	$[Bi_{36}Er_2O_{46}(NO_3)_{18}(dmso)_6]^{4+}$	2544.6967	2544.7071
XXIV	$[Bi_{35}Er_3O_{46}(NO_3)_{18}(dmso)_7]^{4+}$	2553.9382	2553.9531
XXV	$[Bi_{34}Er_4O_{46}(NO_3)_{18}(dmso)_7]^{4+}$	2543.1758	2543.1929

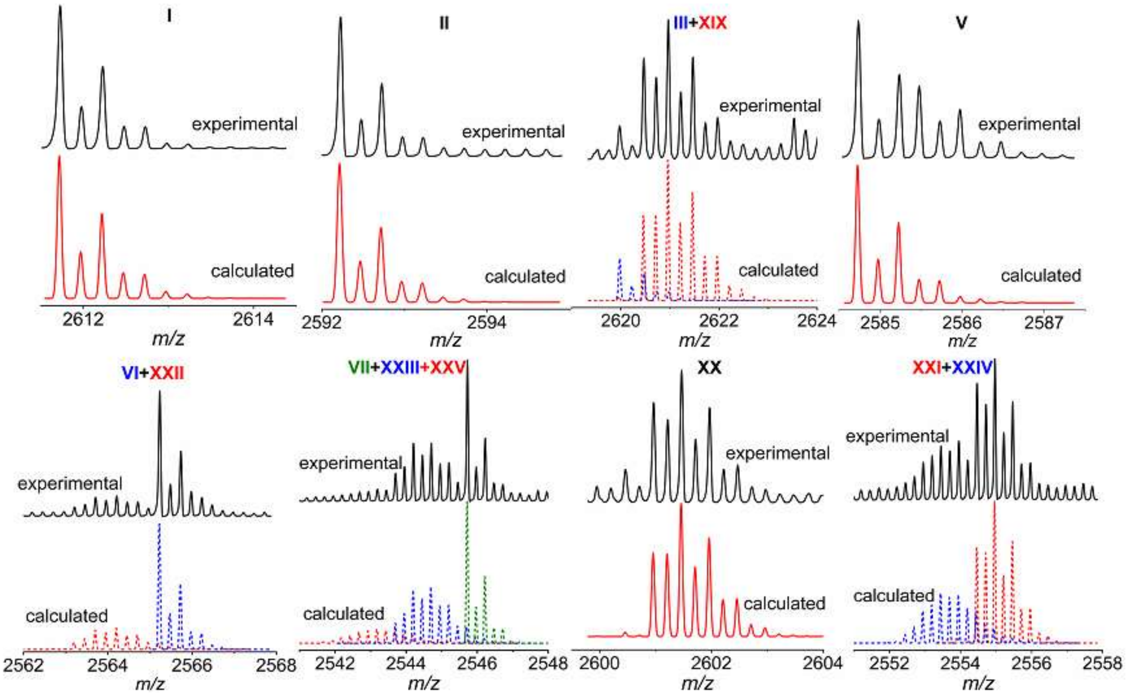

Figure S 9. Isotopic patterns (exp. and calcd.) of different quadruply positive charged bismuth oxido nanocluster cations detected in the gas phase generated from compound **C-1:Er** after electrospraying from MeCN/dmso. $[Bi_{38}O_{45}(NO_3)_{20}(dmso)_7]^{4+}$ (**I**), $[Bi_{38}O_{45}(NO_3)_{20}(dmso)_6]^{4+}$ (**II**), $[Bi_{38}O_{45}(NO_3)_{19}(OH)(dmso)_8]^{4+}$ (**III**), $[Bi_{38}O_{46}(NO_3)_{18}(dmso)_7]^{4+}$ (**V**), $[Bi_{38}O_{46}(NO_3)_{18}(dmso)_6]^{4+}$ (**VI**), $[Bi_{38}O_{46}(NO_3)_{18}(dmso)_5]^{4+}$ (**VII**), $[Bi_{37}ErO_{45}(NO_3)_{20}(dmso)_8]^{4+}$ (**XIX**), $[Bi_{37}ErO_{45}(NO_3)_{20}(dmso)_7]^{4+}$ (**XX**), $[Bi_{37}ErO_{46}(NO_3)_{18}(dmso)_6]^{4+}$ (**XXI**), $[Bi_{36}Er_2O_{46}(NO_3)_{18}(dmso)_7]^{4+}$(**XXII**), $[Bi_{36}Er_2O_{46}(NO_3)_{18}(dmso)_6]^{4+}$ (**XXIII**), $[Bi_{35}Er_3O_{46}(NO_3)_{18}(dmso)_7]^{4+}$(**XXIV**), $[Bi_{34}Er_4O_{46}(NO_3)_{18}(dmso)_7]^{4+}$ (**XXV**).

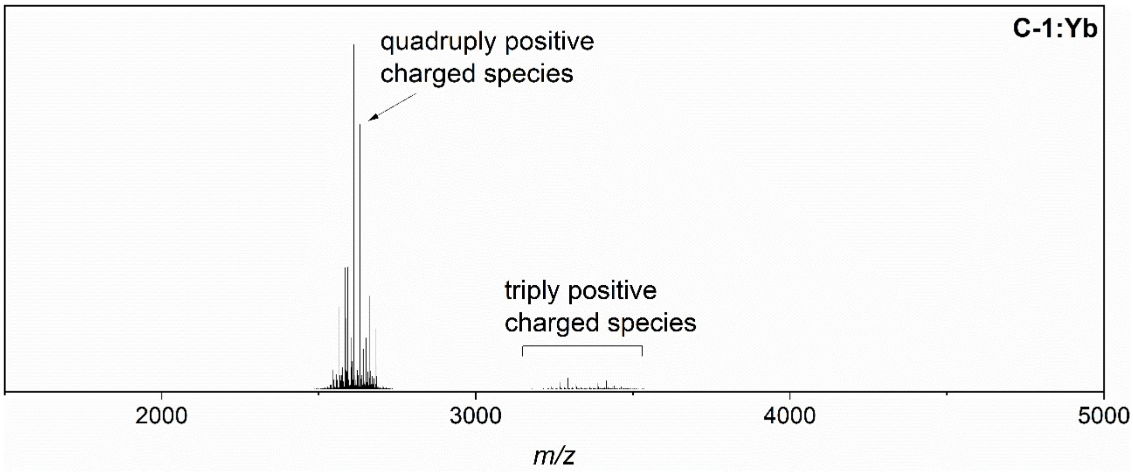

Figure S 10. Survey ESI-MS spectra generated from cluster **C-1:Yb** after electrospraying from MeCN/dmso, with marked triply and quadruply positive charged cationic species.

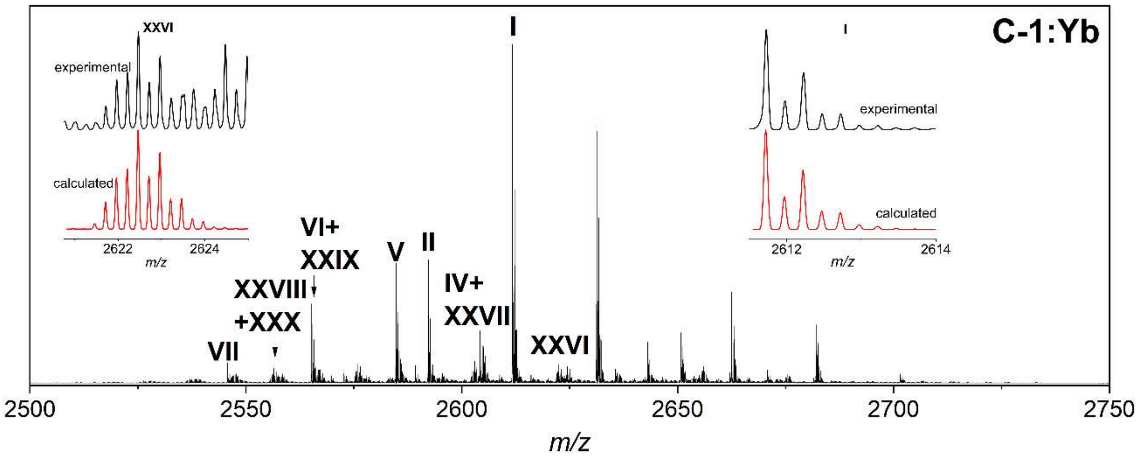

Figure S 11. Part of the ESI mass spectrum of compound **C-1:Yb** electrosprayed from MeCN/dmso showing quadruply positive charged homometallic cations (**I**–**VII**) with $[Bi_{38}O_{45}(NO_3)_{20}(dmso)_7]^{4+}$ (**I**, *m*/*z* = 2611.7214) and heterobimetallic BiO-NC cations (**XXVI**–**XXX**) with $[Bi_{37}YbO_{45}(NO_3)_{20}(dmso)_8]^{4+}$ (**XXVI**, *m*/*z* = 2622.4688) showing the highest abundance.

Table S 5. Selection of quadruply positive charged bismuth oxido nanocluster cations detected in the survey mass spectrum of BiO-NC **C-1:Yb** electrosprayed from MeCN/dmso. Assignment was carried out using the most abundant *m/z* signals.

Label	Cation	*m/z*	
		calculated	BiO-NC C-1:Yb
**I**	$[Bi_{38}O_{45}(NO_3)_{20}(dmso)_7]^{4+}$	2611.7195	2611.7258
**II**	$[Bi_{38}O_{45}(NO_3)_{20}(dmso)_6]^{4+}$	2592.2160	2592.2234
**IV**	$[Bi_{38}O_{46}(NO_3)_{18}(dmso)_8]^{4+}$	2604.2278	2604.2329
**V**	$[Bi_{38}O_{46}(NO_3)_{18}(dmso)_7]^{4+}$	2584.7243	2584.7316
**VI**	$[Bi_{38}O_{46}(NO_3)_{18}(dmso)_6]^{4+}$	2565.2208	2565.2291
**VII**	$[Bi_{38}O_{46}(NO_3)_{18}(dmso)_5]^{4+}$	2545.7174	2545.7256
**XXVI**	$[Bi_{37}YbO_{45}(NO_3)_{20}(dmso)_8]^{4+}$	2622.4626	2622.4688
**XXVII**	$[Bi_{37}YbO_{45}(NO_3)_{20}(dmso)_7]^{4+}$	2602.9589	2602.9665
**XXVIII**	$[Bi_{37}YbO_{46}(NO_3)_{18}(dmso)_6]^{4+}$	2556.4603	2556.4683
**XXIX**	$[Bi_{36}Yb_2O_{46}(NO_3)_{18}(dmso)_7]^{4+}$	2567.2034	2567.2121
**XXX**	$[Bi_{35}Yb_3O_{46}(NO_3)_{18}(dmso)_7]^{4+}$	2558.1928	2558.2037

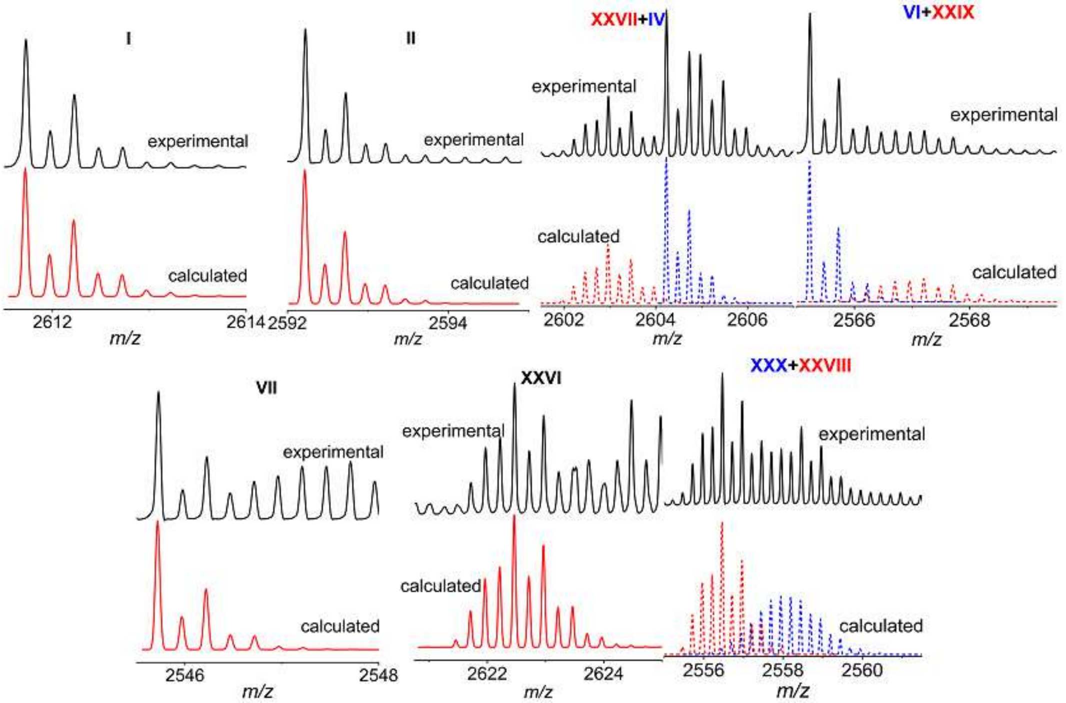

Figure S 12. Isotopic patterns (exp. and calcd.) of different quadrubly positive charged bismuth oxido nanocluster cations detected in the gas phase generated from compound **C-1:Yb** after electrospraying from MeCN/dmso. $[Bi_{38}O_{45}(NO_3)_{20}(dmso)_7]^{4+}$ (**I**), $[Bi_{38}O_{45}(NO_3)_{20}(dmso)_6]^{4+}$ (**II**), $[Bi_{38}O_{46}(NO_3)_{18}(dmso)_8]^{4+}$ (**IV**), $[Bi_{38}O_{46}(NO_3)_{18}(dmso)_7]^{4+}$ (**V**), $[Bi_{38}O_{46}(NO_3)_{18}(dmso)_6]^{4+}$ (**VI**), $[Bi_{38}O_{46}(NO_3)_{18}(dmso)_5]^{4+}$ (**VII**), $[Bi_{37}YbO_{45}(NO_3)_{20}(dmso)_8]^{4+}$ (**XXVI**), $[Bi_{37}YbO_{45}(NO_3)_{20}(dmso)_7]^{4+}$ (**XXVII**), $[Bi_{37}YbO_{46}(NO_3)_{18}(dmso)_6]^{4+}$ (**XXVIII**), $[Bi_{36}Yb_2O_{46}(NO_3)_{18}(dmso)_7]^{4+}$ (**XXIX**), $[Bi_{35}Yb_3O_{46}(NO_3)_{18}(dmso)_7]^{4+}$ (**XXX**).

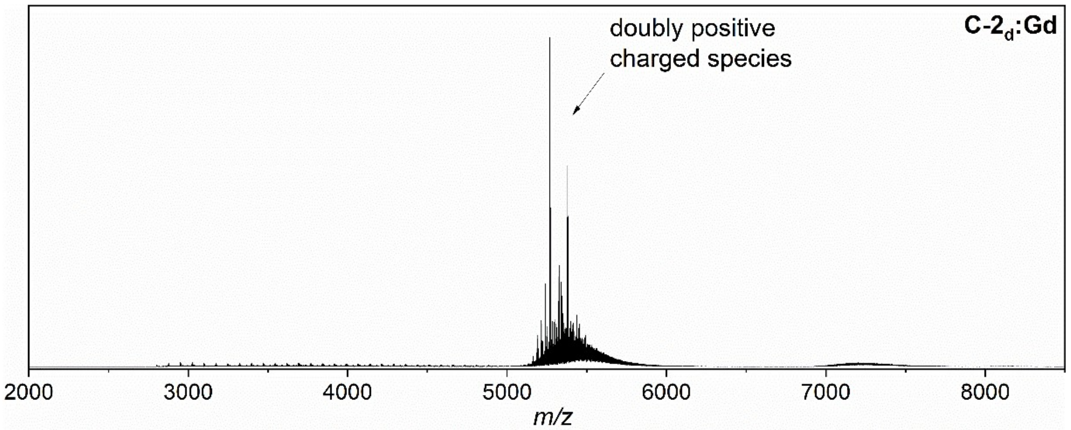

Figure S 13. Survey ESI-MS spectra generated from cluster **C-2$_d$:Gd** after electrospraying from ^{i}PrOH, with marked doubly positive charged cationic species.

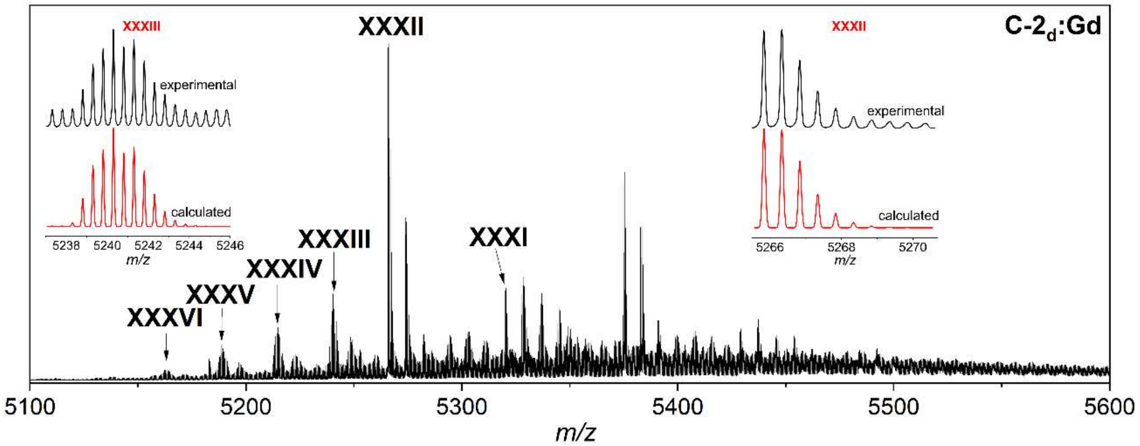

Figure S 14. Part of the ESI mass spectrum of compound **C-2$_d$:Gd** electrosprayed from ^{i}PrOH showing doubly positive charged homometallic cations (**XXXI, XXXII**) with $[Bi_{38}O_{45}(OMc)_{22}]^{2+}$ (**XXXII**, *m*/*z* = 5265.8311) and heterobimetallic BiO-NC cations (**XXXIII–XXXVI**) with $[Bi_{37}GdO_{45}(OMc)_{22}]^{4+}$ (**XXXIII**, *m*/*z* = 5240.3042) showing the highest abundance.

Table S 6. Selection of doubly positive charged bismuth oxido nanocluster cations detected in the survey mass spectrum of BiO-NC **C-2$_d$:Gd** electrosprayed from ^{i}PrOH. Assignment was carried out using the most abundant *m/z* signals.

Label	Cation	*m/z*	
		calculated	BiO-NC C-2$_d$:Gd
**XXXI**	$[Bi_{38}O_{45}(OMc)_{23}Na]^{2+}$	5320.3422	5320.3468
**XXXII**	$[Bi_{38}O_{45}(OMc)_{22}]^{2+}$	5265.8311	5265.8280
**XXXIII**	$[Bi_{37}GdO_{45}(OMc)_{22}]^{2+}$	5240.3042	5240.3022
**XXXIV**	$[Bi_{36}Gd_2O_{45}(OMc)_{22}]^{2+}$	5214.7766	5214.7759
**XXXV**	$[Bi_{35}Gd_3O_{45}(OMc)_{22}]^{2+}$	5188.2477	5188.2466
**XXXVI**	$[Bi_{34}Gd_4O_{45}(OMc)_{22}]^{2+}$	5162.7199	5162.7303

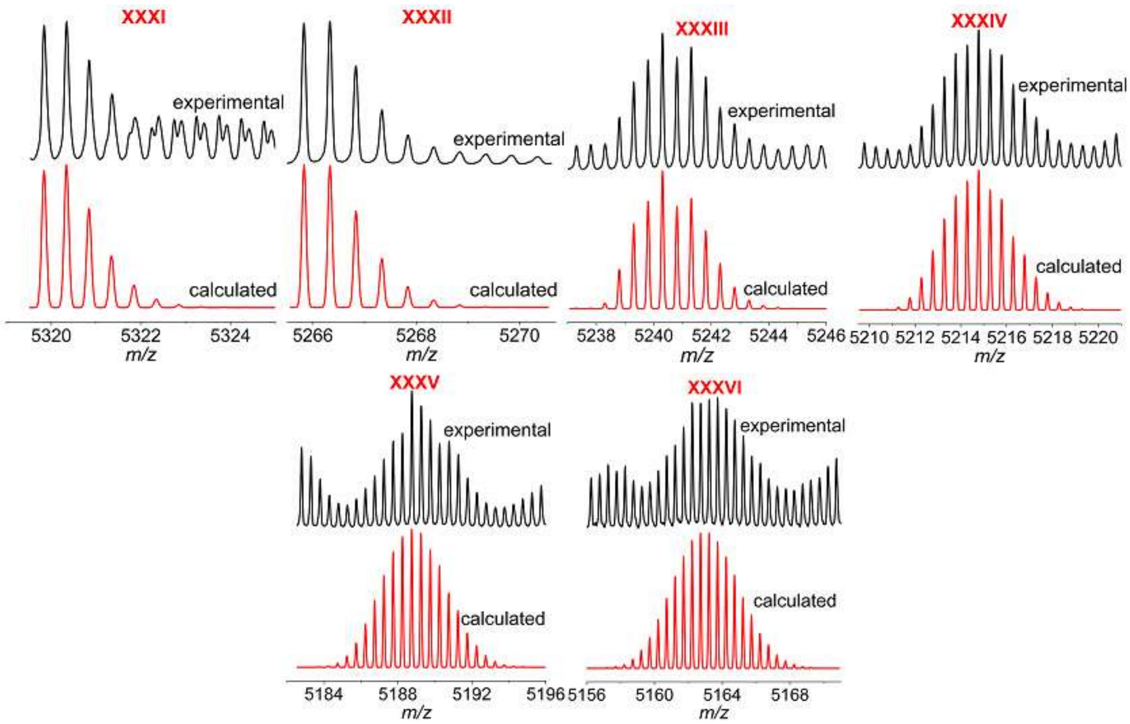

Figure S 15. Isotopic patterns (exp. and calcd.) of different doubly positive charged bismuth oxido nanocluster cations detected in the gas phase generated from compound **C-2$_d$:Gd** after electrospraying from ^{i}PrOH. $[Bi_{38}O_{45}(OMc)_{23}Na]^{2+}$ (**XXXI**), $[Bi_{38}O_{45}(OMc)_{22}]^{2+}$ (**XXXII**), $[Bi_{37}GdO_{45}(OMc)_{22}]^{2+}$ (**XXXIII**), $[Bi_{36}Gd_2O_{45}(OMc)_{22}]^{2+}$ (**XXXIV**), $[Bi_{35}Gd_3O_{45}(OMc)_{22}]^{2+}$ (**XXXV**), $[Bi_{34}Gd_4O_{45}(OMc)_{22}]^{2+}$ (**XXXVI**).

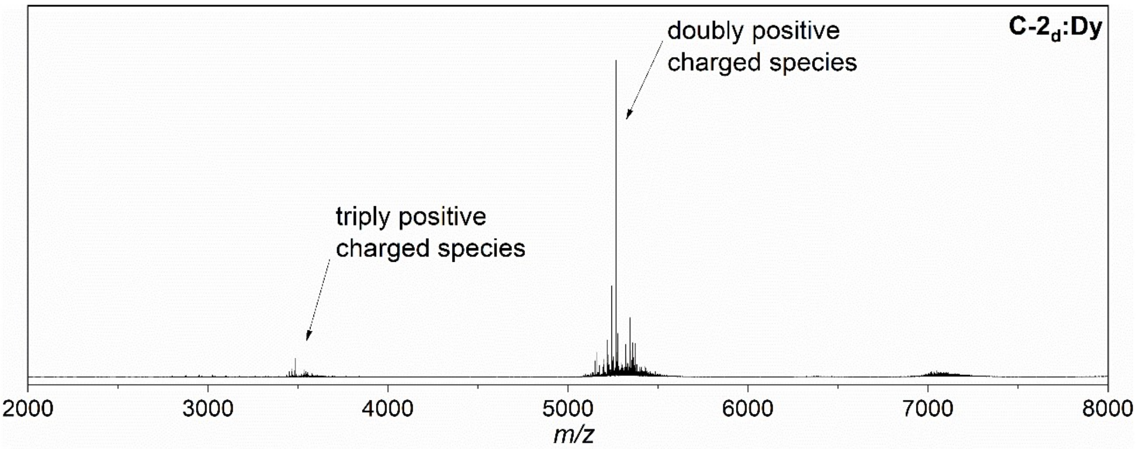

Figure S 16. Survey ESI-MS spectra generated from cluster **C-2$_d$:Dy** after electrospraying from ^{i}PrOH, with marked doubly and triply positive charged cationic species.

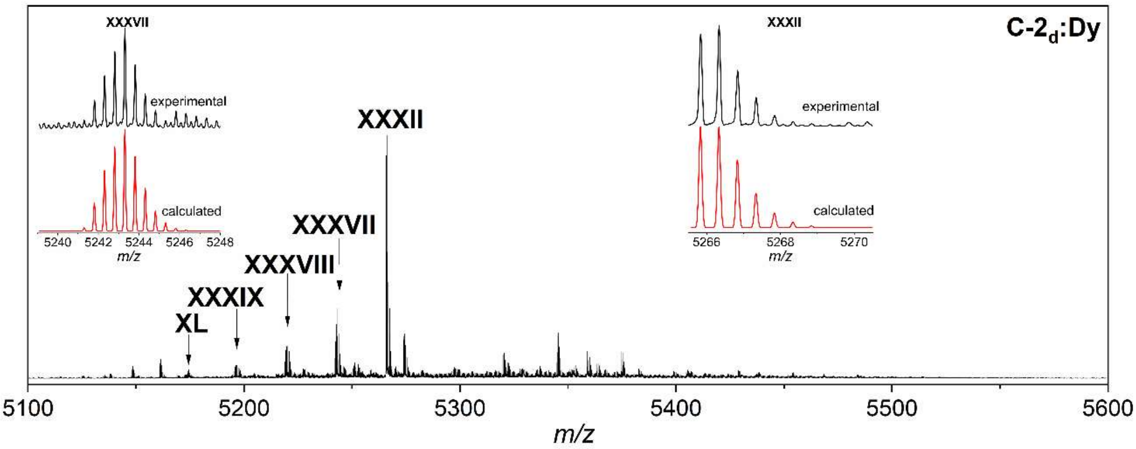

Figure S 17. Part of the ESI mass spectrum of compound **C-2$_d$:Dy** electrosprayed from ^{i}PrOH showing doubly positive charged homometallic cation with $[Bi_{38}O_{45}(OMc)_{22}]^{2+}$ (**XXXII**, *m*/*z* = 5265.8311) and heterobimetallic BiO-NC cations (**XXXVII**–**XL**) with $[Bi_{37}DyO_{45}(OMc)_{22}]^{2+}$ (**XXXVII**, *m*/*z* = 5243.3067) showing the highest abundance.

Table S 7. Selection of doubly positive charged bismuth oxido nanocluster cations detected in the survey mass spectrum of BiO-NC **C-2$_d$:Dy** electrosprayed from ^{i}PrOH. Assignment was carried out using the most abundant *m/z* signals.

Label	Cation	*m/z*	
		**calculated**	**BiO-NC C-2$_d$:Dy**
**XXXII**	$[Bi_{38}O_{45}(OMc)_{22}]^{2+}$	5265.8311	5265.8372
**XXXVII**	$[Bi_{37}DyO_{45}(OMc)_{22}]^{2+}$	5243.3067	5243.3096
**XXXVIII**	$[Bi_{36}Dy_2O_{45}(OMc)_{22}]^{2+}$	5219.7802	5219.7839
**XXXIX**	$[Bi_{35}Dy_3O_{45}(OMc)_{22}]^{2+}$	5196.7543	5196.7614
**XL**	$[Bi_{34}Dy_4O_{45}(OMc)_{22}]^{2+}$	5173.7284	5173.7264

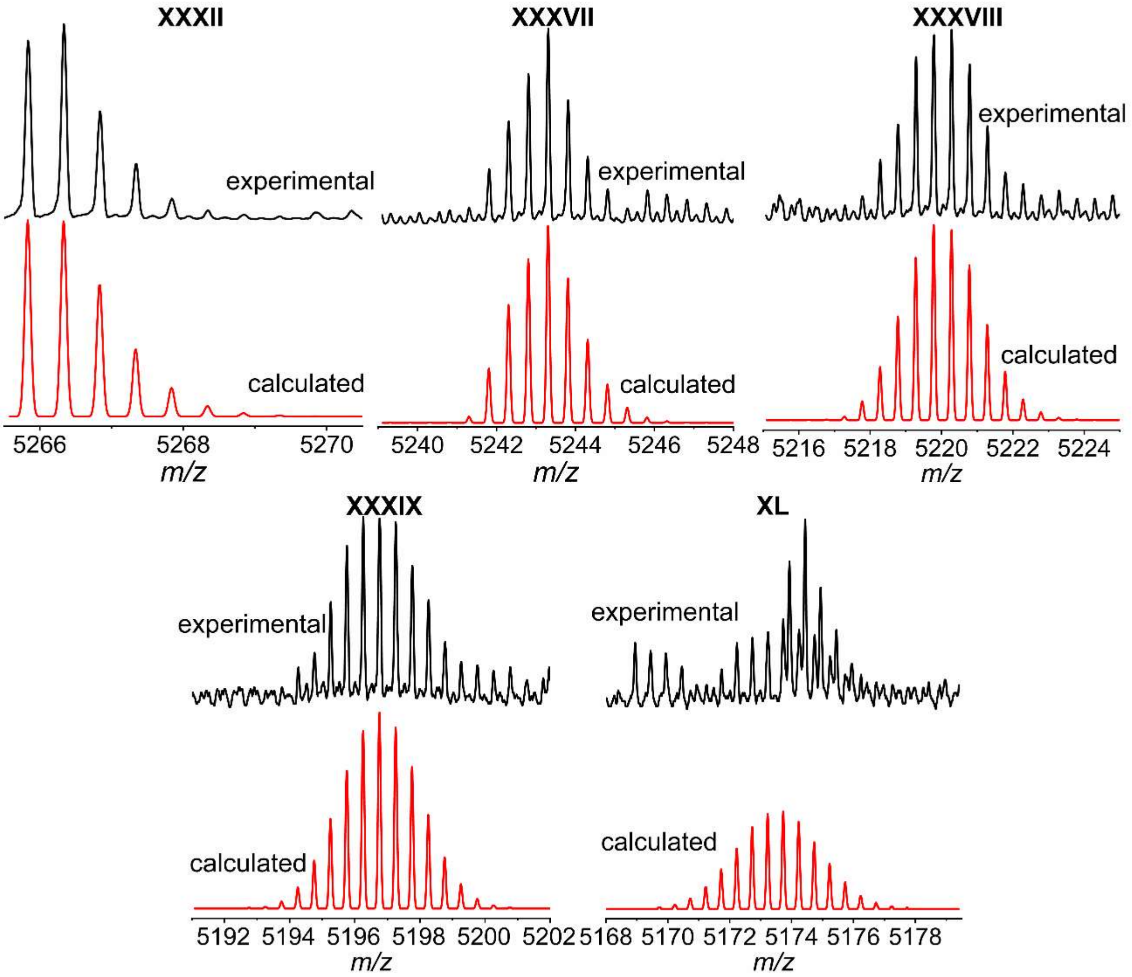

Figure S 18. Isotopic patterns (exp. and calcd.) of different doubly positive charged bismuth oxido nanocluster cations detected in the gas phase generated from compound **C-2$_d$:Dy** after electrospraying from ^{i}PrOH. $[Bi_{38}O_{45}(OMc)_{22}]^{2+}$ (**XXXI**), $[Bi_{37}DyO_{45}(OMc)_{22}]^{2+}$ (**XXXVII**), $[Bi_{36}Dy_2O_{45}(OMc)_{22}]^{2+}$ (**XXXVIII**), $[Bi_{35}Dy_3O_{45}(OMc)_{22}]^{2+}$ (**XXXIX**), $[Bi_{34}Dy_4O_{45}(OMc)_{22}]^{2+}$ (**XXXX**). Please note, that an additional intensive species is detected in the pattern of XXXX, which we could not assign, however its pattern indicates an undoped BiO-NC species.

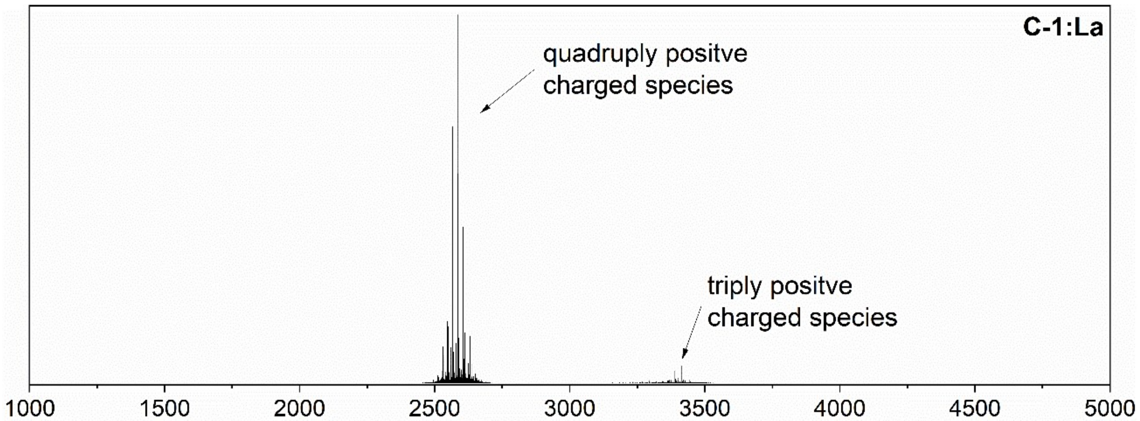

Figure S 19. Survey ESI-MS spectra generated from cluster **C-1:La** after electrospraying from MeCN/dmso, with marked triply and quadruply positive charged cationic species.

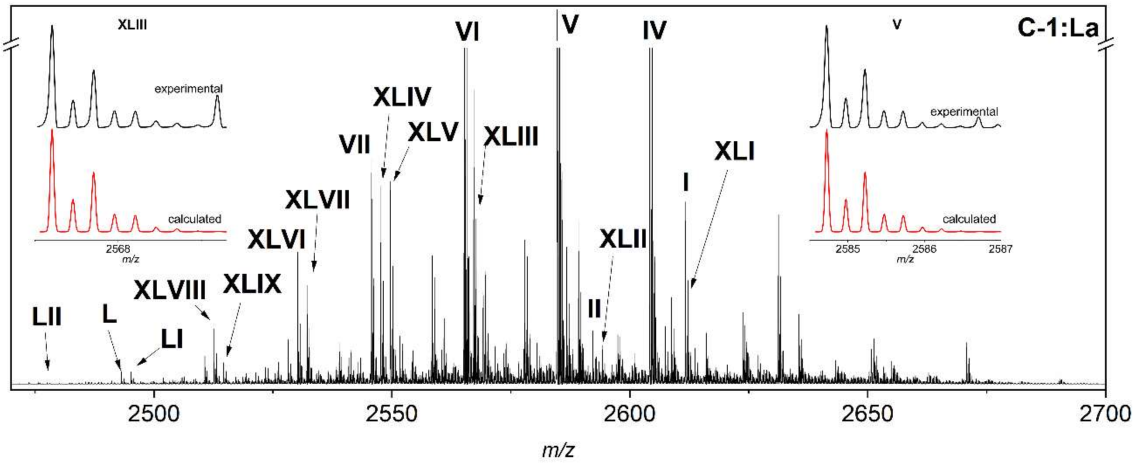

Figure S 20. Part of the ESI mass spectrum of compound **C-1:La** electrosprayed from MeCN/dmso showing quadruply positive charged homometallic cations (**I**–**VII**) with $[Bi_{38}O_{45}(NO_3)_{18}(dmso)_7]^{4+}$ (**V**, *m*/*z* = 2584.7212) and heterobimetallic BiO-NC cations (**XLI**–**LII**) with $[Bi_{37}LaO_{46}(NO_3)_{18}(dmso)_7]^{4+}$ (**XLIII**, *m*/*z* = 2601.4661) showing the highest abundance.

Table S 8. Selection of quadruply positive charged bismuth oxido nanocluster cations detected in the survey mass spectrum of BiO-NC **C-1:La** electrosprayed from MeCN/dmso. Assignment was carried out using the most abundant *m/z* signals.

Label	Cation	*m/z*	
		calculated	BiO-NC C-1:La
**I**	$[Bi_{38}O_{45}(NO_3)_{20}(dmso)_7]^{4+}$	2611.7195	2611.7108
**II**	$[Bi_{38}O_{45}(NO_3)_{20}(dmso)_6]^{4+}$	2592.2160	2592.2115
**IV**	$[Bi_{38}O_{46}(NO_3)_{18}(dmso)_8]^{4+}$	2604.2278	2604.2204
**V**	$[Bi_{38}O_{46}(NO_3)_{18}(dmso)_7]^{4+}$	2584.7243	2584.7212
**VI**	$[Bi_{38}O_{46}(NO_3)_{18}(dmso)_6]^{4+}$	2565.2208	2565.2199
**VII**	$[Bi_{38}O_{46}(NO_3)_{18}(dmso)_5]^{4+}$	2545.7174	2545.7172
**XLI**	$[Bi_{37}LaO_{45}(NO_3)_{20}(dmso)_8]^{4+}$	2613.7045	2613.6999
**XLII**	$[Bi_{37}LaO_{45}(NO_3)_{20}(dmso)_7]^{4+}$	2594.2010	2594.2007
**XLIII**	$[Bi_{37}LaO_{46}(NO_3)_{18}(dmso)_7]^{4+}$	2567.2058	2567.2047
**XLIV**	$[Bi_{37}LaO_{46}(NO_3)_{18}(dmso)_6]^{4+}$	2547.7023	2547.7027
**XLV**	$[Bi_{36}La_2O_{46}(NO_3)_{18}(dmso)_7]^{4+}$	2549.6873	2549.6878
**XLVI**	$[Bi_{36}La_2O_{46}(NO_3)_{18}(dmso)_6]^{4+}$	2530.1838	2530.1849
**XLVII**	$[Bi_{35}La_3O_{46}(NO_3)_{18}(dmso)_7]^{4+}$	2532.1688	2532.1704
**XLVIII**	$[Bi_{35}La_3O_{46}(NO_3)_{18}(dmso)_6]^{4+}$	2512.6653	2512.6667
**XLIX**	$[Bi_{34}La_4O_{46}(NO_3)_{18}(dmso)_7]^{4+}$	2514.6503	2514.6530
**L**	$[Bi_{34}La_4O_{46}(NO_3)_{18}(dmso)_6]^{4+}$	2495.1468	2495.1486
**LI**	$[Bi_{33}La_5O_{46}(NO_3)_{18}(dmso)_7]^{4+}$	2497.1318	2497.1375
**LII**	$[Bi_{33}La_5O_{46}(NO_3)_{18}(dmso)_6]^{4+}$	2477.6283	2477.6299

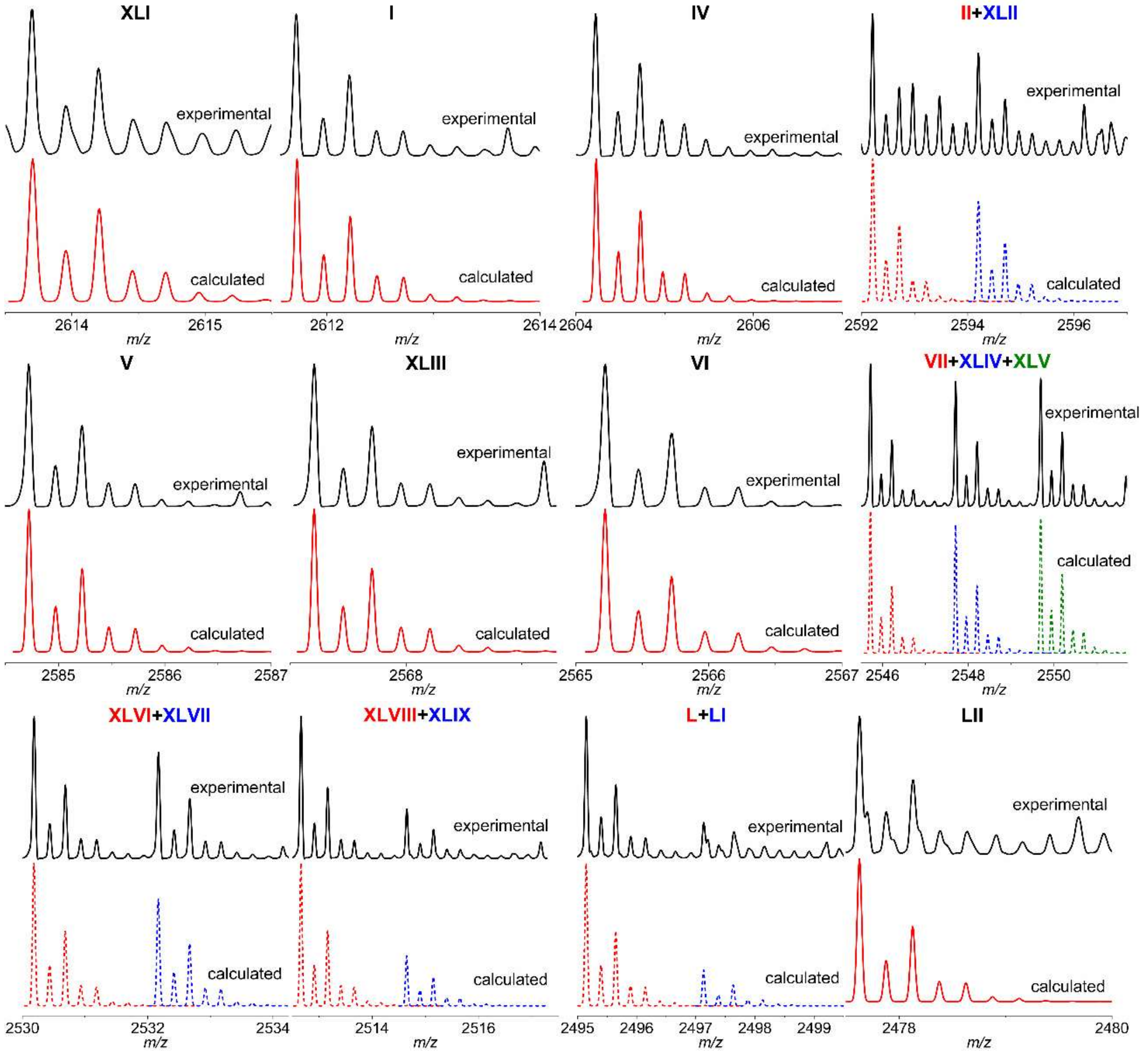

Figure S 21. Isotopic patterns (exp. and calcd.) of different quadruply positive charged bismuth oxido nanocluster cations detected in the gas phase generated from compound **C-1:La** after electrospraying from MeCN/dmso. $[Bi_{38}O_{45}(NO_3)_{20}(dmso)_7]^{4+}$ (**I**), $[Bi_{38}O_{45}(NO_3)_{20}(dmso)_6]^{4+}$ (**II**), $[Bi_{38}O_{45}(NO_3)_{19}(OH)(dmso)_8]^{4+}$ (**III**), $[Bi_{38}O_{46}(NO_3)_{18}(dmso)_7]^{4+}$ (**V**), $[Bi_{38}O_{46}(NO_3)_{18}(dmso)_6]^{4+}$ (**VI**), $[Bi_{38}O_{46}(NO_3)_{18}(dmso)_5]^{4+}$ (**VII**), $[Bi_{37}LaO_{45}(NO_3)_{20}(dmso)_8]^{4+}$ (**XLI**), $[Bi_{37}LaO_{45}(NO_3)_{20}(dmso)_7]^{4+}$ (**XLII**), $[Bi_{37}LaO_{46}(NO_3)_{18}(dmso)_7]^{4+}$ (**XLIII**), $[Bi_{37}LaO_{46}(NO_3)_{18}(dmso)_6]^{4+}$ (**XLIV**), $[Bi_{36}La_2O_{46}(NO_3)_{18}(dmso)_7]^{4+}$ (**XLV**), $[Bi_{36}La_2O_{46}(NO_3)_{18}(dmso)_6]^{4+}$ (**XLVI**), $[Bi_{35}La_3O_{46}(NO_3)_{18}(dmso)_7]^{4+}$ (**XLVII**), $[Bi_{35}La_3O_{46}(NO_3)_{18}(dmso)_6]^{4+}$ (**XLVIII**), $[Bi_{34}La_4O_{46}(NO_3)_{18}(dmso)_7]^{4+}$ (**XLIX**), $[Bi_{34}La_4O_{46}(NO_3)_{18}(dmso)_6]^{4+}$ (**L**), $[Bi_{33}La_5O_{46}(NO_3)_{18}(dmso)_7]^{4+}$ (**LI**), $[Bi_{33}La_5O_{46}(NO_3)_{18}(dmso)_6]^{4+}$ (**LII**).

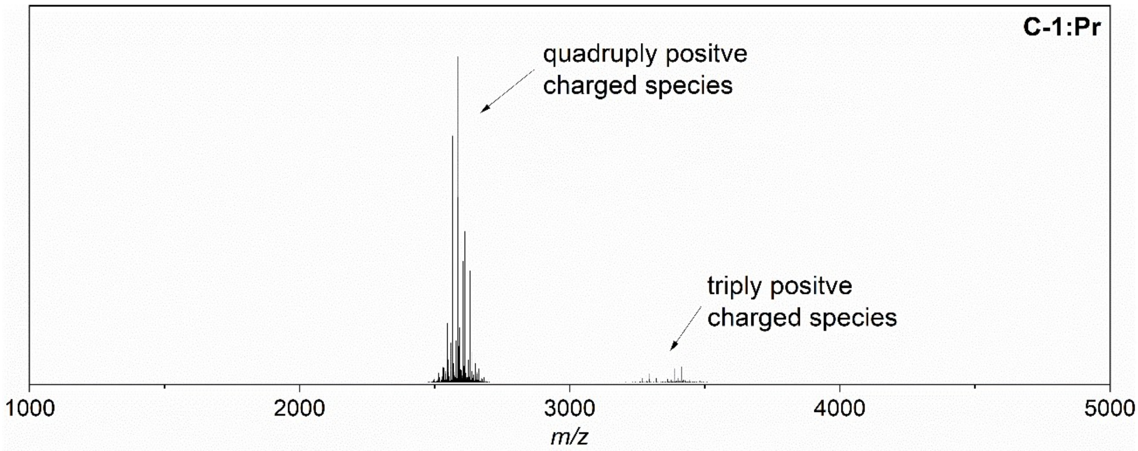

Figure S 22. Survey ESI-MS spectra generated from cluster **C-1:Pr** after electrospraying from MeCN/dmso, with marked triply and quadruply positive charged cationic species.

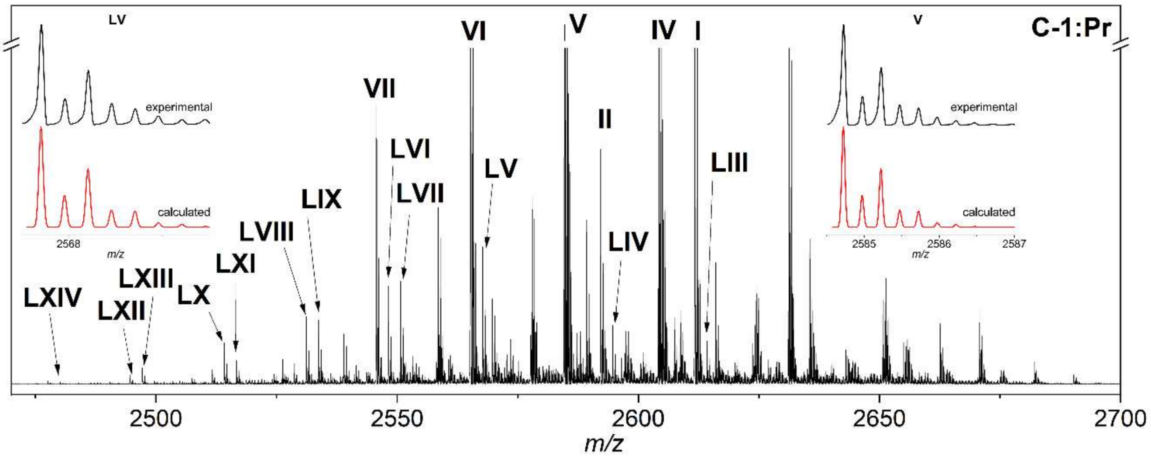

Figure S 23. Part of the ESI mass spectrum of compound **C-1:Pr** electrosprayed from MeCN/dmso showing quadruply positive charged homometallic cations (**I**–**VII**) with $[Bi_{38}O_{45}(NO_3)_{18}(dmso)_7]^{4+}$ (**V**, *m*/*z* = 2584.7259) and heterobimetallic BiO-NC cations (**LIII**–**LXIV**) with $[Bi_{37}PrO_{46}(NO_3)_{18}(dmso)_7]^{4+}$ (**LV**, *m*/*z* = 2567.7084) showing the highest abundance.

Table S 9. Selection of quadruply positive charged bismuth oxido nanocluster cations detected in the survey mass spectrum of BiO-NC **C-1:Pr** electrosprayed from MeCN/dmso. Assignment was carried out using the most abundant *m/z* signals.

Label	Cation	*m/z*	
		**calculated**	**BiO-NC C-1:Pr**
**I**	$[Bi_{38}O_{45}(NO_3)_{20}(dmso)_7]^{4+}$	2611.7195	2611.7178
**II**	$[Bi_{38}O_{45}(NO_3)_{20}(dmso)_6]^{4+}$	2592.2160	2592.2167
**IV**	$[Bi_{38}O_{46}(NO_3)_{18}(dmso)_8]^{4+}$	2604.2278	2604.2273
**V**	$[Bi_{38}O_{46}(NO_3)_{18}(dmso)_7]^{4+}$	2584.7243	2584.7259
**VI**	$[Bi_{38}O_{46}(NO_3)_{18}(dmso)_6]^{4+}$	2565.2208	2565.2230
**VII**	$[Bi_{38}O_{46}(NO_3)_{18}(dmso)_5]^{4+}$	2545.7174	2545.7193
**LIII**	$[Bi_{37}PrO_{45}(NO_3)_{20}(dmso)_8]^{4+}$	2614.2048	2614.2055
**LIV**	$[Bi_{37}PrO_{45}(NO_3)_{20}(dmso)_7]^{4+}$	2594.7013	2594.7041
**LV**	$[Bi_{37}PrO_{46}(NO_3)_{18}(dmso)_7]^{4+}$	2567.7061	2567.7084
**LVI**	$[Bi_{37}PrO_{46}(NO_3)_{18}(dmso)_6]^{4+}$	2548.2027	2548.2051
**LVII**	$[Bi_{36}Pr_2O_{46}(NO_3)_{18}(dmso)_7]^{4+}$	2550.6880	2550.6906
**LVIII**	$[Bi_{36}Pr_2O_{46}(NO_3)_{18}(dmso)_6]^{4+}$	2531.1845	2531.1865
**LIX**	$[Bi_{35}Pr_3O_{46}(NO_3)_{18}(dmso)_7]^{4+}$	2533.6698	2533.6721
**LX**	$[Bi_{35}Pr_3O_{46}(NO_3)_{18}(dmso)_6]^{4+}$	2514.1663	2514.1681
**LXI**	$[Bi_{34}Pr_4O_{46}(NO_3)_{18}(dmso)_7]^{4+}$	2516.6516	2516.6537
**LXII**	$[Bi_{34}Pr_4O_{46}(NO_3)_{18}(dmso)_6]^{4+}$	2497.1481	2497.1497
**LXIII**	$[Bi_{33}Pr_5O_{46}(NO_3)_{18}(dmso)_7]^{4+}$	2499.6334	2499.6361
**LXIV**	$[Bi_{33}Pr_5O_{46}(NO_3)_{18}(dmso)_6]^{4+}$	2480.1299	2480.1309

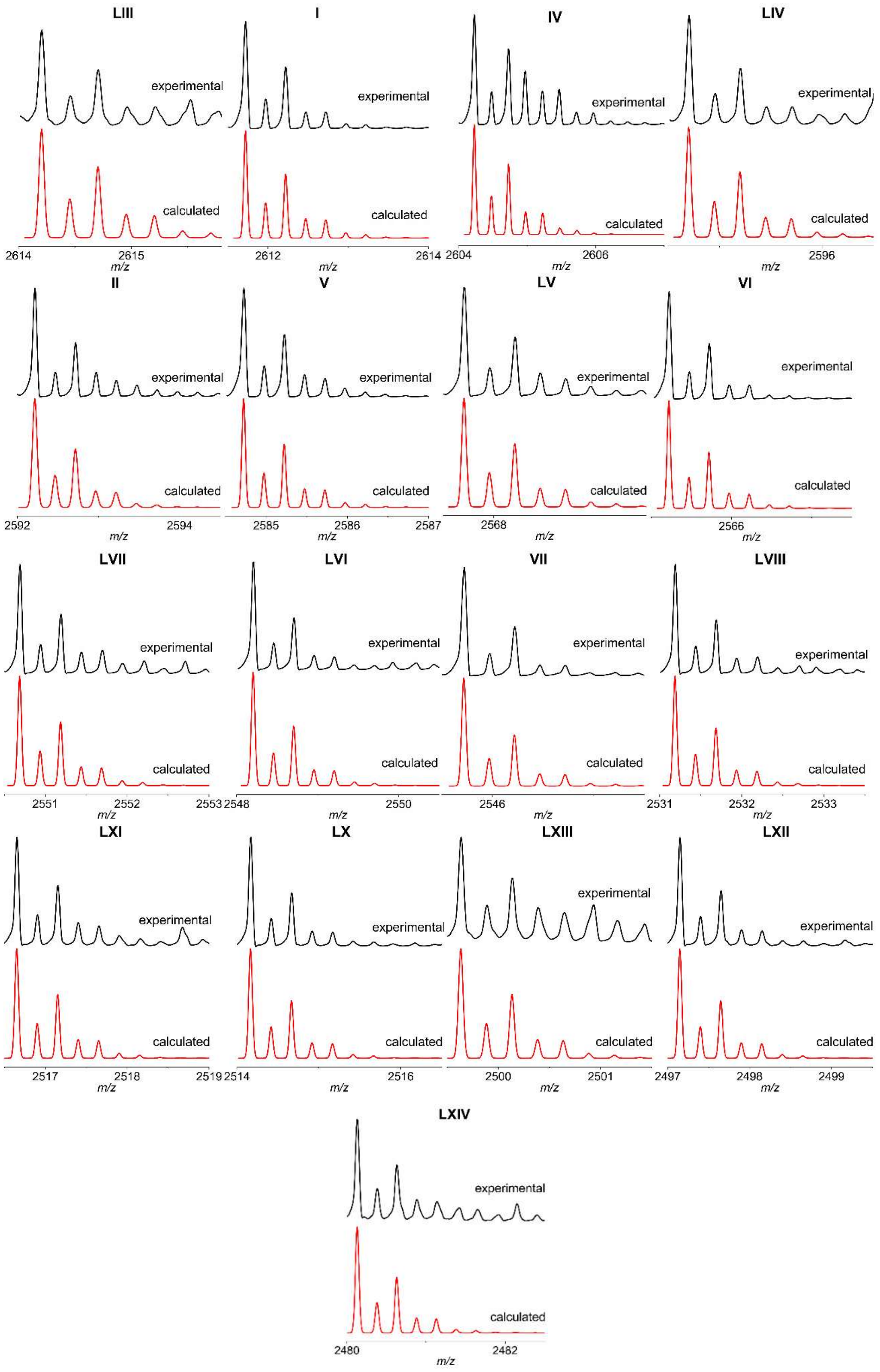

Figure S 24. Isotopic patterns (exp. and calcd.) of different quadruply positive charged bismuth oxido nanocluster cations detected in the gas phase generated from compound **C-1:Pr** after electrospraying from MeCN/dmso. $[Bi_{38}O_{45}(NO_3)_{20}(dmso)_7]^{4+}$ (**I**), $[Bi_{38}O_{45}(NO_3)_{20}(dmso)_6]^{4+}$ (**II**), $[Bi_{38}O_{46}(NO_3)_{18}(dmso)_7]^{4+}$ (**V**), $[Bi_{38}O_{46}(NO_3)_{18}(dmso)_6]^{4+}$ (**VI**), $[Bi_{38}O_{46}(NO_3)_{18}(dmso)_5]^{4+}$ (**VII**), $[Bi_{37}PrO_{45}(NO_3)_{20}(dmso)_8]^{4+}$ (**LIII**), $[Bi_{37}PrO_{45}(NO_3)_{20}(dmso)_7]^{4+}$ (**LIV**), $[Bi_{37}PrO_{46}(NO_3)_{18}(dmso)_7]^{4+}$ (**LV**), $[Bi_{37}PrO_{46}(NO_3)_{18}(dmso)_6]^{4+}$ (**LVI**), $[Bi_{36}Pr_2O_{46}(NO_3)_{18}(dmso)_7]^{4+}$ (**LVII**), $[Bi_{36}Pr_2O_{46}(NO_3)_{18}(dmso)_6]^{4+}$ (**LVIII**), $[Bi_{35}Pr_3O_{46}(NO_3)_{18}(dmso)_7]^{4+}$ (**LIX**), $[Bi_{35}Pr_3O_{46}(NO_3)_{18}(dmso)_6]^{4+}$ (**LX**), $[Bi_{34}Pr_4O_{46}(NO_3)_{18}(dmso)_7]^{4+}$ (**LXI**), $[Bi_{34}Pr_4O_{46}(NO_3)_{18}(dmso)_6]^{4+}$ (**LXII**), $[Bi_{33}Pr_5O_{46}(NO_3)_{18}(dmso)_7]^{4+}$ (**LXIII**), $[Bi_{33}Pr_5O_{46}(NO_3)_{18}(dmso)_6]^{4+}$ (**LXIV**).

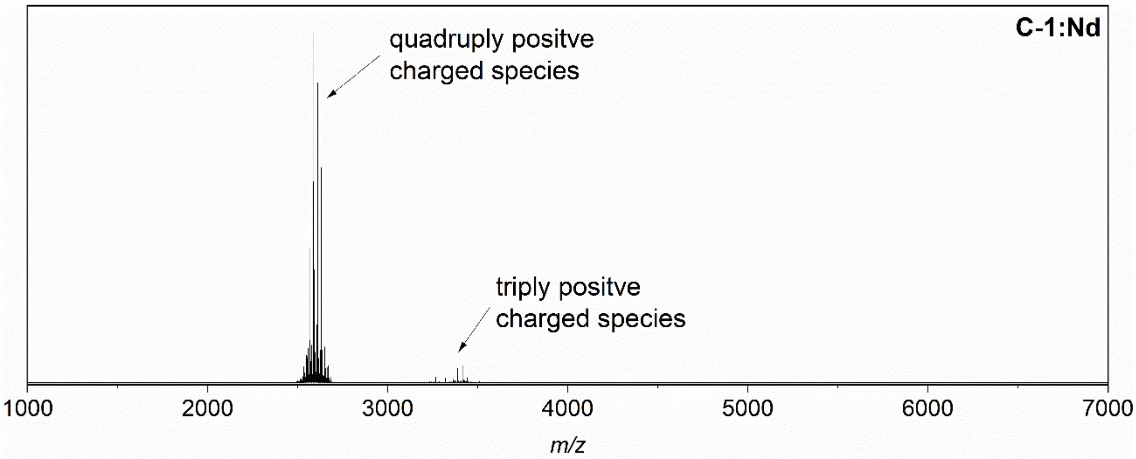

Figure S 25. Survey ESI-MS spectra generated from cluster **C-1:Nd** after electrospraying from MeCN/dmso, with marked triply and quadruply positive charged cationic species.

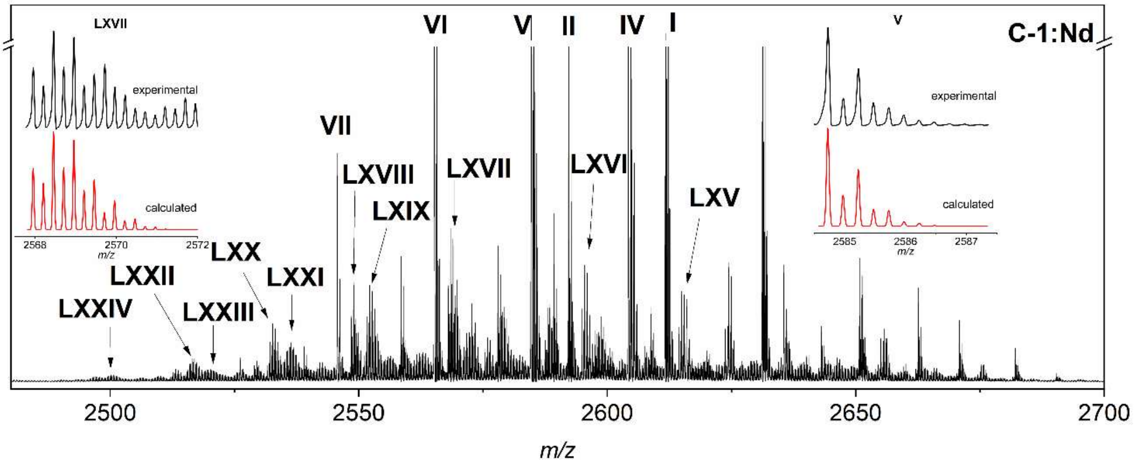

Figure S 26. Part of the ESI mass spectrum of compound **C-1:Nd** electrosprayed from MeCN/dmso showing quadruply positive charged homometallic cations (**I**–**VII**) with $[Bi_{38}O_{45}(NO_3)_{18}(dmso)_7]^{4+}$ (**V**, *m*/*z* = 2584.7249) and heterobimetallic BiO-NC cations (**LXV**–**LXXIV**) with $[Bi_{37}NdO_{46}(NO_3)_{18}(dmso)_7]^{4+}$ (**LXVII**, *m*/*z* = 2568.4577) showing the highest abundance.

Table S 10. Selection of quadruply positive charged bismuth oxido nanocluster cations detected in the survey mass spectrum of BiO-NC **C-1:Nd** electrosprayed from MeCN/dmso. Assignment was carried out using the most abundant *m/z* signals.

Label	Cation	***m/z***	
		**calculated**	**BiO-NC C-1:Nd**
**I**	$[Bi_{38}O_{45}(NO_3)_{20}(dmso)_7]^{4+}$	2611.7195	2611.7180
**II**	$[Bi_{38}O_{45}(NO_3)_{20}(dmso)_6]^{4+}$	2592.2160	2592.2156
**IV**	$[Bi_{38}O_{46}(NO_3)_{18}(dmso)_8]^{4+}$	2604.2278	2604.2271
**V**	$[Bi_{38}O_{46}(NO_3)_{18}(dmso)_7]^{4+}$	2584.7243	2584.7249
**VI**	$[Bi_{38}O_{46}(NO_3)_{18}(dmso)_6]^{4+}$	2565.2208	2565.2220
**VII**	$[Bi_{38}O_{46}(NO_3)_{18}(dmso)_5]^{4+}$	2545.7174	2545.7174
**LXV**	$[Bi_{37}NdO_{45}(NO_3)_{20}(dmso)_8]^{4+}$	2614.9552	2614.9560
**LXVI**	$[Bi_{37}NdO_{45}(NO_3)_{20}(dmso)_7]^{4+}$	2595.4517	2595.4531
**LXVII**	$[Bi_{37}NdO_{46}(NO_3)_{18}(dmso)_7]^{4+}$	2568.4565	2568.4577
**LXVIII**	$[Bi_{37}NdO_{46}(NO_3)_{18}(dmso)_6]^{4+}$	2548.9531	2548.9536
**LXIX**	$[Bi_{36}Nd_2O_{46}(NO_3)_{18}(dmso)_7]^{4+}$	2552.1891	2552.1915
**LXX**	$[Bi_{36}Nd_2O_{46}(NO_3)_{18}(dmso)_6]^{4+}$	2532.6856	2532.6872
**LXXI**	$[Bi_{35}Nd_3O_{46}(NO_3)_{18}(dmso)_7]^{4+}$	2536.4222	2536.4241
**LXXII**	$[Bi_{35}Nd_3O_{46}(NO_3)_{18}(dmso)_6]^{4+}$	2516.9188	2516.9198
**LXXIII**	$[Bi_{34}Nd_4O_{46}(NO_3)_{18}(dmso)_7]^{4+}$	2520.1548	2520.1578
**LXXIV**	$[Bi_{34}Nd_4O_{46}(NO_3)_{18}(dmso)_6]^{4+}$	2500.6514	2500.6530

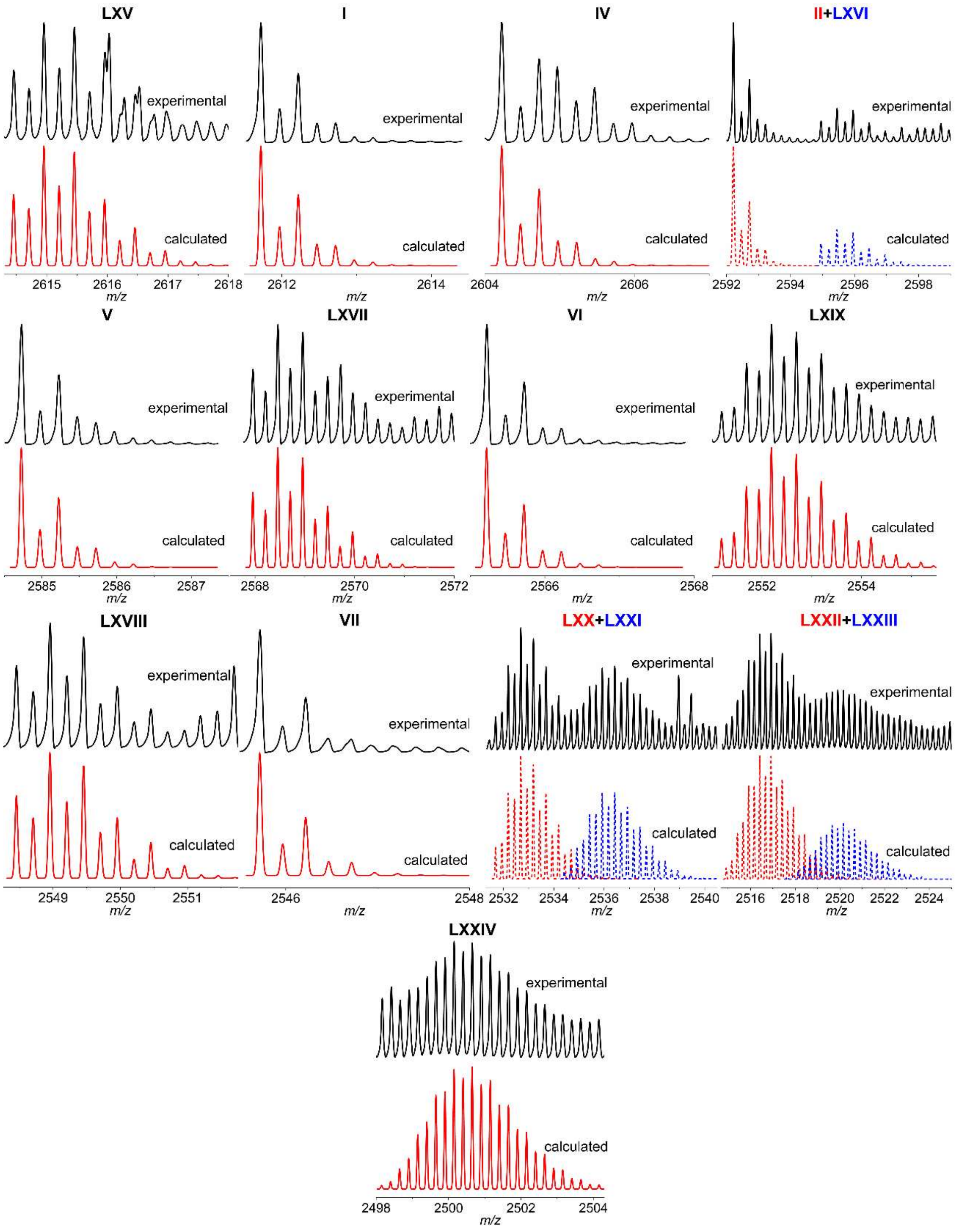

Figure S 27. Isotopic patterns (exp. and calcd.) of different quadruply positive charged bismuth oxido nanocluster cations detected in the gas phase generated from compound **C-1:Nd** after electrospraying from MeCN/dmso. $[Bi_{38}O_{45}(NO_3)_{20}(dmso)_7]^{4+}$ (**I**), $[Bi_{38}O_{45}(NO_3)_{20}(dmso)_6]^{4+}$ (**II**), $[Bi_{38}O_{46}(NO_3)_{18}(dmso)_7]^{4+}$ (**V**), $[Bi_{38}O_{46}(NO_3)_{18}(dmso)_6]^{4+}$ (**VI**), $[Bi_{38}O_{46}(NO_3)_{18}(dmso)_5]^{4+}$ (**VII**), $[Bi_{37}NdO_{45}(NO_3)_{20}(dmso)_8]^{4+}$ (**LXV**), $[Bi_{37}NdO_{45}(NO_3)_{20}(dmso)_7]^{4+}$ (**LXVI**), $[Bi_{37}NdO_{46}(NO_3)_{18}(dmso)_7]^{4+}$ (**LXVII**), $[Bi_{37}NdO_{46}(NO_3)_{18}(dmso)_6]^{4+}$ (**LXVIII**), $[Bi_{36}Nd_2O_{46}(NO_3)_{18}(dmso)_7]^{4+}$ (**LXVIX**), $[Bi_{36}Nd_2O_{46}(NO_3)_{18}(dmso)_6]^{4+}$ (**LXX**), $[Bi_{35}Nd_3O_{46}(NO_3)_{18}(dmso)_7]^{4+}$ (**LXXI**), $[Bi_{35}Nd_3O_{46}(NO_3)_{18}(dmso)_6]^{4+}$ (**LXXII**), $[Bi_{34}Nd_4O_{46}(NO_3)_{18}(dmso)_7]^{4+}$ (**LXXIII**), $[Bi_{34}Nd_4O_{46}(NO_3)_{18}(dmso)_6]^{4+}$ (**LXXIV**).

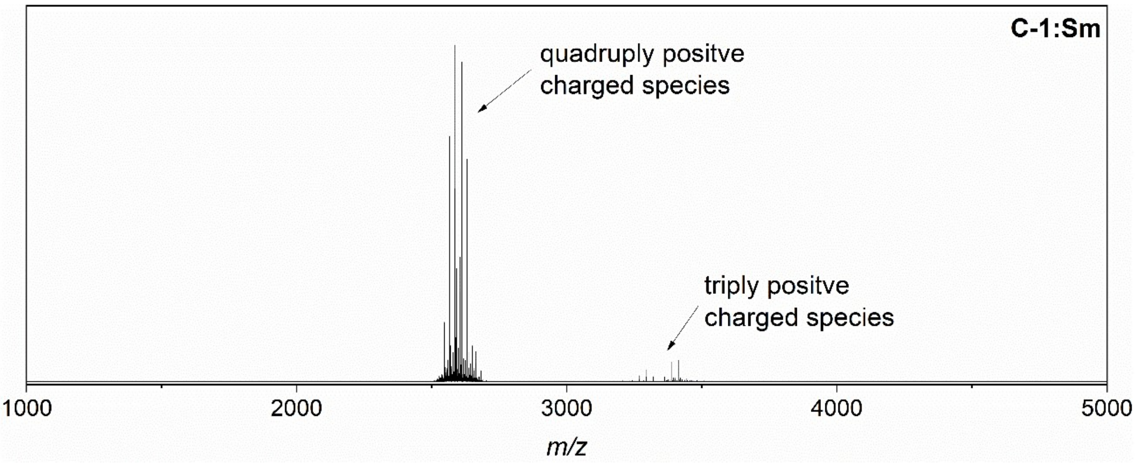

Figure S 28. Survey ESI-MS spectra generated from cluster **C-1:Sm** after electrospraying from MeCN/dmso, with marked triply and quadruply positive charged cationic species.

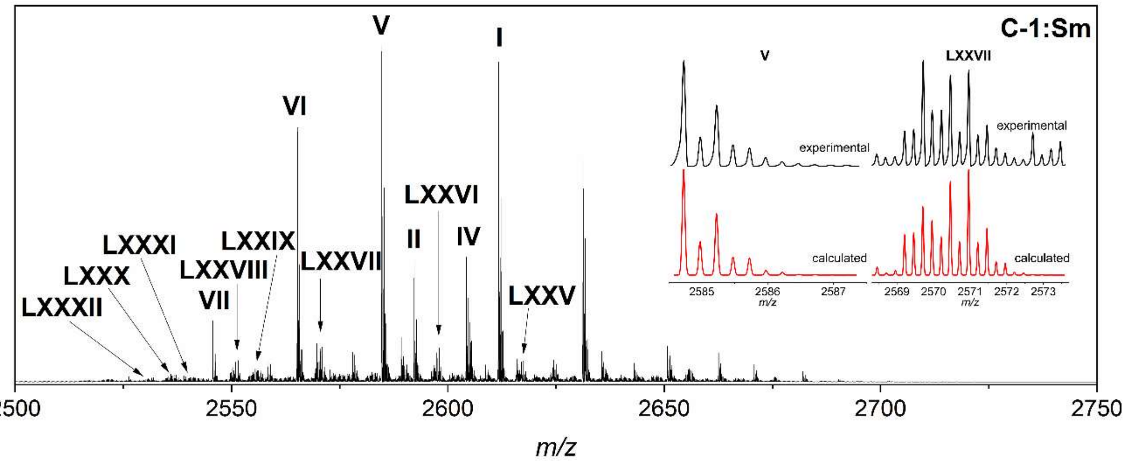

Figure S 29. Part of the ESI mass spectrum of compound **C-1:Sm** electrosprayed from MeCN/dmso showing quadruply positive charged homometallic cations (**I**–**VII**) with $[Bi_{38}O_{45}(NO_3)_{18}(dmso)_7]^{4+}$ (**V**, *m*/*z* = 2584.7246) and heterobimetallic BiO-NC cations (**LXXV**–**LXXXII**) with $[Bi_{37}SmO_{46}(NO_3)_{18}(dmso)_7]^{4+}$ (**LXXVII**, *m*/*z* = 2570.9604) showing the highest abundance.

Table S 11. Selection of quadruply positive charged bismuth oxido nanocluster cations detected in the survey mass spectrum of BiO-NC **C-1:Sm** electrosprayed from MeCN/dmso. Assignment was carried out using the most abundant *m/z* signals.

Label	Cation	*m/z*	
		calculated	BiO-NC C-1:Sm
**I**	$[Bi_{38}O_{45}(NO_3)_{20}(dmso)_7]^{4+}$	2611.7195	2611.7178
**II**	$[Bi_{38}O_{45}(NO_3)_{20}(dmso)_6]^{4+}$	2592.2160	2592.2157
**IV**	$[Bi_{38}O_{46}(NO_3)_{18}(dmso)_8]^{4+}$	2604.2278	2604.2265
**V**	$[Bi_{38}O_{46}(NO_3)_{18}(dmso)_7]^{4+}$	2584.7243	2584.7246
**VI**	$[Bi_{38}O_{46}(NO_3)_{18}(dmso)_6]^{4+}$	2565.2208	2565.2217
**VII**	$[Bi_{38}O_{46}(NO_3)_{18}(dmso)_5]^{4+}$	2545.7174	2545.7179
**LXXV**	$[Bi_{37}SmO_{45}(NO_3)_{20}(dmso)_8]^{4+}$	2617.4580	2617.4591
**LXXVI**	$[Bi_{37}SmO_{45}(NO_3)_{20}(dmso)_7]^{4+}$	2597.9546	2597.9576
**LXXVII**	$[Bi_{37}SmO_{46}(NO_3)_{18}(dmso)_7]^{4+}$	2570.9594	2570.9604
**LXXVIII**	$[Bi_{37}SmO_{46}(NO_3)_{18}(dmso)_6]^{4+}$	2551.4560	2551.4567
**LXXIX**	$[Bi_{36}Sm_2O_{46}(NO_3)_{18}(dmso)_7]^{4+}$	2555.4431	2555.4452
**LXXX**	$[Bi_{36}Sm_2O_{46}(NO_3)_{18}(dmso)_6]^{4+}$	2535.9397	2535.9401
**LXXXI**	$[Bi_{35}Sm_3O_{46}(NO_3)_{18}(dmso)_7]^{4+}$	2541.1779	2541.1797
**LXXXII**	$[Bi_{35}Sm_3O_{46}(NO_3)_{18}(dmso)_6]^{4+}$	2521.6745	2521.6748

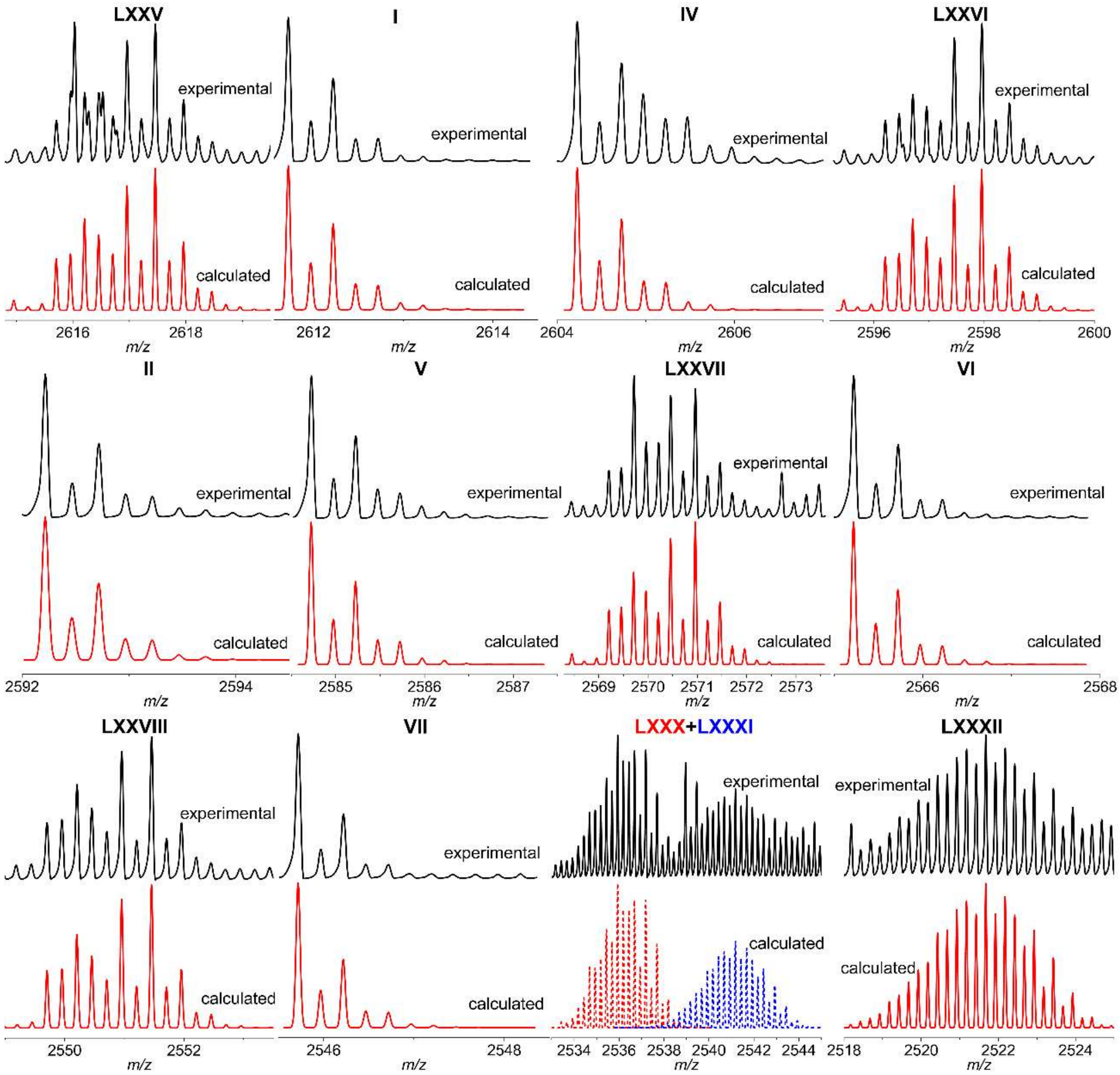

Figure S 30. Isotopic patterns (exp. and calcd.) of different quadruply positive charged bismuth oxido nanocluster cations detected in the gas phase generated from compound **C-1:Sm** after electrospraying from MeCN/dmso. $[Bi_{38}O_{45}(NO_3)_{20}(dmso)_7]^{4+}$ (**I**), $[Bi_{38}O_{45}(NO_3)_{20}(dmso)_6]^{4+}$ (**II**), $[Bi_{38}O_{46}(NO_3)_{18}(dmso)_7]^{4+}$ (**V**), $[Bi_{38}O_{46}(NO_3)_{18}(dmso)_6]^{4+}$ (**VI**), $[Bi_{38}O_{46}(NO_3)_{18}(dmso)_5]^{4+}$ (**VII**), $[Bi_{37}SmO_{45}(NO_3)_{20}(dmso)_8]^{4+}$ (**LXXV**), $[Bi_{37}SmO_{45}(NO_3)_{20}(dmso)_7]^{4+}$ (**LXXVI**), $[Bi_{37}SmO_{46}(NO_3)_{18}(dmso)_7]^{4+}$ (**LXXVII**), $[Bi_{37}SmO_{46}(NO_3)_{18}(dmso)_6]^{4+}$ (**LXXVIII**), $[Bi_{36}Sm_2O_{46}(NO_3)_{18}(dmso)_7]^{4+}$ (**LXXVIX**), $[Bi_{36}Sm_2O_{46}(NO_3)_{18}(dmso)_6]^{4+}$ (**LXXX**), $[Bi_{35}Sm_3O_{46}(NO_3)_{18}(dmso)_7]^{4+}$ (**LXXXI**), $[Bi_{35}Sm_3O_{46}(NO_3)_{18}(dmso)_6]^{4+}$ (**LXXXII**).

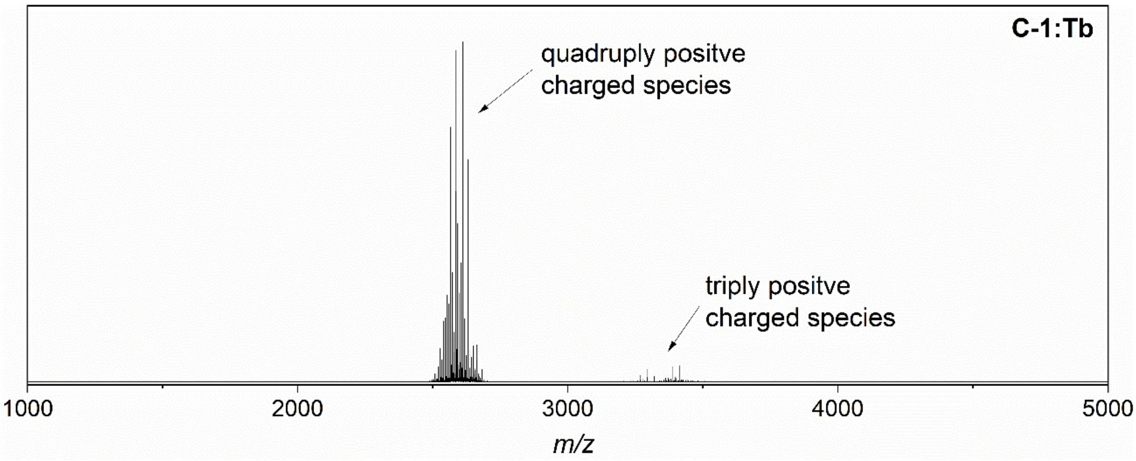

Figure S 31. Survey ESI-MS spectra generated from cluster **C-1:Tb** after electrospraying from MeCN/dmso, with marked triply and quadruply positive charged cationic species.

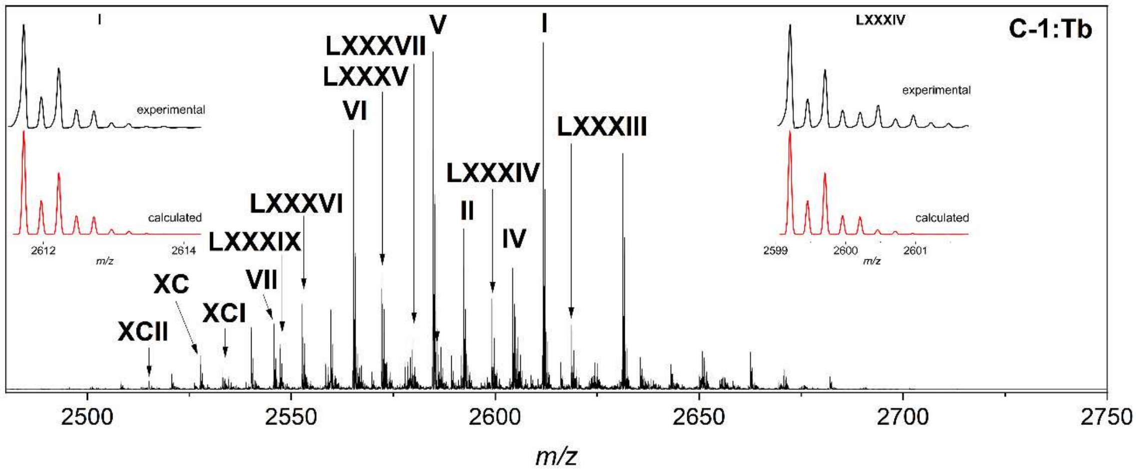

Figure S 32. Part of the ESI mass spectrum of compound **C-1:Tb** electrosprayed from MeCN/dmso showing quadruply positive charged homometallic cations (**I**–**VII**) with $[Bi_{38}O_{45}(NO_3)_{20}(dmso)_7]^{4+}$ (**I**, *m*/*z* = 2611.7181) and heterobimetallic BiO-NC cations (**LXXIII**–**XCII**) with $[Bi_{37}TbO_{45}(NO_3)_{20}(dmso)_7]^{4+}$ (**LXXXIV**, *m*/*z* = 2599.2057) showing the highest abundance.

Table S 12. Selection of quadruply positive charged bismuth oxido nanocluster cations detected in the survey mass spectrum of BiO-NC **C-1:Tb** electrosprayed from MeCN/dmso. Assignment was carried out using the most abundant *m/z* signals.

Label	Cation	***m/z***	
		**calculated**	**BiO-NC C-1:Tb**
**I**	$[Bi_{38}O_{45}(NO_3)_{20}(dmso)_7]^{4+}$	2611.7195	2611.7181
**II**	$[Bi_{38}O_{45}(NO_3)_{20}(dmso)_6]^{4+}$	2592.2160	2592.2158
**IV**	$[Bi_{38}O_{46}(NO_3)_{18}(dmso)_8]^{4+}$	2604.2278	2604.2277
**V**	$[Bi_{38}O_{46}(NO_3)_{18}(dmso)_7]^{4+}$	2584.7243	2584.7251
**VI**	$[Bi_{38}O_{46}(NO_3)_{18}(dmso)_6]^{4+}$	2565.2208	2565.2222
**VII**	$[Bi_{38}O_{46}(NO_3)_{18}(dmso)_5]^{4+}$	2545.7174	2545.7184
**LXXXIII**	$[Bi_{37}TbO_{45}(NO_3)_{20}(dmso)_8]^{4+}$	2618.7092	2618.7079
**LXXXIV**	$[Bi_{37}TbO_{45}(NO_3)_{20}(dmso)_7]^{4+}$	2599.2057	2599.2057
**LXXXV**	$[Bi_{37}TbO_{46}(NO_3)_{18}(dmso)_7]^{4+}$	2572.2106	2572.2119
**LXXXVI**	$[Bi_{37}TbO_{46}(NO_3)_{18}(dmso)_6]^{4+}$	2552.7071	2552.7085
**LXXXVII**	$[Bi_{36}Tb_2O_{46}(NO_3)_{18}(dmso)_8]^{4+}$	2579.2003	2579.2045
**LXXXVIII**	$[Bi_{36}Tb_2O_{46}(NO_3)_{18}(dmso)_7]^{4+}$	2559.6968	2559.6990
**LXXXIX**	$[Bi_{35}Tb_3O_{46}(NO_3)_{18}(dmso)_7]^{4+}$	2547.1830	2547.1861
**XC**	$[Bi_{35}Tb_3O_{46}(NO_3)_{18}(dmso)_6]^{4+}$	2527.6796	2527.6818
**XCI**	$[Bi_{34}Tb_4O_{46}(NO_3)_{18}(dmso)_7]^{4+}$	2534.6693	2534.6739
**XCII**	$[Bi_{34}Tb_4O_{46}(NO_3)_{18}(dmso)_6]^{4+}$	2515.1658	2515.1690

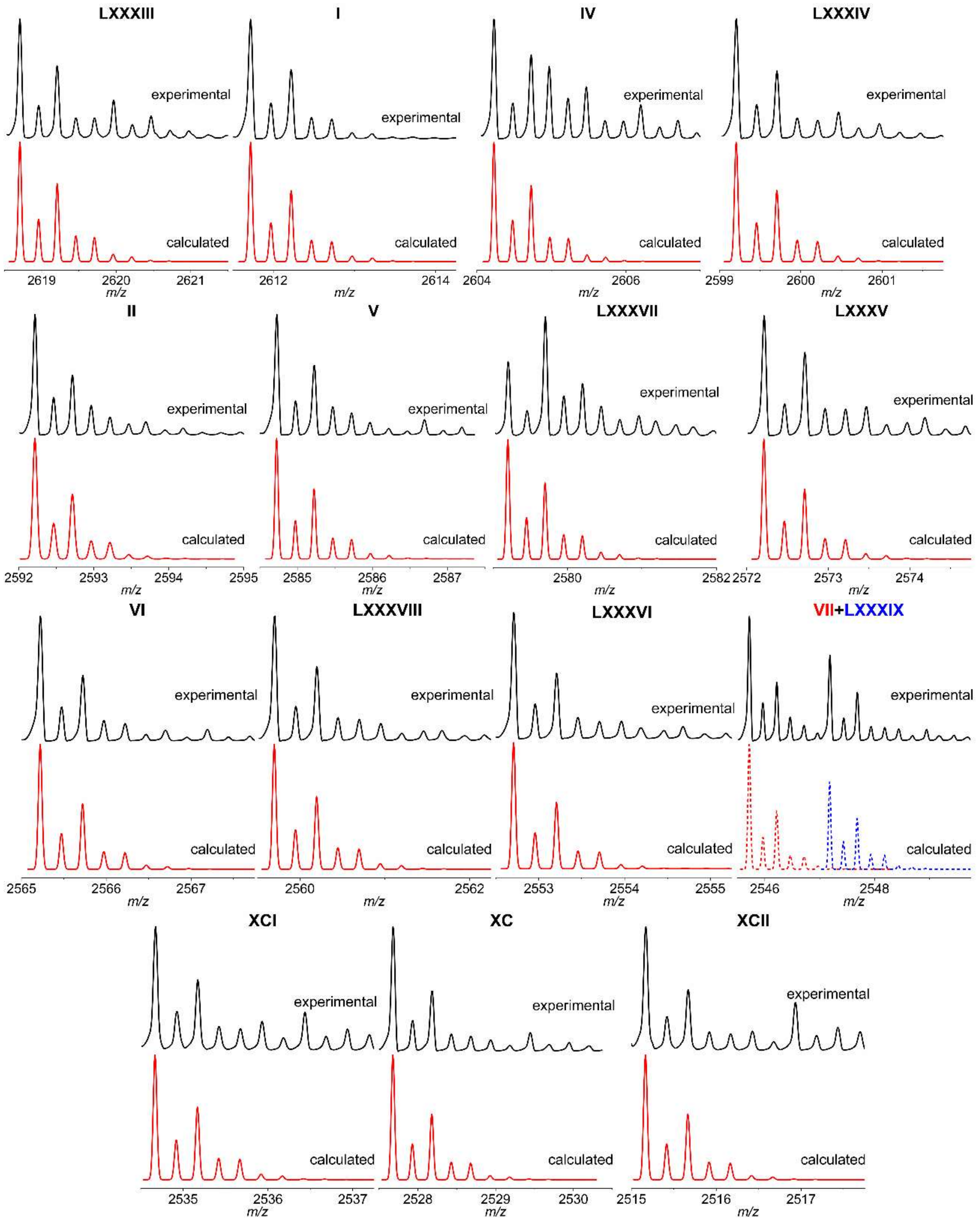

Figure S 33. Isotopic patterns (exp. and calcd.) of different quadruply positive charged bismuth oxido nanocluster cations detected in the gas phase generated from compound **C-1:Tb** after electrospraying from MeCN/dmso. $[Bi_{38}O_{45}(NO_3)_{20}(dmso)_7]^{4+}$ (**I**), $[Bi_{38}O_{45}(NO_3)_{20}(dmso)_6]^{4+}$ (**II**), $[Bi_{38}O_{46}(NO_3)_{18}(dmso)_7]^{4+}$ (**V**), $[Bi_{38}O_{46}(NO_3)_{18}(dmso)_6]^{4+}$ (**VI**), $[Bi_{38}O_{46}(NO_3)_{18}(dmso)_5]^{4+}$ (**VII**), $[Bi_{37}TbO_{45}(NO_3)_{20}(dmso)_8]^{4+}$ (**LXXXIII**), $[Bi_{37}TbO_{45}(NO_3)_{20}(dmso)_7]^{4+}$ (**LXXXIV**), $[Bi_{37}TbO_{46}(NO_3)_{18}(dmso)_7]^{4+}$ (**LXXXV**), $[Bi_{37}TbO_{46}(NO_3)_{18}(dmso)_6]^{4+}$ (**LXXXVI**), $[Bi_{36}Tb_2O_{46}(NO_3)_{18}(dmso)_8]^{4+}$ (**LXXXVII**), $[Bi_{36}Tb_2O_{46}(NO_3)_{18}(dmso)_7]^{4+}$ (**LXXXVIII**), $[Bi_{35}Tb_3O_{46}(NO_3)_{18}(dmso)_7]^{4+}$ (**LXXXIX**), $[Bi_{35}Tb_3O_{46}(NO_3)_{18}(dmso)_6]^{4+}$ (**XC**), $[Bi_{34}Tb_4O_{46}(NO_3)_{18}(dmso)_7]^{4+}$ (**XCI**), $[Bi_{34}Tb_4O_{46}(NO_3)_{18}(dmso)_6]^{4+}$ (**XCII**).

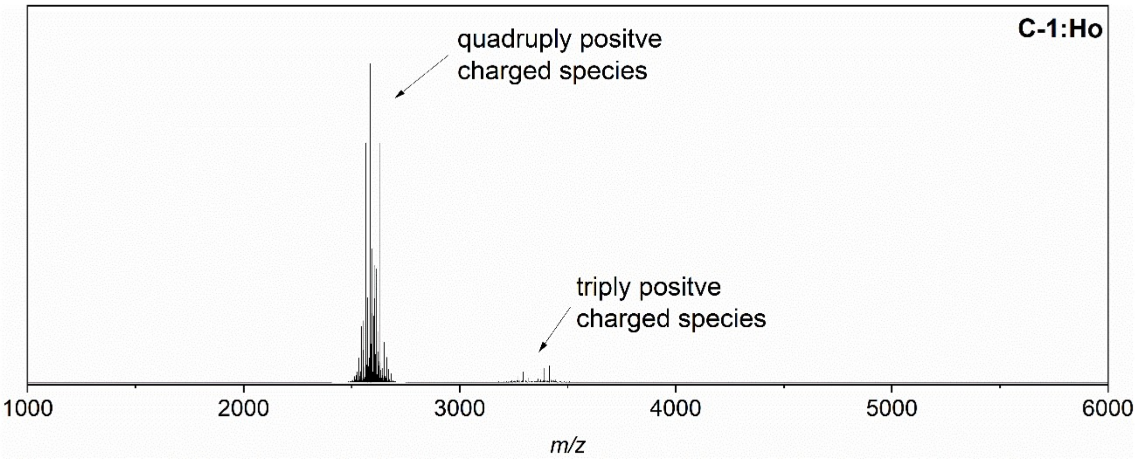

Figure S 34. Survey ESI-MS spectra generated from cluster **C-1:Ho** after electrospraying from MeCN/dmso, with marked triply and quadruply positive charged cationic species.

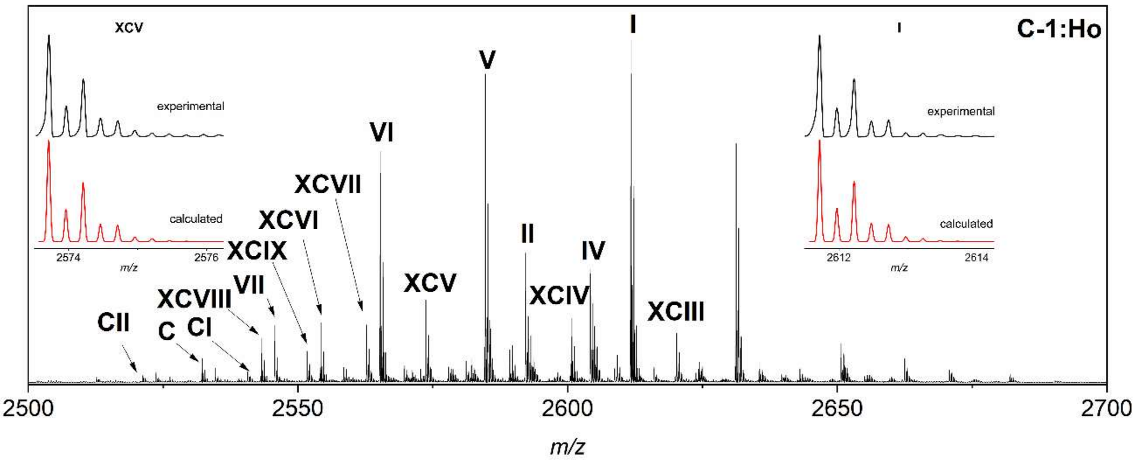

Figure S 35. Part of the ESI mass spectrum of compound **C-1:Ho** electrosprayed from MeCN/dmso showing quadruply positive charged homometallic cations (**I**–**VII**) with $[Bi_{38}O_{45}(NO_3)_{20}(dmso)_7]^{4+}$ (**I**, *m*/*z* = 2611.7181) and heterobimetallic BiO-NC cations (**XCIII**–**CII**) with $[Bi_{37}HoO_{46}(NO_3)_{18}(dmso)_7]^{4+}$ (**XCV**, *m*/*z* = 2599.2057) showing the highest abundance.

Table S 13. Selection of quadruply positive charged bismuth oxido nanocluster cations detected in the survey mass spectrum of BiO-NC **C-1:Ho** electrosprayed from MeCN/dmso. Assignment was carried out using the most abundant *m/z* signals.

Label	Cation	*m/z*	
		calculated	BiO-NC C-1:Ho
**I**	$[Bi_{38}O_{45}(NO_3)_{20}(dmso)_7]^{4+}$	2611.7195	2611.7176
**II**	$[Bi_{38}O_{45}(NO_3)_{20}(dmso)_6]^{4+}$	2592.2160	2592.2157
**IV**	$[Bi_{38}O_{46}(NO_3)_{18}(dmso)_8]^{4+}$	2604.2278	2604.2265
**V**	$[Bi_{38}O_{46}(NO_3)_{18}(dmso)_7]^{4+}$	2584.7243	2584.7247
**VI**	$[Bi_{38}O_{46}(NO_3)_{18}(dmso)_6]^{4+}$	2565.2208	2565.2219
**VII**	$[Bi_{38}O_{46}(NO_3)_{18}(dmso)_5]^{4+}$	2545.7174	2545.7183
**XCIII**	$[Bi_{37}HoO_{45}(NO_3)_{20}(dmso)_8]^{4+}$	2620.2105	2620.2083
**XCIV**	$[Bi_{37}HoO_{45}(NO_3)_{20}(dmso)_7]^{4+}$	2600.7070	2600.7065
**XCV**	$[Bi_{37}HoO_{46}(NO_3)_{18}(dmso)_7]^{4+}$	2573.7118	2573.7129
**XCVI**	$[Bi_{37}HoO_{46}(NO_3)_{18}(dmso)_6]^{4+}$	2554.2083	2554.2097
**XCVII**	$[Bi_{36}Ho_2O_{46}(NO_3)_{18}(dmso)_7]^{4+}$	2562.6993	2562.7006
**XCVIII**	$[Bi_{36}Ho_2O_{46}(NO_3)_{18}(dmso)_6]^{4+}$	2543.1958	2543.1973
**XCIX**	$[Bi_{35}Ho_3O_{46}(NO_3)_{18}(dmso)_7]^{4+}$	2551.6868	2551.6889
**C**	$[Bi_{35}Ho_3O_{46}(NO_3)_{18}(dmso)_6]^{4+}$	2532.1833	2532.1854
**CI**	$[Bi_{34}Ho_4O_{46}(NO_3)_{18}(dmso)_7]^{4+}$	2540.6743	2540.6777
**CII**	$[Bi_{34}Ho_4O_{46}(NO_3)_{18}(dmso)_6]^{4+}$	2521.1708	2521.1735

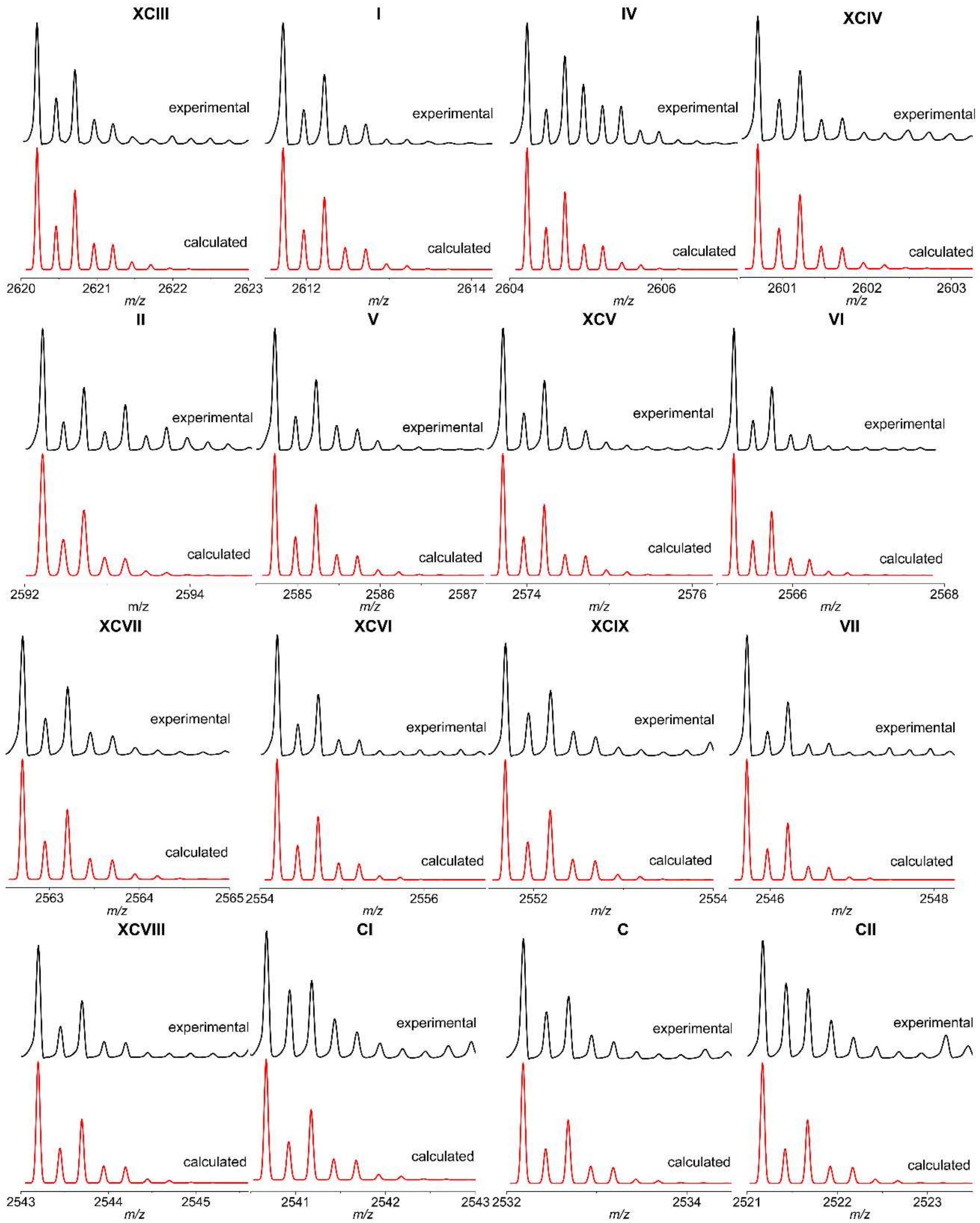

Figure S 36. Isotopic patterns (exp. and calcd.) of different quadruply positive charged bismuth oxido nanocluster cations detected in the gas phase generated from compound **C-1:Ho** after electrospraying from MeCN/dmso. $[Bi_{38}O_{45}(NO_3)_{20}(dmso)_7]^{4+}$ (**I**), $[Bi_{38}O_{45}(NO_3)_{20}(dmso)_6]^{4+}$ (**II**), $[Bi_{38}O_{46}(NO_3)_{18}(dmso)_7]^{4+}$ (**V**), $[Bi_{38}O_{46}(NO_3)_{18}(dmso)_6]^{4+}$ (**VI**), $[Bi_{38}O_{46}(NO_3)_{18}(dmso)_5]^{4+}$ (**VII**), $[Bi_{37}HoO_{45}(NO_3)_{20}(dmso)_8]^{4+}$ (**XCIII**), $[Bi_{37}HoO_{45}(NO_3)_{20}(dmso)_7]^{4+}$ (**XCIV**), $[Bi_{37}HoO_{46}(NO_3)_{18}(dmso)_7]^{4+}$ (**XCV**), $[Bi_{37}HoO_{46}(NO_3)_{18}(dmso)_6]^{4+}$ (**XCVI**), $[Bi_{36}Ho_2O_{46}(NO_3)_{18}(dmso)_7]^{4+}$ (**XCVII**), $[Bi_{36}Ho_2O_{46}(NO_3)_{18}(dmso)_6]^{4+}$ (**XCVIII**), $[Bi_{35}Ho_3O_{46}(NO_3)_{18}(dmso)_7]^{4+}$ (**XCIX**), $[Bi_{35}Ho_3O_{46}(NO_3)_{18}(dmso)_6]^{4+}$ (**C**), $[Bi_{34}Ho_4O_{46}(NO_3)_{18}(dmso)_7]^{4+}$ (**CI**), $[Bi_{34}Ho_4O_{46}(NO_3)_{18}(dmso)_6]^{4+}$ (**CII**).

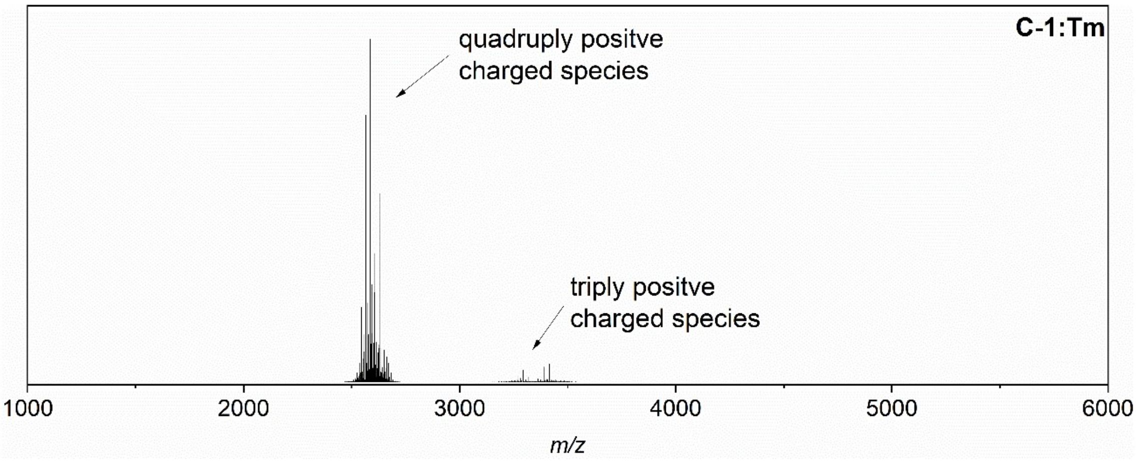

Figure S 37. Survey ESI-MS spectra generated from cluster **C-1:Tm** after electrospraying from MeCN/dmso, with marked triply and quadruply positive charged cationic species.

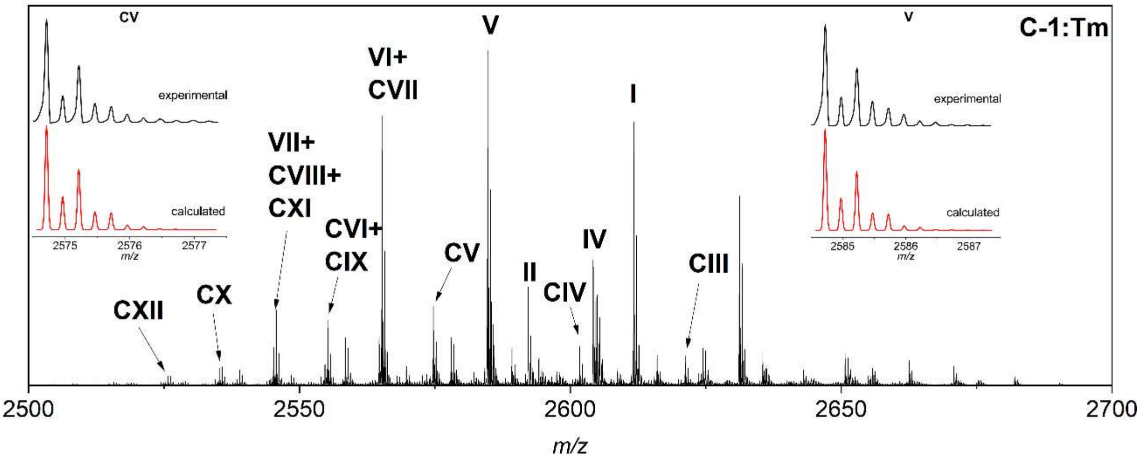

Figure S 38. Part of the ESI mass spectrum of compound **C-1:Tm** electrosprayed from MeCN/DMSO showing quadruply positive charged homometallic cations (**I**–**VII**) with $[Bi_{38}O_{45}(NO_3)_{20}(dmso)_7]^{4+}$ (**V**, *m*/*z* = 2584.7242) and heterobimetallic BiO-NC cations (**XCIII**–**CII**) with $[Bi_{37}TmO_{46}(NO_3)_{18}(dmso)_7]^{4+}$ (**CV**, *m*/*z* = 2574.7120) showing the highest abundance.

Table S 14. Selection of quadruply positive charged bismuth oxido nanocluster cations detected in the survey mass spectrum of BiO-NC **C-1:Tm** electrosprayed from MeCN/dmso. Assignment was carried out using the most abundant *m/z* signals.

**Label**	**Cation**	***m/z***	
		**calculated**	**BiO-NC C-1:Tm**
**I**	$[Bi_{38}O_{45}(NO_3)_{20}(dmso)_7]^{4+}$	2611.7195	2611.7169
**II**	$[Bi_{38}O_{45}(NO_3)_{20}(dmso)_6]^{4+}$	2592.2160	2592.2143
**IV**	$[Bi_{38}O_{46}(NO_3)_{18}(dmso)_8]^{4+}$	2604.2278	2604.2265
**V**	$[Bi_{38}O_{46}(NO_3)_{18}(dmso)_7]^{4+}$	2584.7243	2584.7242
**VI**	$[Bi_{38}O_{46}(NO_3)_{18}(dmso)_6]^{4+}$	2565.2208	2565.2199
**VII**	$[Bi_{38}O_{46}(NO_3)_{18}(dmso)_5]^{4+}$	2545.7174	2545.7132
**CIII**	$[Bi_{37}TmO_{45}(NO_3)_{20}(dmso)_8]^{4+}$	2621.2114	2621.2091
**CIV**	$[Bi_{37}TmO_{45}(NO_3)_{20}(dmso)_7]^{4+}$	2601.7080	2601.7063
**CV**	$[Bi_{37}TmO_{46}(NO_3)_{18}(dmso)_7]^{4+}$	2574.7128	2574.7120
**CVI**	$[Bi_{37}TmO_{46}(NO_3)_{18}(dmso)_6]^{4+}$	2555.2093	2555.2063
**CVII**	$[Bi_{36}Tm_2O_{46}(NO_3)_{18}(dmso)_7]^{4+}$	2564.7012	2564.7017
**CVIII**	$[Bi_{36}Tm_2O_{46}(NO_3)_{18}(dmso)_6]^{4+}$	2545.1978	2545.1971
**CIX**	$[Bi_{35}Tm_3O_{46}(NO_3)_{18}(dmso)_7]^{4+}$	2554.6897	2554.6921
**CX**	$[Bi_{35}Tm_3O_{46}(NO_3)_{18}(dmso)_6]^{4+}$	2535.1862	2535.1874
**CXI**	$[Bi_{34}Tm_4O_{46}(NO_3)_{18}(dmso)_7]^{4+}$	2544.6781	2544.6833
**CXII**	$[Bi_{34}Tm_4O_{46}(NO_3)_{18}(dmso)_6]^{4+}$	2525.1747	2525.1787

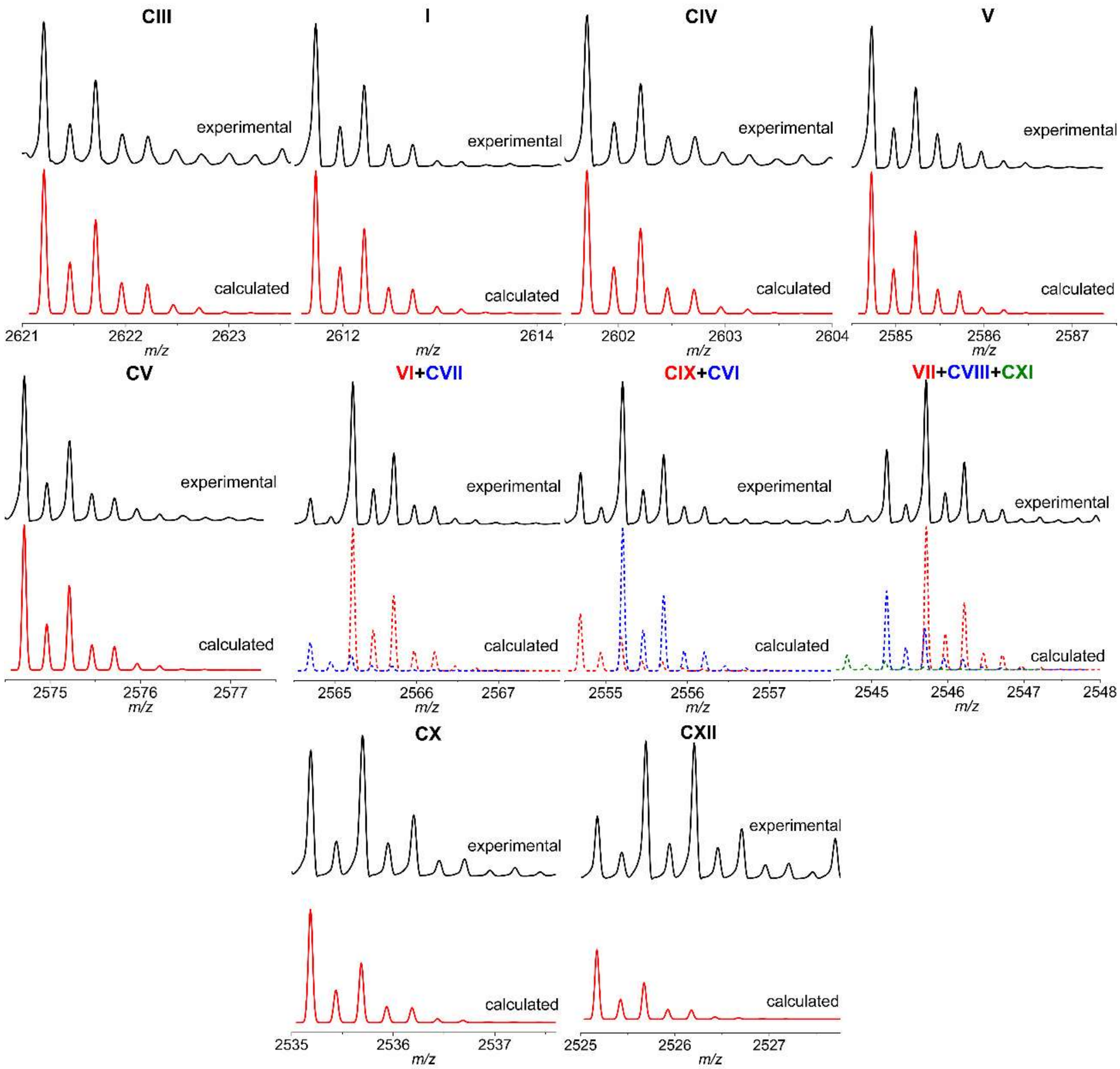

Figure S 39. Isotopic patterns (exp. and calcd.) of different quadruply positive charged bismuth oxido nanocluster cations detected in the gas phase generated from compound **C-1:Tm** after electrospraying from MeCN/dmso. $[Bi_{38}O_{45}(NO_3)_{20}(dmso)_7]^{4+}$ (**I**), $[Bi_{38}O_{45}(NO_3)_{20}(dmso)_6]^{4+}$ (**II**), $[Bi_{38}O_{46}(NO_3)_{18}(dmso)_7]^{4+}$ (**V**), $[Bi_{38}O_{46}(NO_3)_{18}(dmso)_6]^{4+}$ (**VI**), $[Bi_{38}O_{46}(NO_3)_{18}(dmso)_5]^{4+}$ (**VII**), $[Bi_{37}TmO_{46}(NO_3)_{18}(dmso)_6]^{4+}$ (**CIII**), $[Bi_{37}TmO_{45}(NO_3)_{20}(dmso)_7]^{4+}$ (**CIV**), $[Bi_{37}TmO_{46}(NO_3)_{18}(dmso)_7]^{4+}$ (**CV**), $[Bi_{37}TmO_{46}(NO_3)_{18}(dmso)_6]^{4+}$ (**CVI**), $[Bi_{36}Tm_2O_{46}(NO_3)_{18}(dmso)_7]^{4+}$ (**CVII**), $[Bi_{36}Tm_2O_{46}(NO_3)_{18}(dmso)_6]^{4+}$ (**CVIII**), $[Bi_{35}Tm_3O_{46}(NO_3)_{18}(dmso)_7]^{4+}$ (**CIX**), $[Bi_{35}Tm_3O_{46}(NO_3)_{18}(dmso)_6]^{4+}$ (**CX**), $[Bi_{34}Tm_4O_{46}(NO_3)_{18}(dmso)_7]^{4+}$ (**CXI**), $[Bi_{34}Tm_4O_{46}(NO_3)_{18}(dmso)_6]^{4+}$ (**CXII**).

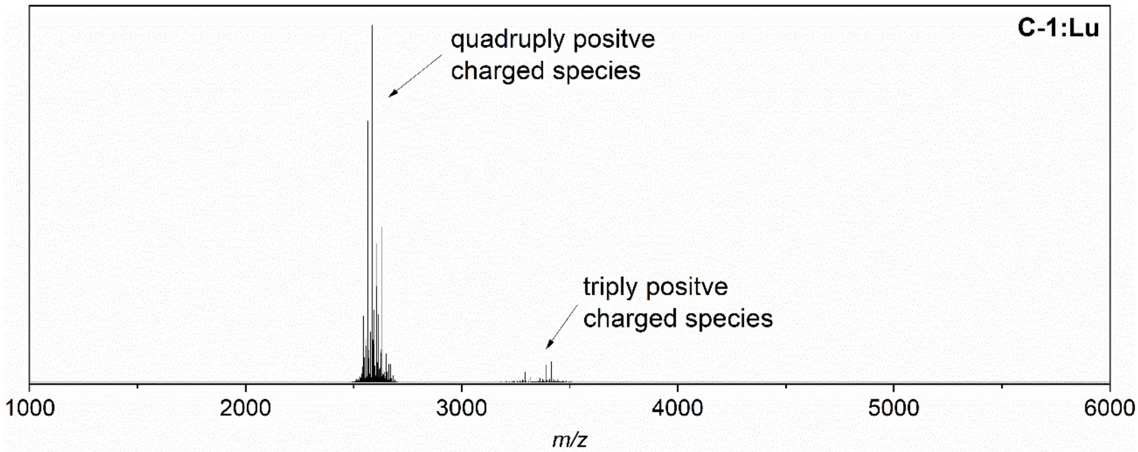

Figure S 40. Survey ESI-MS spectra generated from cluster **C-1:Lu** after electrospraying from MeCN/dmso, with marked triply and quadruply positive charged cationic species.

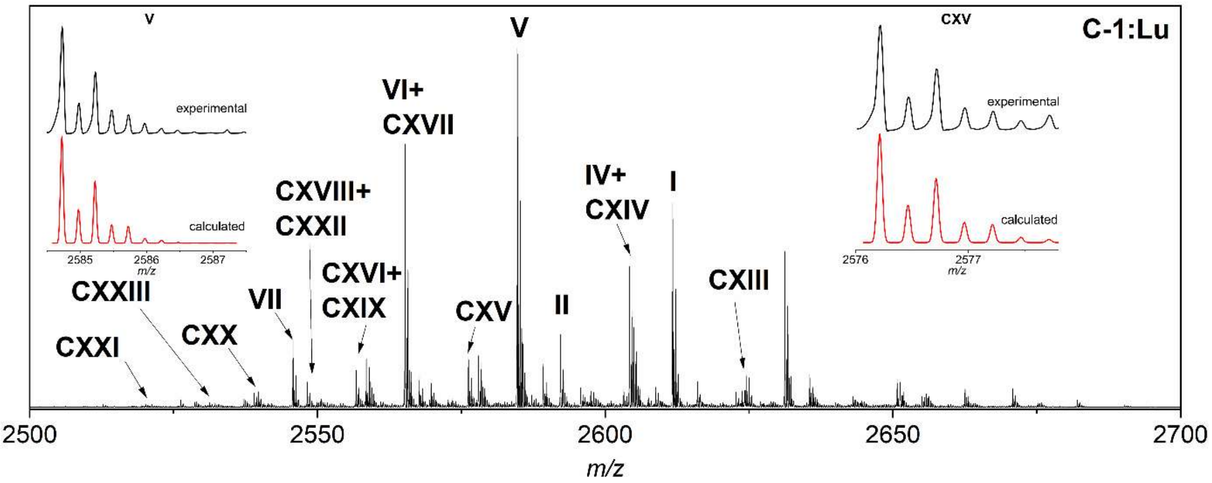

Figure S 41. Part of the ESI mass spectrum of compound **C-1:Tm** electrosprayed from MeCN/dmso showing quadruply positive charged homometallic cations (**I**–**VII**) with $[Bi_{38}O_{45}(NO_3)_{20}(dmso)_7]^{4+}$ (**V**, *m*/*z* = 2584.7276) and heterobimetallic BiO-NC cations (**XCIII**–**CII**) with $[Bi_{37}LuO_{46}(NO_3)_{18}(dmso)_7]^{4+}$ (**CXV**, *m*/*z* = 2576.2176) showing the highest abundance.

Table S 15. Selection of quadruply positive charged bismuth oxido nanocluster cations detected in the survey mass spectrum of BiO-NC **C-1:Lu** electrosprayed from MeCN/dmso. Assignment was carried out using the most abundant *m/z* signals.

Label	Cation	*m/z*	
		**calculated**	**BiO-NC C-1:Lu**
**I**	$[Bi_{38}O_{45}(NO_3)_{20}(dmso)_7]^{4+}$	2611.7195	2611.7196
**II**	$[Bi_{38}O_{45}(NO_3)_{20}(dmso)_6]^{4+}$	2592.2160	2592.2184
**IV**	$[Bi_{38}O_{46}(NO_3)_{18}(dmso)_8]^{4+}$	2604.2278	2604.2286
**V**	$[Bi_{38}O_{46}(NO_3)_{18}(dmso)_7]^{4+}$	2584.7243	2584.7276
**VI**	$[Bi_{38}O_{46}(NO_3)_{18}(dmso)_6]^{4+}$	2565.2208	2565.2245
**VII**	$[Bi_{38}O_{46}(NO_3)_{18}(dmso)_5]^{4+}$	2545.7174	2545.7205
**CXIII**	$[Bi_{37}LuO_{45}(NO_3)_{20}(dmso)_8]^{4+}$	2622.7131	2622.7141
**CXIV**	$[Bi_{37}LuO_{45}(NO_3)_{20}(dmso)_7]^{4+}$	2603.2096	2603.2133
**CXV**	$[Bi_{37}LuO_{46}(NO_3)_{18}(dmso)_7]^{4+}$	2576.2144	2576.2176
**CXVI**	$[Bi_{37}LuO_{46}(NO_3)_{18}(dmso)_6]^{4+}$	2556.7109	2556.7144
**CXVII**	$[Bi_{36}Lu_2O_{46}(NO_3)_{18}(dmso)_7]^{4+}$	2567.7045	2567.7084
**CXVIII**	$[Bi_{36}Lu_2O_{46}(NO_3)_{18}(dmso)_6]^{4+}$	2548.2010	2548.2050
**CXIX**	$[Bi_{35}Lu_3O_{46}(NO_3)_{18}(dmso)_7]^{4+}$	2559.1946	2559.2069
**CXX**	$[Bi_{35}Lu_3O_{46}(NO_3)_{18}(dmso)_6]^{4+}$	2539.6911	2539.6985
**CXXI**	$[Bi_{35}Lu_3O_{46}(NO_3)_{18}(dmso)_5]^{4+}$	2520.1876	2520.1937
**CXXII**	$[Bi_{34}Lu_4O_{46}(NO_3)_{18}(dmso)_7]^{4+}$	2550.6847	2550.6947
**CXXIII**	$[Bi_{34}Lu_4O_{46}(NO_3)_{18}(dmso)_6]^{4+}$	2531.1812	2531.1882

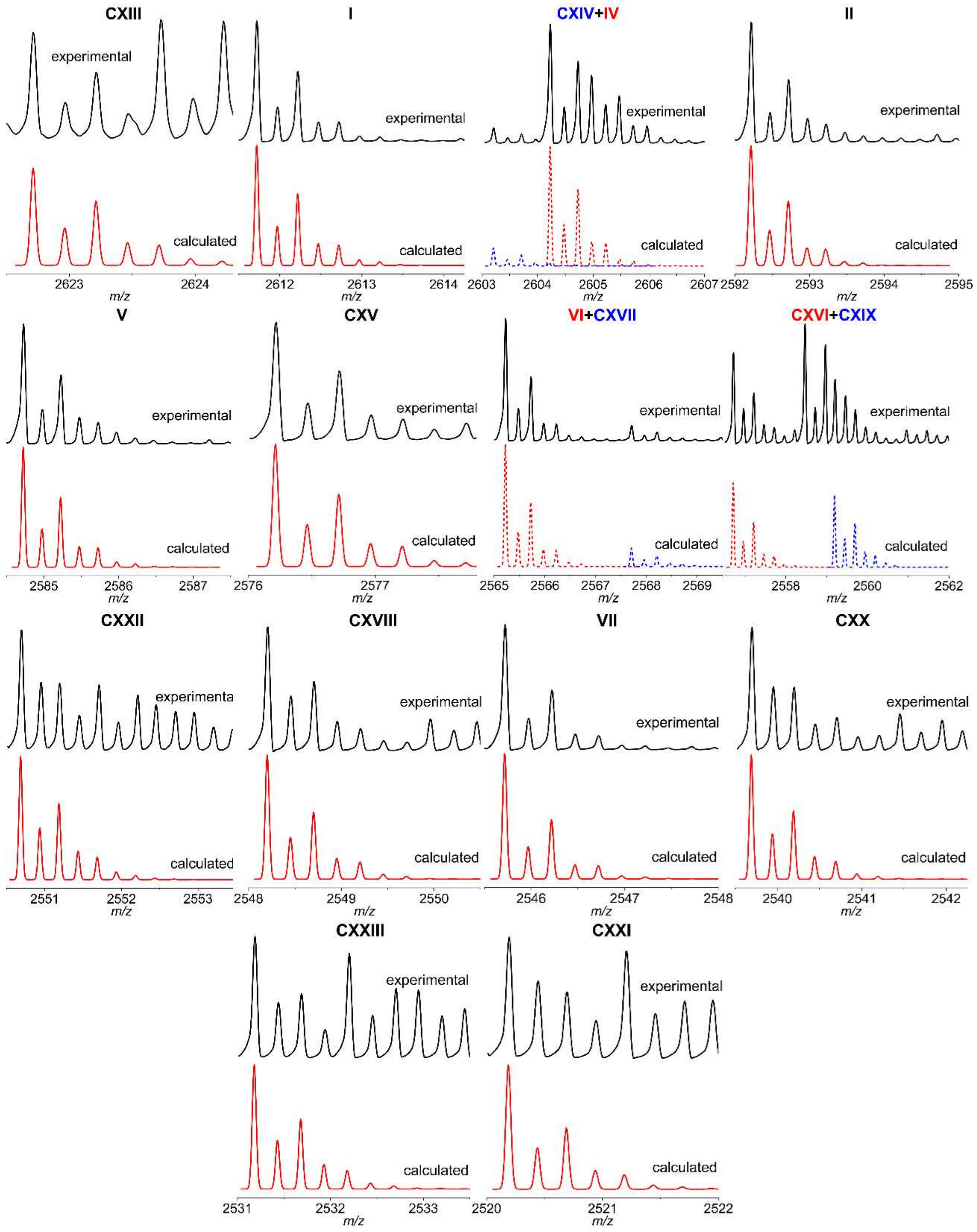

Figure S 42. Isotopic patterns (exp. and calcd.) of different quadruply positive charged bismuth oxido nanocluster cations detected in the gas phase generated from compound **C-1:Lu** after electrospraying from MeCN/dmso. $[Bi_{38}O_{45}(NO_3)_{20}(dmso)_7]^{4+}$ (**I**), $[Bi_{38}O_{45}(NO_3)_{20}(dmso)_6]^{4+}$ (**II**), $[Bi_{38}O_{46}(NO_3)_{18}(dmso)_7]^{4+}$ (**V**), $[Bi_{38}O_{46}(NO_3)_{18}(dmso)_6]^{4+}$ (**VI**), $[Bi_{38}O_{46}(NO_3)_{18}(dmso)_5]^{4+}$ (**VII**), $[Bi_{37}LuO_{45}(NO_3)_{20}(dmso)_8]^{4++}$ (**CXIII**), $[Bi_{37}LuO_{45}(NO_3)_{20}(dmso)_7]^{4+}$ (**CXIV**), $[Bi_{37}LuO_{46}(NO_3)_{18}(dmso)_7]^{4+}$ (**CXV**), $[Bi_{37}LuO_{46}(NO_3)_{18}(dmso)_6]^{4+}$ (**CXVI**), $[Bi_{36}Lu_2O_{46}(NO_3)_{18}(dmso)_7]^{4+}$ (**CXVII**), $[Bi_{36}Lu_2O_{46}(NO_3)_{18}(dmso)_6]^{4+}$ (**CXVIII**), $[Bi_{35}Lu_3O_{46}(NO_3)_{18}(dmso)_7]^{4+}$ (**CXIX**), $[Bi_{35}Lu_3O_{46}(NO_3)_{18}(dmso)_6]^{4+}$ (**CXX**), $[Bi_{35}Lu_3O_{46}(NO_3)_{18}(dmso)_5]^{4+}$ (**CXXI**), $[Bi_{34}Lu_4O_{46}(NO_3)_{18}(dmso)_7]^{4+}$ (**CXXII**), $[Bi_{34}Lu_4O_{46}(NO_3)_{18}(dmso)_6]^{4+}$ (**CXXIII**).

IR Studies:

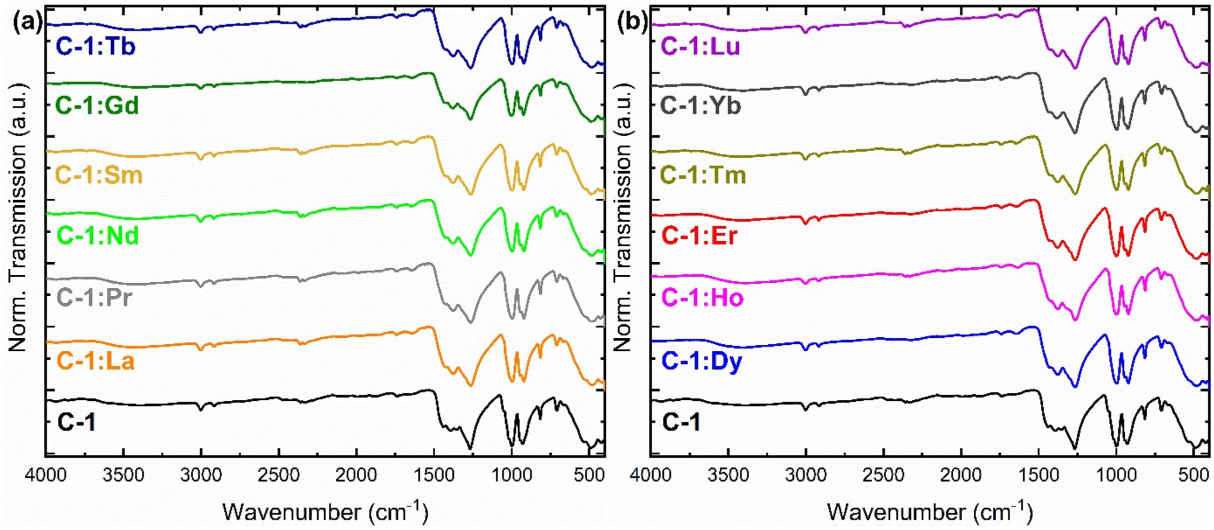

Figure S 43. ATR-IR spectra of different nitrate substituted BiO-NCs (a) **C-1** and **C-1:Ln (**Ln = La, Pr, Nd, Sm, Gd, Tb) and (b) **C-1** and **C-1:Ln (**Ln = Dy, Ho, Er, Tm, Yb, Lu).

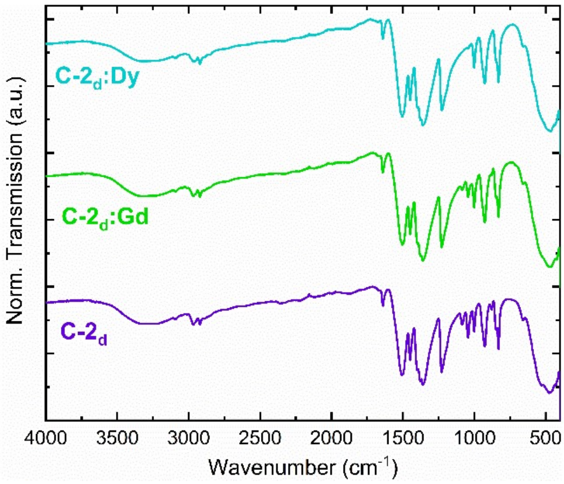

Figure S 44. ATR-IR spectra of the methacrylate substituted BiO-NCs **C-2$_d$**, **C-2$_d$:Gd, C-2$_d$:Dy**.

PXRD:

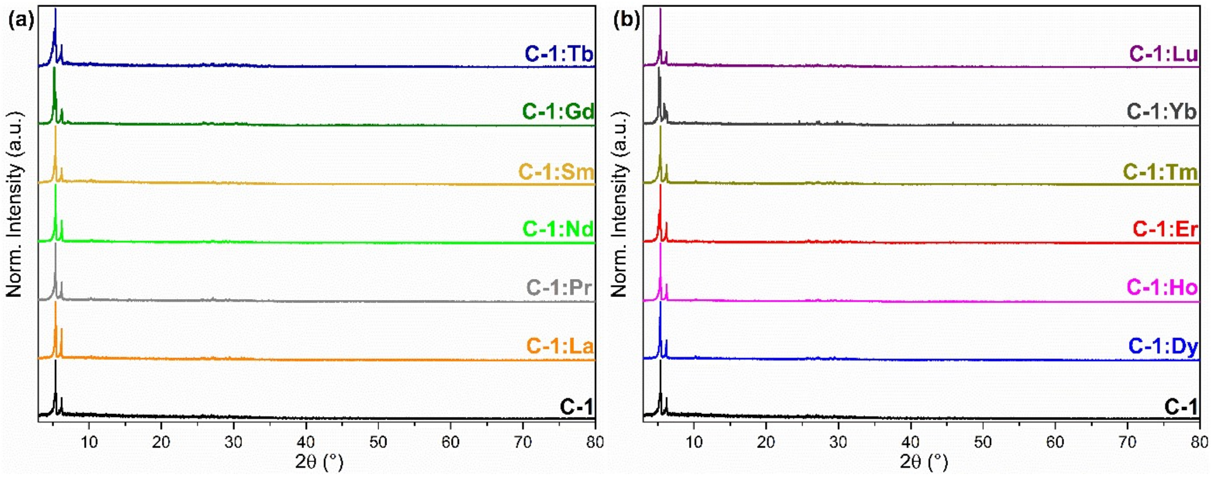

Figure S 45. Comparison of the PXRD patterns of undoped **C-1** (a) with the different lanthanide doped BiO-NCs **C-1:Ln** (Ln = La, Pr, Nd, Sm, Gd, Tb) and (b) with the different lanthanide doped BiO-NCs **C-1:Ln** (Ln = Dy, Ho, Er, Tm, Yb, Lu).

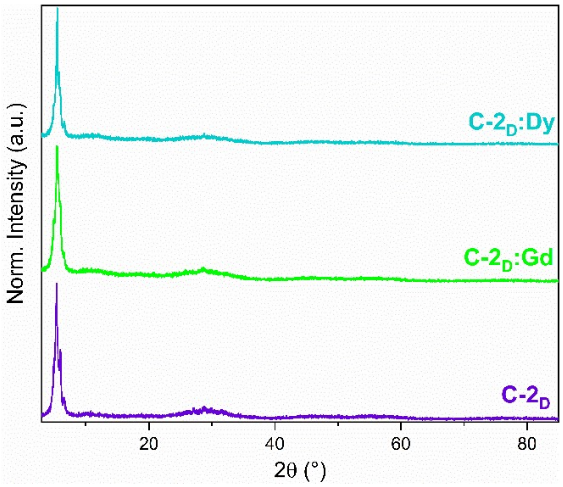

Figure S 46. PXRD pattern of the methacrylate-substituted lanthanide doped BiO-NCs **C-2$_d$:Ln** (Ln = Dy, Gd) and undoped BiO-NC **C-2$_d$**.

Table S 16. Overview of the main diffraction 2θ angles in the powder diffraction pattern and calculated interlayer distance of close packed BiO-NC arrangement of the different BiO-NCs **C-1:Ln**, **C-2$_d$:Gd**, **C-2$_d$:Dy**.

Cluster	2θ angle [°]	Interlayer distance [nm]
**C-1:La**	5.355	1.65
**C-1:Pr**	5.385	1.64
**C-1:Nd**	5.385	1.64
**C-1:Sm**	5.385	1.64
**C-1:Gd**	5.190	1.70
**C-1:Tb**	5.355	1.65
**C-1:Dy**	5.340	1.65
**C-1:Ho**	5.385	1.64
**C-1:Er**	5.340	1.65
**C-1:Tm**	5.385	1.64
**C-1:Yb**	5.160	1.71
**C-1:Lu**	5.355	1.65
**C-2$_d$:Gd***	5.415	1.63
**C-2$_d$:Dy***	5.445	1.62

SC XRD Results:

General remarks

The single crystals X-ray diffraction measurements of **C-1'**, **C-1:Gd**, **C-1:Dy** and **C-2$_E$:Gd** were performed exemplarily and are discussed in the following section. The molecular structures of **C-1:Gd** and **C-1:Dy** were compared with the undoped reference structure **C-1'**. Previously, **C-1** was measured using a different diffractometer, and thus was measured again on the Bruker Venture D8 diffractometer for better comparison.[5] It should be noted that for nitrate functionalized {$Bi_{38}O_{45}$} three crystallographic different crystal structures have been published, as a result of variation in reaction and crystallization parameters and that different degrees of disorder or distortion, flexible coordination and different amounts of packing solvent are observed.[5, 10] Note that for the study reported here a limit for a dative Bi–O bond with $d_{Bi–O}$ < 3.15 Å was used, corresponding to the upper limit for Bi–O distances as discussed in the literature in more detail previously.[8]

<u>Lanthanide dopant determination</u>: Significance of the dysprosium and gadolinium doping in the lanthanide doped BiO-NCs was tested in accordance to a previously reported procedure.[8, 11] Initially we calculated for each individual Bi atomic position whether it is occupied by the respective lanthanide, or not. We used EXYZ and EADP constrains to fix atoms to one and the same position and used a free parameter for refinement of the lanthanide content. In case that the thus calculated lanthanide content did not exceeds significantly its standard derivation with respect to the 3σ criteria,[12] we assume this position occupied by the lanthanide dopants (at least to a minor extend). This refers to the compounds denoted as **C-1:Gd, C-1:Dy**, **C-2$_E$:Gd**. Crystallographic information files (.cif files) of these compounds/refinements have been deposited with the CSD database.

<u>Determination of the significance of lanthanide doping/content:</u> In order to increase the statistical reliability and to eliminate potential interference effects with respect to the lanthanide occupancy we took the final .ins file of the final refined doped BiO-NC and performed another refinement against the .hkl file of the undoped BiO-NC. In selected cases that lead to the exclusion of lanthanide doping at certain Bi positions (3σ criteria, *cf*. below in more detail). In any case the thus obtained lanthanide contents were subtracted from the initially refined ones. This procedure provides a significance test and gives modified values of the Bi to Ln contents. **Please notice:** The thus obtained corrected values are discussed within this work, but not have been deposited with the CSD database.

<u>Crystal Structure of **C-1**, **C-1'** and **C-1:Gd**:</u>
The SC XRD data of **C-1:Gd** is compared to the new measurement of the undoped cluster structure **C-1'**. It should be noted that BiO-NC **C-1'** and doped BiO-NC **C-1:Gd** are isostructural and crystallize in the same space group with similar unit cell parameters compared to the previously published undoped cluster **C-1** (Table S 17).[5]

Table S 17. Crystallographic data for the Gd^{3+} doped BiO-NCs, $[Bi_{38}O_{45}(NO_3)_{24}(dmso)_{26}]$:Gd (**C-1:Gd**) and the undoped $[Bi_{38}O_{45}(NO_3)_{24}(dmso)_{26}]$ (**C-1'**) and $[Bi_{38}O_{45}(NO_3)_{24}(dmso)_{28}]\cdot$2dmso (**C-1**)[5].

	**C-1:Gd**	**C-1'**	**C-1**[5]
Empirical formula	$Bi_{37.31}Gd_{0.69}O_{143}C_{52}H_{156}N_{24}S_{26}$	$Bi_{38}O_{143}C_{52}H_{156}N_{24}S_{26}$	$Bi_{76}O_{294}C_{120}H_{360}N_{48}S_{60}$
M / g·mol^{-1}	12145.03	12180.80	24986.64
T / K	100 K	100 K	100 K
λ / Å	0.71073	0.71073	0.71073
Space group	*C2/c*	*C2/c*	*C2/c*
a / Å	29.5611(10)	29.575(2)	29.6563(2)
b / Å	27.7090(9)	27.758(2)	27.7650(2)
c / Å	29.1489(10)	29.166(2)	29.1273(3)
α / °	90	90	90
β / °	92.775(1)	92.847(3)	92.667(1)
γ / °	90	90	90
V / Å^3	23848.1(14)	23914(3)	23957.6(3)
Z	4	4	2
$D_{calc.}$ / g·cm^{-3}	3.383	3.383	3.464
μ / mm^{-1}	27.927	28.168	28.157
F(000)	21347	21400	22072
Reflections measured	704686	752496	112325
Independent Reflections	30847	32232	22940
$R_{Int.}$	0.0928	0.0740	0.0567
GOF on F^2	1.050	1.043	1.064
Final R indices [I>2σ(I)]	*R1* = 0.0295, *wR2* = 0.0686	*R1* = 0.0288, *wR2* = 0.0785	*R1* = 0.0468, *wR2* = 0.0949
R indices (all data)	*R1* = 0.0389, *wR2* = 0.0729	*R1* = 0.0361, *wR2* = 0.0820	*R1* = 0.0786, *wR2* = 0.1123

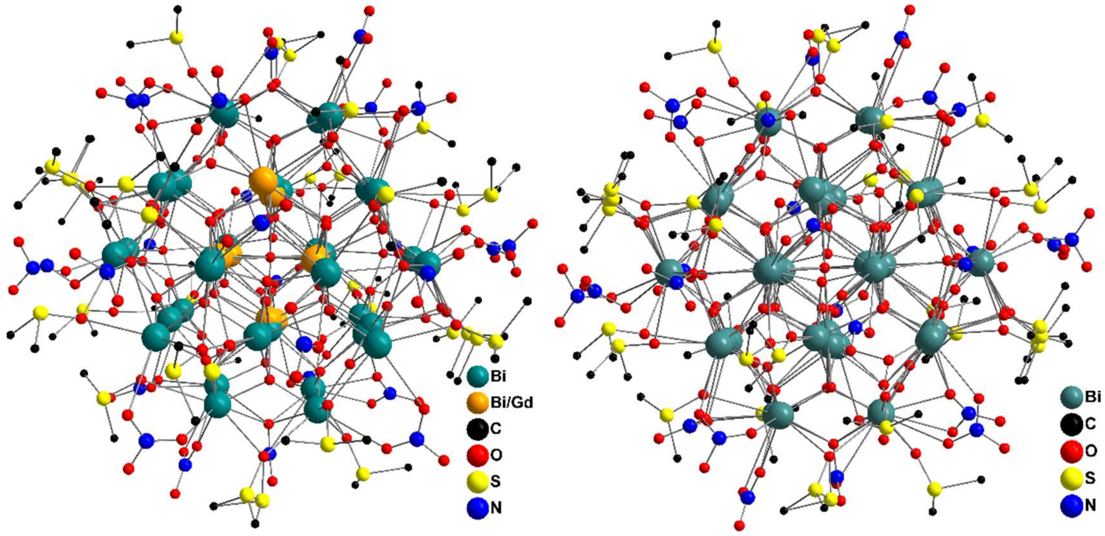

Figure S 47. "Ball-and-Stick" model of Gd^{3+} doped BiO-NC **C-1:Gd** (left) and undoped **C-1'** (right). The orange-colored atoms refer to Bi atom positions doped with Gd^{3+} (Table S 19). Hydrogen atoms are omitted for clarity.

Both clusters **C-1'** and **C-1:Gd** show a disorder of three crystallographic different bismuth positions (half cluster) namely Bi8 (Bi8/Bi8';occupation factors **C-1'** 0.701(10)/0.299(10); **C-1:Gd** 0.800(7)/0.200(7)) Bi9 (Bi9/Bi9'; occupation factors **C-1'** 0.531(14)/0.469(14); **C-1:Gd** 0.450(7)/0.550(7)) and Bi14 (Bi14/,Bi1'; occupation factors **C-1'** 0.582(18)/0.418(18); **C-1:Gd** 0.640(6)/0.360(6)), which could be refined separately showing reliable results. This was necessary, due to a high electron density near to the central Bi positions and was previously

reported for the methacrylate-substituted BiO-NC **C-2**,[8] and the europium doped BiO-NCs.[8] Further we noticed some disorder in ligand shell of the structures **C-1'**, first from a coordinated nitrate ligand, namely O4N'/O34N (associated N12', O4N', O4N'; N12, O35N', O36N') with occupation factors of 0.770(14)/0.230(14). As a result of the disorder if the oxygens O4N'/O34N in **C-1:Gd** they show different coordination modes, with O4N' μ_2-1κ*O*:2κ*O*' and O34N monodentate, whereas both are μ_2-1κ*O*:2κ*O*' in **C-1'**. Further four disordered dmso molecules were observed namely at the S3/S3' (associated O3S, C5, C6) with occupation factors of 0.5/0.5, at the O11S/O11' (associated S11,C21, C22; S11', C21', C22') with occupation factors of 0.837(8)/0.163(8), at the O13S/O13' (associated S13,C25, C26; S13', C25', C26') with occupation factors of 0.489(8)/0.511(8), and at O14S/O14' (associated S14,C27S, C28S ;S14', C27', C28') with occupation factors of 0.418(7)/0.582(7). These disorders lead to different coordination in **C-1'** with μ_2-1κ*O*:2κ*O* (O14') and μ_2-1κ*O*:2κ*O*:3κ*O* (O14S) and in **C-1:Gd** with μ_2-1κ*O*:2κ*O* (O13', O14') and μ_2-1κ*O*:2κ*O*:3κ*O* (O13S, O14S). The comparison of the determined Bi–O bond distances and coordination modes between **C-1'** and **C-1:Gd** is shown in Table S 18.

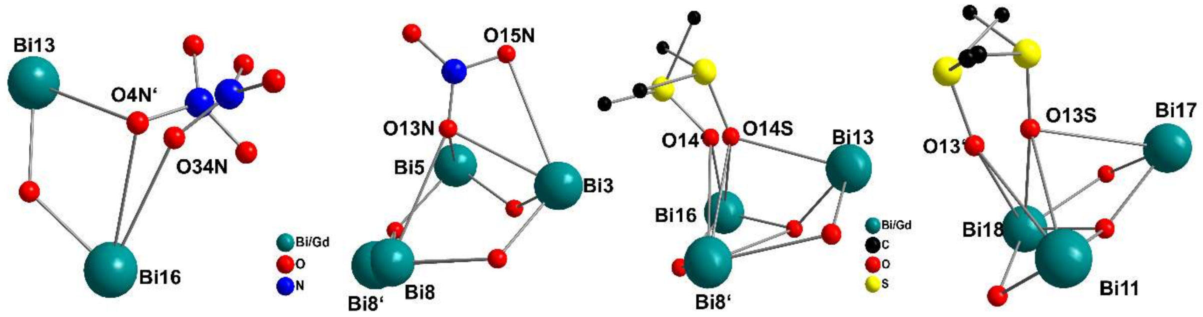

Figure S 48. Selected coordination modes of the different disordered nitrate anions and dmso molecules at **C-1:Gd**.

Table S 18. Summary of the Bi–O distance (*d*) ranges for different coordination modes of Bi in **C-1'** and **C-1:Gd**.

coordination mode	**C-1'**		**C-1:Gd**	
	number	*d* / Å	number	*d* / Å
**oxido**				
μ_3-1κ*O*:2κ*O*:3κ*O*	12	[2.084(5)–2.416(6)*]	12	[2.080(5)–2.394(7)]
μ_4-1κ*O*:2κ*O*:3κ*O*:4κ*O*	32	[2.063(4)–3.068(6)*]	32	[2.073(4)–2.995(5)]
μ_6-1κ*O*:2κ*O*:3κ*O*:4κ*O*:5κ*O*:6κ*O*	1	[2.423(5)–2.858(7)*]	1	[2.4028(16)–2.7631(12)]
**nitrato**				
monodentate			(2*)	[2.88(7)]
bidentate	2	[2.659(7)–2.886(12)]	2	[2.656(6)–2.897(9)]
μ_2-1κ*O*:2κ*O*'	6	[2.853(7)–3.109(9)]	4(2*)	[2.827(9)–3.048(7)]
μ_2-1κ*O*,*O*':2κ*O*'	4	[2.622(6)–3.073(8)]	4(2*)	[2.595(6)–3.103(6)]
μ_3-1κ*O*:2κ*O*':3κ*O*'	2	[2.794(6)–2.983(6)]	2	[2.800(6)–2.976(6)]
μ_3-1κ*O*:2κ*O*,*O*':3κ*O*'	2	[2.663(6)–3.074(5)]	2	[2.639(5)–3.084(5)]
μ_3-1κ*O*:2κ*O*: 3κ*O*,*O*'	2	[2.637(8)–3.137(11)]	(2*)	[2.629(7)–3.086(10)]
μ_4-1κ*O*:2κ*O*:3κ*O*:4κ*O*	4	[2.736(5)–3.063(6)]	4	[2.747(5)–3.062(5)]
μ_4-1κ*O*:2κ*O*:3κ*O*:4κ*O*,*O*'	2	[2.793(7)–3.095(7)]	2	[2.780(5)–3.107(7)]
non-coordinated	0		0	
**dmso**				
μ_2-1κ*O*:2κ*O*	22(2*)	[2.524(6)–2.971(6)]	20(4*)	[2.524(5)–2.971(6)]
μ_3-1κ*O*:2κ*O*:3κ*O*	2(2*)	[2.590(5)–3.091(11)]	2(4*)	[2.575(5)–3.116(14)]

*different coordination modes for disordered molecules (*cf.* Figure S 48)

For cluster **C-1:Gd** the Bi–O connectivity is the same compare to **C-1'** the bond distances are only slightly affected by the doping with 2.073(4) Å–2.995(5) Å (**C-1:Gd**) compared to

2.063(4) Å–3.068(4) Å (**C-1'**). The coordination mode of the nitrate is manly influenced by the partial distortion of the bismuth, nitrate and dmso.

<u>Significance test of Gd^{3+} doping in **C-1:Gd**:</u> As described above the europium occupations were compared with regard to its significance (Table S 19). Using this procedure, we determined, that in total five different (half cluster) crystallographic independent positions (Figure S 49) in the BiO-NC **C-1:Gd**, are doped with Gd^{3+} showing contents between 3.0(6)% and 10.5(11)%. It should be noted that three of these positions (Bi8, Bi9 and Bi14) are partially disordered with different occupation rates, which were considered in the calculations. From these respective disordered Bi positions only one position does not show Gd occupancy. This determination, thus lead to the modified formula [$Bi_{37.32(10)}Gd_{0.68(10)}O_{143}C_{52}H_{156}N_{24}S_{26}$] for **C-1:Gd**.

Table S 19. Evaluation of the significant amount of gadolinium using the difference in Gd amount of **C-1:Gd** and **C-1'(:Gd)** (form theoretical doping of **C-1'** with Gd) with respect to the 3σ criteria. In the case that the 3σ value is higher than the difference in the Dy amount, it is considered to be zero.

atom	**C-1:Gd** occupancy in %		**C-1'(:Gd)** occupancy in %		difference Gd in %	Standard derivation $\sigma = \sqrt{\Delta(1)^2 + \Delta(2)^2}$	3σ
	Bi	Gd	Bi	Gd			
Bi6	0.9697(60)	0.0303(60)	1.0000	–	**0.0303**	0.0060	0.0179
Bi8	0.7824(85)	0.0176(85)	0.8000	–	0.0176	0.0085	**0.0255**
Bi8'	0.2000	–	0.2000	–	0.0000	0.0000	**0.0000**
Bi9	0.3529(156)	0.0971(156)	0.4500	–	**0.0971**	0.0156	0.0469
Bi9'	0.5500	–	0.5500	–	0.0000	0.0000	**0.0000**
Bi14	0.5291(108)	0.1109(108)	0.6337(136)	0.0063(136)	**0.1046**	0.0174	0.0521
Bi1'	0.3600	–	0.3600	–	0.0000	0.0000	**0.0000**
Bi15	0.9107(64)	0.0893(64)	1.0000	–	**0.0893**	0.0064	0.0193
					∑ **0.3389**		

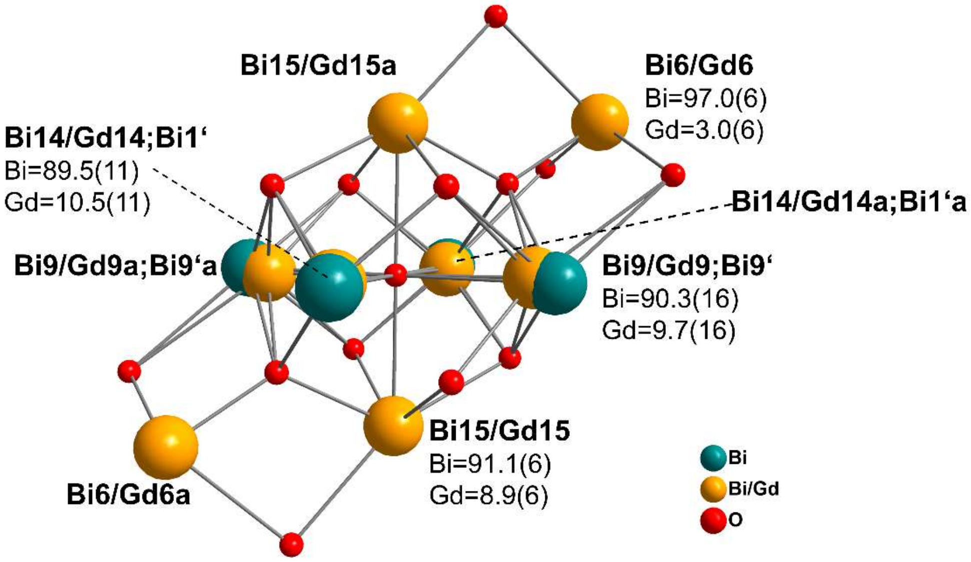

Figure S 49. "Ball-and-Stick" model showing the central {$(Bi/Gd)_6O_9$} motif of **C-1:Gd** highlighting the significant positions for mixed occupancy. Atom labels and calculated occupancy parameters of Bi/Gd determined from the significance test are given in %.

Crystal Structure of **C-1:Dy** and **C-1**:

The XRD data for Dy^{3+} doped BiO-NC **C-1:Dy** match with the previously published data of **C-1**.[5]

Table S 20. SC XRD crystallographic data for the Dy^{3+} doped BiO-NC, $[Bi_{38}O_{45}(NO_3)_{24}(dmso)_{26}]$:Dy·dmso (**C-1:Dy**) and the undoped BiO-NC $[Bi_{38}O_{45}(NO_3)_{24}(dmso)_{26}]$ (**C-1'**).

	**C-1:Dy**	**C-1'**
Empirical formula	$Bi_{36.97}Dy_{1.03}O_{144}C_{54}H_{162}N_{24}S_{27}$	$Bi_{38}O_{143}C_{52}H_{156}N_{24}S_{26}$
M / g·mol^{-1}	12210.85	12180.80
T / K	100 K	100 K
λ / Å	0.71073	0.71073
Space group	*C2/c*	*C2/c*
a / Å	29.631(6)	29.575(2)
b / Å	27.766(5)	27.758(2)
c / Å	29.180(6)	29.166(2)
α / °	90	90
β / °	92.776(9)	92.847(3)
γ / °	90	90
V / Å^3	23979(8)	23914(3)
Z	4	4
$D_{calc.}$ / g·cm^{-3}	3.383	3.383
μ / mm^{-1}	27.663	28.168
F(000)	21498	21400
Reflections measured	465186	752496
Independent Reflections	26140	32232
$R_{Int.}$	0.0900	0.0740
GOF on F^2	1.055	1.043
Final R indices [I>2σ(I)]	*R1* = 0.0375, *wR2* = 0.0953	*R1* = 0.0288, *wR2* = 0.0785
R indices (all data)	*R1* = 0.0476, *wR2* = 0.1005	*R1* = 0.0361, *wR2* = 0.0820

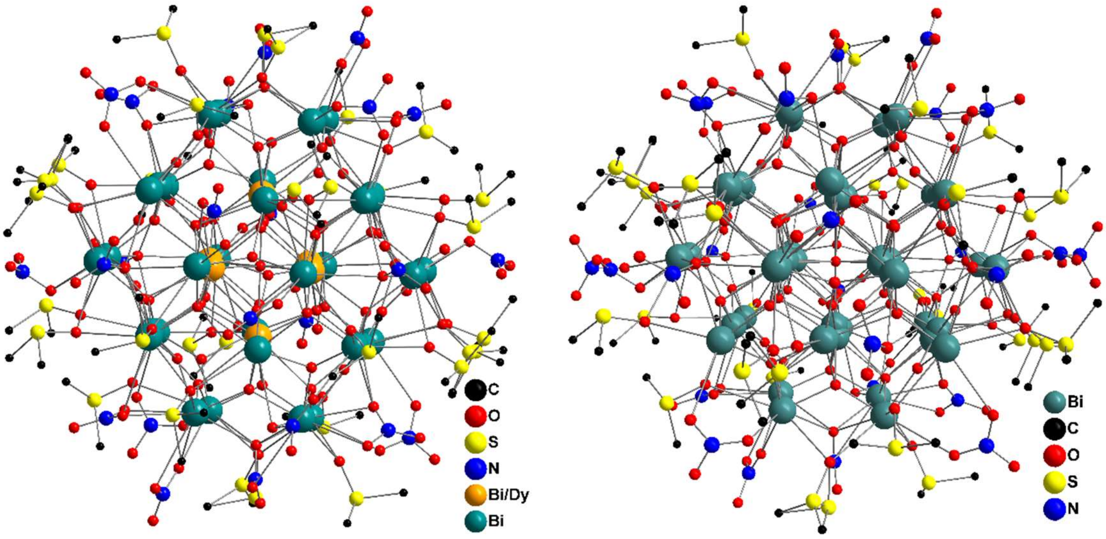

Figure S 50. "Ball-and-Stick" model of the Dy^{3+} doped BiO-NC **C-1:Dy** (*left*) and the undoped BiO-NC **C-1'** (*right*). The orange-colored atoms refer to Bi atom positions doped with Dy^{3+} (Table S 22). Hydrogen atoms as well as non-coordinated solvent molecules are omitted for clarity.

Table S 21. Summary of the Bi–O distance (*d*) ranges for different coordination modes of Bi in **C-1:Dy** and **C-1'**.

coordination mode	**C-1:Dy**		**C-1'**	
	number	*d* / Å	number	*d* / Å
**oxido**				
μ_3-1κ*O*:2κ*O*:3κ*O*	14	[2.080(6)–2.562(11)]	12	[2.084(5)–2.416(6)*]
μ_4-1κ*O*:2κ*O*:3κ*O*:4κ*O*	30	[2.082(6)–2.930(7)]	32	[2.063(4)–3.068(6)*]
μ_6-1κ*O*:2κ*O*:3κ*O*:4κ*O*:5κ*O*:6κ*O*	1	[2.5448(5)–2.6234(6)]	1	[2.423(5)–2.858(7)*]
**nitrato**				
bidentate	2	[2.669(9)–2.898(11)]	2	[2.659(7)–2.886(12)]
μ_2-1κ*O*:2κ*O*	2	[2.891(11)–2.972(12]		
μ_2-1κ*O*:2κ*O*'	4	[2.841(10)–3.048(10)]	6	[2.853(7)–3.109(9)]
μ_2-1κ*O*,*O*':2κ*O*'	4	[2.612(8)–3.115(9)]	4	[2.622(6)–3.073(8)]
μ_3-1κ*O*:2κ*O*':3κ*O*'	6	[2.655(11)–3.087(10)]	2	[2.794(6)–2.983(6)]
μ_3-1κ*O*:2κ*O*,*O*':3κ*O*'	0	-	2	[2.663(6)–3.074(5)]
μ_3-1κ*O*:2κ*O*: 3κ*O*,*O*'	0	-	2	[2.637(8)–3.137(11)]
μ_4-1κ*O*:2κ*O*:3κ*O*:4κ*O*	4	[2.748(7)–3.069(8)]	4	[2.736(5)–3.063(6)]
μ_4-1κ*O*:2κ*O*:3κ*O*:4κ*O*,*O*'	2	[2.794(7)–3.108(8)]	2	[2.793(7)–3.095(7)]
**dmso**				
μ_2-1κ*O*:2κ*O*	22	[2.534(8)–2.988(8)]	22(2*)	[2.524(6)–2.971(6)]
μ_3-1κ*O*:2κ*O*:3κ*O*	4	[2.593(7)–3.14(2)]	2(2*)	[2.590(5)–3.091(11)]
non-coordinated	1	< 3.15		

*different coordination modes for disordered molecules (*cf.* Figure S 50)

The doping further only slightly influenced the respective M–O distance in the $[Bi_{38}O_{45}]^{24+}$ cluster core structure ranges from 2.080(6) Å to 2.930(7) Å for **C-1:Dy** and from 2.053(11) Å to 2.981(11) Å for **C-1**. The coordination modes for the nitrate and dmso ligands do slightly differ in both structures (*cf.* Table S 21). As a result, the respective bond distance ranges are slightly affected, likewise.

<u>Significance test of Dy^{3+} doping in **C-1:Dy**</u>: As described above the dysprosium occupancies were compared regarding its significance (Table S 22). Using this procedure, we determined, that in total three (half cluster) crystallographic different positions (Figure S 51) in the BiO-NC **C-1:Dy**, are doped with Dy^{3+} showing contents between 11.7(11) % and 7.1(12) %. This determination, thus lead to the modified formula $[Bi_{37.42(7)}Dy_{0.58(7)}O_{144}C_{54}H_{162}N_{24}S_{27}]$ for **C-1:Dy**.

Table S 22. Evaluation of the significant amount of dysprosium using the difference in Dy amount of **C-1:Dy** and **C-1'(:Dy)** (form theoretical doping of **C-1'** with Dy) with respect to the 3σ criteria. In the case that the 3σ value is higher than the difference in the Dy amount, it is considered to be zero.

atom	**C-1:Dy** occupancy in %		**C-1'(:Dy)** occupancy in %		difference Dy in %	Standard derivation $\sigma = \sqrt{\Delta(1)^2 + \Delta(2)^2}$	3σ
	Bi	Dy	Bi	Dy			
Bi1	0.9868(105)	0.0132(105)	0.9607(121)	0.0393(121)	-0.0261	0.0160	**0.048**
Bi2	0.9961(103)	0.0039(103)	0.9798(124)	0.0202(124)	-0.0163	0.0161	**0.048**
Bi6	0.9809(105)	0.0191(105)	1.00		0.0191	0.0162	**0.049**
Bi7	0.9719(106)	0.0281(106)	0.9808(125)	0.0192(125)	0.0089	0.0163	**0.049**
Bi9	0.9286(116)	0.0714(116)	1.00		**0.0714**	0.0184	0.055
Bi11	0.8958(113)	0.1042(113)	1.00		**0.1042**	0.0177	0.053
Bi13	0.9649(105)	0.0351(105)	0.9581(125)	0.0429(125)	-0.0069	0.0163	**0.049**
Bi14	0.9632(103)	0.0368(103)	0.9836(122)	0.0164(122)	0.0204	0.0160	**0.048**
Bi15	0.8639(110)	0.1361(110)	0.9804(130)	0.0196(130)	**0.1165**	0.0170	0.051
Bi16	0.9585(104)	0.0415(104)	0.9579(122)	0.0421(122)	-0.0006	0.0160	**0.048**
Bi17	0.9728(104)	0.0272(104)	0.9976(122)	0.0024(122)	0.0247	0.01160	**0.048**
					**∑ 0.2921**		

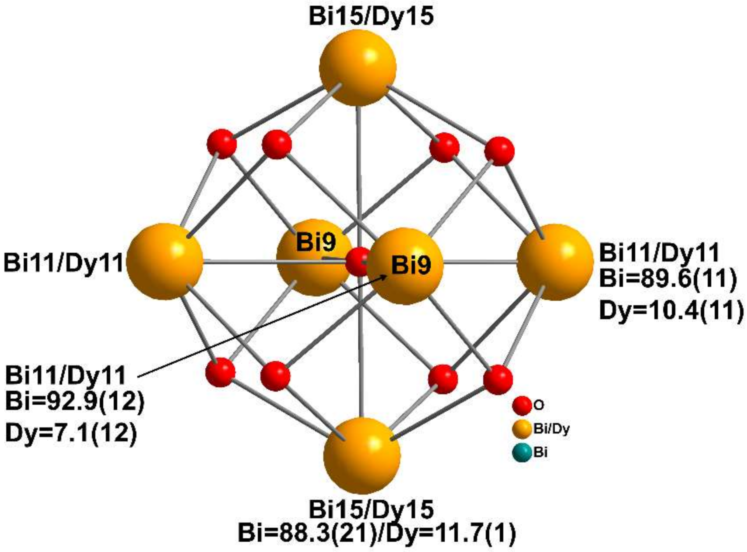

Figure S 51. "Ball-and-Stick" model of a part of BiO-NC **C-1:Dy** showing the central {(Bi/Dy_6)O_9} polyhedral including the atom labels and occupancy parameters of Bi/Dy determined from the significance test, the values are given in %.

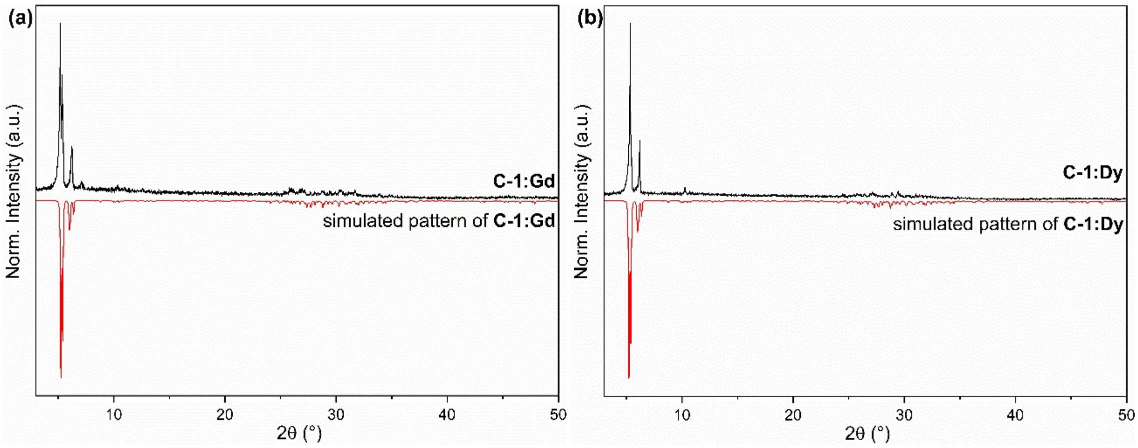

Figure S 52. Recorded PXRD patterns of BiO-NCs at room temperature in comparison to the calculated patterns generated from the respective SC XRD data of (a) bulk **C-1:Gd** (SC XRD, T = 100 K) (b) bulk **C-1:Dy** (SC XRD, T = 100 K). The respective patterns are in good agreement. Small differences in the patterns are caused by the different temperatures applied performing the measurements (PXRD: T = 298 K, SC XRD: T = 100 K) and partial loss of packing solvent during grinding the crystals for PXRD studies.

Crystal Structure of **C-2$_E$** and **C-2$_E$:Gd:**

Table S 23. SC-XRD crystallographic data for the Gd^{3+} doped BiO-NC, $[Bi_{38}O_{45}(OMc)_{24}(EtOH)_{14}]$:Gd (**C-2$_E$:Gd**), $[Bi_{38}O_{45}(OMc)_{24}(EtOH)_{14}]$ (**C-2$_E$**).[8]

	**C-2$_E$:Gd**	**C-2$_E$**
Empirical formula	$Bi_{33.24}Gd_{4.76}O_{107}C_{124}H_{202}$	$Bi_{38}O_{107}C_{124}H_{202}$
M / g·mol^{-1}	11096.80	11346.08
T / K	100	100(2)
λ / Å	0.71073	0.71073
Space group	$P\bar{1}$	$P\bar{1}$
a / Å	16.9763(7)	16.9833(11)
b / Å	18.9629(8)	18.9862(11)
c / Å	20.1879(8)	20.1839(13)
α / °	111.274(2)	111.157(2)
β / °	111.424(2)	111.443(2)
γ / °	100.614(2)	100.725(2)
V / Å^3	5248.4(4)	5256.6(6)
Z	1	1
$D_{calc.}$ / g·cm^{-3}	3.511	3.584
μ / mm^{-1}	29.283	31.759
F(000)	4864	4956
Reflections measured	479569	601223
Independent Reflections	26629	22908
$R_{Int.}$	0.0515	0.0599
GOF on F^2	1.059	1.112
Final R indices [I>2σ(I)]	R_1 = 0.0398, wR_2 = 0.1101	R_1 = 0.0234, wR_2 = 0.0537
R indices (all data)	R_1 = 0.0428, wR_2 = 0.1127	R_1 = 0.0260, wR_2 = 0.0546

Significance test of Gd^{3+} doping in **C-2$_E$:Gd:** As described above the gadolinium occupancy was compared with regard to its significance (Table S 24). This determination lead to the formula $[Bi_{34.75(11)}Gd_{3.25(11)}O_{107}C_{124}H_{202}]$ for **C-2$_E$:Gd.** Noteworthy, at several Bi positions the determined values are slightly above the 3σ criteria in the case of **C-2$_E$:Gd**, thus consequently leading to a higher "crystallographic" doping content. However, the occupancy with Gd^{3+} of the inner $\{Bi_6O_9\}$ fragment is largely preferred. Please note that **C-2$_E$:Gd** was prepared starting from **C-1:Gd**, which shows mainly, but not exclusive, inner core occupancy of Gd. While reorganization upon exchange of the ligand periphery in solution might lead to reorganization of the metal oxido core and thus differences in occupation of bismuth positions, we assume that crystal choice and quality is more likely. Work on the mechanism of cluster formation is in progress.

Table S 24. Evaluation of the significant occupation of atom positions by gadolinium using the difference in Gd amount of **C-2$_E$:Gd** and **C-2$_E$(:Gd)** (form theoretical doping of **C-2$_E$** with Gd) with respect to the 3σ criteria. In the case that the 3σ value is higher than the difference in the Gd amount, it is considered to be zero.

atom	**C-2$_E$:Gd** occupancy in %		**C-2$_E$(:Gd)** occupancy in %		difference Gd in %	Standard derivation $\sigma = \sqrt{\Delta(1)^2 + \Delta(2)^2}$	3σ
	Bi	Gd	Bi	Gd			
Bi1	0.9252(144)	0.0748(144)	0.9737(93)	0.0263(93)	0.049	0.0171	**0.051**
Bi2	0.9414(147)	0.0586(147)	0.9773(93)	0.0227(93)	0.036	0.0174	**0.052**
Bi3	0.8484(144)	0.1516(144)	0.9605(93)	0.0395(93)	**0.112**	0.0172	0.052
Bi4	0.7245(144)	0.2754(144)	0.9307(94)	0.0693(94)	**0.206**	0.0172	0.051
Bi5	0.9151(145)	0.0849(145)	0.9789(94)	0.0211(94)	**0.064**	0.0172	0.052
Bi6	0.6631(142)	0.3369(142)	0.9673(95)	0.0327(95)	**0.304**	0.0171	0.051
Bi7	0.9264(145)	0.0736(145)	0.9824(94)	0.0176(94)	**0.056**	0.0173	0.052
Bi8	0.9122(144(	0.0878(144)	0.9764(94)	0.0236(94)	**0.064**	0.0172	0.052
Bi9	0.8371(143)	0.1629(143)	0.9513(93)	0.0487(93)	**0.114**	0.0171	0.051
Bi10	0.8543(143)	0.1457(143)	0.9624(94)	0.0376(94)	**0.108**	0.0171	0.051
Bi11	0.8562(143)	0.1438(143)	0.9563(93)	0.0437(93)	**0.100**	0.0170	0.051
Bi12	0.9067(143)	0.0933(143)	0.9704(93)	0.0296(93)	**0.064**	0.0171	0.051
Bi13	0.7707(144)	0.2293(144)	0.9691(94)	0.0309(94)	**0.198**	0.0172	0.052
Bi14	0.9073(143)	0.0927(143)	0.9781(93)	0.0219(93)	**0.071**	0.0171	0.051
Bi15	0.9172(145)	0.0828(145)	0.9697(93)	0.0303(93)	**0.053**	0.0172	0.052
Bi16	0.9174(145)	0.0826(145)	0.9746(94)	0.0254(93)	**0.057**	0.0173	0.052
Bi17	0.9231(145)	0.0769(143)	0.9725(93)	0.0275(93)	0.049	0.0171	**0.051**
Bi18	0.9305(145)	0.0695(145)	0.9839(94)	0.0161(94)	**0.053**	0.0173	0.052
Bi19	0.9452(144)	0.0558(144)	0.9813(94)	0.0187(93)	0.036	0.0172	**0.052**
					**∑ 1.625**		

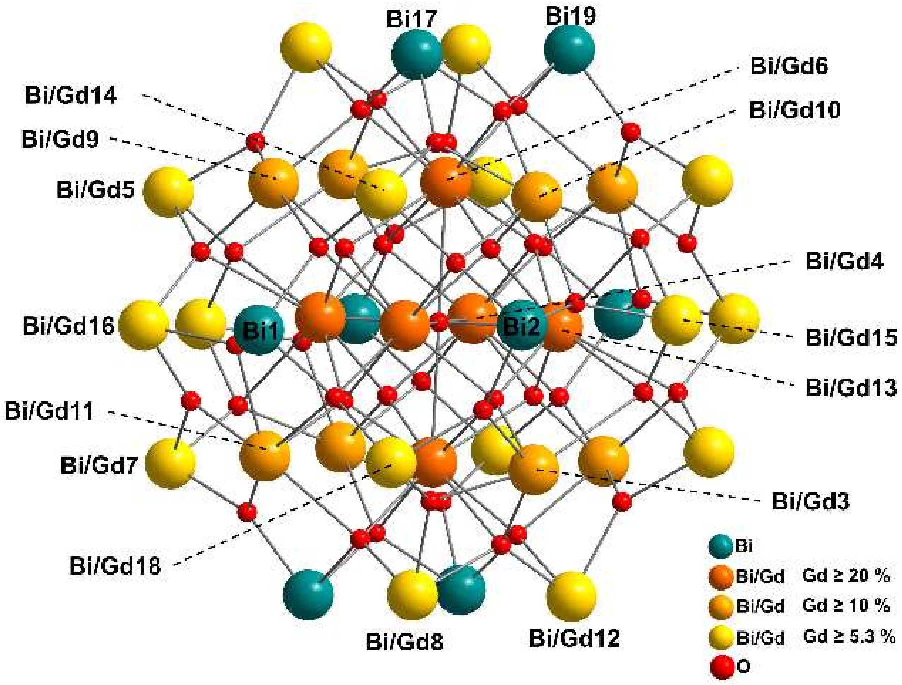

Figure S 53. "Ball-and-Stick" model of a part of BiO-NC **C-2$_E$:Gd** showing crystallographic different Bi or Bi/Gd positions including the atom labels and different colors based on the Gd occupancy ranges. Gd occupancy is high in the inner {Bi/Gd_6O_9} fragment and decreasing towards the outer positions, exact calculated occupancies are not mentioned for clarity but can be viewed in (Table S 24).

The cluster core structures of **C-2$_E$:Gd** and of **C-2$_E$** show the same connectivity and generally similar M–O bond distances with 2.069(5) Å–2.903(11) Å (**C-2$_E$:Gd**) and 2.065(5) Å–3.055(5) Å (**C-2$_E$**). Differences occur not as result of the doping but slight differences at

individual positions. All methacrylate and ethanol ligands show the same coordination modes in both clusters and differ only minor in the respective bond distances (*cf.* Table S 25).

Table S 25. Summary of the Bi–O distance (*d*) range for different coordination modes of Bi **C-2$_E$**, **C-2$_E$:Gd**.

coordination mode	**C-2$_E$:Gd**		**C-2$_E$**	
	number	*d* / Å	number	*d* / Å
**oxido**				
μ3-1κ*O*:2κ*O*:3κ*O*	16	[2.099(6)–2.421(6)]	16	[2.091(5)–2.417(5)]
μ4-1κ*O*:2κ*O*:3κ*O*:4κ*O*	28	[2.069(5)–2.903(11)]	28	[2.065(5)–3.055(5)]
μ6-1κ*O*:2κ*O*:3κ*O*:4κ*O*:5κ*O*:6κ*O*	1	[2.4430(4)–2.720']	1	[2.4281(3)–2.8133(3)]
**EtOH**				
1κ*O*	8	[2.748(7)–2.983(10)]	8	[2.737(6)–2.977(7)]
μ2-1κ*O*:2κ*O*	6	[2.738(7)–3.056(6))]	6	[2.739(6)–3.072(6)]
non-coordinated				>3.15
**methacrylato**				
μ2-1κ*O*:2κ*O*	6	[2.490(6)–2.816(7)]	6	[2.489(6)–2.804(6)]
μ2-1κ*O*,*O*':2κ*O*'	4	[2.390(8)–3.017(8)]	4	[2.386(8)–3.021(7)]
μ3-1κ*O*:2κ*O*':3κ*O*'	4	[2.638(10)–3.057(7)]	4	[2.625(8)–3.043(6)]
μ3-1κ*O*:2κ*O*:3κ*O*,*O*'	0	-	0	-
μ3-1κ*O*:2κ*O*:2κ*O*':3κ*O*'	4	[2.411(6)–3.127(7)]	4	[2.398(5)–3.148(6)]
μ4-1κ*O*:2κ*O*:3κ*O*':4κ*O*'	4	[2.556(6)–2.912(6)]	4	[2.556(6)–2.899(6)]
μ4-1κ*O*:2κ*O*: 3κ*O*:3κ*O*':4κ*O*'	2	[2.707(6)–3.053(7)]	2	[2.695(6)–3.088(6)]

UV–vis Studies and Transition Assignment:

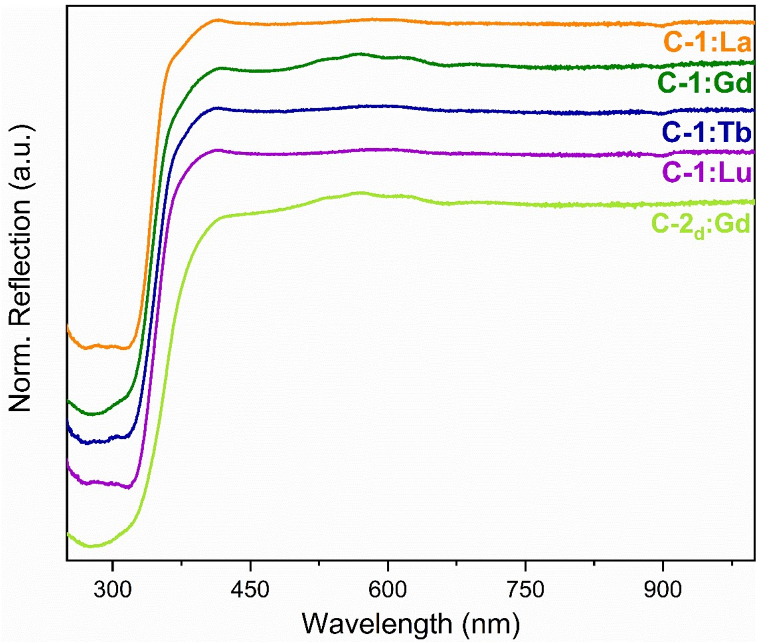

Figure S 54. UV–vis diffuse reflection spectra (DRS) of lanthanide doped BiO-NCs **C-1:Ln** (Ln = La, Gd, Tb and Lu) and **C-2$_d$:Gd**.

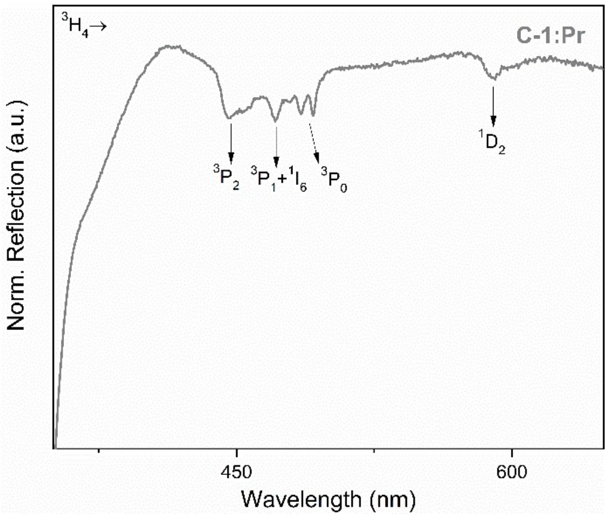

Figure S 55. Enlarged part of the UV–vis DRS spectra of Pr^{3+} doped BiO-NCs with assigned optical absorption transitions from Pr^{3+} with the ground state $^3H_4$ to the respective excited states.

Table S 26. Summary of the optical absorption bands in the Pr^{3+} doped BiO-NC, with assigned transitions from the ground state $^3H_4$ respective to the excited states.[13-14]

Cluster	→ $^3P_2$	→ $^3P_1$+$^1I_6$	→ $^3P_1$	→ $^1D_2$
**C-1:Pr**	446 nm	471 nm	486 nm + 492 nm	590 nm

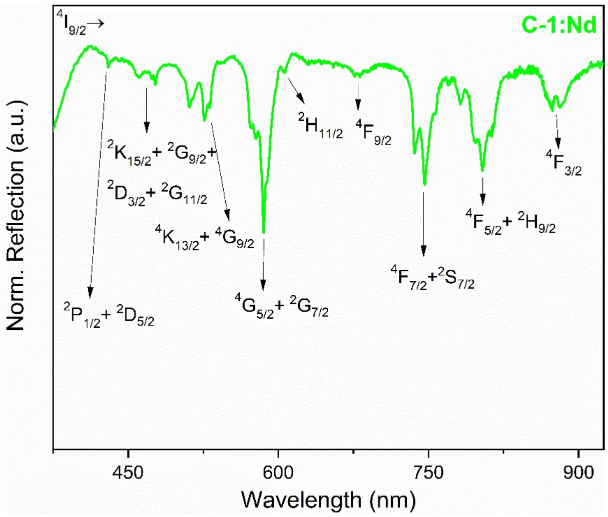

Figure S 56. Enlarged part of the UV–vis DRS spectra of Nd^{3+} doped BiO-NCs with assigned optical absorption transitions from Nd^{3+} with the ground state $^4I_{9/2}$ to the respective excited states.

Table S 27. Summary of the optical absorption bands in the Nd^{3+} doped BiO-NC, with assigned transitions from the ground state $^4I_{9/2}$ respective to the excited states.[14-15]

Cluster	→ $^2P_{1/2}$ +$^2D_{5/2}$	→ $^2K_{15/2}$+$^2G_{9/2}$ +$^2D_{3/2}$+$^2G_{11/2}$	→ $^4K_{13/2}$+ $^4G_{9/2}$	→ $^4G_{5/2}$+ $^2G_{7/2}$	→ $^2H_{11/2}$	→ $^4F_{9/2}$	→ $^4F_{7/2}$ +$^2S_{7/2}$
**C-1:Nd**	430 nm	461 nm<br>476 nm	526 nm<br>510 nm	585 nm	606 nm	680 nm	736 nm<br>736 nm
**Cluster**	→ $^4F_{5/2}$ +$^2H_{9/2}$	→ $^4F_{3/2}$					
**C-1:Nd**	803 nm	881 nm<br>874 nm					

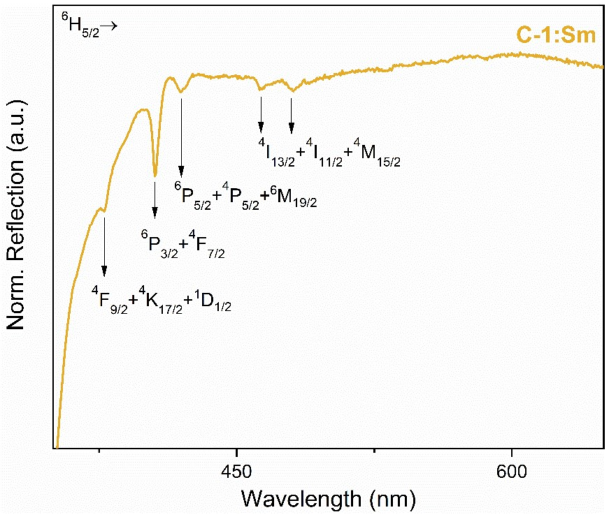

Figure S 57. Enlarged part of the UV–vis DRS spectra of Sm^{3+} doped BiO-NCs with assigned optical absorption transitions from Sm^{3+} with the ground state $^6H_{5/2}$ to the respective excited states.

Table S 28. Summary of the optical absorption bands in the Sm^{3+} doped BiO-NC, with assigned transitions from the ground state $^6H_{5/2}$ respective to the excited states.[16]

**Cluster**	$\rightarrow$ $^4F_{9/2}+^4K_{17/2}+^1D_{1/2}$	$\rightarrow$ $^6P_{3/2}+^4F_{7/2}$	$\rightarrow$ $^6P_{5/2}+^4P_{5/2}+^6M_{19/2}$	$\rightarrow$ $^4I_{13/2}+^4I_{11/2}+^4M_{15/2}$
**C-1:Sm**	378 nm	405 nm	419 nm	463 nm +480 nm

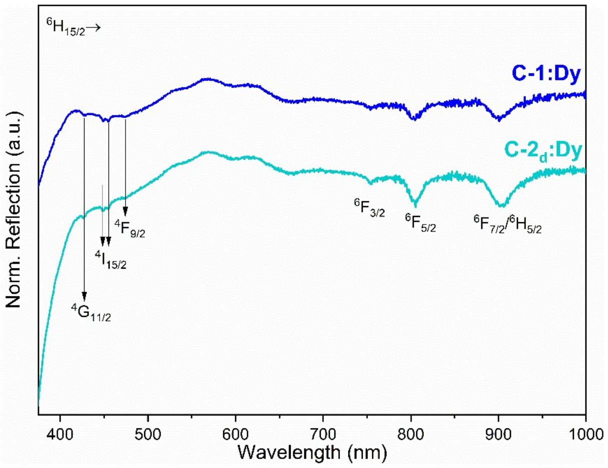

Figure S 58. Enlarged part of the UV–vis DRS spectra of Dy^{3+} doped BiO-NCs with assigned optical absorption transitions from Dy^{3+} with the ground state $^6H_{15/2}$ to the respective excited states.

Table S 29. Summary of the optical absorption bands in the Dy^{3+} doped BiO-NCs, with assigned transitions from the ground state $^6H_{15/2}$ respective to the excited states

**BiO-NC**	$\rightarrow$ $^4G_{11/2}$	$\rightarrow$ $^4I_{15/2}$	$\rightarrow$ $^4F_{9/2}$	$\rightarrow$ $^6F_{3/2}$	$\rightarrow$ $^6F_{5/2}$	$\rightarrow$ $^6F_{7/2}+^6H_{5/2}$
**C-1:Dy**	426 nm	454 nm<br>448 nm	473 nm	754 nm	805 nm	901 nm
**C-2$_d$:Dy**	426 nm	449 nm<br>455 nm	474 nm	754 nm	805 nm	906 nm

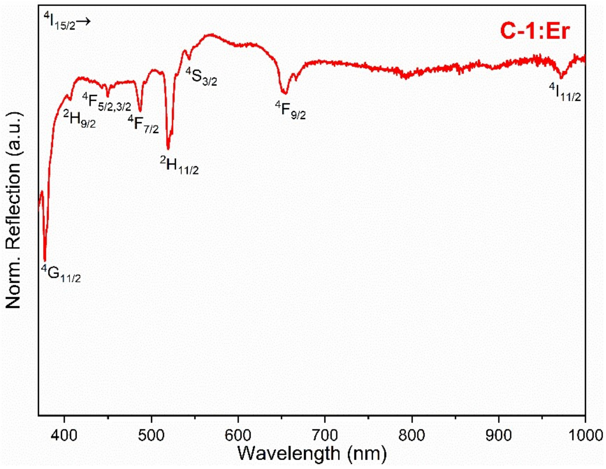

Figure S 59. Enlarged part of the UV–vis DRS spectra of the Er^{3+} doped BiO-NC **C-1:Er** with assigned optical absorption transitions from Er^{3+} with the ground state $^4I_{15/2}$ to the respective excited states.

Table S 30. Summary of the optical absorption bands in the Er^{3+} doped BiO-NC, with assigned transitions from the ground state $^4I_{15/2}$ respective to the excited states.[17]

Cluster	→ $^4G_{11/2}$	→ $^2H_{9/2}$	→ $^4F_{5/2,3/2}$	→ $^4F_{7/2}$	→ $^2H_{11/2}$	→ $^4S_{3/2}$	→ $^4F_{9/2}$	→ $^4I_{11/2}$
**C-1:Er**	377 nm	407 nm	450 nm	487 nm	530 nm<br>523 nm<br>519 nm	543 nm	655 nm<br>666 nm	971 nm

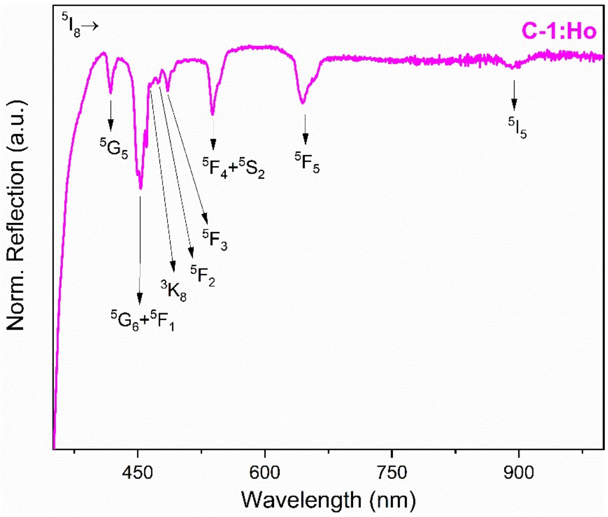

Figure S 60. Enlarged part of the UV–vis DRS spectra of the Ho^{3+} doped BiO-NC **C-1:Ho** with assigned optical absorption transitions from Ho^{3+} with the ground state $^4I_8$ to the respective excited states.

Table S 31. Summary of the optical absorption bands in the Ho^{3+} doped BiO-NC, with assigned transitions from the ground state $^4I_8$ respective to the excited states.[18-19]

Cluster	→ $^5G_5$	→ $^5G_6$+$^5F_1$	→ $^3K_8$	→ $^5F_2$	→ $^5F_3$	→ $^5F_4$+ $^5S_2$	→ $^5F_5$	→ $^5I_5$
**C-1:Ho**	418 nm	453 nm	465 nm	474 nm	485 nm	538 nm	644 n m	891 nm

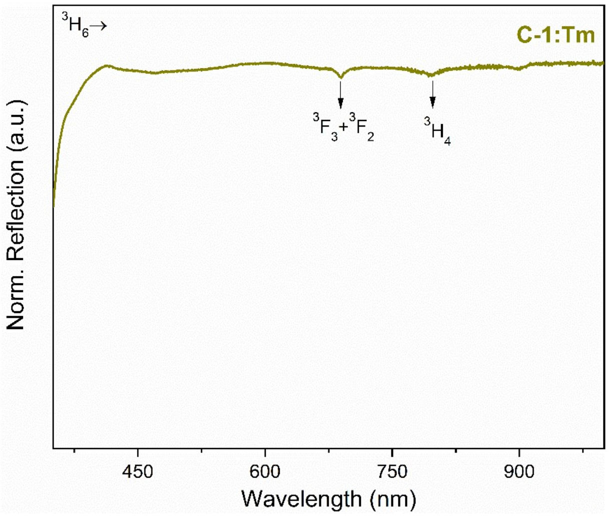

Figure S 61. Enlarged part of the UV–vis DRS spectra of the Tm^{3+} doped BiO-NC **C-1:Tm** with assigned optical absorption transitions from Tm^{3+} with the ground state $^3H_6$ to the respective excited states.

Table S 32. Summary of the optical absorption bands in the Tm^{3+} doped BiO-NC, with assigned transitions from the ground state $^3H_6$ respective to the excited states.[20]

**Cluster**	$\rightarrow$ $^3F_3+^3F_2$	$\rightarrow$ $^3H_4$
**C-1:Tm**	689 nm	797 nm

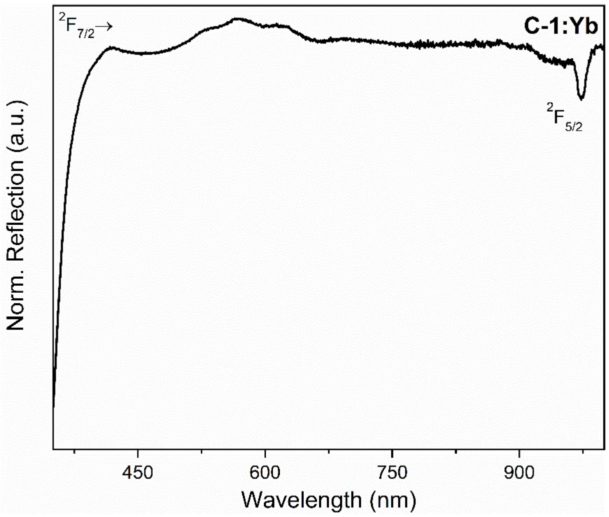

Figure S 62. Enlarged part of the UV–vis DRS spectra of the Yb^{3+} doped BiO-NC **C-1:Yb** with assigned optical absorption transitions from Yb^{3+} with the ground state $^2F_{7/2}$ to the respective excited states.

Table S 33. Summary of the optical absorption bands in the Yb^{3+} doped BiO-NC, with assigned transitions from the ground state $^2F_{7/2}$ respective to the excited states. [1]

**Cluster**	$\rightarrow$ $^2F_{5/2}$
**C-1:Yb**	950 nm + 875 nm

PL Studies of **C-1:Ln** at ambient conditions:

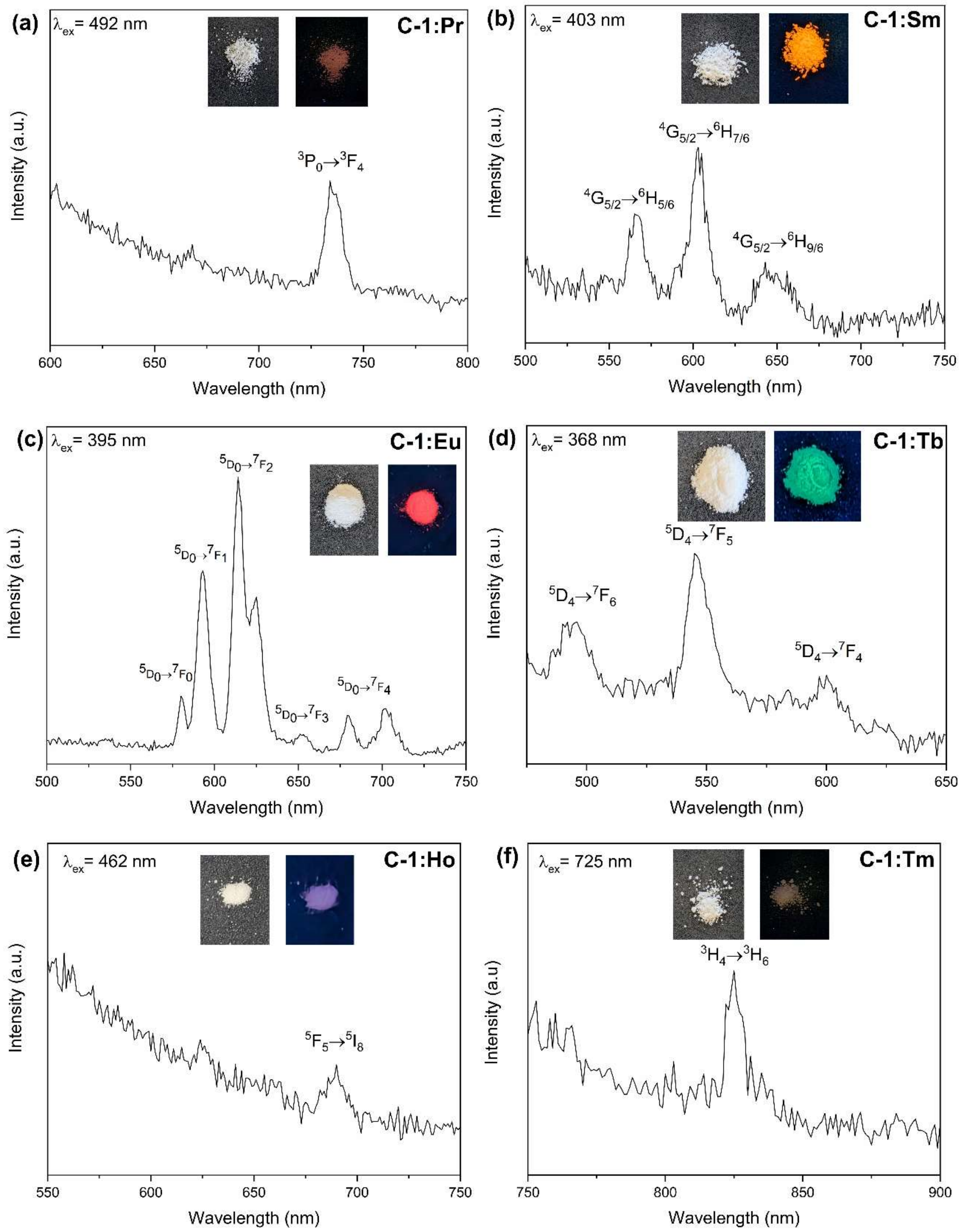

Figure S 63. PL emission spectra of (a) the Pr^{3+} doped BiO-NC **C-1:Pr** under excitation with 492 nm, (b) the Sm^{3+} doped BiO-NC **C-1:Sm** under excitation with 403 nm, (c) the Eu^{3+} doped BiO-NC **C-1:Eu** under excitation with 395 nm (d) the Tb^{3+} doped BiO-NC **C-1:Tb** under excitation with 368 nm, (e) the Ho^{3+} doped BiO-NC **C-1:Ho** under excitation with 462 nm and (f) the Tm^{3+} doped BiO-NC **C-1:Tm** under excitation with 725 nm. Photos of the respective BiO-NCs under daylight (left) and under UV light (right, 256 nm) are included for visualization.

Please note that the PL results for the Eu^{3+} doped BiO-NCs as well as for the undoped BiO-NCs were recently discussed in detail elsewhere.[8]

PL Studies of **C-1:Gd**:

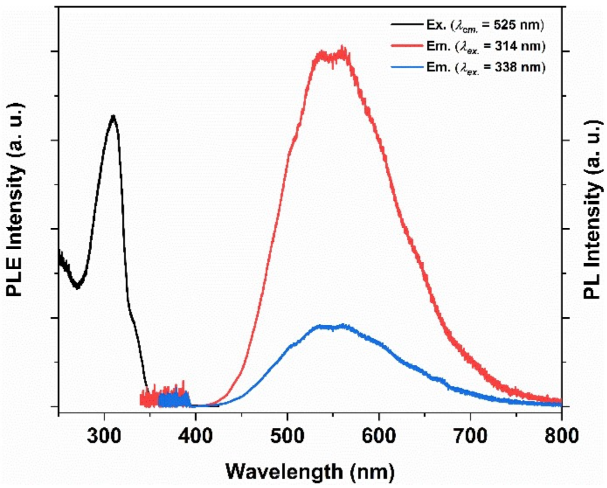

Figure S 64. PLE and PL studies of Gd^{3+}-doped cluster of **C-1:Gd**.

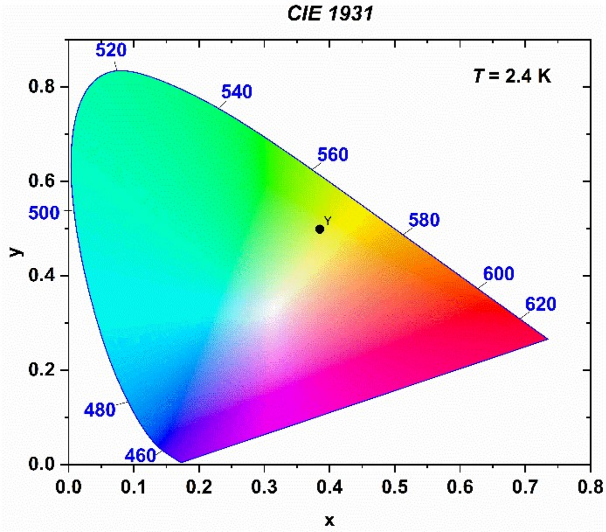

Figure S 65. CIE coordinates X = 0.3850 and Y = 0.4985 of Gd doped **C-1:Gd** obtained for the emission at 2.4 K under excitation at 314 nm.

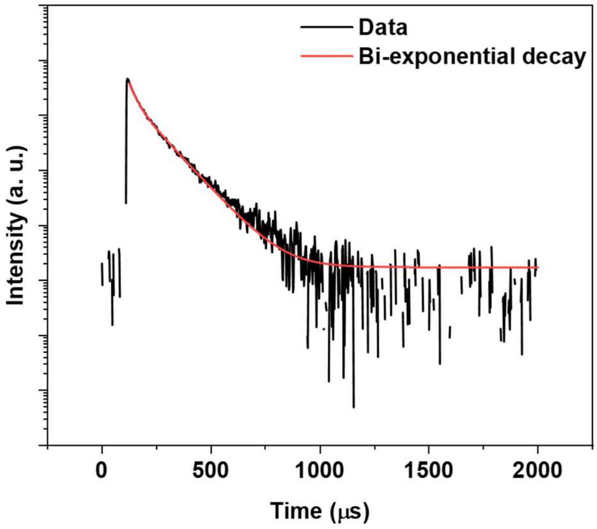

Figure S 66 Lifetime study of **C-1:Gd** at 2.4 K**.** The sample was excited at 314 nm and emission was monitored at 540 nm**.**

Table S 34. PL lifetimes of BiO-NC **C-1:Gd** measured at 2.4 K at 314 nm and emission was monitored at 540 nm**.**

BiO-NC	Lifetimes (τ/µs)	
	τ_1	τ_2
**C-1:Gd**	29 ± 1	102± 1

PL Studies of **C-1:Er**:

Table S 35. Assignments of transitions observed in the PLE spectrum (Figure 5) of the BiO-NC **C-1:Er** at 2.4 K.

Wavelength	Transition	Region	Notes
**366 nm**	$^4G_{9/2} \leftarrow {}^4I_{15/2}$	UV	High-lying band; weak parity-forbidden emission
**381 nm**	$^4G_{11/2} \leftarrow {}^4I_{15/2}$	Near-UV	Violet Er^{3+} emission
**407 nm**	$^2H_{9/2} \leftarrow {}^4I_{15/2}$	Violet	Distinct high-energy line
**450 nm**	$^4F_{5/2}/{}^4/F_{3/2} \leftarrow {}^4I_{15/2}$	Blue	Composite blue band
**470 nm**	$^4F_{7/2} \leftarrow {}^4I_{15/2}$	Blue	Often appears as a shoulder or distinct band in Er^{3+} spectra
**484 nm**	$^4F_{7/2} \leftarrow {}^4I_{15/2}$	Blue-cyan	Stronger blue emission; overlaps with 470 nm manifold
**523 nm**	$^2H_{11/2} \leftarrow {}^4I_{15/2}$	Green	Thermally coupled “hot band”
**544 nm**	$^4S_{3/2} \leftarrow {}^4I_{15/2}$	Green	Canonical Er^{3+} green emission
**655 nm**	$^4F_{9/2} \leftarrow {}^4I_{15/2}$	Red	Red Er^{3+} emission
**977 nm**	$^4I_{11/2} \leftarrow {}^4I_{15/2}$	NIR	Strong ~980 nm band; telecom pumping level

Table S 36. Summary of the photoluminescence emission bands in the Er^{3+} doped BiO-NC **C-1:Er**, with assigned transitions.

**Cluster**	$^2H_{11/2} \rightarrow {}^4I_{15/2}$	$^4F_{9/2} \rightarrow {}^4I_{15/2}$	$^4F_{9/2} \rightarrow {}^4I_{15/2}$	$^4I_{13/2} \rightarrow {}^4I_{15/2}$
**C-1:Er**	520–535 nm	550–570 nm	645–660 nm	1500–1650 nm

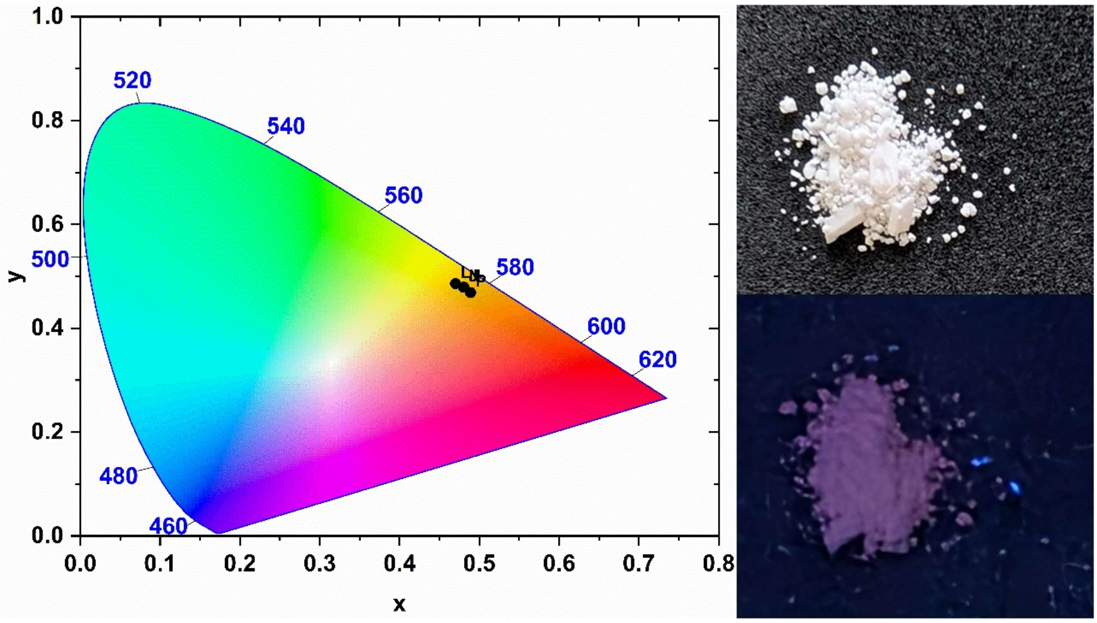

Figure S 67. CIE 1931 coordinates of Er doped **C-1:Er** at different temperatures 2.4 K (J), 77 K (L), 180 K (N) and 300 K (P) obtained upon excitation at 310 nm. CIE coordinates (black dot in the figure) are J, X = 0.481 and Y = 0.479; L, X = 0.470 and Y = 0.486; N, X = 0.480 and Y = 0.479; P, X = 0.489 and Y = 0.469. Images of the erbium doped BiO-NC under ambient condition and irradiation with daylight (top) and under UV light (256 nm, bottom) are included for visualization.

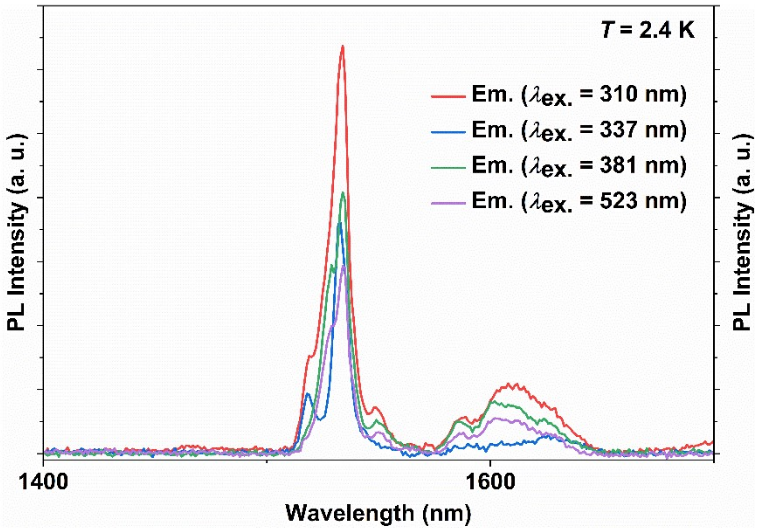

Figure S 68. Excitation wavelength dependent PL spectra of BiO-NC **C-1:Er** at 2.4 K.

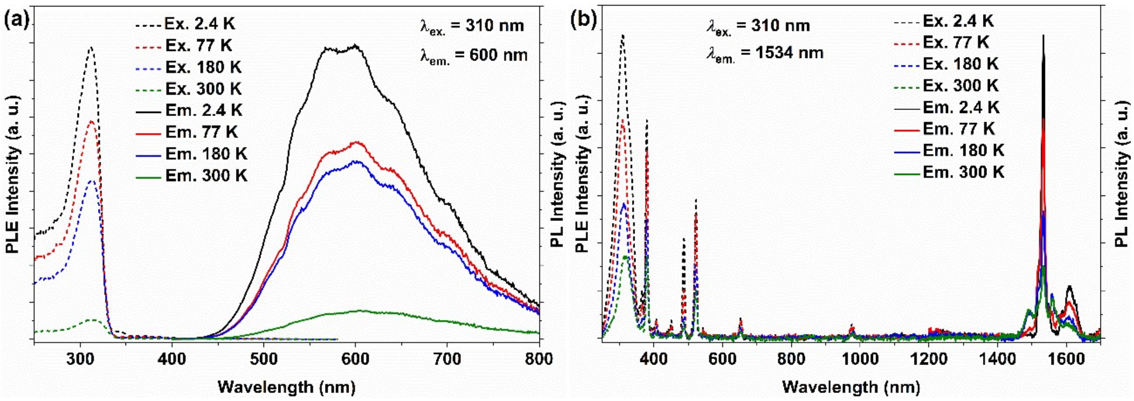

Figure S 69. Temperature-dependent PLE and PL spectra of BiO-NC **C-1:Er**, (a) in the visible light range at an emission wavelength of 600 nm and (b) in the NIR light range at an emission wavelength of 1534 nm, all under excitation with 310 nm.

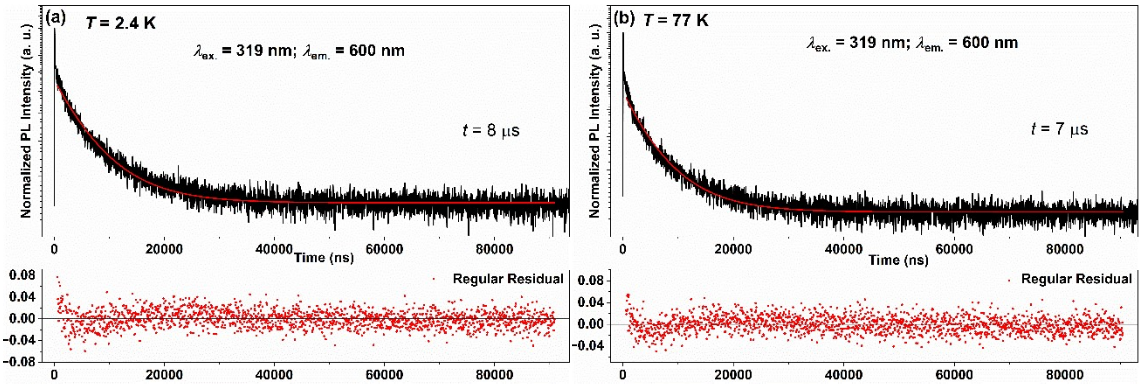

Figure S 70. Decay profiles using TCSPC method form **C-1:Er** obtained by monitoring emission at 600 nm under excitation with 319 nm (LED) at (a) 2.4 K an (b) 77 K. The profiles can be satisfactorily fitted with a mono-exponential function yielding lifetimes in the few μs regime.

Table S 37. PL lifetimes of BiO-NC **C-1:Er** measured at different temperatures excited with 319 nm and emission was monitored at 600 nm**.**

Temperature	Lifetimes (τ/μs)
	τ_1
2.4 K	8 ± 1
77 K	7 ± 1

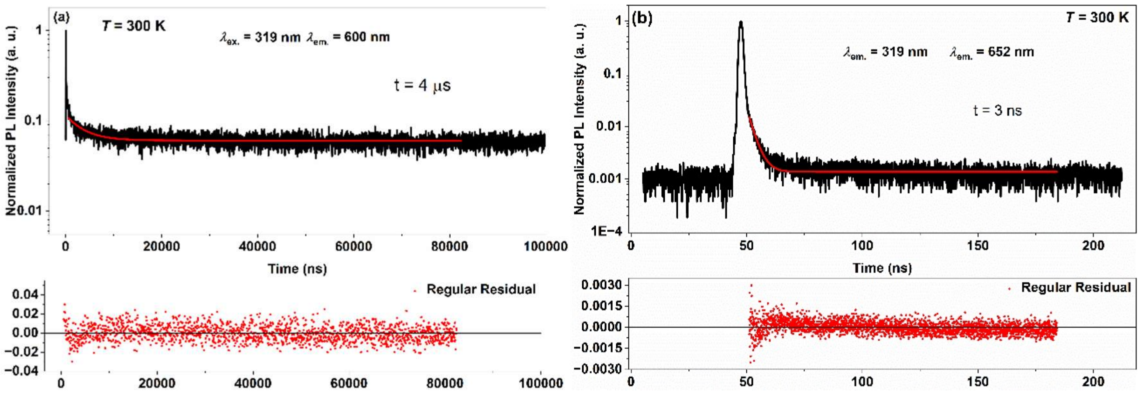

Figure S 71. Decay profiles using TCSPC method form **C-1:Er** obtained by monitoring emission at 600 nm and 652 nm at 300 K. The profiles can be satisfactorily fitted with a mono-exponential function yielding lifetimes in the few ns regime.

Table S 38. PL lifetimes of BiO-NC **C-1:Er** measured at 300 K under excitation with 319 nm monitored at different wavelength**.**

Wavelength	Lifetimes (τ)
	τ_1
$\lambda_{em.}$ = 600 nm	4 ± 1 μs
$\lambda_{em.}$ = 652 nm	3 ± 1 ns

PL Studies of **C-1:Yb**:

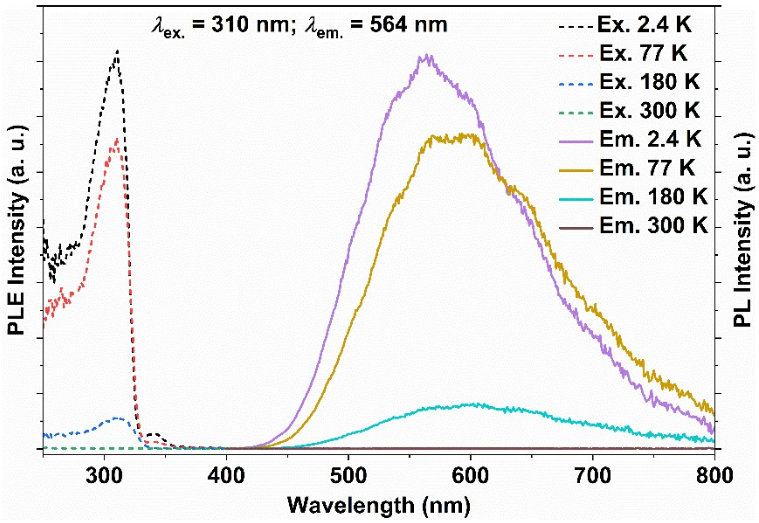

Figure S 72. (a) Temperature dependent PL emission spectra (solid line) under excitation with $\lambda_{ex.}$ = 310 nm, and PL excitation spectra (dotted lines) monitored at $\lambda_{em.}$ = 562 nm of solid cluster **C-1:Yb** at different temperatures 2.4 K, 77 K, 180 K and 300 K.

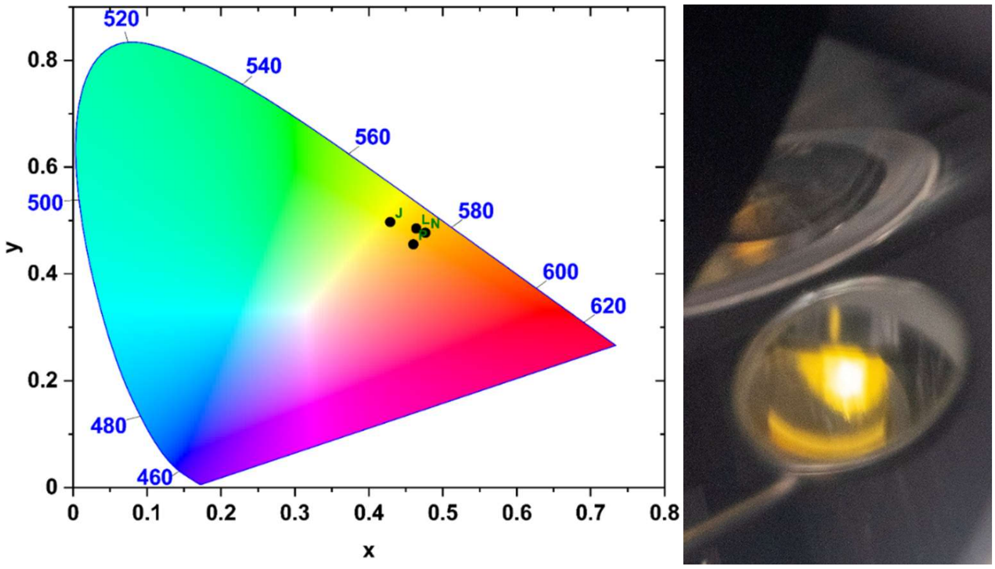

Figure S 73. (Left) CIE coordinates of Yb doped **C-1:Yb** at different temperatures 2.4 K (J), 77 K (L), 180 K (N) and 300 K (P) obtained upon excitation at 310 nm. CIE coordinates (black dot in the figure) are J, X = 0.429 and Y = 0.496; L, X = 0.464 and Y = 0.484; N, X = 0.477 and Y = 0.476; P, X = 0.461 and Y = 0.455. (right) Emission color of the Yb^{3+}-doped cluster at 2.4 K on excitation at 310 nm. Similar emission color is noted for the other clusters discussed in this study.

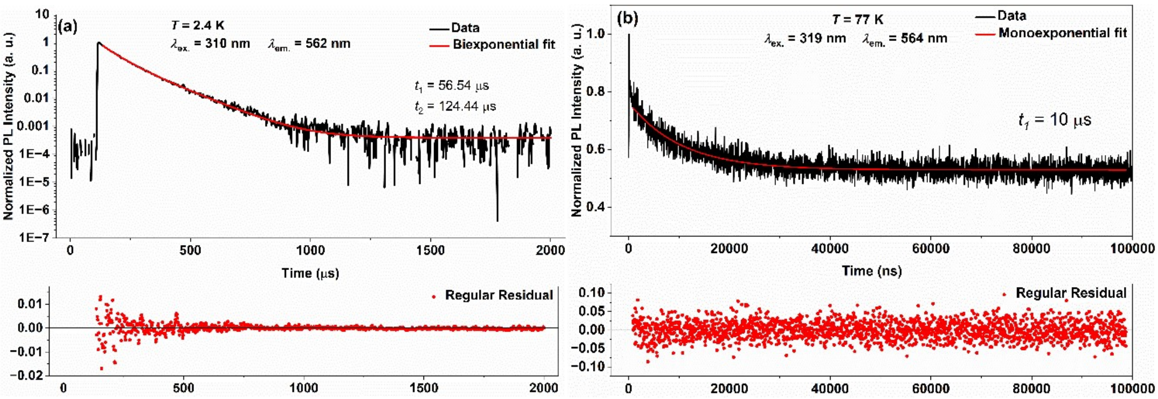

Figure S 74. (a) PL decay spectra of BiO-NC **C-1:Yb** at 2.4 K under excitation with $\lambda_{ex.}$ = 310 nm at an emission wavelength of $\lambda_{em.}$ = 564 nm with the respective biexponential fit. (b) PL decay spectra of BiO-NC **C-1:Yb** at 77 K under excitation with $\lambda_{ex.}$ = 319 nm (LED) at an emission wavelength of $\lambda_{em.}$ = 564 nm with the respective monoexponential fit.

Table S 39. PL lifetimes of BiO-NC **C-1:Yb** measured at 300 K under excitation with 325 nm monitored at different wavelength**.**

Wavelength	Lifetimes (τ/µs)	
	τ_1	τ_2
$\lambda_{ex.}$ = 310 nm	57 ± 2	124 ± 3
$\lambda_{ex.}$ = 319 nm	10 ± 1	

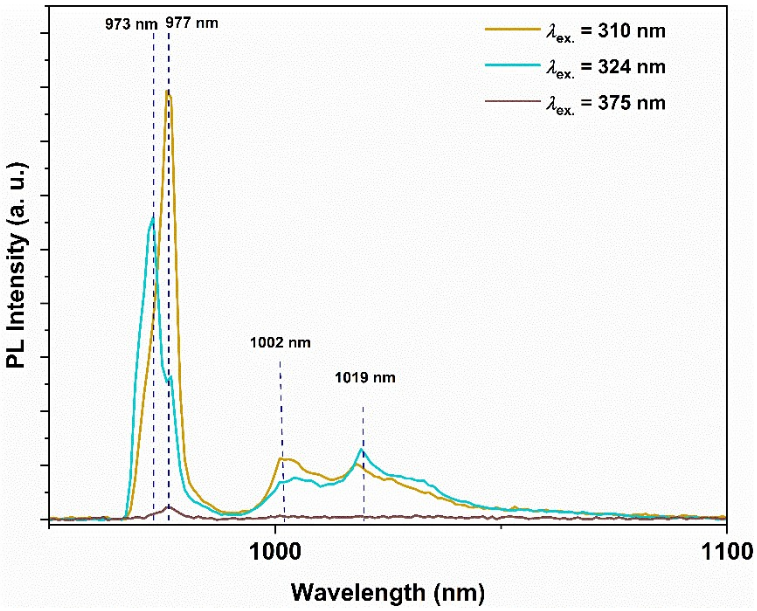

Figure S 75. Excitation wavelength dependent PL emission spectra of **C-1:Yb** at 2.4 K.

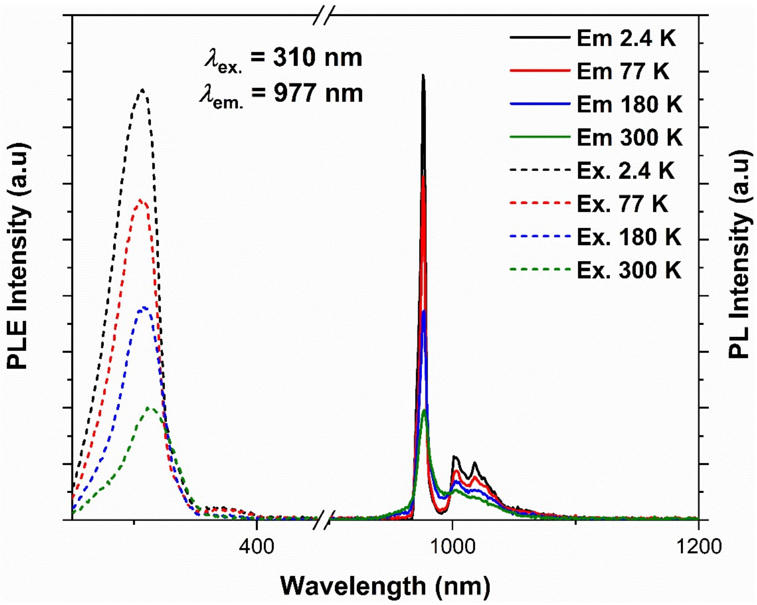

Figure S 76. Temperature dependence of PLE monitored at $\lambda_{em.}$ = 977 nm and PL under excitation at $\lambda_{ex.}$ = 310 nm of **C-1:Yb** at different temperatures.

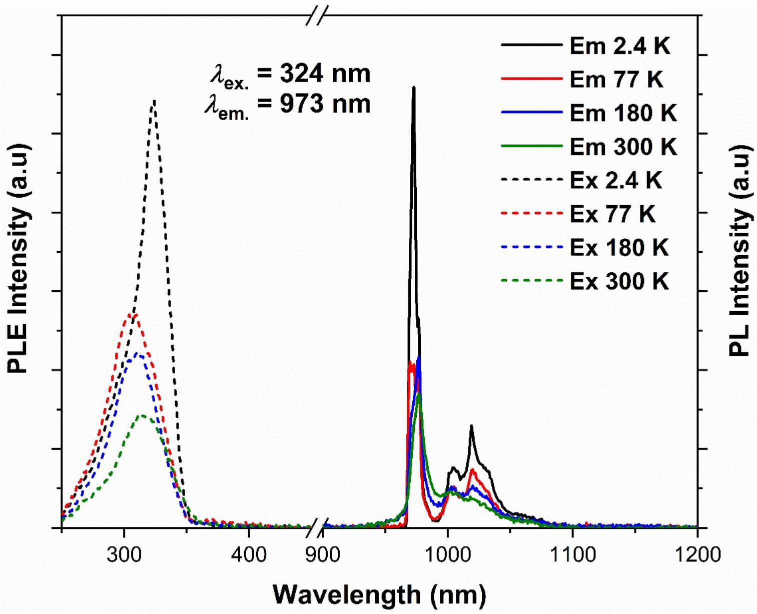

Figure S 77. Temperature dependence of PLE monitored at $\lambda_{em.}$ = 973 nm and PL under excitation at $\lambda_{ex.}$ = 324 nm of **C-1:Yb** at different temperatures.

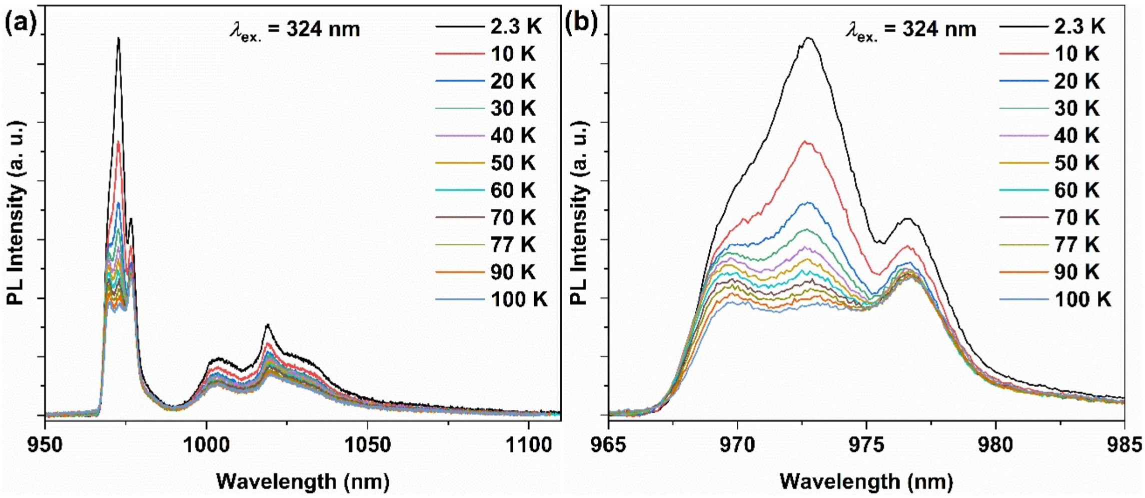

Figure S 78. PL emission spectra of BiO-NC **C-1:Yb** under excitation with $\lambda_{ex.}$ = 324 nm at different temperatures.

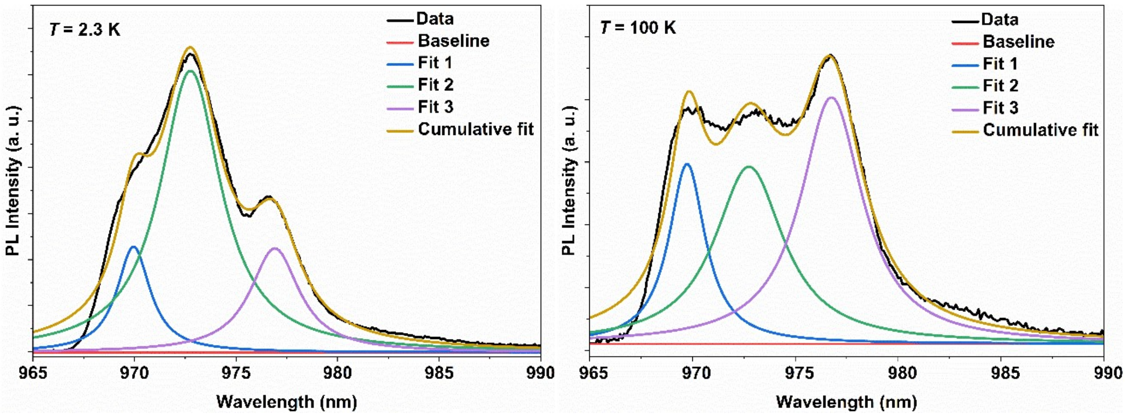

Figure S 79. Lorentz deconvoluted PL emission of **C-1:Yb** in the 965 nm to 990 nm range on excitation at 324 nm at T = 2.3 K (left) and at T = 100 K (right).

Table S 40. Integrated intensities and branching ratios associated with the J manifolds of the $^2F_{5/2} \rightarrow {}^2F_{7/2}$ transition at 2.4 K and 100 K.

Transition	2.4 K		100 K	
	Intensity	β_R	Intensity	β_R
$^2F_{5/2}(2) \rightarrow {}^2F_{7/2}$	1315184	13.91	884059	19.52
$^2F_{5/2}(1) \rightarrow {}^2F_{7/2}$	6217721	65.76	1590665	35.12
$^2F_{5/2}(0) \rightarrow {}^2F_{7/2}$	1921545	20.32	2053980	45.35

PL Studies of **C-2$_d$:Dy**:

Table S 41. Summary of the photoluminescence emission bands in the Dy^{3+} doped BiO-NC **C-2$_d$:Dy**, with assigned transitions from the excited state $^4F_{9/2}$ respective to the excited states.

**Cluster**	$\rightarrow {}^6H_{15/2}$	$\rightarrow {}^6H_{13/2}$	$\rightarrow {}^6H_{11/2}$	$\rightarrow {}^6H_{9/2} + {}^6F_{11/2}$
**C-2$_d$:Dy**	480 nm<br>485 nm	577 nm	667 nm	757 nm

Table S 42. Summary of the photoluminescence excitation bands in the Dy^{3+} doped BiO-NC **C-2$_d$:Dy**, with assigned transitions from the ground state $^6H_{15/2}$ respective to the excited states.

**Cluster**	$\rightarrow {}^6P_{7/2}$	$\rightarrow {}^6P_{7/2} + {}^4I_{11/2}$	$\rightarrow {}^4F_{7/2} + {}^4I_{13/2}$	$\rightarrow {}^4G_{11/2}$	$\rightarrow {}^4I_{15/2}$
**C-2$_d$:Dy**	348 nm	363 nm		425 nm	448 nm

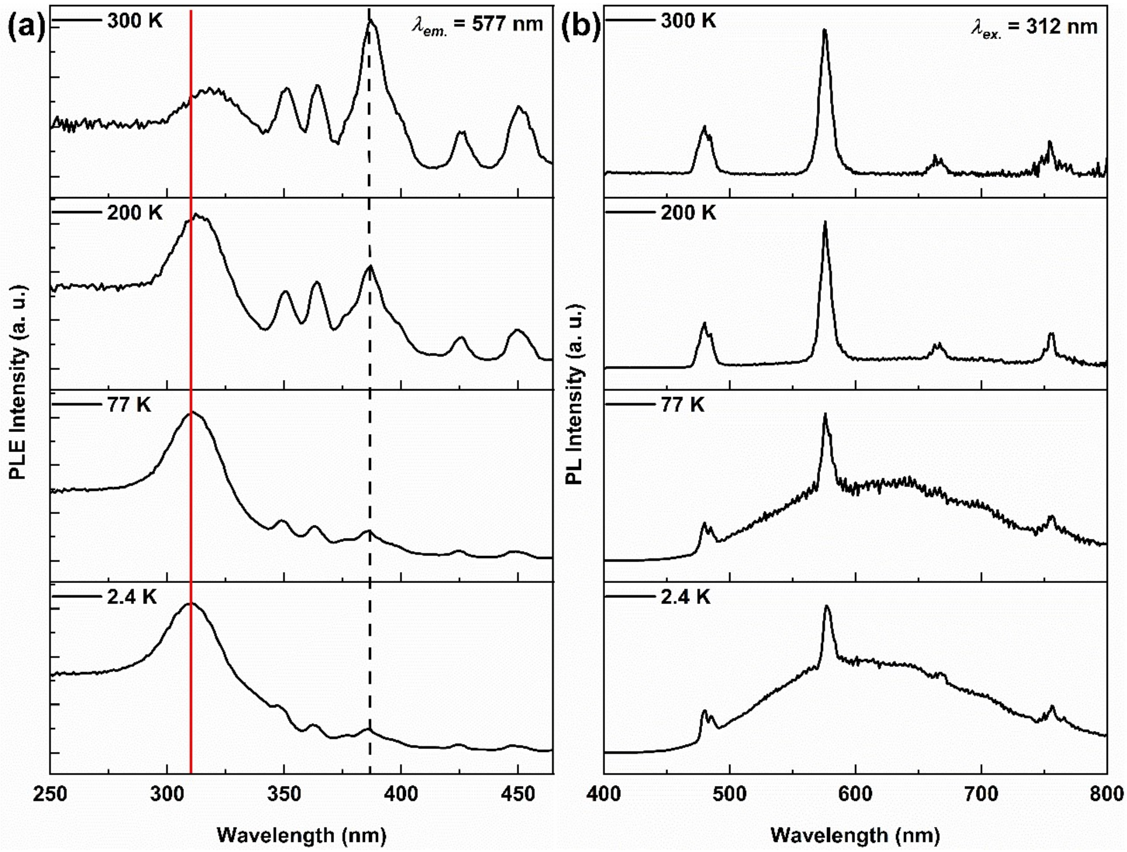

Figure S 80. Temperature-dependent (a) PLE spectra at $\lambda_{em.}$ = 577 nm and (b) PL spectra at $\lambda_{ex.}$ = 312 nm of **C-2$_d$:Dy**.

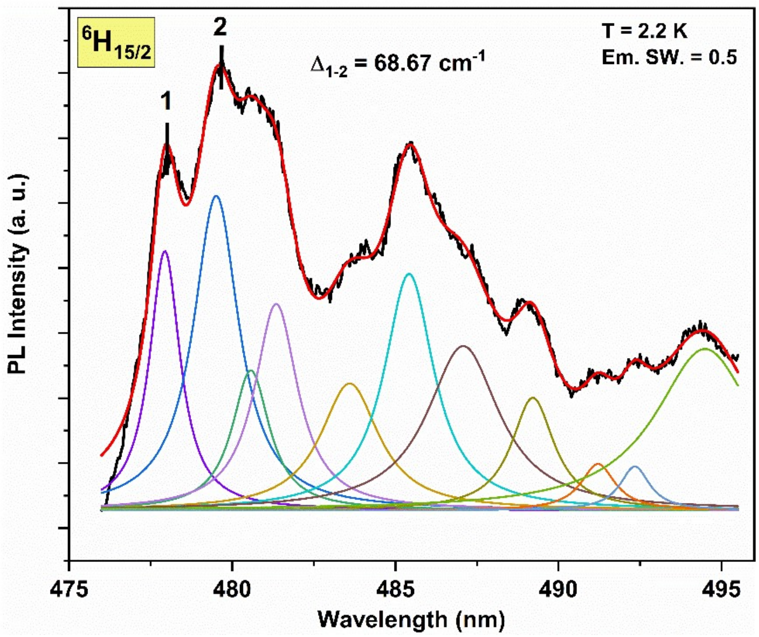

Figure S 81. Part of the emission spectra of **C-2$_d$:Dy** showing the $^6H_{15/2}$. The Peaks are fitted using Lorenz function to calculate the energy separation between the ground state and the first excited duplets.

For the $^4F_{9/2} \rightarrow {}^6H_{15/2}$ transition, eight peaks—J = 15/2; (2J + 1)/2—are expected considering the fact that Dy^{3+} is a Kramers ion. A satisfactory Lorentz deconvolution of the spectrum shown in Figure 84 requires eleven components indicating the mixing of hot bands with the $^4F_{9/2} \rightarrow {}^6H_{15/2}$ transition.[21] This prohibits the assignment of CF splitting between each J level. Nevertheless, by considering the first and second Lorentz components as ground (KD1) and first excited (KD2) doublets, we calculate 68.67 cm^{-1} as the energy difference ($\Delta_{1\text{-}2}$) between the levels.

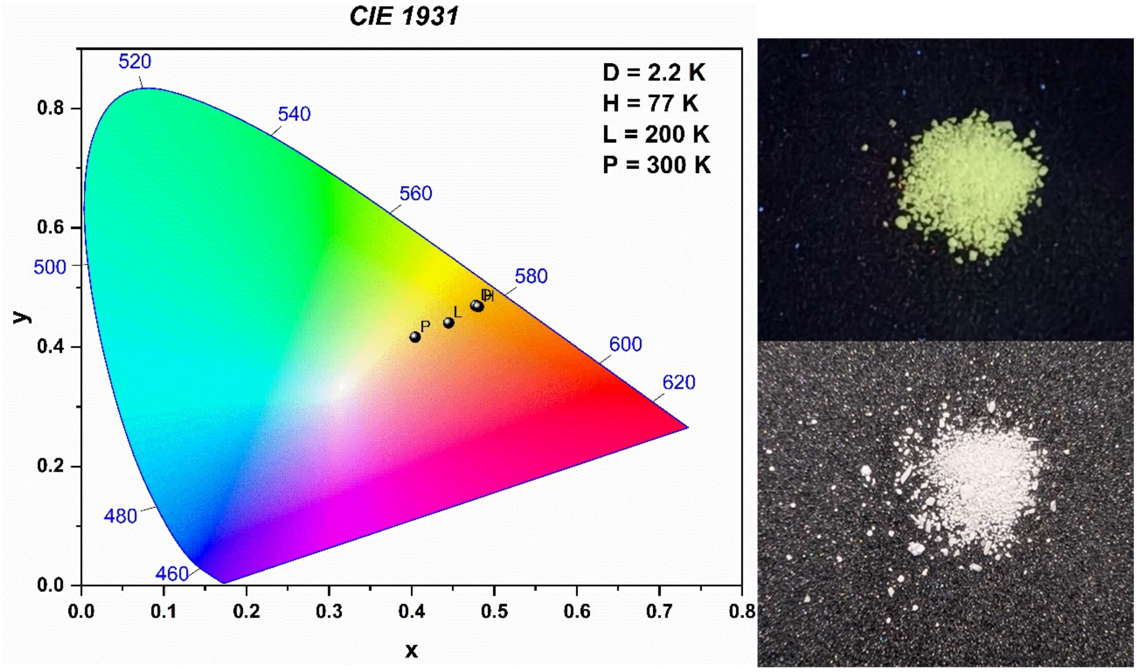

Figure S 82. CIE coordinates of Dy doped **C-2$_d$:Dy** at different temperatures 2.4 K (D), 77 K (H), 180 K (L) and 300 K (P) obtained upon excitation at 310 nm. CIE coordinates (black dot in the figure) are D, X = 0.477 and Y = 0.470; H, X = 0.481 and Y = 0.468; L, X = 0.445 and Y = 0.441; P, X = 0.404 and Y = 0.417. Images of the dysprosium doped BiO-NC under ambient condition and irradiation with daylight (top) and under UV light (256 nm, bottom) are included for visualization.

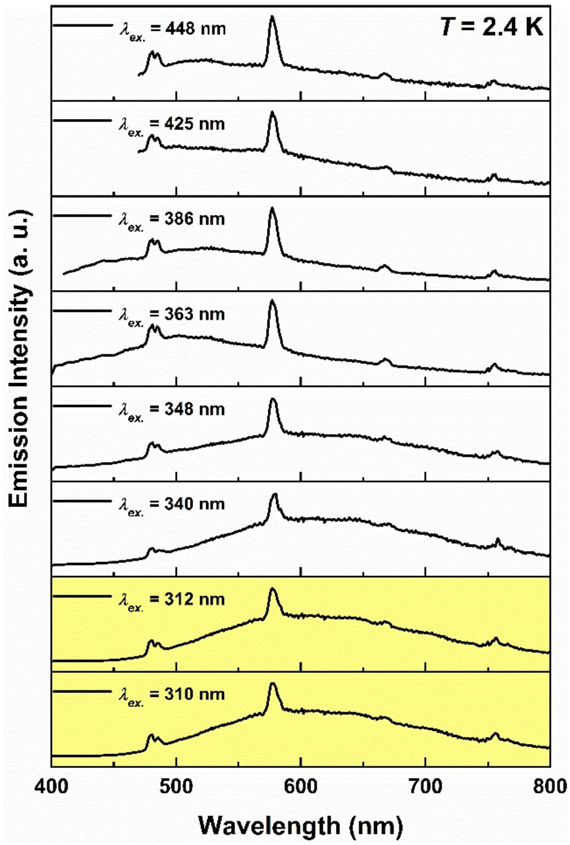

Figure S 83. Excitation-wavelength-dependent emission characteristics of **C-2$_d$:Dy** cluster at 2.4 K, yellow marked are excited from Bi^{3+}-based excitation.

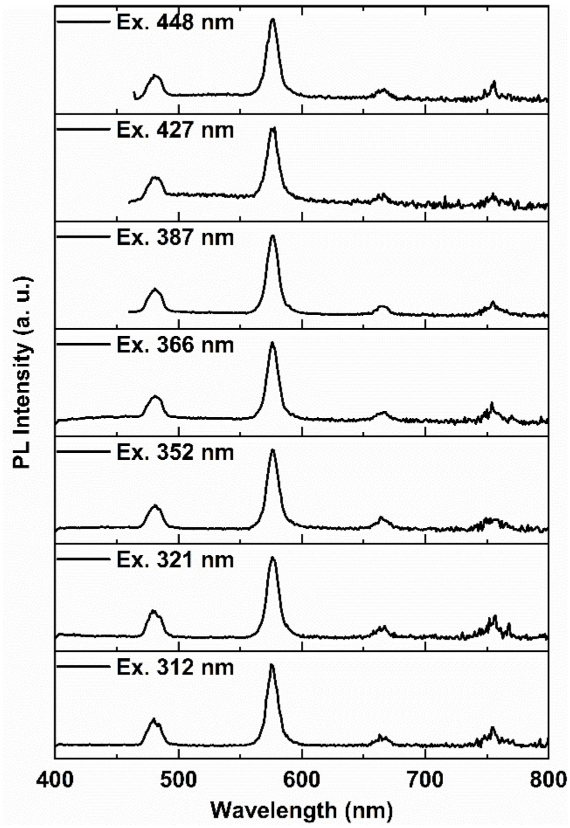

Figure S 84. Excitation-wavelength-dependent PL spectra of **C-2$_d$:Dy** at T = 300 K.

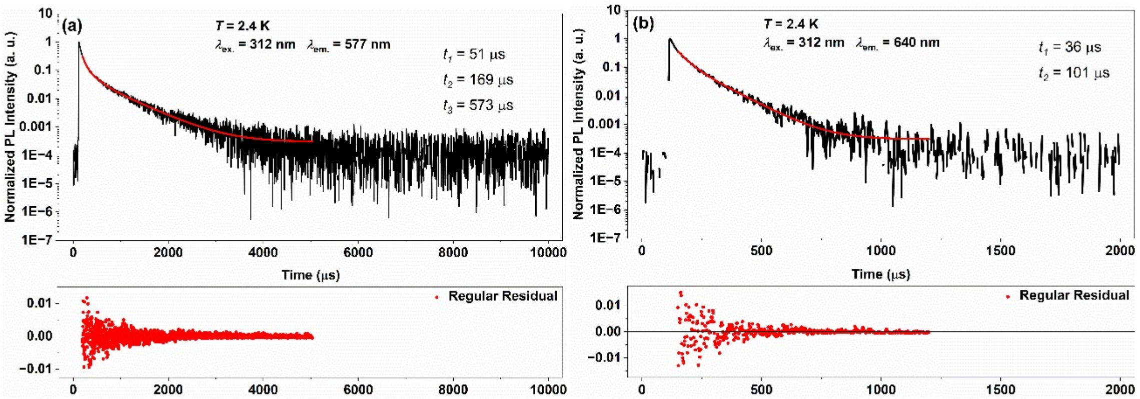

Figure S 85. Emission decay profiles of **C-2$_d$:Dy** at 2.4 K und excitation with $\lambda_{ex.}$ = 312 nm (a) at $\lambda_{em.}$ = 577 nm and (b) at $\lambda_{em.}$ = 640 nm

Table S 43. PL lifetimes of BiO-NC **C-2$_d$:Dy** measured at 2.4 K at different $\lambda_{em.}$.

Wavelength	Lifetimes (τ/µs)		
	τ_1	τ_2	τ_3
$\lambda_{em.}$ = 577 nm	51 ± 2	169 ± 9	573 ± 15
$\lambda_{em.}$ = 640 nm	36 ± 2	101 ± 4	-

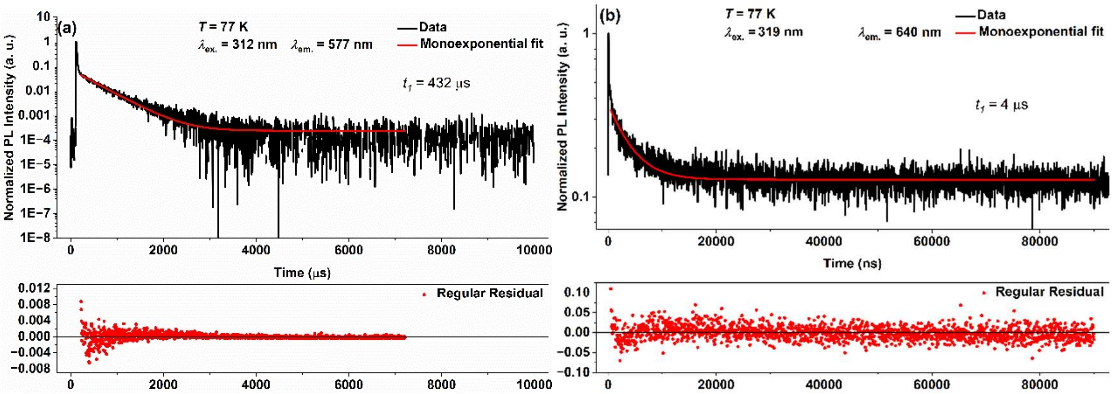

Figure S 86. PL decay spectra of BiO-NC **C-2$_d$:Dy** at 77 K (a) under excitation with $\lambda_{ex.}$ = 312 nm at an emission wavelength of $\lambda_{em.}$ = 577 nm and (b) under excitation with $\lambda_{ex.}$ = 319 nm (LED) at an emission wavelength of $\lambda_{em.}$ = 460 nm with the respective monoexponential fit.

Table S 44. PL lifetimes of BiO-NC **C-2$_d$:Dy** measured at 77 K at different $\lambda_{em.}$.

Wavelength	Lifetimes (τ/µs)
	τ_1
$\lambda_{em.}$ = 577 nm	432 ± 5
$\lambda_{em.}$ = 640 nm	3.6 ± 1

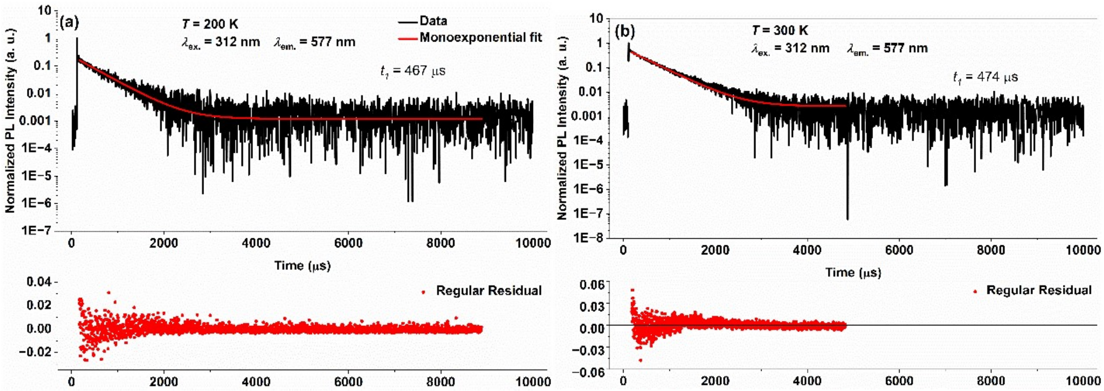

Figure S 87. PL decay spectra of BiO-NC **C-2$_d$:Dy** under excitation with $\lambda_{ex.}$ = 312 nm at an emission wavelength of $\lambda_{em.}$ = 577 nm at different temperatures (a) 200 K and (b) 300 K with the respective monoexponential fit.

Table S 45. PL lifetimes of BiO-NC **C-2$_d$:Dy** measured at different Temperatures at different $\lambda_{em.}$ = 577 nm under $\lambda_{ex.}$ = 577 nm.

Temperature	Lifetimes (τ/µs)
	τ_1
200 K	467 ± 3
300 K	474 ± 2

Magnetic Susceptibility Measurements of BiO-NCs:

Table S 46. $\chi_M T$ values from ZFC and FC susceptibility measurements of BiO-NC **C-2$_d$:Gd** at 0.05 T.

T (K)	**C-2$_d$:Gd** $\chi_M T$ (cm^{-3}·K·mol^{-1})	T (K)	**C-2$_d$:Gd** $\chi_M T$ (cm^{-3}·K·mol^{-1})
2.00	3.71	300.02	5.10
3.00	4.02	289.37	5.10
4.03	4.21	279.98	5.08
4.99	4.25	270.67	5.07
6.01	4.40	260.87	5.14
7.00	4.49	250.18	5.07
8.01	4.56	240.80	5.07
9.00	4.60	229.77	5.07
10.00	4.64	220.53	5.06
11.02	4.67	209.64	5.06
12.00	4.70	199.88	5.05
13.02	4.73	189.79	5.05
14.03	4.76	179.62	5.05
15.00	4.77	169.66	5.04
16.00	4.79	159.66	5.04
17.00	4.80	149.68	5.04
18.00	4.81	139.70	5.04
19.00	4.82	129.77	5.10
20.00	4.83	119.79	5.03
30.00	4.90	109.83	5.05
40.00	4.93	99.86	5.03
50.02	4.96	89.92	5.03
60.04	4.97	79.97	5.02
70.10	4.98	70.02	5.01
80.13	4.99	60.05	4.99
90.16	5.00	50.04	4.97
100.14	5.03	40.01	4.93
110.15	5.00	30.00	4.90
120.19	5.01	19.99	4.83
130.26	5.02	19.04	4.82
140.29	5.02	18.01	4.81
150.34	5.02	17.00	4.80
160.37	5.03	16.00	4.79
170.41	5.03	15.00	4.77
180.45	5.04	14.08	4.76
190.49	5.04	13.04	4.73
200.52	5.05	12.02	4.69
210.55	5.05	11.03	4.66
220.59	5.08	10.01	4.61
230.45	5.08	9.01	4.57
240.44	5.09	8.01	4.52
250.32	5.09	7.01	4.46
260.44	5.09	6.01	4.38
270.44	4.79	5.01	4.26
280.45	5.10	4.00	4.14
290.43	5.08	3.00	3.89
300.43	--	1.99	3.67

Table S 47. $\chi_M T$ values from ZFC and FC susceptibility measurements of BiO-NC **C-2$_d$:Dy** at 0.05 T.

T (K)	**C-2$_d$:Dy** $\chi_M T$ (cm^{-3}·K·mol^{-1})	T (K)	**C-2$_d$:Dy** $\chi_M T$ (cm^{-3}·K·mol^{-1})
2.00	7.20	300.02	10.00
3.00	7.41	289.34	10.00
4.01	7.54	280.01	9.86
4.99	7.49	270.69	9.92
6.00	7.65	261.52	9.90
7.01	7.77	249.98	9.89
8.00	7.85	240.55	9.87
9.01	7.92	230.21	9.85
10.01	7.97	220.31	9.83
11.02	8.02	209.83	9.81
12.00	8.07	199.86	9.78
13.01	8.13	189.71	9.75
14.02	8.18	179.59	9.81
15.00	8.22	169.60	9.70
16.00	8.26	159.63	9.68
17.00	8.30	149.66	9.65
18.00	8.33	139.72	9.61
19.00	8.36	129.76	9.57
20.00	8.39	119.78	9.53
30.00	8.65	109.83	9.50
40.00	8.82	99.85	9.46
50.02	9.00	89.91	9.41
60.06	9.12	80.00	9.34
70.10	9.22	70.01	9.25
80.13	9.30	60.05	9.14
90.16	9.37	50.05	9.01
100.19	9.45	40.01	8.84
110.22	9.38	30.00	8.65
120.18	9.49	19.99	8.38
130.27	9.53	19.04	8.35
140.31	9.57	18.00	8.31
150.34	9.60	17.00	8.28
160.39	9.64	16.00	8.24
170.43	9.66	15.00	8.20
180.48	9.70	14.06	8.15
190.50	9.73	13.03	8.10
200.52	9.75	12.01	8.04
210.56	9.78	11.03	7.97
220.45	9.82	10.00	7.90
230.43	9.84	9.00	7.84
240.44	9.86	8.00	7.77
250.44	9.88	7.01	7.70
260.34	9.90	6.01	7.60
270.73	9.93	5.01	7.48
280.45	9.95	4.00	7.39
290.45	9.98	3.00	7.18
300.43	--	1.98	7.19

NMR Studies on Methacrylate Functionalized BiO-NCs:

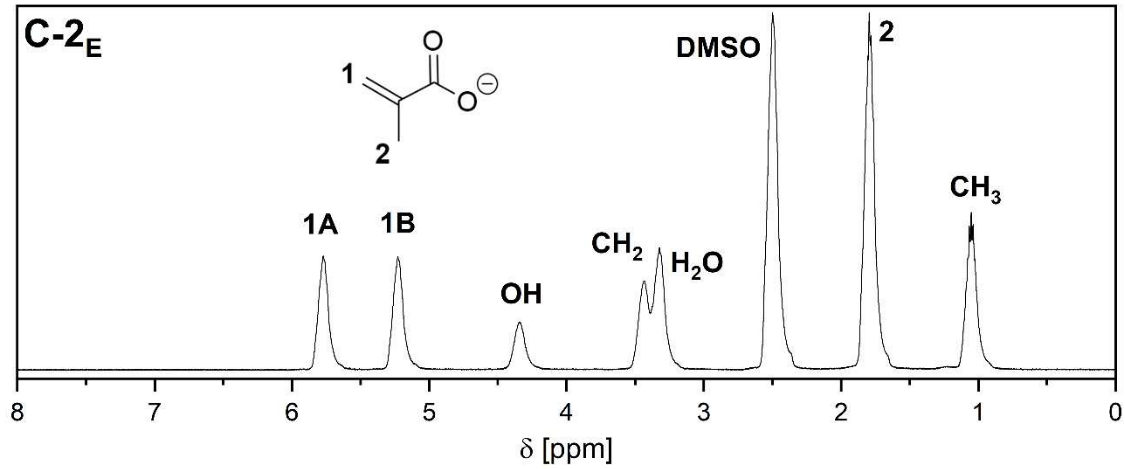

Figure S 88. ^{1}H NMR spectra (500.3 MHz, 298 K) of **C-2$_E$** in dmso-d$_6$. The signals marked with OH, CH_2, CH_3 belong to the ethanol signals which coordinates to the cluster.

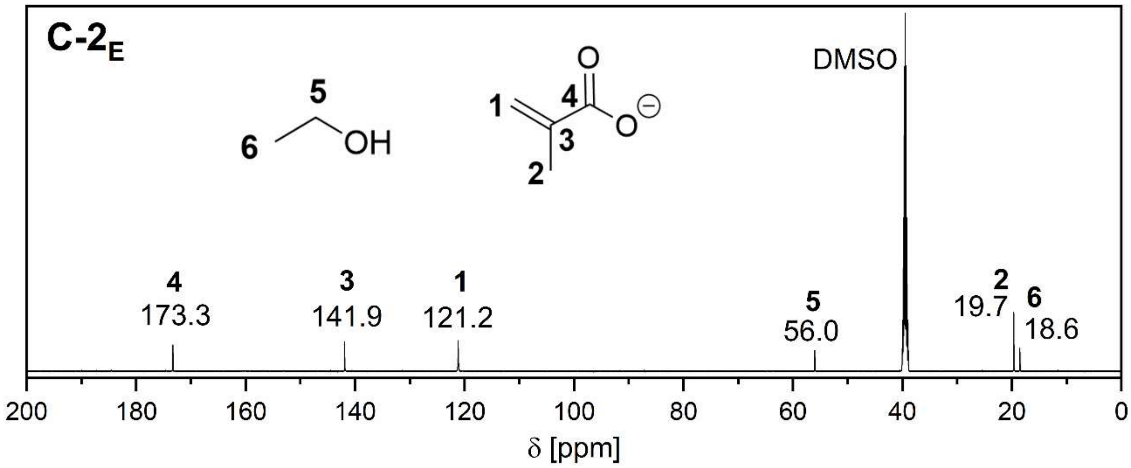

Figure S 89. ^{13}C NMR spectra (125.8 MHz, 298 K) of **C-2$_E$** in dmso-d$_6$, signals 5 and 6 belong to ethanol.

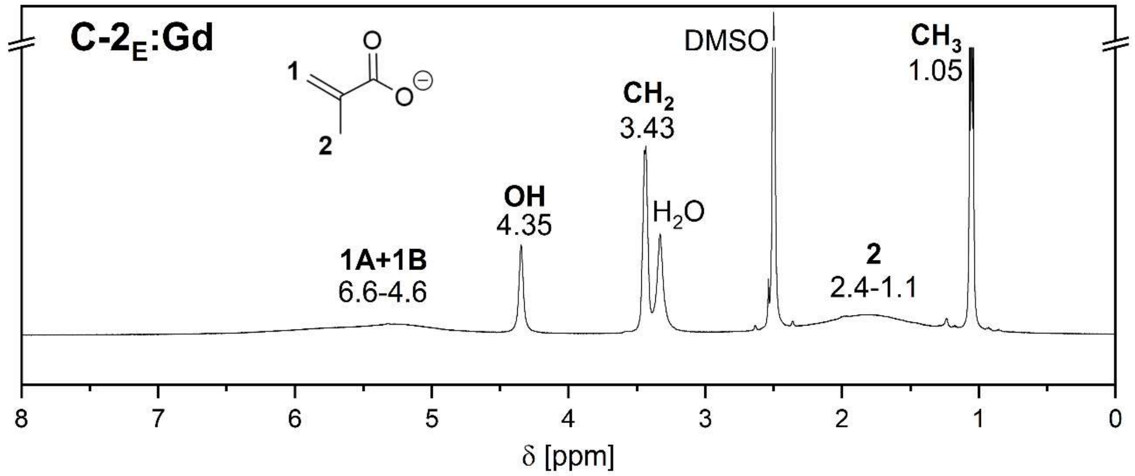

Figure S 90. ^{1}H NMR spectra (500.3 MHz, 298 K) of **C-2$_E$:Gd** in dmso-d$_6$. The signals marked with OH, CH_2, CH_3 belong to the ethanol signals which coordinates to the cluster.

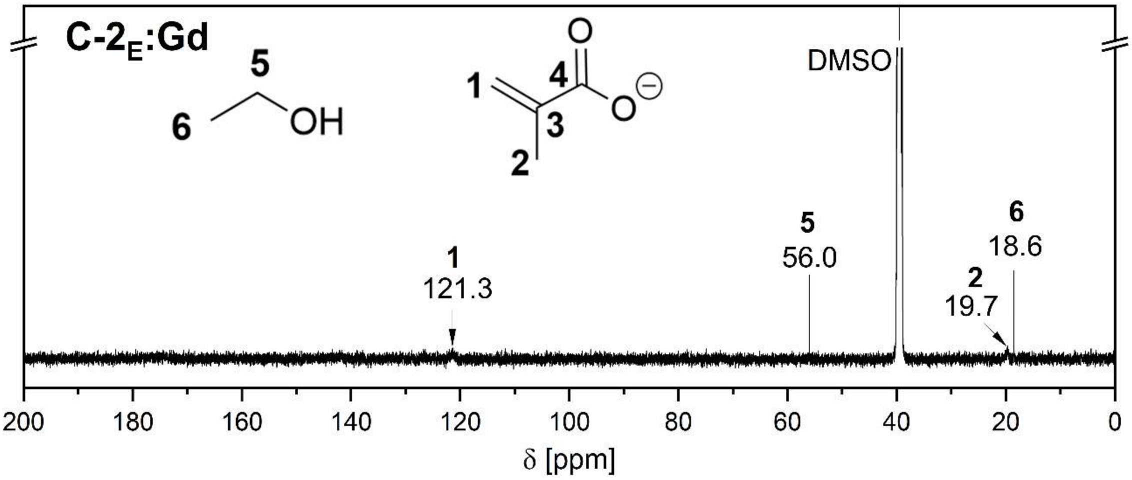

Figure S 91. ^{13}C NMR spectra (125.8 MHz, 298 K) of **C-2$_E$:Gd** in dmso-d$_6$, signals 5 and 6 belong to ethanol. Note that not all signals of the $^{-}$OMc ligands are present as a result of the Gd^{3+} dopant, the concentration and the respective lower intensity of quaternary carbons.

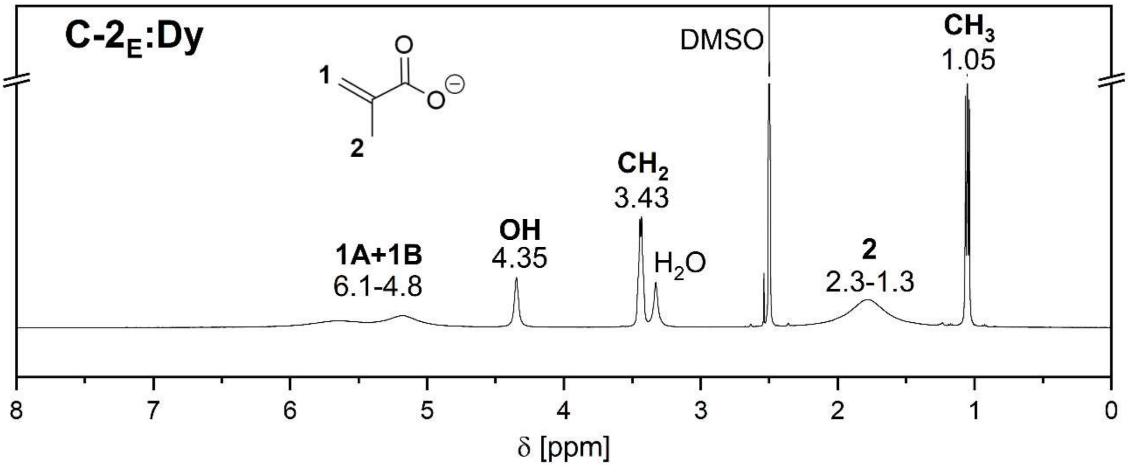

Figure S 92. ^{1}H NMR spectra (500.3 MHz, 298 K) of **C-2$_E$:Dy** in dmso-d$_6$. The signals marked with OH, CH_2, CH_3 belong to the ethanol signals which coordinates to the cluster.

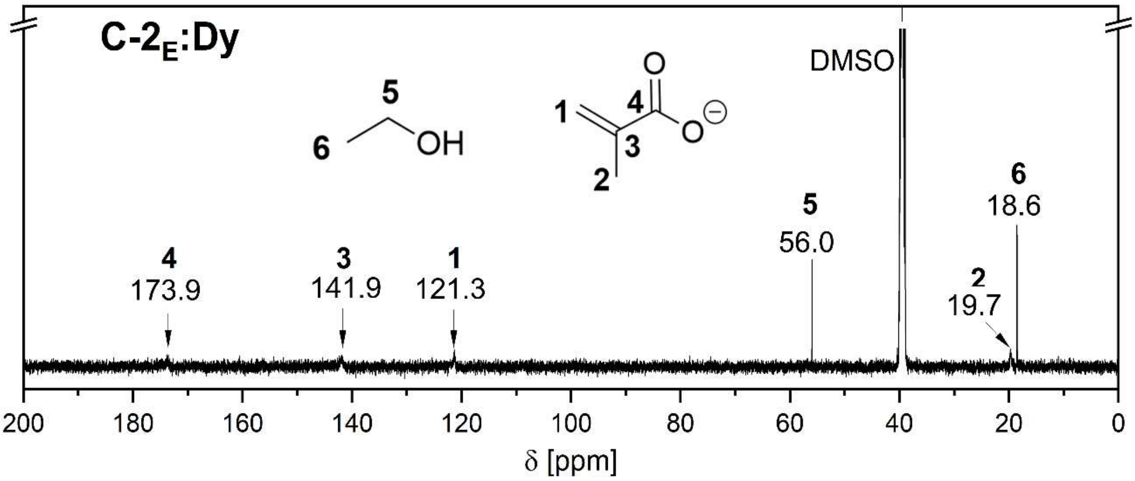

Figure S 93. ^{13}C NMR spectra (125.8 MHz, 298 K) of **C-2$_E$:Dy** in dmso-d$_6$, Signals 5 and 6 belong to ethanol.

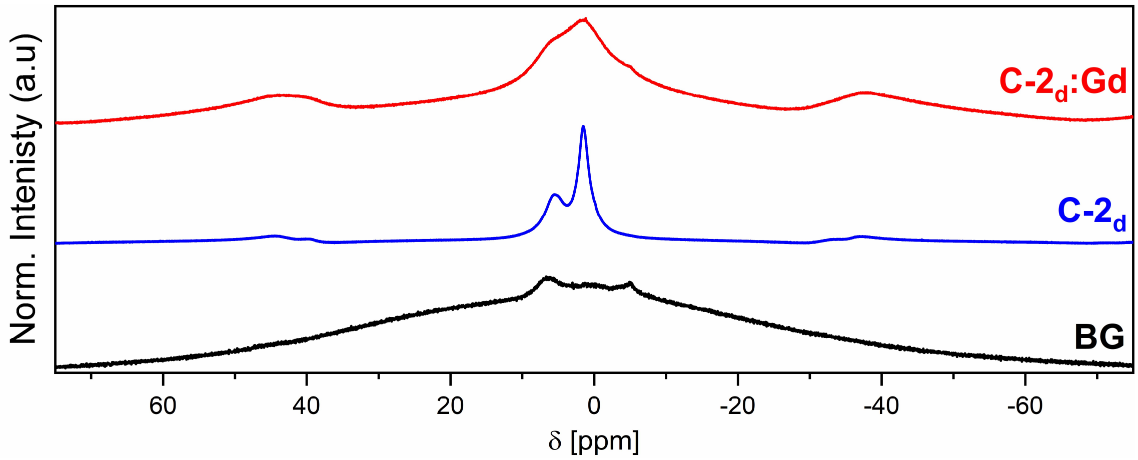

Figure S 94. Normalized ^{1}H CP MAS NMR spectra (15 KHz, 298 K) of **C-2$_d$:Gd** (red), **C-2$_d$** (blue), and sample background (BG, black).

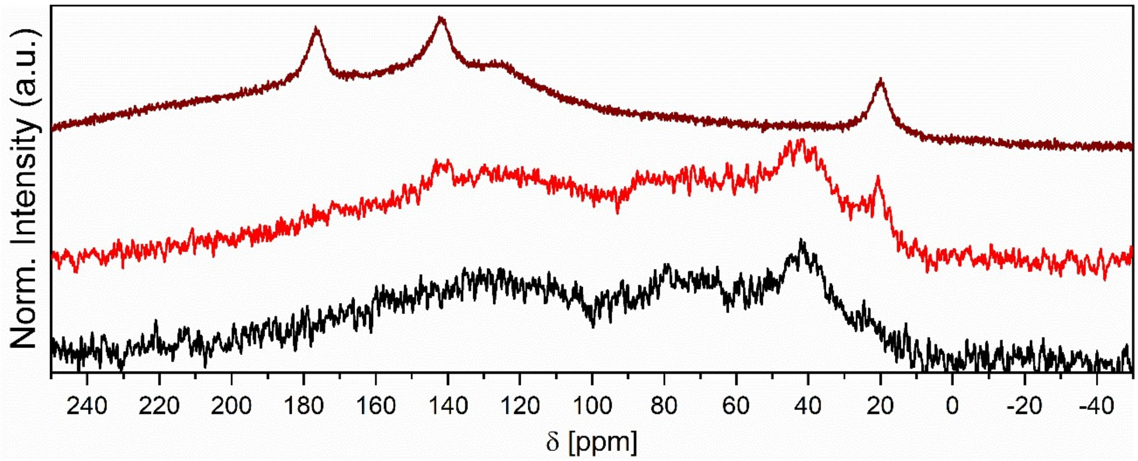

Figure S 95. $^{13}C\{^1H\}$ CP MAS NMR (15 KHz, 298 K) of the sample background (black), **C-2$_d$:Gd** (red) and $^{13}C\{^1H\}$ MAS NMR (15 KHz, 298 K) of **C-2$_d$:Gd** (dark red) measured without CP.

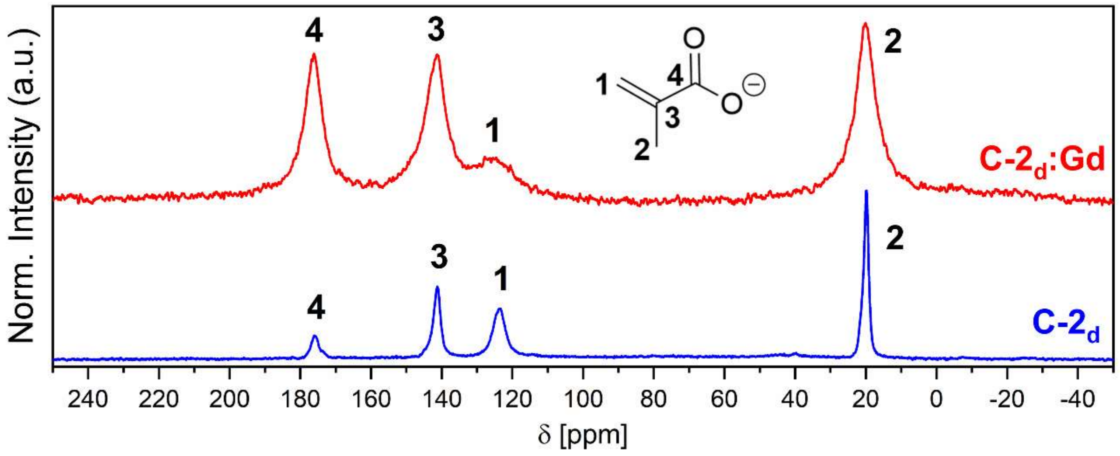

Figure S 96. $^{13}C\{^1H\}$ MAS NMR (15 KHz, 298 K) of **C-2$_d$:Gd** (red) measured with background cancelling and without CP (top) and $^{13}C\{^1H\}$ CP MAS NMR (15 KHz, 298 K) of **C-2$_d$** (blue).

DNP Spectra of **C-1:Gd:**

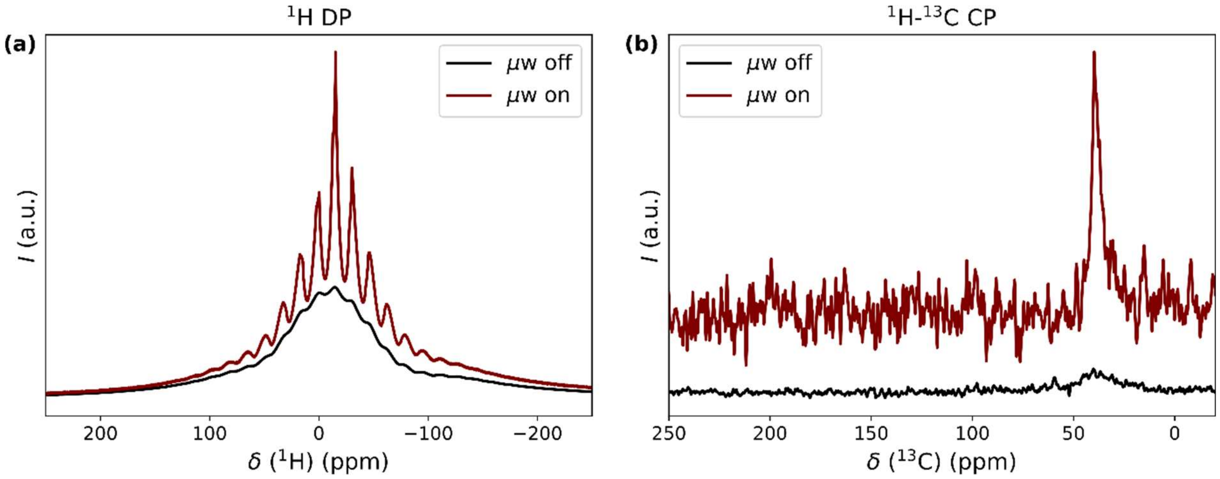

Figure S 97. ^{1}H DP (a) and ^{1}H-^{13}C CPMAS spectra of pure **C-1:Gd** at 100 K and 6 kHz MAS frequency with (red) and without (black) microwave irradiation (263 GHz) at the positive proton solid effect DNP matching condition. In both cases the enhancement factor could be determined to be approximately 10. For the CP spectra, 128 scans were accumulated with microwave irradiation and 8192 scans without, explaining the difference in signal-to-noise ratio.

G-band EPR Spectra of **C-2d:Gd:**

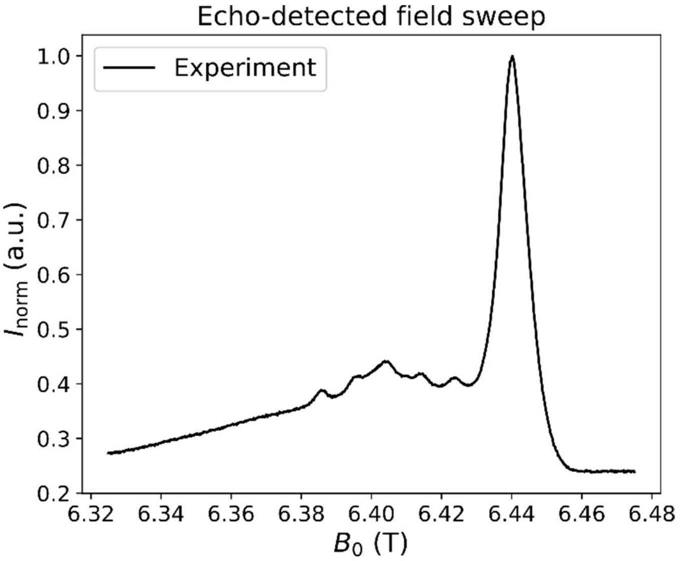

Figure S 98. G-band (180 GHz) echo detected field sweep EPR spectrum of **C-2$_d$:Gd** powder at 10 K.

TGA Analysis of Gd doped BiO-NCs:

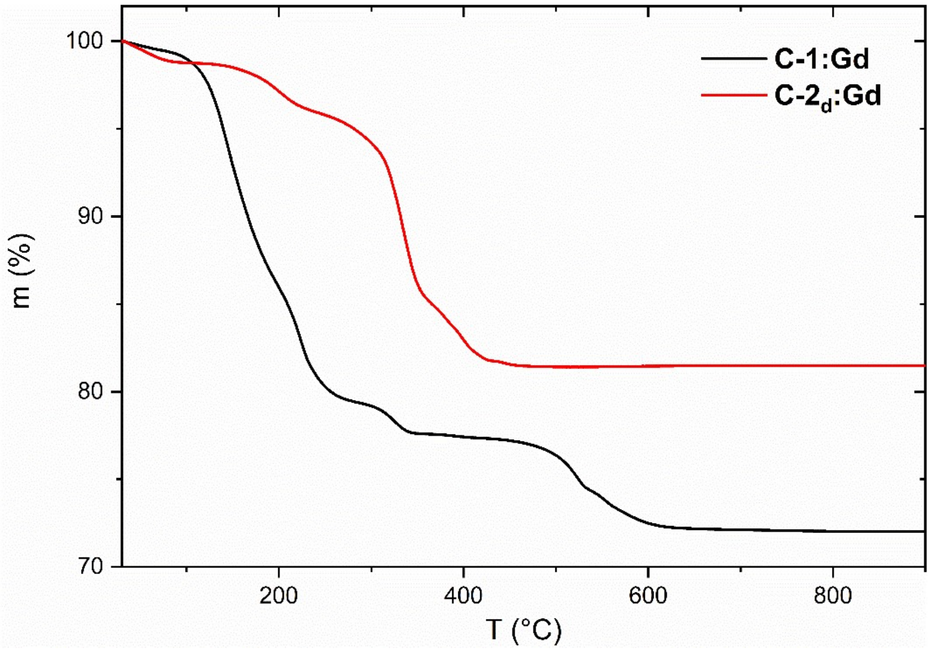

Figure S 99. TGA of the BiO-NCs **C-1:Gd** and **C-2$_d$:Gd** between 30 °C and 900°C with a heat rate of 10 K/min under Air-flow.

References:

1. Esteban-Gómez, D.; Büldt, L. A.; Pérez-Lourido, P.; Valencia, L.; Seitz, M.; Platas-Iglesias, C., Understanding the Optical and Magnetic Properties of Ytterbium(III) Complexes. *Inorg. Chem.* **2019,** *58* (6), 3732-3743. DOI: 10.1021/acs.inorgchem.8b03354

2. Arun Kumar, S.; Senthilselvan, J., Photoluminescence and thermoluminescence investigations of Yb:YAG nanoparticles by dual-surfactant functionalization and microwave calcination method. *Mater. Chem. Phys.* **2018,** *217*, 334-349. DOI: 10.1016/j.matchemphys.2018.06.053

3. Azuah, R. T.; Kneller, L. R.; Qiu, Y.; Tregenna-Piggott, P. L.; Brown, C. M.; Copley, J. R.; Dimeo, R. M., DAVE: a comprehensive software suite for the reduction, visualization, and analysis of low energy neutron spectroscopic data. *J. Res. Natl. Inst. Stand. Technol.* **2009,** *114* (6), 341. DOI: 10.6028/jres.114.025

4. Rohrer, M.; Brügmann, O.; Kinzer, B.; Prisner, T. F., High-field/high-frequency EPR spectrometer operating in pulsed and continuous-wave mode at 180 GHz. *Appl. Magn. Reson.* **2001,** *21* (3), 257-274. DOI: 10.1007/BF03162406

5. Miersch, L.; Schlesinger, M.; Troff, R. W.; Schalley, C. A.; Rüffer, T.; Lang, H.; Zahn, D.; Mehring, M., Hydrolysis of a Basic Bismuth Nitrate-Formation and Stability of Novel Bismuth Oxido Clusters. *Chem. Eur. J.* **2011,** *17* (25), 6985-6990. DOI: 10.1002/chem.201100673

6. Miersch, L.; Rüffer, T.; Mehring, M., Organic–inorganic hybrid materials starting from the novel nanoscaled bismuth oxido methacrylate cluster $[Bi_{38}O_{45}(OMc)_{24}(DMSO)_9]\cdot 2DMSO\cdot 7H_2O$. *Chem. Commun.* **2011,** *47* (22), 6353-6355. DOI: 10.1039/c1cc11299f

7. Morgenstern, A.; Thomas, R.; Sharma, A.; Weber, M.; Selyshchev, O.; Milekhin, I.; Dentel, D.; Gemming, S.; Tegenkamp, C.; Zahn, D. R. T.; Mehring, M.; Salvan, G., Deposition of Nanosized Amino Acid Functionalized Bismuth Oxido Clusters on Gold Surfaces. *Nanomater.* **2022,** *12* (11), 1815-1832. DOI: 10.3390/nano12111815

8. Thomas, R.; Nguyen, T. N. H.; Weber, M.; Rüffer, T.; Göhler, F.; Deka, A.; Pöppl, A.; Seyller, T.; Tegenkamp, C.; Mehring, M., Atomically precise bismuth oxido nanoclusters: cerium doping for optical modification and supramolecular self-assembly on Au(111). *Nanoscale* **2025,** *17*, 18291-18304. DOI: 10.1039/D5NR02346G

9. Thomas, R.; Kuppusamy, S. K.; Rüffer, T.; Weber, M.; Ruben, M.; Mehring, M., Europium Doped Atomically Precise Bismuth Oxido Nanoclusters as Molecular Building Blocks for Photoluminescent Hybrid Materials. *ChemRxiv* **2026,** *2026* (0224). DOI: 10.26434/chemrxiv.15000367/v1
10. Miersch, L.; Rüffer, T.; Schlesinger, M.; Lang, H.; Mehring, M., Hydrolysis Studies on Bismuth Nitrate: Synthesis and Crystallization of Four Novel Polynuclear Basic Bismuth Nitrates. *Inorg. Chem.* **2012,** *51* (17), 9376-9384. DOI: 10.1021/ic301148p
11. Weber, M.; Rüffer, T.; Speck, F.; Göhler, F.; Weimann, D. P.; Schalley, C. A.; Seyller, T.; Lang, H.; Mehring, M., From a Cerium-Doped Polynuclear Bismuth Oxido Cluster to *β*-Bi_2O_3:Ce. *Inorg. Chem.* **2020,** *59* (6), 3353-3366. DOI: 10.1021/acs.inorgchem.9b03240
12. Cruickshank, D. W. J., The accuracy of electron-density maps in X-ray analysis with special reference to dibenzyl. *Acta Crystallogr. Sect. B* **1949,** *2* (2), 65-82. DOI: 10.1107/S0365110X49000175
13. Klimesz, B.; Ryba-Romanowski, W.; Lisiecki, R., Spectroscopic and Thermographic Qualities of Praseodymium-Doped Oxyfluorotellurite Glasses. *Molecules* **2024,** *29* (13), 3041. DOI: 10.3390/molecules29133041
14. Sharma, S. K.; Behm, T.; Köhler, T.; Beyer, J.; Gloaguen, R.; Heitmann, J., Library of UV-Visible Absorption Spectra of Rare Earth Orthophosphates, $LnPO_4$ (Ln = La-Lu, except Pm). *Crystals* **2020,** *10* (7), 593. DOI: 10.3390/cryst10070593
15. Nawrocki, P. R.; Sørensen, T. J., Optical spectroscopy as a tool for studying the solution chemistry of neodymium(iii). *Phys. Chem. Chem. Phys.* **2023,** *25* (29), 19300-19336. DOI: 10.1039/D3CP02033A
16. Mortensen, S. S.; Marciniak Nielsen, M. A.; Nawrocki, P.; Sørensen, T. J., Electronic Energy Levels and Optical Transitions in Samarium(III) Solvates. *J. Phys. Chem. A.* **2022,** *126* (46), 8596-8605. DOI: 10.1021/acs.jpca.2c04793
17. Wang, R.; Meng, X.; Yin, F.; Feng, Y.; Qin, G.; Qin, W., Heavily erbium-doped low-hydroxyl fluorotellurite glasses for 2.7 µm laser applications. *Opt. Mater. Express* **2013,** *3* (8), 1127-1136. DOI: 10.1364/OME.3.001127
18. Singh, V.; Seshadri, M.; Taikar, D.; Dhoble, S. J.; Yadav, R. S., Optical transitions and radiative properties of green emitting Ho^{3+}:YVO_4 phosphor. *RSC Adv.* **2023,** *13* (6), 3592-3601. DOI: 10.1039/D2RA06287A
19. Yu, D. C.; Huang, X. Y.; Ye, S.; Zhang, Q. Y.; Wang, J., A sequential two-step near-infrared quantum splitting in Ho^{3+} singly doped $NaYF_4$. *AIP Advances* **2011,** *1* (4). DOI: 10.1063/1.3666981
20. Blackburn, O. A.; Tropiano, M.; Sørensen, T. J.; Thom, J.; Beeby, A.; Bushby, L. M.; Parker, D.; Natrajan, L. S.; Faulkner, S., Luminescence and upconversion from thulium(iii) species in solution. *Phys. Chem. Chem. Phys.* **2012,** *14* (38), 13378-13384. DOI: 10.1039/C2CP42228J
21. Kuppusamy, S. K.; Pachl, C.; Jing, Z.; Paul, S.; Heinrich, B.; Fuhr, O.; Klyatskaya, S.; Wernsdorfer, W.; Powell, A. K.; Fink, K.; Ruben, M., Ligand-field symmetry and magneto-optical correlations in a luminescent Dy(III) single-molecule magnet. *Dalton Trans.* **2026**. DOI: 10.1039/D6DT00081A
